# Computationally Guided Molecular Design to Minimize the LE/CT Gap in D‐π‐A Fluorinated Triarylboranes for Efficient TADF via D and π‐Bridge Tuning

**DOI:** 10.1002/adfm.202002064

**Published:** 2020-06-02

**Authors:** Ayush K. Narsaria, Florian Rauch, Johannes Krebs, Peter Endres, Alexandra Friedrich, Ivo Krummenacher, Holger Braunschweig, Maik Finze, Jörn Nitsch, F. Matthias Bickelhaupt, Todd B. Marder

**Affiliations:** ^1^ Department of Theoretical Chemistry Amsterdam Institute of Molecular and Life Sciences (AIMMS) and Amsterdam Center for Multiscale Modeling (ACMM) Vrije Universiteit Amsterdam De Boelelaan 1083 Amsterdam NL‐1081 HV The Netherlands; ^2^ Institute for Inorganic Chemistry Julius‐Maximilians‐Universität Würzburg Am Hubland Würzburg D‐97074 Germany; ^3^ Institute for Sustainable Chemistry & Catalysis with Boron Julius‐Maximilians‐Universität Würzburg Am Hubland Würzburg D‐97074 Germany; ^4^ Institute for Molecules and Materials (IMM) Radboud University Heyendaalseweg 135 Nijmegen NL‐6525 AJ The Netherlands

**Keywords:** boron, charge transfer, delayed fluorescence, organic light‐emitting diodes, singlet–triplet gap quantum efficiency

## Abstract

In this combined experimental and theoretical study, a computational protocol is reported to predict the excited states in D‐π‐A compounds containing the B(^F^Xyl)_2_ (^F^Xyl = 2,6‐bis(trifluoromethyl)phenyl) acceptor group for the design of new thermally activated delayed fluorescence (TADF) emitters. To this end, the effect of different donor and π‐bridge moieties on the energy gaps between local and charge‐transfer singlet and triplet states is examined. To prove this computationally aided design concept, the D‐π‐B(^F^Xyl)_2_ compounds **1**–**5** were synthesized and fully characterized. The photophysical properties of these compounds in various solvents, polymeric film, and in a frozen matrix were investigated in detail and show excellent agreement with the computationally obtained data. Furthermore, a simple structure–property relationship is presented on the basis of the molecular fragment orbitals of the donor and the π‐bridge, which minimize the relevant singlet–triplet gaps to achieve efficient TADF emitters.

## Graphical abstract

A combination of experiment and theory leads to new insights into accurately and cost‐efficiently predicting the phenomenon of thermally activated delayed fluorescence. The calculations guide syntheses in optimizing the energy gaps between the states of different multiplicity involved in order to develop new efficient emitter materials.

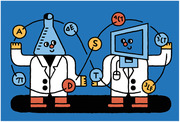

## 1 Introduction

**Scheme 1:**
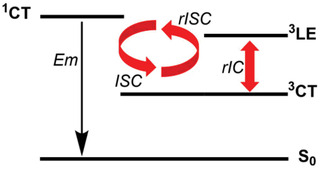
Dynamic two‐step TADF mechanism for a D‐A molecule. Em: emission; ISC: intersystem crossing; rISC: reverse intersystem crossing; and rIC: reverse internal conversion.

Achieving 100% internal quantum efficiency (IQE) in organic light‐emitting diodes (OLEDs)^[^ ^1^ ^]^ has been a challenging issue for several years. Spin‐statistics dictate that electrons and holes recombine to generate 25% singlet and 75% triplet excitons.^[^ ^2^ ^]^ Conventional fluorophores utilize only the singlet excitons leading to low IQE. Phosphorescent materials, on the other hand, are able to achieve, in theory, 100% IQE,^[^ ^3^ ^]^ but require the involvement of expensive and less abundant heavy metal atoms essential for introducing large spin–orbit coupling to access rapid intersystem crossing.^[^ ^3^, ^4^ ^]^ In 2012, Adachi and co‐workers demonstrated that organic molecules can undergo an alternative mechanism known as thermally activated delayed fluorescence (TADF). In fact, TADF was first reported by Perrin in 1929,^[^ ^5^ ^]^ and later investigated by others in the 20th century (so‐called E‐type fluorescence),^[^ ^6^ ^]^ such that organic TADF emitters can harvest both singlet and triplet excitons and thus, potentially, enhance the IQE to 100%.^[^ ^7^ ^]^ TADF is a unimolecular process in which the triplet state is thermally up‐converted via reverse intersystem crossing (rISC) back to the singlet state.^[^ ^8^ ^]^ The efficiency of rISC determines the overall performance of the TADF process and is enabled by a small singlet–triplet energy splitting (Δ*E*_ST_) such that the thermal energy at ambient temperature is sufficient to upconvert the triplet back to the singlet. In principle, a small Δ*E*_ST_ can be achieved by spatially separating the highest occupied molecular orbital (HOMO) and lowest unoccupied molecular orbital (LUMO) in donor–acceptor (D‐A) molecules, resulting in a small overlap of their wavefunctions and, thereby, a small exchange energy, *K* (in the Hartree–Fock approximation: Δ*E*_ST_ = 2*K*).^[^ ^9^ ^]^ In the same way, twisted D‐A molecules, in which the donor and acceptor orbitals are almost orthogonal, display small values for *K*. Another strategy is to enhance the spin–orbit coupling $〈{\text{T}}_{1}|{\hat{H}}_{\text{SO}}|{\text{S}}_{1}〉$ between pure spin states T_1_ and S_1_, where ${\hat{H}}_{\text{SO}}$ = ξ$\hat{L}•\hat{S}$ and $\hat{L}$ and $\hat{S}$ are total orbital and spin angular momentum, respectively, in order to increase the rate of the ISC and rISC processes.^[^ ^10^ ^]^ However, generally, the spin–orbit coupling is very small in organic molecules (typically < 1 cm^−1^),^[^ ^11^ ^]^ in contrast to that in phosphors containing heavy metal atoms. To maximize the rISC efficiency, several researchers elucidated the crucial role of high‐energy triplet (local‐excited) states to accelerate the rISC process. Based on carefully designed spectroscopic and quantum dynamics experiments, they elucidated an efficient dynamic two‐step rISC mechanism (**Scheme** **1**).^[^ ^11^, ^12^ ^]^

First, local‐excited and charge‐transfer states (^3^LE and ^3^CT) are coupled via nonadiabatic coupling (reverse internal conversion: rIC), the strength of which depends on the size of the vibronic coupling (VC) and the corresponding energy gap (Equation (1)).^[^ ^12e^ ^]^ Subsequently, a second‐order perturbation term couples ^3^CT and ^1^CT states mediated by the intermediate ^3^LE state (Equation (2))^[^ ^12e^ ^]^

(1) $$\;k_{\text{rIC}}=\;\frac{2\pi }{\hbar }{\left|\left\langle {}^{3}\psi_{\text{LE}}\left|{\hat{H}}_{\text{vib}}\right|{\;}^{3}\psi_{\text{CT}}\right\rangle \right|}^{2}\;\times \;\delta \left({}_{}^{3}E_{\text{LE}}-{\;}_{}^{3}E_{\text{CT}}\right)$$

(2) $$k_{\text{rISC}}=\;\frac{2\pi }{\hbar }{\left|\frac{\left\langle {}^{1}\psi_{\text{CT}}\left|{\hat{H}}_{\text{SOC}}\right|{\;}^{3}\psi_{\text{LE}}\right\rangle \left\langle {\;}^{3}\psi_{\text{LE}}\left|{\hat{H}}_{\text{vib}}\right|{\;}^{3}\psi_{\text{CT}}\right\rangle }{\delta \left({}_{}^{3}E_{\text{LE}}-{\;}_{}^{3}E_{\text{CT}}\right)}\right|}^{2}\times \delta \left({}_{}^{3}E_{\text{CT}}-{\;}_{}^{1}E_{\text{CT}}\right)$$

Thus, in order to design an efficient rISC process it is of paramount importance to decrease (at least) *two* gaps, the energy difference between ^3^LE and ^1^CT (Δ$E_{1_{\text{CT}}-3_{\text{LE}}}$), and ^1^CT and ^3^CT (Δ$E_{1_{\text{CT}}-3_{\text{CT}}}$), respectively,^[^ ^9c^ ^]^ instead of focusing only on the gap between S_1_ and T_1_. In many cases, the lowest ^1^CT and ^3^CT states are described by the transition from a donor (D) to an acceptor (A) which, in the case of twisted molecules (large dihedral angle between the donor and acceptor moieties), lie close in energy leading to a small Δ$E_{1_{\text{CT}}-3_{\text{CT}}}$.^[^ ^13^ ^]^

One class of compounds that has successfully been employed for TADF is triarylboranes.^[^ ^14^ ^]^ Three‐coordinate boron can be used as an acceptor moiety due to the vacant p_z_ orbital perpendicular to its plane. Triarylboranes represent a well‐researched acceptor class^[^ ^15^ ^]^ and have been employed for a plethora of different applications such as linear^[^ ^16^ ^]^ and nonlinear^[^ ^17^ ^]^ optics, sensors,^[^ ^18^ ^]^ and OLEDs.^[^ ^14^, ^19^ ^]^ In all of these applications, it is important to note that, if no explicit reactivity of the boron center toward nucleophiles is desired, electronic or steric protection is necessary. We and others have recently reported methodologies to enhance the accepting properties as well as the stability of boron by the introduction of *ortho* trifluoromethylaryl moieties.^[^ ^15^, ^16^, ^20^ ^]^ Increased stabilization can be partially attributed to a direct interaction between the electron pairs of the fluorine and the boron center, which is supported by short B–F distances in crystal structures.^[^ ^16k^ ^]^ In our experience, the trifluoromethyl groups also improve the volatility as well as the solubility of compounds, thereby improving their processability. The impact of trifluoromethyl substitution on carbazole for the development of deep blue TADF emitters has been demonstrated recently and investigated concerning their photostability. No direct correlation of the photostability with the employment of trifluoromethyl groups was observed.^[^ ^21^ ^]^

**Scheme 2:**
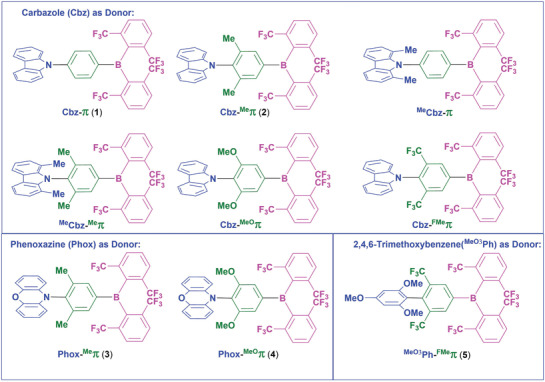
Calculated D‐π‐A systems with varying substituents on the donor (blue) and π‐bridge (green), while the acceptor (magenta) is the same in all compounds. Compounds **1**–**5** were synthesized and fully characterized and are numbered in the text (compounds without numbers are models used for computations).

Herein, we report on a quantum‐chemical exploration of structure–property relationships in a series of donor(D)‐π‐bridge‐acceptor(A) systems containing the B(^F^Xyl)_2_ (^F^Xyl = 2,6‐bis(trifluoromethyl)phenyl) acceptor group to design TADF emitters by selectively tuning the energy gap between the ^3^LE (^3^LE_D_: LE state confined on D, ^3^LE_π_: LE state confined on π‐bridge) and the CT states, via functionalization of the donor (D) and the π‐bridge (**Scheme** **2**).

To this end, we used *para*‐phenylene (π = 1,4‐C_6_H_4_) as the π‐bridge containing various substituents with varying electron‐donating or ‐accepting strength in our computations. Subsequently, as a proof of concept, we experimentally corroborated our theoretical predictions. We demonstrate how the occurrence of a ^3^LE_π_ state provides an effective handle by which the CT and LE states can be independently tuned, thus adjusting the energy gap between them.

More importantly, we outline a novel and rational design strategy to generate an array of TADF emitters with exceptionally small energy gaps, and emission energies which span nearly the complete spectrum of visible light.

## 2 Results and Discussion

### 2.1 In Silico Molecular Design

In order to meet the required accuracy for the energy prediction of the charge transfer state, we used an optimally tuned range‐separated functional (ZORA‐LC‐BLYP*/TZ2P/COSMO, see Supporting Information for detailed information) with a predetermined range separation parameter γ derived from the ionization potential of *N* and *N*+1 electron systems and benchmarked our protocol against available experimental data. A good linear relationship (correlation coefficient *R* ^2^ = 0.94, see Figure S3, Supporting Information) and an excellent mean absolute deviation of 0.05 eV are achieved. Overall, our protocol can successfully predict the gaps in an unknown molecule D‐π‐A except in cases where the assignment of the nature of the excited states as CT or LE becomes ill defined.^[^ ^22^ ^]^ All of the computed photophysical data for the compounds in Scheme 2 are listed in **Table** **1**. To lay the foundation, we experimentally and theoretically investigated the structural and photophysical properties of 9‐(4‐(bis(2,6‐bis(trifluoromethyl)phenyl)boryl)phenyl)‐9*H*‐carbazole, Cbz‐π (**1**, Scheme 2), which is composed of the moderately strong electron‐donor carbazolyl (Cbz) and an unsubstituted phenylene ring connecting the carbazole nitrogen with the B(^F^Xyl)_2_ acceptor. Photophysical measurements showed only prompt fluorescence (τ_PF_ = 10.0 ns in toluene) and revealed no delayed fluorescence or TADF behavior. The computed dihedral angles between the donor and the π‐bridge, and between the π‐bridge and the acceptor in the equilibrium ground state structure are 49° and 27°, respectively. From a single‐crystal X‐ray diffraction study, we found slightly smaller angles of 43.06(7)° and 17.83(10)°, respectively. Such small dihedral angles enable spatial delocalization of the frontier Kohn–Sham (KS) molecular orbitals, leading to a relatively large exchange energy *K* and thus a large Δ$E_{1_{\text{CT}}-3_{\text{CT}}}$ of 0.43 eV (Table 1). On the other hand, Δ$E_{1_{\text{CT}}-3_{\text{LE}}}$ is rather small (0.06 eV). However, Δ$E_{1_{\text{CT}}-3_{\text{CT}}}$ decreases to 0.13 eV upon gradually increasing the donor‐ and π–A dihedral angle to 90° (Figure S6, Supporting Information), highlighting the importance of decoupling the donor and the acceptor via increased steric hindrance.^[^ ^9^ ^]^

**Table 1:** Calculated photophysical data for all compounds in Scheme 2. The table shows the vertical excitation energies (in eV and nm) with its corresponding oscillator strength (*f*), charge transfer metrics (*Λ* and *R*_eh_ in Å), assignment of the excited states along with the MO composition (configuration interaction, CI) of the transition (in %) obtained from one‐electron Tamm–Dancoff approximation (TDA)‐DFT excitations, the computed S–T gaps, and the emission energies (Δ*E*(S_1_) and Δ*E*_VE_(T_1_)) in eV and nm. Fields in bold represent the mapping of the FC‐T*_n_* state corresponding to the relaxed T_1_ state according to the difference density plots

Cpd.	State	Energy		Assign.	*f*	CI (%)	*Λ* ^a)^	*R*_eh_ ^b)^ [Å]	Δ*E*_¹CT‐³LE_	Δ*E*_¹CT‐³CT_	Δ*E*_³CT‐³LE_
		[eV]	[nm]						[eV]	[eV]	[eV]
Cbz‐π (**1**)	FC‐S_1_	3.42	363	^1^CT	0.512	H→L (84), H‐3→L (4)	0.36	5.52	0.06	0.43	0.49
	**FC‐T_1_**	**2.99**	**415**	**^3^CT**	–	H→L (64), H‐3→L (19)	**0.42**	**4.82**			
	FC‐T_2_	3.48	356	^3^LE_D_	–	H‐1→L+4 (60), H→L+5 (32)	0.63	1.66			
	FC‐T_3_	3.54	350	^3^LE_D_	–	H‐1→L+4 (61), H→L+5 (24)	0.62	2.38			
	FC‐T_4_	3.82	325	^3^LE_π_	–	H‐4→L (64), H‐5→L+5 (7)	0.51	1.77			
	FC‐T_5_	3.89	319	^3^LE_A_	–	H‐6→L (48), H‐5→L+3 (15)	0.66	0.66			
	Δ*E*(S_1_)	3.19	389	_	–						
	Δ*E*_VE_(T_1_)	2.57	482	**FC‐T_1_**	–						
Cbz‐^Me^π (**2**)	FC‐S_1_	3.56	348	^1^CT	0.075	H→L (85), H→L+5 (7)	0.18	6.35	0.17	0.24	0.07
	**FC‐T_1_**	**3.32**	**373**	**^3^CT**	–	H‐5→L (47), H→L (33)	**0.36**	**5.01**			
	FC‐T_2_	3.39	366	^3^LE_π_	–	H‐2→L (83), H‐2→L+5 (11)	0.43	2.51			
	FC‐T_3_	3.44	360	^3^LE_D_	–	H→L+4 (81), H→L+5 (12)	0.62	0.82			
	FC‐T_4_	3.53	351	^3^LE_D_	–	H‐1→L+4 (77), H‐1→L+5 (10)	0.69	1.24			
	FC‐T_5_	3.68	337	^3^CT	–	H→L (51), H‐4→L (30)	0.30	5.52			
	Δ*E*(S_1_)	3.17	391	–	–						
	Δ*E*_VE_(T_1_)	2.65	468	**FC‐T_1_**	–						
Cbz‐^MeO^π	FC‐S_1_	3.44	360	^1^CT	0.175	H‐2→L (55), H→L (37)	0.35	4.19	0.58	0.31	0.27
	FC‐T_1_	2.86	434	^3^LE_π_	–	H‐2→L (77), H→L (7)	0.40	2.74			
	**FC‐T_2_**	**3.13**	**396**	**^3^CT**	–	H→L (39), H‐3→L (32)	**0.38**	**4.40**			
	FC‐T_3_	3.50	354	^3^LE_D_	–	H→L+5 (61), H→L+4 (30)	0.61	2.15			
	FC‐T_4_	3.55	349	^3^LE_D_	–	H‐1→L+5 (54), H‐1→L+4 (32)	0.59	2.83			
	FC‐T_5_	3.80	326	^1^CT	–	H→L (38), H‐3→L (36)	0.39	4.20			
	Δ*E*(S_1_)	2.91	426		–						
	Δ*E*_VE_(T_1_)	2.61	475	**FC‐T_2_**	–						
^Me^Cbz‐π	FC‐S_1_	3.27	379	^1^CT	0.122	H→L (85), H→L+4 (10)	0.19	6.42	0.17	0.14	0.31
	**FC‐T_1_**	**3.13**	**396**	**^3^CT**	–	H→L (68), H‐3→L (15)	**0.26**	**5.68**			
	FC‐T_2_	3.44	360	^3^LE_D_	–	H→L+5 (89)	0.68	0.07			
	FC‐T_3_	3.54	350	^3^LE_D_	–	H‐1→L+5 (74), H→L+7 (4)	0.72	1.02			
	FC‐T_4_	3.63	342	^3^LE_D_	–	H‐3→L (57), H→L (17)	0.50	3.38			
	FC‐T_5_	3.74	332	^3^LE_π_	–	H‐5→L (67), H‐1→L (13)	0.46	2.72			
	Δ*E*(S_1_)	2.74	452		–						
	Δ*E*_VE_(T_1_)	2.49	498	**FC‐T_1_**	–						
^Me^Cbz‐^Me^π	FC‐S_1_	3.23	384	^1^CT	0.012	H→L (87), H→L+4 (9)	0.12	6.54	0.13	0.03	0.16
	**FC‐T_1_**	**3.20**	**388**	**^3^CT**	–	H→L (78), H→L+4 (8)	**0.27**	**5.20**			
	FC‐T_2_	3.36	369	^3^LE_π_	–	H‐3→L (49), H‐2→L (36)	0.47	3.36			
	FC‐T_3_	3.36	369	^3^LE_D_	–	H‐4→L (71), H‐4→L+4 (11)	0.35	4.63			
	FC‐T_4_	3.40	365	^3^LE_D_	–	H→L+5 (92)	0.65	0.13			
	FC‐T_5_	3.54	350	^3^LE_D_	–	H‐1→L+5 (82)	0.76	0.28			
	Δ*E*(S_1_)	2.71	457		–						
	Δ*E*_VE_(T_1_)	2.57	482	**FC‐T_1_**	–						
Phox‐^Me^π (**3**)	FC‐S_1_	2.83	438	^1^CT	0.000	H→L (85), H→L+4 (10)	0.10	6.57	0.23	0.01	0.24
	**FC‐T_1_**	**2.82**	**440**	**^3^CT**	–	H→L (85), H→L+4 (10)	**0.10**	**6.57**			
	FC‐T_2_	3.06	405	^3^LE_D_	–	H→L+5 (89)	0.69	0.26			
	FC‐T_3_	3.40	365	^3^LE_π_	–	H‐2→L (83), H‐2→L+4 (13)	0.43	2.61			
	FC‐T_4_	3.42	363	^3^LE_π_	–	H‐3→L (78), H‐3→L+4 (14)	0.58	2.40			
	FC‐T_5_	3.49	355	^3^LE_D_	–	H→L+7 (92)	0.73	0.09			
	Δ*E*(S_1_)	2.15	577	–	–						
	Δ*E*_VE_(T_1_)	2.00	620	**FC‐T_1_**	–						
Phox‐^MeO^π**(4**)	FC‐S_1_	2.76	449	^1^CT	0.070	H→L (88), H→L+4 (8)	0.17	6.49	0.04	0.09	0.13
	**FC‐T_1_**	**2.67**	**464**	**^3^CT**	–	H→L (74), H‐1→L (10)	**0.21**	**5.91**			
	FC‐T_2_	2.80	443	^3^LE_π_	–	H‐1→L (76), H→L (9)	0.39	3.29			
	FC‐T_3_	3.10	400	^3^LE_D_	–	H→L+5 (92)	0.69	0.12			
	FC‐T_4_	3.37	368	^3^LE_π_	–	H‐3→L (70), H‐3→L+4 (12)	0.46	3.43			
	FC‐T_5_	3.52	352	^3^LE_D_	–	H→L+7 (64), H→L+6 (24)	0.68	0.46			
	Δ*E*(S_1_)	2.02	614	–	–						
	Δ*E*_VE_(T_1_)	1.75	709	FC‐T_1_	–						
CBz‐^FMe^π	FC‐S_1_	3.03	409	^1^CT	0.000	H→L (80), H→L+2 (11)	0.10	6.48	0.52	0.01	0.53
	**FC‐T_1_**	**3.02**	**411**	**^3^CT**	–	H→L (80), H→L+2 (12)	**0.11**	**6.48**			
	FC‐T_2_	3.54	350	^3^CT	–	H‐1→L (77), H‐1→L+6 (9)	0.14	6.12			
	FC‐T_3_	3.55	349	^3^LE_D_	–	H‐1→L+6 (79), H‐1→L (9)	0.70	0.83			
	FC‐T_4_	3.59	345	^3^LE_D_	–	H→L+6 (94)	0.68	0.33			
	FC‐T_5_	3.66	339	^3^LE_π_	–	H‐8→L (31), H‐6→L (26), H‐5→L (20)	0.68	0.12			
	Δ*E*(S_1_)	2.64	470		–						
	Δ*E*_VE_(T_1_)	2.35	528	**FC‐T_1_**	–						
^MeO3^Ph‐^FMe^π (**5**)	FC‐S_1_	3.51	353	^1^CT	0.002	H‐1→L (71), H‐1→L+4 (9)	0.12	6.81	0.02	0.03	0.05
	**FC‐T_1_**	**3.48**	**356**	**^3^CT**	–	H‐1→L (64), H‐1→L+4 (8)	**0.16**	**6.38**			
	FC‐T_2_	3.53	351	^3^LE_π_	–	H‐3→L (64), H‐3→L+4 (7)	0.57	2.49			
	FC‐T_3_	3.69	336	^3^CT	–	H→L (79), H→L+4 (7)	0.08	6.59			
	FC‐T_4_	3.84	323	^3^LE_A_	–	H‐4→L (51), H‐5→L+3 (11)	0.65	0.65			
	FC‐T_5_	3.85	322	^3^LE_A_	–	H‐5→L (48), H‐4→L+3 (8)	0.66	0.82			
	Δ*E*(S_1_)	2.91	426		–						
	Δ*E*_VE_(T_1_)	2.46	504	**FC‐T_1_**	–						

a) Degree of spatial overlap between occupied and virtual orbitals involved in the excitation: $\Lambda =\;\frac{\sum_{i,a}c_{i,a}^{2}〈\varphi_{a}||\varphi_{i}〉}{\sum_{i,a}c_{i,a}^{2}}$

b) Hole–electron distance: $R_{\text{eh}}=\;\frac{\sum_{i,a}c_{i,a}^{2}\left|〈\varphi_{a}|r|\varphi_{a}〉-〈\varphi_{i}|r|\varphi_{i}〉\right|}{\sum_{i,a}c_{i,a}^{2}}$.

**Figure 1:**
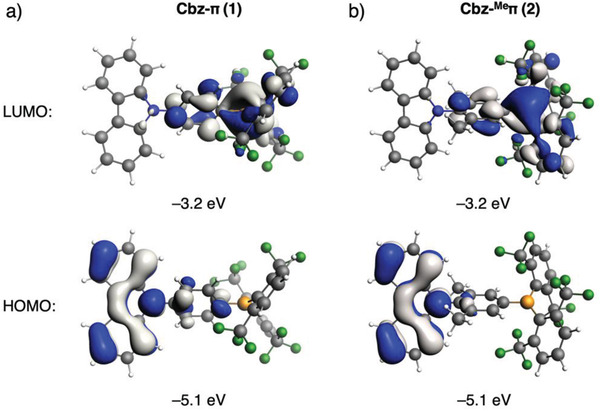
HOMO and LUMO of a) Cbz‐π (**1**); and b) Cbz‐^Me^π (**2**). Surface isovalue: ± 0.03 [*e a*_0_^−3^]^½^.

Of particular interest is the lowest localized triplet state (^3^LE_D_) which is at 3.48 eV and is spatially confined on the Cbz donor in Cbz‐π (**1**), while the localized triplet state on the bridge (^3^LE_π_) at 3.82 eV is rather high in energy and thus energetically forbidden to participate in the TADF process. We anticipated that inserting donor groups, such as methyl, at the 3‐ and 5‐position on the phenylene bridge would increase the dihedral angle between D and π–A and simultaneously red‐shift the ^3^LE_π_ energy such that the latter state becomes an integral part of the TADF mechanism. To achieve this, we modeled 9‐(4‐(bis(2,6‐bis(trifluoromethyl)phenyl)boryl)‐2,6‐dimethylphenyl)‐9*H*‐carbazole (Cbz‐^Me^π, **2**). Now, in contrast with the delocalized frontier KS molecular orbitals in Cbz‐π (**1**), the HOMO in Cbz‐^Me^π (**2**) is localized on the Cbz subunit, while the LUMO is localized more on the acceptor and is derived mostly from the empty p_z_ orbital on boron in B(^F^Xyl)_2_ (**Figure** **1**).

As predicted, the Δ$E_{1_{\text{CT}}-3_{\text{CT}}}\;$becomes smaller (0.24 eV) owing to a small exchange energy *K* arising from a large dihedral angle (88°) between the D and the π‐bridge. Indeed, experimentally, we found a dihedral angle of 76.08(6)° in the solid state for compound Cbz‐^Me^π (**2**). Moreover, the ^3^LE_π_ energy, which is essentially a π→π* transition on the phenylene bridge, is red‐shifted from 3.82 eV in Cbz‐π (**1**) to 3.39 eV in Cbz‐^Me^π (**2**). As a result, the ^3^LE_D_ is replaced by ^3^LE_π_ as the lowest LE state, which results in a small Δ$E_{1_{\text{CT}}-3_{\text{LE}}}$ of 0.17 eV. This is a direct outcome of a decrease in the HOMO–LUMO gap of the bridge itself (4.8 eV in ^Me^π‐H_2_ vs 5.1 eV in π‐H_2_, see Table S5, Supporting Information) due to the mild positive inductive effect of the –CH_3_ substituents. Note that, in contrast with the ^3^LE_D_ state, the ^3^LE_π_ can, in principle be tuned independently from the ^1^CT and ^3^CT states, i.e., by the modification of the bridge moiety.

Recently, Bickelhaupt et al. studied how the interplay of electronic properties of D, π, or A fragments affects the electronic property of the overall D‐π‐A molecule.^[^ ^23^ ^]^ Subsequently, to simplify the picture and to link the changes in the different excited states with changes in the difference between the occupied and virtual orbital energies participating in the corresponding excitation, we need to consider two relevant HOMO–LUMO gaps: the HOMO–LUMO gap corresponding to the donor moiety (D‐H) denoted as $\Delta E_{\text{H}-\text{L}}^{\text{D}}$ and the HOMO–LUMO gap corresponding to the π‐bridge denoted as $\Delta {\text{E}}_{\text{H–L}}^{\pi }$.

A smaller $\Delta {\text{E}}_{\text{H–L}}^{\pi }$ results in a red‐shifted ^3^LE_π_ or π→π* energy in ^Me^π‐H_2_, compared with that in π‐H_2_. Concomitantly, the energy gap $\Delta E_{3_{\text{CT}}-3_{\text{LE}}}$, which determines the extent of resonance between the triplet states, is smaller in Cbz‐^Me^π (**2**, 0.07 eV in toluene) compared with that in Cbz‐π (**1**, 0.37 eV). Thus, based on this simplified discussion, the former should have a much higher probability of showing TADF behavior than the latter. Indeed, we found experimentally that Cbz‐^Me^π (**2**) shows a delayed fluorescence lifetime of 13.6 µs and an experimentally observed singlet–triplet gap in methylcyclohexane of 0.13 eV, in excellent agreement with the calculated Δ$E_{1_{\text{CT}}-3_{\text{LE}}}$ gap of 0.17 eV.

Further modeling of the bridge by replacing both methyl groups with methoxy substituents at the 2,6‐positions of the phenylene bridge (Scheme 2) to generate 9‐(4‐(bis(2,6‐bis(trifluoromethyl)‐phenyl)boryl)‐2,6‐dimethoxyphenyl)‐9*H*‐carbazole (Cbz‐^MeO^π) leads to a further red‐shifted ^3^LE_π_ state (3.39 eV in Cbz‐^Me^π to 2.86 eV in Cbz‐^MeO^π) as expected by the higher lying HOMO of the bridge fragment in Cbz‐^MeO^π compared to Cbz‐^Me^π (**2**).

However, the introduction of two methoxy groups pushes the ^3^LE_π_ state below the ^3^CT. Such a large change in ^3^LE_π_ energy results in a large Δ$E_{1_{\text{CT}}-3_{\text{LE}}}$ of 0.58 eV, rendering the rISC process inefficient. So, the combination of D and π‐bridge in Cbz‐^Me^π (**2**) and Cbz‐^MeO^π serves as an example of a match and a mismatch, respectively, of the TADF energy gaps. Interestingly, the CT energies are hardly affected for both Cbz‐^Me^π and Cbz‐^MeO^π (see Table 1).

Another interesting example is the case of 10‐(4‐(bis(2,6‐bis(trifluoromethyl)phenyl)‐boryl)‐2,6‐dimethylphenyl)‐10H‐phenoxazine (Phox‐^Me^π, **3**, Scheme 2), in which the donor is transformed to phenoxazine (Phox) and the π‐bridge is 2,6‐dimethylphenylene. This has multiple effects on the excited states. First, the large steric hindrance on the donor and π–A molecular planes [calculated: 89°, experimentally found in the solid state: 82.05(7)°] induced by hydrogens at the 1,9 positions of Phox and the methyl groups on the ^Me^π, results in a large spatial separation of the HOMO at the donor subunit and the LUMO at the acceptor subunit. This leads to an expected extremely small Δ$E_{1_{\text{CT}}-3_{\text{CT}}}$ gap of 0.01 eV in Phox‐^Me^π (**3**). Second, the introduction of Phox results in the decrease of the CT (both the lowest ^1^CT and ^3^CT) energies from ≈3.50 eV in Cbz‐^Me^π (**2**) to ≈2.80 eV in Phox‐^Me^π (**3**). Phox is a stronger electron donor than Cbz (HOMO = −4.3 and −5.0 eV for Phox‐H and Cbz‐H, respectively ‘see Table S5', Supporting Information). Lastly, the lowest ^3^LE state is again switched from the bridge (^3^LE_π_) in Cbz‐^Me^π (**2**) to the donor moiety (^3^LE_D_) in Phox‐^Me^π (**3**). This is due to a significantly stabilized ^3^LE_D_ in Phox‐^Me^π (**3**: 3.06 eV) compared to that in Cbz‐^Me^π (**2**: 3.44 eV), while the ^3^LE_π_ state is essentially unaffected. Again, this is directly related to the stronger electron‐donating ability of Phox compared to Cbz, which leads to a higher HOMO energy and a smaller $\Delta {\text{E}}_{\text{H–L}}^{\text{D}}$ of 2.9 eV in Phox‐H compared to 3.3 eV in Cbz‐H.

Therefore, despite a small Δ$E_{1_{\text{CT}}-3_{\text{CT}}}$ of 0.01 eV, the Δ$E_{1_{\text{CT}}-3_{\text{LE}}}$ is relatively large (0.23 eV) due to a strongly stabilized ^1^CT state. However, experimentally it was found that compound Phox‐^Me^π (**3**) shows TADF with a delayed fluorescence lifetime of 0.7 µs and an experimentally observed singlet–triplet gap of 0.04 eV in toluene, which compares nicely with the calculated Δ$E_{1_{\text{CT}}-3_{\text{CT}}}$ of 0.01 eV.

To decrease the Δ$E_{1_{\text{CT}}-3_{\text{LE}}}$ gap in Phox‐^Me^π further, we need to stabilize the ^3^LE as well and, therefore, we replaced the methyl with methoxy groups on the π‐bridge. As expected, 10‐(4‐(bis(2,6‐bis(trifluoromethyl)phenyl)boryl)‐2,6‐dimethoxyphenyl)‐10*H*‐phenoxazine (Phox‐^MeO^π, **4**) displays a significant stabilization of the ^3^LE_π_ state, compared with that of Phox‐^Me^π (2.80 eV vs 3.40 eV). This enhanced stabilization proceeds with ^3^LE_D_ being replaced by ^3^LE_π_ as the lowest ^3^LE state. Consequently, the Δ$E_{1_{\text{CT}}-3_{\text{LE}}}$ drops to a mere 0.04 eV and Phox‐^MeO^π (**4**) exhibits ideal energy gaps for an efficient rISC process. The experimentally obtained singlet–triplet gap of 0.03 eV is in reasonable agreement with the corresponding calculated gap (Δ$E_{1_{\text{CT}}-3_{\text{CT}}}$) of 0.09 eV (vide infra).

**Scheme 3:**
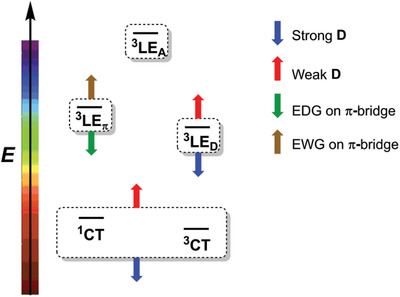
Schematic illustration of the independent tuning of the local excited state on the π‐bridge (^3^LE_π_); the local excited state on the donor D (^3^LE_D_) and the charge transfer from donor D to acceptor A in a D‐π‐A molecule. The local excited state (^3^LE_A_) on the acceptor is almost independent of the variation of the donor and bridge moieties. Tuning of the emission color is controlled by the energy of ^1^CT.

Overall, we can summarize our findings thus far as follows: the nature and the energy of the lowest ^3^LE state involve an intricate interplay between the strength of the electron‐donating substituent on the π‐bridge and the strength of the electron donor (D), whereas, the energy of the CT state is a function of the strength of the donor (D) and the acceptor (A). It can be deduced that a combination of a strong donor (D) and a strong electron‐donating substituent on the π‐bridge or a weaker donor (D) and a weaker electron‐donating substituent, i.e., an electron‐withdrawing group, on the π‐bridge is required to achieve the desired TADF features (**Scheme** **3**).

To generate more appropriate combinations, we computed the frontier molecular orbital energies (Table S5, Supporting Information) and the HOMO–LUMO gaps of the various D‐H and π‐H_2_ and used the data as a guideline for further calculations. Again, considering Cbz‐π (**1**) as the reference system, adding two methyl groups at the 1,8‐positions (Scheme 2) to the Cbz donor to form ^Me^Cbz raises the HOMO by 0.1 eV (Table S5, Supporting Information). As a result, the CT energies (both the lowest ^1^CT and ^3^CT) corresponding to both 9‐(4‐(bis(2,6‐bis(trifluoromethyl)phenyl)boryl)phenyl)‐1,8‐dimethyl‐9*H*‐carbazole (^Me^Cbz‐π) and 9‐(4‐(bis(2,6‐bis(trifluoromethyl)phenyl)boryl)‐2,6‐dimethylphenyl)‐1,8‐dimethyl‐9*H*‐carbazole (^Me^Cbz‐^Me^π) are slightly decreased, compared to those in the unsubstituted Cbz‐π (**1**). In contrast, the lowest LE state changes its nature from being confined on ^Me^Cbz in the former (^3^LE_D_) to ^Me^π in the latter (^3^LE_π_). As a result, the high‐lying ^3^LE_π_ at 3.74 eV in ^Me^Cbz‐π drops to 3.36 eV in ^Me^Cbz‐^Me^π leading to a small Δ$E_{1_{\text{CT}}-3_{\text{LE}}}$ of 0.13 eV. Therefore, both donors Cbz (explained above) and ^Me^Cbz match with the bridge ^Me^π to exhibit small TADF energy gaps.

Finally, we analyzed the effect of adding two electron‐withdrawing trifluoromethyl groups (–CF_3_) to the phenyl bridge to generate ^FMe^π (Scheme 2). To find a match with ^FMe^π as the bridge, we probed two different types of donors: i) a moderately strong electron‐donor Cbz to generate 9‐(4‐(bis(2,6‐bis(trifluoromethyl)phenyl)boryl)‐2,6‐bis(trifluoromethyl)phenyl)‐9*H*‐carbazole (Cbz‐^FMe^π) and ii) a moderately weak electron‐donor such as 2,4,6‐trimethoxybenzene (${}^{{\text{MeO}}_{3}}\text{Ph}$ in Scheme 2) to generate 4′‐(bis(2,6‐bis(trifluoromethyl)phenyl)boryl)‐2′,6′‐bis(trifluoromethyl)‐[1,1′‐biphenyl]‐2,4,6‐trimethoxy‐benzene (${}^{{\text{MeO}}_{\text{3}}}\text{Ph}$‐^FMe^π, **5**).

The bulky –CF_3_ group forces the D and π–A molecular planes to become nearly perpendicular (≈89°) in all cases, thereby decoupling the donor and the acceptor. As a result, Δ$E_{1_{\text{CT}}-3_{\text{CT}}}$ is, in general, quite small. The ^3^LE_π_ state, which is localized on ^FMe^π for both cases, is stabilized compared to the localized and higher lying ^3^LE_π_ state in phenylene (3.80 eV in phenylene‐bridged vs ≈3.53 and 3.66 eV in ${}^{{\text{MeO}}_{\text{3}}}\text{Ph}$‐^FMe^π (**5**) and Cbz‐^FMe^π, respectively). This is due to a large negative inductive effect of –CF_3_ which strongly stabilizes the LUMO by 1.2 eV in ^FMe^π, compared to phenylene. Still, such a high‐lying ^3^LE_π_ state would only match with a high‐lying CT state emerging from a weak D. As predicted, in the case of the weak donor ^MeO₃^Ph, the Δ$E_{1_{\text{CT}}-3_{\text{LE}}}$ and Δ$E_{3_{\text{CT}}-3_{\text{LE}}}$ reduce to ≈0.05 and ≈0.06 eV, respectively, while in the case of the moderately strong donor Cbz, the Δ$E_{1_{\text{CT}}-3_{\text{LE}}}$ and Δ$E_{3_{\text{CT}}-3_{\text{LE}}}$ gaps are ten times larger (≈0.5 eV). In the latter case, the CT states are much more stabilized than the ^3^LE_π_, leading to a large Δ$E_{1_{\text{CT}}-3_{\text{LE}}}$. Clearly, ^MeO₃^Ph, has a lower energy HOMO (by 0.3 eV compared to Cbz). Thus, the larger energy gap between HOMO and LUMO increases the CT energies of the corresponding D‐π‐A compounds. As a result, ${}^{{\text{MeO}}_{\text{3}}}\text{Ph}$‐^FMe^π (**5**) displays a remarkably small Δ$E_{1_{\text{CT}}-3_{\text{LE}}}$ and, concomitantly, a small Δ$E_{3_{\text{CT}}-3_{\text{LE}}}$ as well.

**Scheme 4:**
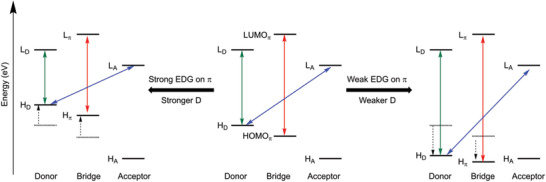
Schematic illustration of the modulation of the HOMO–LUMO energy gap for the lowest CT states (blue), local‐excited states at the donor (green) and at the bridge (red) via a combination of a strong donor–strong electron‐donating group (EDG) on the bridge, or a weak donor–weak electron‐donating group (i.e., an electron‐withdrawing group) on the bridge. Here, H_D_(L_D_), H_π_(L_π_), and H_A_(L_A_) denote the HOMO(LUMO) of donor, bridge, and acceptor, respectively.

Therefore, a combination of a weak donor with electron‐withdrawing –CF_3_ substituents on the bridge in ${}^{{\text{MeO}}_{\text{3}}}\text{Ph}$‐^FMe^π (**5**), a moderate donor with a moderate electron‐donating substituent on the bridge in Cbz‐^Me^π (**2**), and a strong donor with a strong electron‐donating substituent on the bridge in Phox‐^MeO^π (**4**) results in favorable TADF energy gaps; **Scheme** **4** summarizes this conclusion.

Additionally, the calculated delayed fluorescence energies in these three model systems range from deep blue (calc.: 391 nm, exp.: 460 nm) for Cbz‐^Me^π (**2**), to blue (calc.: 426 nm, exp.: 483 nm) for ${}^{{\text{MeO}}_{\text{3}}}\text{Ph}$‐^FMe^π (**5**) and red (calc.: 614 nm, exp.: 689 nm) for Phox‐^MeO^π (**4**) (see Δ*E*(S_1_) in Table 1 for calculated and λ_em_ in **Table** **2** for the experimentally observed values, respectively).

**Table 2:** Photophysical data for compounds **1**–**5**. Calculated gaps are shown in curly brackets

	Solvent	λ_abs_ [nm]	ε [10^3^ M^−1^ cm^−1^]	λ_em_ [nm]	Stokes shift [cm^−1^]	τ_PF_ (%) [ns]	τ_DF_ (%) [µs]	*Φ*_PL_	Δ*E*_ST_ [eV]
**1**	Hexane	400	17.7	423	1359	7.6 (100)	–	0.97	–
	Toluene	398		470	3976	10.0 (100)	–	0.98	
	THF	387		524	6756	14.8 (100)	–	0.89	
**2**	Hexane	384	3.8	412	1838	5.3 (97)	1.7 (3)	0.16	0.13^a)^ {0.17}
	Toluene	380		460	4576	18.0 (62)	13.6 (38)	0.18	
	THF	370		515	7610	34.0 (21)	2.9 (79)	0.61	
	77 K^b)^	400		414^c)^	1019	10.0^c)^	–^c)^	–	
	PMMA^d)^	387		448	3518	10.3 (63)	8.4 (37)	0.55	
**3**	Hexane	465	1.2	536	2849	40.0 (20)	1.4 (80)	0.79	0.04^e)^ {0.01}
	Toluene	463		623	5547	38.1 (35)	0.7 (65)	0.26	
	THF	450		745	8799	–^f)^	–^f)^	–^f)^	
	77 K^b)^	480		540	2315	127.3 (30)	3.3 (70)^g)^	–	
	PMMA^d)^	443		568	4968	57.4 (41)	1.3 (59)	0.65	
**4**	Hexane	506	1.8	600	3096	40.1 (43)	1.4 (57)	0.54	0.03^e)^ {0.09}
	Toluene	508		689	5171	9.1 (90)	0.6 (10)	0.02	
	THF	480		778	7980	–^f)^	–^f)^	–^f)^	
	77 K^b)^	468		553	3284	55.4 (84)	2.6 (16)	–	
	PMMA^d)^	464		598	4829	37.0 (74)	1.6 (26)	0.40	
**5**	Hexane	354	1.1	439	5470	13.8 (99)	0.4 (1)	0.13	0.03^h)^ {0.03}
	Toluene	354		483	7545	34.7 (79)	29.3 (21)	0.42	
	THF	352		559	10520	22.5 (82)	1.2 (18)	0.12	
	77 K^i)^	361		469	6379	80.3 (59)	3.4 (41)	–	
	PMMA^d)^	355		477	7205	29.4 (58)	5.5 (42)	0.85	

a) Measured in 2‐MeTHF and obtained from the onset of the fluorescence and phosphorescence spectra

b) Measured in a methylcyclohexane glass matrix

c) Phosphorescence with vibrational bands at 445 and 470 nm and τ_Phos,av_ = 1.0 s was observed

d) Measured in a 1 wt% PMMA film

e) Measured in toluene and obtained from an Arrhenius plot

f) Too weak to measure

g) Delayed component contains more than one lifetime, averaged lifetime (τ_DF,av_) is used

h) Measured in 2‐MeTHF and obtained from an Arrhenius plot

i) Measured in a 2‐MeTHF glass matrix.

After having characterized the excited state behavior of the model compounds in silico and having identified the potential TADF emitters, compounds **1**–**5** were synthesized, and all intermediates and resulting products were characterized by elemental analysis, single crystal X‐ray diffraction, ^1^H, ^13^C{^1^H}, ^11^B, and ^19^F NMR spectroscopy, cyclic voltammetry, and High resolution mass spectrometry (HRMS). The photophysical properties of compounds **1**–**5** in various solvents, polymeric films, and in a frozen matrix were investigated in detail and the results are compared with the computationally obtained data.

### 2.2 Synthesis

**Scheme 5:**
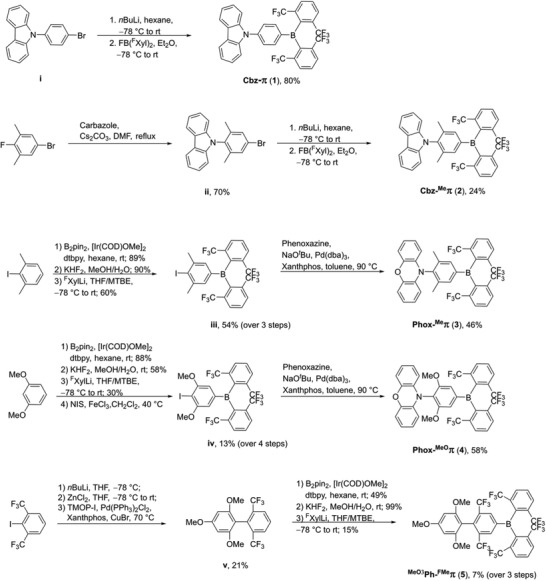
Synthesis of compounds **1**–**5**.

The carbazole derivatives Cbz‐π (**1**) and Cbz‐^Me^π (**2**) were synthesized starting from the donor‐bridge moiety (**Scheme** **5**). The starting material **i** was synthesized according to the literature^[^ ^24^ ^]^ via an Ullman type amination. The methylated derivative **ii** was synthesized by nucleophilic *ipso*‐substitution of fluorine in 5‐bromo‐2‐fluoro‐1,3‐dimethylbenzene with carbazole. Both compounds were subsequently lithiated using *n*BuLi and reacted with bis(2,6‐bis(trifluoromethyl)phenyl)fluoroborane to form **1** and **2**, respectively.

When attempting to synthesize Phox‐^Me^π (**3**) analogously to **2**, we encountered problems with the synthesis of the donor‐bridge moiety. To circumvent this, an alternative route was employed. To introduce the boron center, 2‐iodo‐1,3‐dimethylbenzene was borylated using an iridium‐catalyzed C—H borylation.^[^ ^25^ ^]^ This C—H borylation exhibits a high meta selectivity due to steric factors.^[^ ^26^ ^]^ The boronate ester then fluorinated using KHF_2_ to form the corresponding potassium aryltrifluoroborate salt. Aryltrifluoroborate salts are widely used as nucleophiles in Suzuki–Miyaura‐type cross‐coupling reactions and are desirable intermediates due to their high stability.^[^ ^27^ ^]^ We and others have previously reported their application as convenient precursors to triarylboranes.^[^ ^16^, ^20^, ^28^ ^]^ The potassium aryltrifluoroborate salt was reacted with three equivalents of (2,6‐bis(trifluoromethyl)‐phenyl)lithium (^F^XylLi), prepared by the reaction of 2‐iodo‐1,3‐bis(trifluoromethyl)benzene with *n*BuLi, to form the triarylborane **iii** in 54% yield over three steps. Compared to the synthesis of **ii**, this approach is advantageous as it does not require expensive 5‐bromo‐2‐fluoro‐1,3‐dimethylbenzene as the bridging moiety. In the last step, the phenoxazine donor moiety was introduced via a palladium‐catalyzed Buchwald–Hartwig amination using NaO*^t^*Bu as the base and Xantphos as the ligand. Compound Phox‐^MeO^π (**4**) was synthesized using the same basic approach. First, 1,3‐dimethoxybenzene was regioselectively borylated, then fluorinated and reacted with ^F^XylLi to form the corresponding triarylborane. In order to introduce iodine, the triarylborane was reacted with NIS using FeCl_3_ as a Lewis acid to give **iv** in 13% yield over four steps. In this case, the iodination can be carried out at the triarylborane due to the electron‐rich and *ortho‐*directing nature of the methoxy substituents. The donor moiety was introduced analogously to **3** in a palladium‐catalyzed Buchwald–Hartwig amination. In the synthesis of ${}^{{\text{MeO}}_{\text{3}}}\text{Ph}$‐^FMe^π (**5**), the most problematic step is the coupling of 1,3,5‐trimethoxybenzene with ^F^XylLi. For this reason, it was chosen as the first step. In a one‐pot reaction, ^F^XylLi was prepared and converted to (^F^Xyl)_2_ Zn which was then reacted with 2,4,6‐trimethoxyiodobenzene in a palladium‐catalyzed Negishi coupling reaction using Xantphos as the ligand with CuBr as an additive. Both the conversion to the organozinc compound and the copper salt are essential in this reaction. Then, **v** was borylated, fluorinated, and reacted with ^F^XylLi to give **5** in 7% yield over three steps.

Compounds **1**–**5** were investigated by ^1^H, ^11^B, ^19^F, and ^13^C {^1^H} NMR spectroscopy. For all compounds, a characteristic triplet and doublet corresponding to the ^F^Xyl backbone was observed in the ^1^H NMR spectra. The signals in the ^19^F NMR spectra corresponding to the *ortho*‐CF_3_ groups are broadened due to hindered rotation of the ^F^Xyl moieties. This has been previously observed and studied for other *ortho*‐CF_3_‐substituted triarylboranes.^[^ ^16^, ^20^ ^]^

### 2.3 Solid‐State Structures

**Figure 2:**
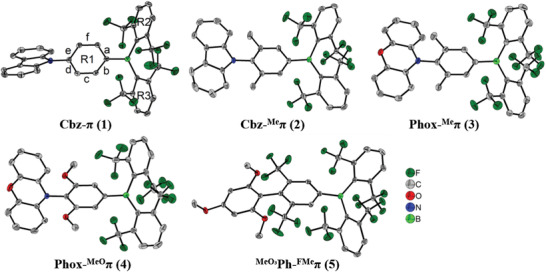
The solid‐state molecular structures of **1**–**5** determined by single‐crystal X‐ray diffraction at 100 K. All ellipsoids are drawn at the 50% probability level, and H atoms are omitted for clarity.

Solid‐state structures of compounds **1**–**5** determined by single‐crystal X‐ray diffraction are shown in **Figure** **2** and selected bond lengths and angles are listed in Table S7 (Supporting Information) A detailed discussion of all structural parameters can be found in the Supporting Information.

The phenylene bridge between the donor and acceptor moieties of compound **1** exhibits a quinoidal distortion, as the c and f bonds (Figure 2) are more than 0.008–0.026 Å shorter than the a, b, d, and e bonds (Table S7, Supporting Information). This indicates a strongly polarized ground state of **1**. In the parent compound, 9‐phenyl‐9*H*‐carbazole, no quinoidal distortion is present and C—N bond lengths are 1.420(5) and 1.427(4) Å, respectively, for two symmetrically nonequivalent molecules.^[^ ^29^ ^]^ This is only slightly longer, within 1–2 standard deviations, if compared to **1** (C—N = 1.417(2) Å). Compared to **1**, the C–N bond to R1 is elongated in compound **2** (C—N = 1.430(2) Å, ΔC—N = 0.013 Å) which is likely due to the steric repulsion between the CH_3_ groups on R1 and the carbazole donor moiety. However, in compound **3** no steric effect of the CH_3_ groups on the C—N bond length is observed as the C—N bond of **3** (1.436(2) Å) is the same length as that of the parent compound 10‐phenyl‐10*H*‐phenoxazine (1.435(3) Å) within one estimated standard deviation (esd).^[^ ^30^ ^]^ Compounds **2**–**5** do not show a quinoidal distortion of the phenylene bridges within three esd's of the C—C bond lengths. This is attributed to the substitution with either CH_3_, MeO, or CF_3_ groups *ortho* to the donor moiety associated with an increase of the torsion angle between the donor moiety (NC_3_ in **1**–**4**/CC_3_ in **5**) and R1. These torsion angles are 43.06(7)° (**1**), 76.08(6)° (**2**), 82.05(7)° (**3**), 89.55(8)° (**4**), and 87.37(12)° (**5**), respectively (Table S7, Supporting Information). Groups on the *ortho* positions were specifically introduced in order to increase the torsion angles and, hence, limit the orbital overlap between donor and acceptor, which is crucial for efficient TADF. The smallest torsion angle is observed for compound **1** with the unsubstituted phenylene bridge. Due to steric repulsion between the *ortho*‐substituted CH_3_ groups and the carbazole donor moiety, the torsion angle is significantly increased in compound **2**, however, to a slightly smaller extent than compared to the phenoxazine derivatives **3**, **4** and the biphenyl derivative **5**. This is attributed to the more extended sizes of the donor moieties of **3**, **4**, and **5** as well as the more extended MeO and CF_3_ groups compared to CH_3_. Similarly to previously reported *ortho* CF_3_‐substituted triarylboranes,^[^ ^16^, ^20^ ^]^ compounds **1**–**5** exhibit four short B—F contacts (2.516(2)–2.926(3) Å, Table S7, Supporting Information) per B(^F^Xyl)_2_ moiety, which are shorter than the sum of their van der Waals radii (3.39 Å).^[^ ^31^ ^]^ This indicates a stabilizing effect of the *ortho* CF_3_ groups on the triarylborane.

### 2.4 Electrochemistry

Cyclic voltammograms of the D‐π‐A compounds **1**–**5** were recorded in order to determine their electronic properties (Figure S79 and Table S12, Supporting Information). All compounds exhibit reversible reduction events corresponding to the B(^F^Xyl)_2_ acceptor moiety. The reduction potentials of compounds **1**–**4** are very similar (*E*_1/2_ ≈ −2 V vs Fc/Fc^+^). The xylyl bridged species **2** and **3** are shifted to slightly more negative potentials than **1** and **4**. The reduction potential of **5**, however, is anodically shifted by about 150 mV. This is due to the CF_3_ groups on the bridge that are positioned *meta* to the boron center and thus have a larger electron withdrawing effect than the ones in the *ortho* positions at the terminal ^F^Xyl moieties.^[^ ^20m^ ^]^ In our previous study on trifluoromethylarylboranes, we observed that exchanging mesityl for fluoromesityl leads to a cathodic shift of about 1 V (Ph_2_NPhB(Mes)_2_: *E*_1/2, red_ = −2.60 V vs Fc/Fc^+^; Ph_2_NPhB(^F^Mes)_2_: *E*_1/2, red_ = −1.66 V vs Fc/Fc^+^).^[^ ^20h^ ^]^ Compounds **1**–**4** are anodically shifted compared to the B(^F^Mes)_2_ compound (Δ*E* ≈ 0.4 V) and cathodically shifted compared to the BMes_2_ compound (Δ*E* ≈ 0.6 V). This also illustrates the stronger electron withdrawing effect of the CF_3_ group in the *para* position as compared to the ones in the *ortho* positions. Only the phenoxazine derivatives **3** and **4** exhibit reversible oxidation events corresponding to the donor moiety (*E*_1/2_ ≈ 0.3 V vs Fc/Fc^+^). Compound **4** is about 50 mV cathodically shifted compared to **3**, which might be due to the electron‐donating nature of the OMe groups on the bridge. The carbazole derivatives **1** and **2** exhibit irreversible oxidation events characteristic of the carbazole moiety (*E*_pa_ ≈ 1 V vs Fc/Fc^+^). The oxidation potential of compound **5** is very close to the limit of the solvent window (*E*_pa_ = 1.21 V vs Fc/Fc^+^). In comparison, the oxidation potential of 1,3,5‐trimethoxybenzene is *E*_1/2, ox_ = 1.5 V.^[^ ^32^ ^]^

### 2.5 Photophysical Studies

First, we studied the photophysical properties of Cbz‐**π** (**1**) in various solvents (Table 2 and Supporting Information). While the absorption is hardly affected by the polarity of the solvent, the emission maxima in hexane, toluene, and THF are gradually red‐shifted from 423 to 470 and 524 nm, respectively. This agrees well with the calculated CT character of the lowest singlet excited state (Table 1). In agreement with the large calculated Δ$E_{3_{\text{CT}}-3_{\text{LE}}}$ and Δ$E_{1_{\text{CT}}-3_{\text{CT}}}$ gaps of 0.49 and 0.43 eV, respectively, we found only prompt fluorescence (PF) for compound **1** with measured lifetimes between 7.6 and 14.8 ns. The quantum yields (QYs) are near unity in hexane and toluene, indicating that the fluorescence is an efficient process. This demonstrates that bypassing of the intersystem crossing pathway leads to very effective organic fluorescent emitters.

**Figure 3:**
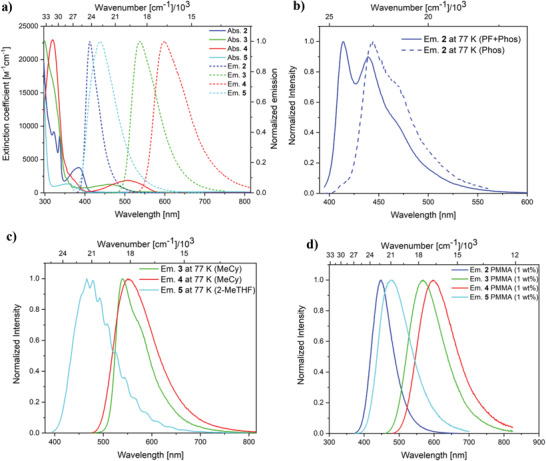
a) Absorption (solid line) and emission (dashed line) spectra of compounds **2**–**5** in degassed hexane. b) Overall emission spectrum (blue solid line) and time‐gated spectrum (blue dashed line, > 5 ms) of **2** in a glassy matrix (MeCy methylcyclohexane) at 77 K (PF = prompt fluorescence, Phos = phosphorescence). c) Emission spectra at 77 K of compounds **3**–**5** in a glassy matrix (see Table 2). All concentrations are < 2 × 10^−5^ mol L^−1^. d) Emission spectra of compounds **2**–**5** in 1 wt% PMMA films.

However, the introduction of two methyl groups at the 2,6‐positions of the phenylene bridge to form Cbz‐^Me^π (**2**) increases the dihedral angle between the carbazole and the bridge to 76.08(6)° in **2**, when compared to compound **1** (43.06(7)°). Therefore, we observe a decrease in the spatial overlap between the donor and acceptor orbitals vide supra; *Λ* drops from 0.36 for the lowest singlet transition in **1** to 0.18 in **2**, indicating a stronger CT character for **2**. At the same time, the experimentally observed extinction coefficient in hexane is four times smaller for **2** (3.8 × 10^3^ m ^−1^ cm^−1^ vs 17.7 × 10^3^ m ^−1^ cm^−1^). Interestingly, while the QY in hexane is only 16% and in toluene 18%, in the most polar solvent (THF), the QY is notably higher (61%). Both the absorption and the emission spectra are slightly blue‐shifted in all solvents (in hexane: 412 nm; **Figure** **3**a) and a stronger solvatochromic effect is observed, when comparing the spectra and Stokes' shifts of compound **2** with **1**. As computationally predicted, Cbz‐^Me^π (**2**) displays both, a prompt and delayed component in the decay of the emission signal at room temperature. The lifetimes measured in toluene are 18.0 ns for the prompt fluorescence and 13.6 µs for the delayed component, with relative percentages of 62% and 38%, respectively. The delayed fluorescence lifetime is several orders of magnitude shorter than one would expect for a pure phosphorescent emission of an organic molecule without any heavy atom (ms to s).^[^ ^33^ ^]^ Interestingly, the highest relative percentage for the delayed fluorescence component to the emission decay is found in the most polar solvent (THF: 79%), in which the quantum yield is also the highest. We observe that the relative percentages of the prompt and the delayed component are strongly dependent on the polarity of the solvent. At 77 K, embedded in a glassy matrix (methylcyclohexane, MeCy), compound **2** shows a structured emission with bands at 414, 445, and 470 nm (Figure 3c, blue line). The latter two bands correspond to a phosphorescent emission with an averaged lifetime of τ_Phos,av_ = 1.0 s, while the high energy band at 414 nm shows a much shorter lifetime of τ_PF_ = 10.0 ns. As these lifetimes are many orders of magnitude different, we were able to measure a time‐gated emission spectrum, recording the emission signal after a delay of 5 ms. The result is shown in Figure 3b (blue dashed line) and is the emission spectrum of the pure phosphorescent (Phos) component of the total emission, which is a mixture of the prompt fluorescence and the phosphorescence (PF+Phos). From these two spectra, we were able to extract Δ$E_{1_{\text{CT}}-3_{\text{LE}}}$ (from the onset of prompt fluorescence and phosphorescence spectra), which has a value of 0.13 eV and is in good agreement with the calculated gap (0.17 eV). In a 1 wt% polymeric film (PMMA = poly(methylmethacrylate)), compound **2** exhibits a structureless emission with a maximum at 448 nm (Figure 3d), and prompt and delayed lifetimes of τ_PF_ = 10.3 ns (63%) and τ_DF_ = 8.4 µs (37%), respectively. The photoluminescence quantum yield of 55% indicates that the emission process is moderately efficient in a polymeric film.

Both compounds bearing a phenoxazine donor (Phox‐^Me^π (**3**) and Phox‐^MeO^π (**4**)) show a further red‐shift of the emission spectra: **3** and **4** exhibit maxima of 623 and 689 nm in toluene, respectively. As the emission maxima are strongly solvent dependent, and these compounds display large Stokes' shifts and small extinction coefficients (1.2 × 10^3^ and 1.8 × 10^3^ M^−1^ cm^−1^), the corresponding transitions have strong charge‐transfer character. Both compounds show a prompt and a delayed component of the emission decay, but their relative contributions are again strongly solvent dependent. In toluene, the lifetimes are 38.1 ns (35%) and 0.7 µs (65%) for **3**, while for **4** the lifetimes are 9.1 ns (90%) and 0.6 µs (10%). Thus, their delayed components are much shorter than that of **2**. The quantum yield of **3** in toluene is higher (26%) than that of **2** (18%). However, for compound **4**, we found the opposite behavior, as the QY is much lower (2%) in toluene. In contrast to compound **2**, we found no sign of phosphorescence at 77 K. Instead, we observed a relative long‐lived prompt fluorescence (τ_PF_ = 127.3 ns (**3**) and τ_PF_ = 55.4 ns (**4**)) and a second component of the decay, with values of τ_DF_ = 3.3 µs (**3**) and τ_DF_ = 2.6 µs (**4**), which we assign to delayed fluorescence. In addition, we measured and plotted the relative components of the decays over a temperature range of 250–300 K and extracted from these Arrhenius plots the experimental values for Δ$E_{1_{\text{CT}}-3_{\text{CT}}}$ (Figures S76–S78 in the Supporting Information). Both compounds have gaps of only 0.04 and 0.03 eV, for **3** and **4**, respectively, which agrees reasonably well with the calculated gaps (0.01 eV for **3** and 0.09 eV for **4**). Such small gaps also explain why we did not observe phosphorescence, because, even at 77 K, the rISC process still takes place. The TADF process in a PMMA film is efficient, with high quantum yields for **3** (65%) to moderate for **4** (40%), and emission maxima at 568 and 598 nm for **3** and **4**, respectively (Figure 3d). The lifetimes measured are τ_PF_ = 57.4 ns (41%) and τ_DF_ = 1.3 µs (59%) for **3** and τ_PF_ = 37.0 ns (74%) and τ_DF_ = 1.6 µs (26%) for **4**.

The combination of a weak donor (${}^{{\text{MeO}}_{3}}\text{Ph}$) with an electron‐withdrawing group‐containing bridge (^FMe^Ph) in compound ${}^{{\text{MeO}}_{\text{3}}}\text{Ph}$‐^FMe^π (**5**) gives rise to an emission maximum in toluene of 483 nm and lifetimes of τ_PF_ = 34.7 ns (79%) and τ_DF_ = 29.3 µs (21%). Interestingly, compound **5** has the highest quantum yield (42%) in toluene for all of our TADF emitters **2**–**5**) but has a lower QY in all other solvents. Compound **5** also displays the largest Stokes' shift (10 520 cm^−1^ in THF) of our compounds. In agreement with the calculated small gaps for **5**, there was no phosphorescence at 77 K, but an emission with a maximum at 469 nm with a pronounced vibrational fine structure was observed. The biexponential decay gives lifetimes of τ_PF_ = 80.3 ns (59%) and τ_DF_ = 3.4 µs (41%). From an Arrhenius plot of the relative lifetimes, we obtained Δ$E_{1_{\text{CT}}-3_{\text{CT}}}$ of 0.03 eV for **5**, which is in excellent agreement with the calculated value of 0.03 eV. The quantum yield of 85% for **5** in PMMA is the highest of the compounds. In combination with the lifetimes of τ_PF_ = 29.4 ns (58%) and τ_DF_ = 5.5 µs (42%), ${}^{{\text{MeO}}_{\text{3}}}\text{Ph}$‐^FMe^π (**5**) is an excellent candidate for further testing in an OLED device.

## 3 Conclusion

We demonstrate how the computationally guided design of excited states leads to efficient TADF emitters. This is accomplished by the development of an accurate theoretical description of the local and charge‐transfer states. A benchmark study shows an excellent agreement between experimentally observed singlet–triplet gaps and calculated values, as indicated by a small mean absolute deviation of 0.05 eV. However, it is important to note that our protocol is limited to cases in which CT and LE states are well defined and are not heavily mixed. The in silico modifications of the donor and bridge moieties allowed us to derive a structure–property relationship, where the tuning of the intersystem crossing and rISC processes can be achieved by modifying the energy of the local excited state at the bridge (^3^LE_π_). In contrast to the local‐excited state at the donor (^3^LE_D_), the ^3^LE_π_ can be tuned independently from the charge‐transfer states. Thus, one strategy to minimize the relevant Δ$E_{1_{\text{CT}}-3_{\text{LE}}}$ and Δ$E_{3_{\text{CT}}-3_{\text{LE}}}$ gaps is to stabilize or destabilize the ^3^LE_π_ state relative to the CT states, which can be achieved by the introduction of acceptor or donor substituents, respectively, at the π‐bridge.

Furthermore, as a proof of concept, five computationally designed D‐A compounds were synthesized and fully characterized. While **1** is a pure fluorescent emitter with a quantum yield of near unity, the decrease of the singlet–triplet gaps in **2**–**5** switches on the delayed fluorescence pathway, exactly as quantum chemically predicted. An added benefit of this strategy is that the emission maxima of these systems can be fine‐tuned and range from deep blue to red.

Depending on the polarity of the solvent, the relative contribution of the delayed fluorescence to the overall decay can be as high as 80%. The overall emission quantum yields in solution for **2**–**5** are lower than that of the pure fluorescent emitter **1**, but increases in a polymer matrix up to 85% for **5**. Compound **5** is an ideal candidate for further research on its application in an optimized light‐emitting device. However, while that is beyond the scope of this paper, we have demonstrated how the combination of computational methods with experimental data leads to new insights into the excited state properties of D‐π‐A compounds and provides a rational strategy to manipulate them.

## Conflict of Interest

The authors declare no conflict of interest.

## Supporting information

Supporting Information
