# Supplementary material for: Computationally Guided Molecular Design to Minimize the LE/CT Gap in D‐π‐A Fluorinated Triarylboranes for Efficient TADF via D and π‐Bridge Tuning
Source: Adv Funct Mater. 2020 Jun 2;30(31):2002064. doi: 10.1002/adfm.202002064 (PMC7405949; doi:10.1002/adfm.202002064)
Supplement: Supplementary file 1 — Supporting Information [file ADFM-30-2002064-s001.pdf]

Copyright WILEY-VCH Verlag GmbH & Co. KGaA, 69469 Weinheim, Germany, 2020.

ADVANCED FUNCTIONAL MATERIALS

Supporting Information

for *Adv. Funct. Mater.,* DOI: 10.1002/adfm.202002064

Computationally Guided Molecular Design to Minimize the LE/CT Gap in D-π-A Fluorinated Triarylboranes for Efficient TADF via D and π-Bridge Tuning

Ayush K. Narsaria, Florian Rauch, Johannes Krebs, Peter Endres, Alexandra Friedrich, Ivo Krummenacher, Holger Braunschweig, Maik Finze, Jörn Nitsch, F. Matthias Bickelhaupt,* and Todd B. Marder**

Supporting Information

Computationally Guided Molecular Design to Minimize the LE/CT Gap in D-π-A Fluorinated Triarylboranes for Efficient TADF via D and π-Bridge Tuning

Ayush Narsaria,[a§] Florian Rauch,[c§] Johannes Krebs,[c] Peter Endres,[c] Dr. Alexandra Friedrich,[c] Dr. Ivo Krummenacher,[c] Prof. Dr. Holger Braunschweig,[c] Prof. Dr. Maik Finze,[c] Dr. Jörn Nitsch,[c] Prof. Dr. F. Matthias Bickelhaupt[a,b*] and Prof. Dr. Todd B. Marder[c*]*

[a)]Department of Theoretical Chemistry, Amsterdam Institute of Molecular and Life Sciences (AIMMS), and Amsterdam Center for Multiscale Modeling (ACMM), Vrije Universiteit Amsterdam, De Boelelaan 1083, 1081 HV Amsterdam, The Netherlands.

E-Mail: f.m.bickelhaupt@vu.nl

[b)]Institute for Molecules and Materials (IMM), Radboud University, Heyendaalseweg 135, NL-6525 AJ, Nijmegen (The Netherlands)

[c)]Institute for Inorganic Chemistry, Julius-Maximilians-Universität Würzburg, Am Hubland, D-97074 Würzburg (Germany) and Institute for Sustainable Chemistry & Catalysis with Boron Julius-Maximilians-Universität Würzburg, Am Hubland, D-97074, Würzburg (Germany) E-Mails: joern.nitsch@uni-wuerzburg.de and todd.marder@uni-wuerzburg.de

[§] These authors contributed equally.

Table of Contents

Detailed description of the methodology & computational details 4

Modeling solvent/matrix effects.. 7

Ground state (S_0) optimization.. 8

Optimal γ parameter in RS functionals.. 8

Excited state calculations.. 10

Phosphorescence emission energy 10

Fluorescence emission energy.. 10

LE/CT gaps 11

Discussion 18

Synthesis and characterization 21

General experimental details 21

Column chromatography 22

NMR spectra 22

Crystal structure determination 22

Photophysical measurements 23

Electrochemical measurements 24

Synthetic procedure 25

2-iodo-1,3-bis(trifluoromethyl)benzene (FXylI) 25

Bis(2,6-bis(trifluoromethyl)phenyl)fluoroborane (FB(FXyl)$_2$) 25

9-(4-(bis(2,6-bis(trifluoromethyl)phenyl)boryl)phenyl)carbazole (1) 26

9-(4-bromo-2,6-dimethylphenyl)carbazole (ii) 27

9-(4-(bis(2,6-bis(trifluoromethyl)phenyl)boryl)-2,6-dimethylphenyl)carbazole (2) 28

2-(4-iodo-3,5-dimethylphenyl)-4,4,5,5-tetramethyl-1,3,2-dioxaborolane 29

Potassium(4-iodo-3,5-dimethylphenyl)trifluoroborate 30

Bis(2,6-bis(trifluoromethyl)phenyl)(4-iodo-3,5-dimethylphenyl)borane (iii) 31

10-(4-(bis(2,6-bis(trifluoromethyl)phenyl)boryl)-2,6-dimethylphenyl) phenoxazine (3) 32

Bis(2,6-bis(trifluoromethyl)phenyl)(3,5-dimethoxyphenyl)borane (iv’) 33

Bis(2,6-bis(trifluoromethyl)phenyl)(4-iodo-3,5-dimethoxyphenyl)borane (iv) 34

10-(4-(bis(2,6-bis(trifluoromethyl)phenyl)boryl)-2,6-dimethoxyphenyl)phenoxazine (4) 35

2,4,6-trimethoxy-2',6'-bis(trifluoromethyl)-1,1'-biphenyl 36

4,4,5,5-tetramethyl-2-(2',4',6'-trimethoxy-2,6-bis(trifluoromethyl)-[1,1'-biphenyl]-4-yl)-1,3,2-dioxaborolane 37

Potassium(2',4',6'-trimethoxy-2,6-bis(trifluoromethyl)-[1,1'-biphenyl]-4-yl)trifluoroborate 38

Bis(2,6-bis(trifluoromethyl)phenyl)(2',4',6'-trimethoxy-2,6-bis(trifluoromethyl)-[1,1'-biphenyl]-4-yl)borane 39

Structures in the solid state 40

NMR spectra 51

Photophysical data 81

Electrochemical data 84

XYZ coordinates 85

References 110

Detailed description of the methodology & computational details

Methodology & Computational Details

All calculations were performed using the Amsterdam Density Functional (ADF) 2018 quantum chemistry package developed by SCM.[1] Electronic ground-state geometry optimizations were performed with the dispersion corrected BLYP-D3(BJ)[2] functional in combination with the TZ2P basis set and small frozen core approximation, i.e., the 1s electrons for second period elements were kept frozen.

Relativistic effects were accounted for using the scalar zeroth-order regular approximation (ZORA).[3] In order to account for solvation effects, the calculations were conducted with the COSMO formalism in toluene ($\varepsilon = 2.38$), except for the benchmark systems **B4–B8** which were optimized in their respective media. Geometries were verified by analytical frequency calculations to be (local) minima.[4] The optimal γ value in a range-separated (RS) functional, in our case LC-BLYP, was determined by the Janak's theorem[5] (Equation S1) by minimizing the $J(\gamma)$ function.[6]

$$J(\gamma) = \sqrt{\sum_{i=0}^{1}[\varepsilon_H(N+i) + IP(N+i)]} \tag{S1}$$

This scheme is well-known to provide accurate values for CT states.[6d, 7] Calculations to find the optimal γ value (tuning calculations) were performed in the gas phase in combination with the TZ2P functional on the BLYP-D3(BJ) optimized ground state geometries. We used PLAMS[1c] as implemented in ADF and the open-source python library QMFlows[8] to automate the job submission and handling procedure. The vertical or Franck-Condon excitation energies (FC-S_n and FC-T_n) were calculated using the optimally tuned LC-BLYP* functional (where * denotes the tuned LC-BLYP functional) in combination with the TZ2P basis set and without a frozen core within the Tamm-Dancoff approximation (TDA). This approximation has been extensively tested. It saves computational time and avoids triplet instability issues.[7b, 9] We also performed Natural Transition Orbital (NTO)[10] analyses as some of the excited states exhibit heavily mixed electronic configurations. Moreover, NTO allows for qualitative characterization of the excited states as local excited (LE_D: localized triplet exciton on the donor, LE_π: localized triplet exciton on the π-bridge, or LE_A: localized triplet exciton on the acceptor) and charge transfer (CT). Furthermore, metrics such as Tozers index (Λ)[11] (Equation S2) in conjunction with the hole–electron distance descriptor (R_{eh})[12] (Equation S3) were also employed to quantify the nature of excited states. It has to be noted that excited states involved in TADF processes are sometimes a mixture of CT/LE and not “pure” LE or CT.[13] However, in order to characterize the different excited states, and to ensure a fair comparison between them, we assign, although not strictly, $\Lambda > 0.5$ and $R_{eh} < 2.0$ Å as LE and the opposite as CT.

$$\Lambda = \frac{\sum_{i,a} c_{i,a}^2 \langle |\varphi_a| | |\varphi_i| \rangle}{\sum_{i,a} c_{i,a}^2} \quad \text{(S2)}$$

$$R_{\text{eh}} = \frac{\sum_{i,a} c_{i,a}^2 |\langle \varphi_a | r | \varphi_a \rangle - \langle \varphi_i | r | \varphi_i \rangle|}{\sum_{i,a} c_{i,a}^2} \quad \text{(S3)}$$

In the equations above,φ_a and φ_i represents the occupied and unoccupied one-electron wavefunction respectively, $|\langle \varphi_a | r | \varphi_a \rangle|$ represents the norm of the one-electron wavefunction centroid and $c_{i,a}$ represents the weight of the one-electron excitation. As TADF is an emission process, and there are multiple close-lying triplet excited states involved in the TADF mechanism which might lead to state crossing, it is crucial to determine which vertically excited FC-T_n (where n can be > 1) state relaxes to the adiabatic T_1 state. For this reason, we plotted the electron difference density profile ($\Delta\rho = \rho_{T_1}(T_1) - \rho_{S_0}(T_1)$) which qualitatively maps the nature of the relaxed T_1 to the corresponding FC-T_n state; $\rho_{T_1}(T_1)$ and $\rho_{S_0}(T_1)$ is computed using UKS and RKS, respectively, at the LC-BLYP*/TZ2P/COSMO level of theory without a frozen core approximation.

Except for **B1** and **Cbz-Ph** to some extent and especially **B7** where there is mixture of ^{3}CT and ^{3}LE, we found that the relaxed T_1 state is essentially a ^{3}CT state. Similar situations, in which the ^{3}CT state is lower in energy than the ^{3}LE state, have been observed previously.[14] We also evaluated the $S_0 \leftarrow T_1$ emission energy (Figure S1), denoted as $\Delta E_{\text{VE}}(T_1)$, as well as the $S_0 \leftarrow S_1$ emission energy, denoted as $\Delta E(S_1)$. As stated in the manuscript, the excited states in the TADF mechanism are in dynamic equilibrium with each other which in turn is intricately dependent on the corresponding energy gaps. Therefore, we report three energy gaps (differences in vertical excitation energies obtained from TDA-LC-BLYP*) in this study: $\Delta E_{^1\text{CT}-^3\text{CT}}$, $\Delta E_{^1\text{CT}-^3\text{LE}}$, and $\Delta E_{^3\text{LE}-^3\text{CT}}$. Here, ^{3}CT denotes the FC-T_n corresponding to the relaxed T_1 state and ^{3}LE denotes the lowest FC-T_n with predominant LE character. An important aspect which is often overlooked when benchmarking S–T gaps is the actual measurement method involved to extract the experimental gaps. The gap can be extracted in two ways, first from fitting the integrated delayed fluorescence (DF) emission or its lifetime as a function of temperature which yields the activation energy ($\Delta E_{\text{a}}^{\text{TADF}}$), and second, from the difference between the onset of fluorescence and phosphorescence signals at low temperature which is defined as the optical gap ($\Delta E_{\text{opt}}^{\text{TADF}}$).[15]

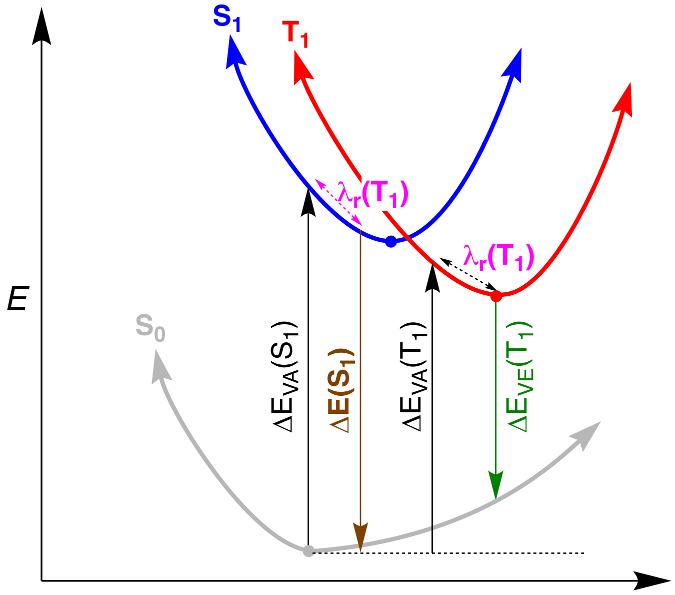

Figure S1. Jablonski diagram depicting the protocol used to compute the emission energies: $\Delta E(S_1)$ in brown; and $\Delta E_{VE}(T_1)$ in green. The geometrical reorganization energy $\lambda_r(T_1)$ is depicted in pink and the vertical Franck-Condon absorption energies, $\Delta E_{VA}(S_1)$ and $\Delta E_{VA}(T_1)$, are depicted in black. The bold dots depict the minima of the corresponding electronic states. The geometrical reorganization energy $\lambda_r(T_1)$ is determined by the energy difference between the relaxed T_1 and the corresponding FC-Tn state. The $S_0 \leftarrow S_1$ emission energy ($\Delta E(S_1)$) was approximated by subtracting $\lambda_r(T_1)$ from FC-S_1, i.e. by assuming that the reorganization energies for singlet and triplet (both CT states) are same.

We benchmarked our protocol using a set of eight known boron-based D-π-A compounds (**B1–B8,** see Figure S2).[16] For this purpose, we compared the experimental singlet–triplet (S–T) gaps with our computed ΔE_{ST}. The experimentally measured gap depends on the type of measurement performed and thus it is highly crucial to benchmark against the correct gap.[15a] As such, the gap obtained from $\Delta E_a^{\mathrm{TADF}}$ correspond to $\Delta E_{^1\mathrm{CT}-^3\mathrm{CT}}$, while the optical gap corresponds to $\Delta E_{^1\mathrm{CT}-^3\mathrm{LE}}$.

Overall, our protocol can successfully predict the three gaps in an unknown molecule D-π-A except in cases where the assignment of the nature of the excited states as CT or LE becomes ill defined vide infra.[17]

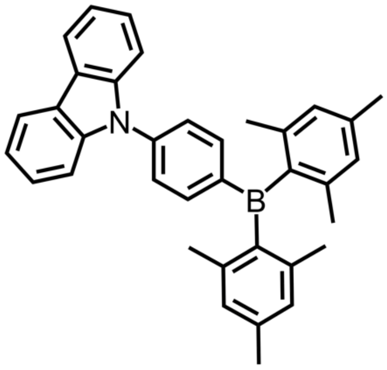

B1

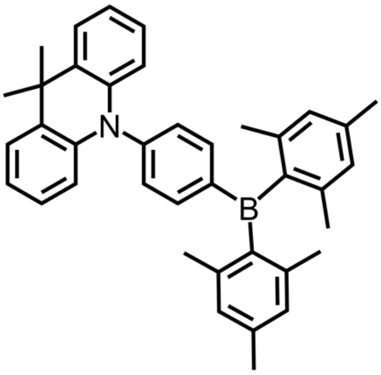

B2

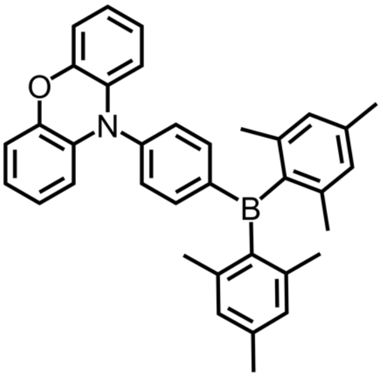

B3

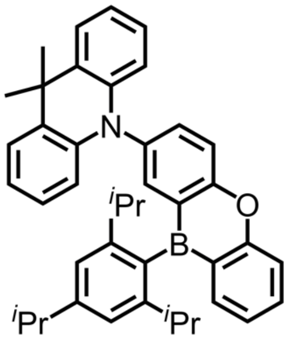

B4

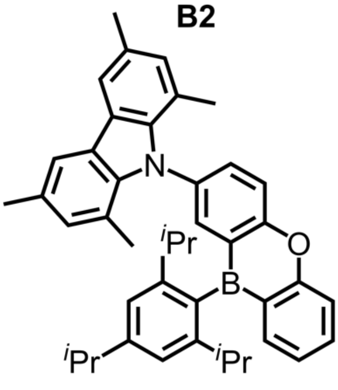

B5

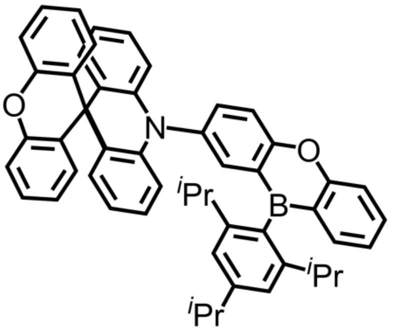

B6

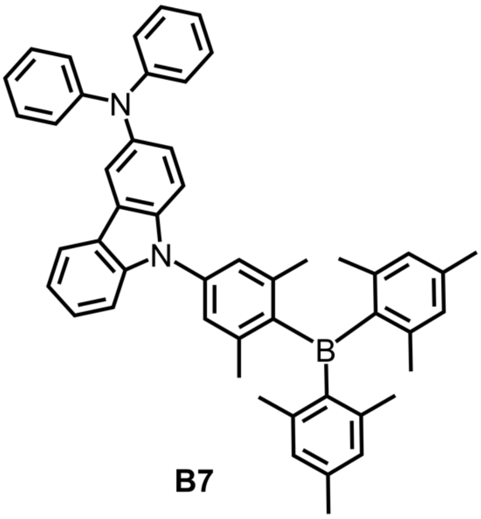

B7

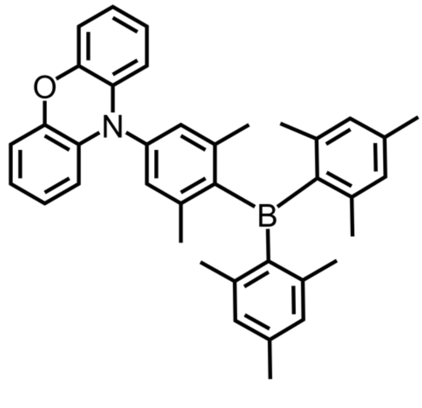

B8

Figure S2. Structures of the molecules **B1** – **B8**[16] used in our benchmark study. **B1** – **B4** were measured in frozen toluene, **B4-B6** in poly(propylene)fumarate (PPF), **B7** in bis[2-(diphenylphosphino)phenyl] ether oxide (DPEPO) and **B8** in 4,4′-bis(N-carbazolyl)-1,1′-biphenyl (CBP).

Modeling solvent/matrix effects

We benchmarked our method using a set of eight known boron-based D–π-A compounds (**B1–B8,** Figure S2).[16] For this purpose, we compared the experimental singlet-triplet (S–T) gaps measured in toluene at 77 K or at lower temperatures with our estimated $\Delta E_{S\text{-}T}$ computed in toluene. However, the gaps in **B4–B8** were measured in amorphous thin-film matrices. As the matrix, poly(propylene)fumarate (PPF) was used in the cases of **B4–B6**, bis[2-(diphenylphosphino)phenyl]

ether oxide (DPEPO) in the case of **B7**, and 4,4′-bis(*N*-carbazolyl)-1,1′-biphenyl (CBP) in the case of **B8**. Therefore, to mimic the environment of the amorphous films we calculated its static dielectric constant ε using the Clausius-Mossotti relation[18] (Equation S4):

$$\frac{\varepsilon - 1}{\varepsilon + 2} = \frac{4\pi\alpha}{3V} \qquad \text{(S4)}$$

where V is the surface volume obtained from the continuum solvation model (COSMO)[19] calculation using atomic radii and α term denotes the isotropic component of the molecular polarizability obtained from the response calculation.[20] We calculated these parameters with the range-separated (RS) CAMY-B3LYP functional[21] using the TZ2P slater-type basis set.[22]

Table S1. Calculated permittivity using Equation S4 of the amorphous thin films used to measure the S–T gap in the selected benchmark molecules (**B4**–**B8**).

**Thin film**	**Volume (V in bohr^3)**	**Polarizability (α in bohr^3)**	**ε**
PPF	3930.18	456.80	3.75
DPEPO	3983.89	466.76	3.88
CBP	3375.52	449.56	4.78

Ground state (S_0) optimization

Electronic ground-state geometry optimizations were performed with the dispersion corrected BLYP-D3(BJ) functional[2c, 2d, 2f, 23] in combination with the TZ2P basis set and small frozen core approximation (i.e., the 1s electrons for second period elements were kept frozen) using restricted Kohn-Sham (RKS). Relativistic effects were accounted for using the scalar zeroth-order regular approximation (ZORA).[24] In order to account for the solvation effects, all calculations were conducted with the COSMO formalism in toluene (ε = 2.38). Due to the different environment for **B4**–**B8**, as mentioned above, all the optimizations for these systems were done using their respective permittivity as described in Table S1. All optimized structures were verified by analytical frequency calculations to be (local) minima.[4]

Optimal γ parameter in RS functionals

A range-separated (RS) functional, in our case ZORA-LC-BLYP, separates the exchange term into two components: short-range component which is described by DFT exchange and the long-range component which is described by the Hartree-Fock (HF) exchange, based on the interelectronic parameter r_{12} and a switching function. In ADF, the switching function is based on the Yukawa potential

exp(−γr_{12}),[21] where γ represents the inverse of the inter-electronic distance r_{12} at which the exchange term switches from DFT-like to HF-like exact exchange as described by Equation S5 (in our case α = 0 and β = 1). According to exact Kohn-Sham and generalized Kohn-Sham theory, the negative HOMO energy $-\varepsilon_{\mathrm{H}}$(N) for an N-electron system should be equal to the vertical ionization potential IP(N).[5, 25] Most of the DFT functionals give a large difference between the two quantities, and therefore, the optimally tuned version tries to minimize this difference. The formalism of the N-electron system can be extended to an $N+1$ electron system to provide a better estimation of the HOMO–LUMO gap as described by Equation S6. The optimal γ for a particular molecule is obtained by minimizing the J(γ) function.[6] This approach has been applied to all the benchmark and model systems as they are known to provide accurate values for the gap.[6d, 7] All tuning calculations have been performed in the gas phase in combination with the TZ2P functional on the BLYP-D3(BJ) optimized ground state geometries. We used PLAMS as implemented in ADF2018 and the open-source python library QMFlows to automate the job submission and handling procedure and to obtain the optimal γ for each molecule (Table S2).

$$\frac{1}{\mathrm{r}_{12}} = \frac{1-[\alpha+\beta\,(1-\exp(-\gamma \mathrm{r}_{12})]}{\mathrm{r}_{12}} + \frac{\alpha+\beta\,(1-\exp(-\gamma \mathrm{r}_{12})}{\mathrm{r}_{12}} \tag{S5}$$

$$J(\gamma) = \sqrt{\sum_{i=0}^{1}[\varepsilon_H(N+i) + IP(N+i)]} \tag{S6}$$

Table S2. Self-consistently tuned γ (in a_0^{-1}) for the benchmark molecules with ZORA-LC-BLYP*/TZ2P/COSMO (toluene) level of theory.

Benchmark molecule	Optimum γ
**B1**	0.28
**B2**	0.26
**B3**	0.28
**B4**	0.28
**B5**	0.26
**B6**	0.26
**B7**	0.26
**B8**	0.28

Excited state calculations

Vertical or Franck-Condon excitation energies (FC-S_n and FC-T_n) were calculated using the optimally tuned LC-BLYP* functional (where * denotes the tuned LC-BLYP functional) in combination with the TZ2P basis set within the Tamm-Dancoff approximation (TDA). This approximation was employed as it saves computational time and avoids triplet instability issues.[7b, 9] We did not use a frozen core approximation, in other words, all the electrons were correlated. To account for the solvation effect, we used the non-equilibrium solvation model described in COSMO with toluene as the condensed phase (see Modeling Solvent section). Non-equilibrium solvation is necessary to account for the slow relaxation of the solvent nuclei compared to its electrons. For this purpose, only the optical part of the dielectric constant (dynamic dielectric constant) is considered in the TDA calculations, which in the case of toluene, corresponds to a value of 2.24. For **B4**–**B8** we used a fixed dynamic dielectric constant of 2.0 as the change in the excitation energies is negligible with change in the dynamic dielectric constant parameter.[26]

Phosphorescence emission energy

To obtain the relaxed ground triplet state (T_1) we employed unrestricted Kohn-Sham (UKS) at the LC-BLYP*/TZ2P level of theory with no frozen core approximation. Again, these calculations were performed using the equilibrium COSMO solvation model with toluene as the solvent except for **B4**–**B8**, which were computed at their respective permittivity noted in Table S1. We also performed single point energy calculations using RKS at the optimized T_1 geometry to estimate the $S_0 \leftarrow T_1$ vertical emission energy $\Delta E_{VE}(T_1)$. The difference between the energies obtained from the latter calculation and the relaxed T_1 state provides $\Delta E_{VE}(T_1)$, the phosphorescence emission energy maximum. To comment on the nature of the relaxed T_1 excited state, we plotted electron difference density profile ($\Delta\rho = \rho_{T_1}(T_1) - \rho_{S_0}(T_1)$) which is essential to map the relaxed T_1 qualitatively to the corresponding FC-T_n state (Figure S7 and Figure S8). We found, in all cases, that the relaxed T_1 state is essentially a ^{3}CT state (except for **B7** in the benchmark systems). A similar situation has been observed previously in other systems in which the ^{3}CT state is lower in energy than the ^{3}LE state.[14]

Fluorescence emission energy

The geometrical reorganization energy $\lambda_r(T_1)$, or the T_1 relaxation energy, is determined by the energy difference between the LC-BLYP* relaxed T_1 state and the corresponding FC-T_n state. This leads to correct local minima for computing accurate $\lambda_r(T_1)$. To see how much the change in geometry changes the optimal γ we again performed the self-consistent tuning of the γ parameter on some test cases. We

found that the change in the optimal γ value is negligible and so is the change in vertical excitation energies. The $S_0 \leftarrow S_1$ emission energy ($\Delta E(S_1)$) was approximated as the difference between the FC-S_1 and the $\lambda_r(T_1)$. This protocol of estimating the fluorescence emission energy involves approximations. First, we assume that the relaxation of CT states, or a mixture thereof, is larger than the LE state due to the electron and hole occupying different regions in space leading to more reorganization of the electron density upon relaxation, which is generally the case. Moreover, the transitions to ^{3}CT corresponding to the relaxed T_1 and ^{1}CT are composed mostly of the same pair of MOs, e.g., HOMO → LUMO (Table S 3, except for **B7**). Second, the $\Delta E(S_1)$ computed from the above-mentioned formalism is blue-shifted as it does not correspond to the true vertical emission energy maximum, but resembles the adiabatic energy or the onset of fluorescence emission ($\Delta E^{0\text{-}0}(S_1)$) without the zero-point vibrational energy (ZPVE) correction term. Figure S1 describes the approximations and the formalism used to approximate the fluorescence emission energy ($\Delta E(S_1)$). As it is not the vertical emission energy, we refrain from calling it so, and thus named it $\Delta E(S_1)$.

LE/CT gaps

The mechanism of TADF might involve several higher excited states which have small energy gaps between them resulting in a dynamic equilibrium with each other.[27] As a result, the kinetics of the individual steps involved in the reverse intersystem crossing (rISC) depends intricately on the energy gaps between these excited states. Therefore, we benchmarked and report three gaps (differences in vertical excitation energies obtained from TDA-LC-BLYP*) in this study: $\Delta E_{^1\mathrm{CT}-^3\mathrm{CT}}$, $\Delta E_{^1\mathrm{CT}-^3\mathrm{LE}}$, and $\Delta E_{^3\mathrm{LE}-^3\mathrm{CT}}$. Here ^{3}CT denotes the FC-T_n corresponding to the relaxed T_1 state and ^{3}LE denotes the lowest FC-T_n with predominant LE character. An important aspect which is often overlooked when benchmarking the S–T gaps is the actual method of measurement involved to extract the experimental gaps. Thus, the gap can be extracted in two ways, first from fitting the integrated delayed fluorescence (DF) emission as a function of temperature which gives the activation energy ($\Delta E_a^{\mathrm{TADF}}$), and second, from the difference between the onset of fluorescence and phosphorescence signals which is defined as the optical gap. Recently, Gibson and Penfold reported how non-adiabatic coupling derived from the involvement of an intermediate triplet state can reduce the activation energy in the rISC process and can give rise to two different energy gaps corresponding to the optical gap and the activation energy.[15] For this reason, the experimentally measured gap can change depending on the type of measurement performed and so it becomes highly crucial to benchmark the correct gap. As such, the gap obtained from the activation energy measurements correspond to $\Delta E_{^1\mathrm{CT}-^3\mathrm{CT}}$, while the optical gap corresponds to $\Delta E_{^1\mathrm{CT}-^3\mathrm{LE}}$. Compound **B8** serves as the perfect example to demonstrate the effect of comparing the correct computed gap with the experimental S–T gap. In this case, Kaji and coworkers[16b] measured the

gap using the activation energy obtained from the Arrhenius plot, so, $\Delta E_{1_{\mathrm{CT}}-3_{\mathrm{CT}}}$ corresponds to the measured gap resulting in an error of 0.06 eV, while comparing $\Delta E_{1_{\mathrm{CT}}-3_{\mathrm{LE}}}$ results in an error of 0.22 eV. The third gap $\Delta E_{3_{\mathrm{LE}}-3_{\mathrm{CT}}}$ determines the strength of the non-adiabatic coupling between the triplet states involved in the reverse internal conversion (rIC). Smaller $\Delta E_{3_{CT}-3_{\mathrm{LE}}}$ facilitates faster k_{rISC}.[15b, 27c]

Table S 3. Calculated photophysical data for **B1-B8**. The table shows the vertical excitation energies (in eV and nm) with its corresponding, charge transfer metrics (Λ and R_{eh} in Å), assignment of the excited states along with the MO composition (configuration interaction, CI) of the transition (in %), the computed S–T gaps and the emission energies ($\Delta E(S_1)$ and $\Delta E_{VE}(T_1)$) in eV and nm. Experimental values in curly brackets. Fields in bold represents the mapping of the FC-T_n state corresponding to the relaxed T_1 state according to the difference density plots.

**Cpd.**	**State**	**Energy**		**Assign.**	**CI (%)**	**Λ**[a)]	**R_{eh}**[b)] **[Å]**	**$\Delta E_{^1CT-^3LE}$**	**$\Delta E_{^1CT-^3CT}$**	**$\Delta E_{^3CT-}$**
		**[eV]**	**[nm]**					**[eV]**	**[eV]**	**[eV]**
**B1**	**FC-T_1**	**3.21**	**386**	**$^3CT/^3LE_\pi$**	**H→L (57), H-7→L (17)**	**0.47**	**3.71**	0.36 {0.46}	0.46	0.10
	FC-T_2	3.31	375	$^3LE_A/^3CT$	H-2→L (81)	0.52	2.02			
	FC-T_3	3.45	359	^3LE_A	H-3→L(60)	0.57	1.01			
	FC-T_4	3.45	359	^3LE_D	H→L+1 (88)	0.62	1.29			
	FC-T_5	3.54	350	^3LE_D	H-1→L +1(85)	0.76	0.02			
	FC-S_1	3.67	338	^1CT	H→L (70), H-3→L (17)	0.42	3.87			
	$\Delta E(S_1)$	3.37 {3.13}	368 {398}	-						
	$\Delta E_{VE}(T_1)$	2.65 {2.81}	468 {442}	$^3CT/^3LE_\pi$						
**B2**	**FC-T_1**	**3.20**	**388**	**^3CT**	**H→L (84)**	**0.14**	**5.82**	0.04 {0.04}	0.01	0.05
	FC-S_1	3.21	386	^1CT	H→L (87)	0.13	6.04			
	FC-T_2	3.25	382	^3LE_A	H-1→L(84)	0.52	1.85			
	FC-T_3	3.35	370	^3LE_A	H-2→L (77)	0.57	1.35			
	FC-T_4	3.44	360	^3LE_D	H→L +4(65)	0.60	1.27			
	FC-T_5	3.57	347	^3LE_D	H→L+3(90),	0.65	0.08			
	$\Delta E(S_1)$	2.70 {2.66}	459 {466}	-						
	$\Delta E_{VE}(T_1)$	2.45 {2.66}	506 {466}	^3CT						
**B3**	FC-T_1	3.07	404	^3LE_D	H→L+2 (40), H→L+1 (33)	0.51	2.75	0.05 {0.03}	0.02	0.03
	**FC-T_2**	**3.10**	**400**	**^3CT**	**H→L (79)**	**0.19**	**5.64**			
	FC-S_1	3.12	397	^1CT	H→L(85)	0.17	5.88			
	FC-T_3	3.27	379	^3LE_A	H-1→L(83)	0.51	1.92			
	FC-T_4	3.37	368	^3LE_A	H-2→L(70)	0.54	0.73			
	FC-T_5	3.46	358	^3LE_D	H→L+6(32), H→L+1(26)	0.5	2.86			
	$\Delta E(S_1)$	2.48 {2.49}	500 {498}	-						
	$\Delta E_{VE}(T_1)$	2.25 {2.45}	551 {506}	^3CT						
**B4**	FC-T_1	3.27	379	$^3LE_\pi$	H-3→L (81)	0.63	0.81	0.03 {0.10}	0.02	0.01
	**FC-T_2**	**3.28**	**378**	**^3CT**	**H→L (74)**	**0.23**	**5.04**			
	FC-S_1	3.30	376	^1CT	H→L(85)	0.17	5.66			
	FC-T_3	3.48	356	^3LE_D	H→L+4(56), H→L+2(23)	0.54	2.23			
	FC-T_4	3.61	343	^3LE_D	H→L+3(91)	0.65	0.18			
	FC-T_5	3.76	330	$^3LE_\pi$	H-6→L(51), H-7→L(20)	0.59	1.16			
	$\Delta E(S_1)$	2.85 {2.97}	435 {417}	-						
	$\Delta E_{VE}(T_1)$	2.64 {2.87}	470 {432}	^3CT						

-Table S 3. continued-

**B5**	FC-T_1	3.23	384	^3LE_A	H-4→L (89)	0.67	0.62	0.19 {0.12}	0.03	0.16
	FC-T_2	3.27	379	^3LE_D	H→L+1 (90)	0.67	0.26			
	**FC-T_3**	**3.39**	**366**	**^3CT**	**H→L(78)**	**0.21**	**5.76**			
	FC-T_4	3.50	354	^3LE_A	H-1→L+1(81)	0.72	0.56			
	FC-S_1	3.42	363	^1CT	H→L(87)	0.16	6.24			
	FC-T_5	3.72	333	^3LE_A	H-2→L(88),	0.24	3.7			
	ΔE(S_1)	2.96 {3.11}	419 {399}							
	ΔE_{VE}(T_1)	2.72 {2.99}	456 {415}	^3CT						
**B6**	FC-T_1	3.24	383	^3LE_A	H-3→L (87)	0.62	1.62	0.08 {0.06}	0.02	0.06
	**FC-T_2**	**3.30**	**376**	**^3CT**	**H→L(79)**	**0.17**	**5.4**			
	FC-S_1	3.32	373	^1CT	H→L (86)	0.15	5.71			
	FC-T_3	3.43	362	^3LE_D	H→L+1(64)	0.48	0.55			
	FC-T_4	3.6	344	^3LE_D	H→L+4(91)	0.65	0.42			
	FC-T_5	3.73	332	^3CT	H-2→L(92)	0.21	3.9			
	ΔE(S_1)	2.93 {3.01}	423 {412}							
	ΔE_{VE}(T_1)	2.68 {2.95}	463 {420}	^3CT						
**B7**	FC-T_1	3.00	413	^3LE_D	H→L+1 (82)	0.56	2.25	0.48	0.36 {0.06}	0.12
	**FC-T_2**	**3.12**	**397**	**$^3LE_\pi$/^3CT**	**H-1→L (29), H-4→L (25), H→L (16)**	**0.42**	**4.28**			
	FC-T_3	3.14	395	^3LE_A	H-3→L(86)	0.57	1.42			
	FC-T_4	3.30	376	^3LE_D	H→L+3(75)	0.58	1.73			
	FC-T_5	3.35	370	^3LE_A	H-4→L(43)	0.56	1.18			
	FC-T_6	3.43	362		H→L(8)	0.63	0.94			
	FC-S_1	3.46	358	^1LE_D	H-2→L+1(24), H-2→L+6(22)	0.56	2.51			
	FC-S_2	3.48	356	^1LE_D/^1CT	H→L+1(86)	0.35	5.73			
	ΔE(S_1)	3.09 {2.60}	401 {477}							
	ΔE_{VE}(T_1)	2.44	508	$^3LE_\pi$ /^3CT						
**B8**	FC-T_1	3.03	409	^3LE_D	H→L+1 (66), H→L+3 (25)	0.61	1.27	0.34	0.01 {0.07}	0.33
	FC-T_2	3.15	394	^3LE_A	H-1→L (86)	0.57	1.32			
	FC-T_3	3.26	380	^3LE_A	H-2→L(83)	0.63	0.61			
	**FC-T_4**	**3.36**	**369**	**^3CT**	**H→L(76)**	**0.12**	**6.42**			
	FC-S_1	3.37	368	^1CT	H→L(80)	0.1	6.66			
	FC-T_5	3.45	359	^3LE_D	H→L+3(48), H→L+1(12)	0.5	2.81			
	ΔE(S_1)	2.51 {2.43}	494 {509}							
	ΔE_{VE}(T_1)	2.26	549	^3CT						

a) Degree of spatial overlap between occupied and virtual orbitals involved in the excitation: $\Lambda = \frac{\sum_{i,a} c_{i,a}^2 \langle \psi_a | \psi_i \rangle}{\sum_{i,a} c_{i,a}^2}$

b) Hole-electron distance: $R_{\mathrm{eh}} = \frac{\sum_{i,a} c_{i,a}^2 |\langle \psi_a | r | \psi_a \rangle - \langle \psi_i | r | \psi_i \rangle|}{\sum_{i,a} c_{i,a}^2}$

Table S4. Statistical parameters (signed error, mean deviation, and mean absolute deviation, all in eV) based on benchmarking the S–T gap and emission energies for all of the benchmark molecules. Left: including compound **B7**; right: without **B7**.

**Molecule**	$\Delta\Delta E_{S\text{-}T}$	$\Delta\Delta E(S_1)$	$\Delta\Delta E_{VE}(T_1)$
**B1**	-0.10	0.24	-0.16
**B2**	0.00	0.04	-0.21
**B3**	0.02	-0.01	-0.20
**B4**	-0.07	-0.12	-0.23
**B5**	0.07	-0.15	-0.27
**B6**	0.02	-0.08	-0.27
**B7**	0.30	0.49	–
**B8**	-0.06	0.08	–
MD	0.02	0.06	-0.22
MAD	0.08	0.15	0.22

**Molecule**	$\Delta\Delta E_{S\text{-}T}$	$\Delta\Delta E(S_1)$	$\Delta\Delta E_{VE}(T_1)$
**B1**	-0.10	0.24	-0.16
**B2**	0.00	0.04	-0.21
**B3**	0.02	-0.01	-0.20
**B4**	-0.07	-0.12	-0.23
**B5**	0.07	-0.15	-0.27
**B6**	0.02	-0.08	-0.27
**B8**	-0.06	0.08	–
MD	-0.02	0.00	-0.22
MAD	0.05	0.10	0.22

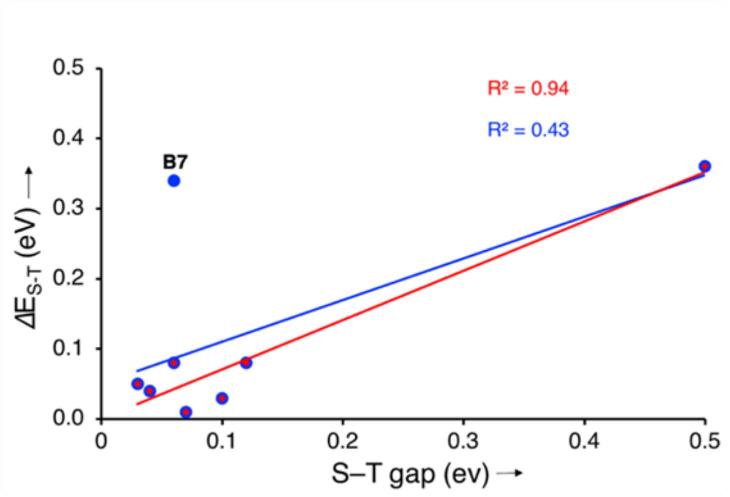

Figure S3. Correlation between the experimentally obtained S–T gap and the computed gap $\Delta E_{\text{S-T}}$. Linear fit in blue fits all molecules in the benchmark test set while the linear fit in red excludes **B7**.

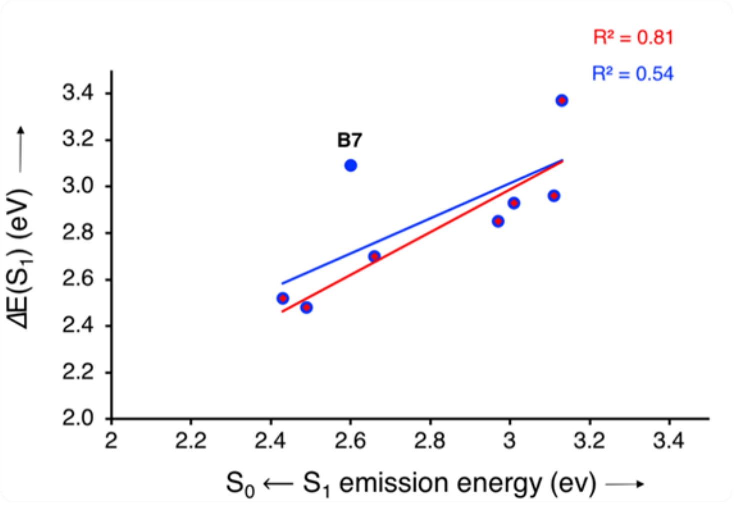

Figure S4. Correlation between the experimentally obtained $S_0 \leftarrow S_1$ emission energy and the computed fluorescence emission energy $\Delta E(S_1)$. Linear fit in blue fits all molecules in the benchmark test set while the linear fit in red excludes **B7**.

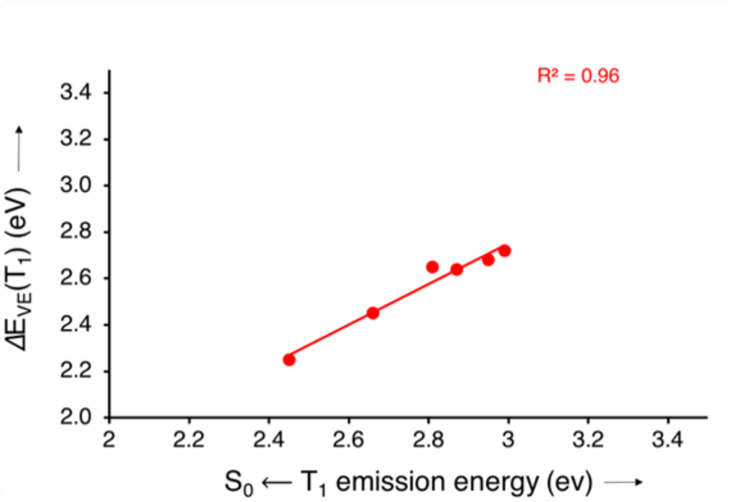

Figure S5. Correlation between the experimentally obtained $S_0 \longleftarrow T_1$ emission energy and the computed phosphorescence emission energy $\Delta E_{VE}(T_1)$.

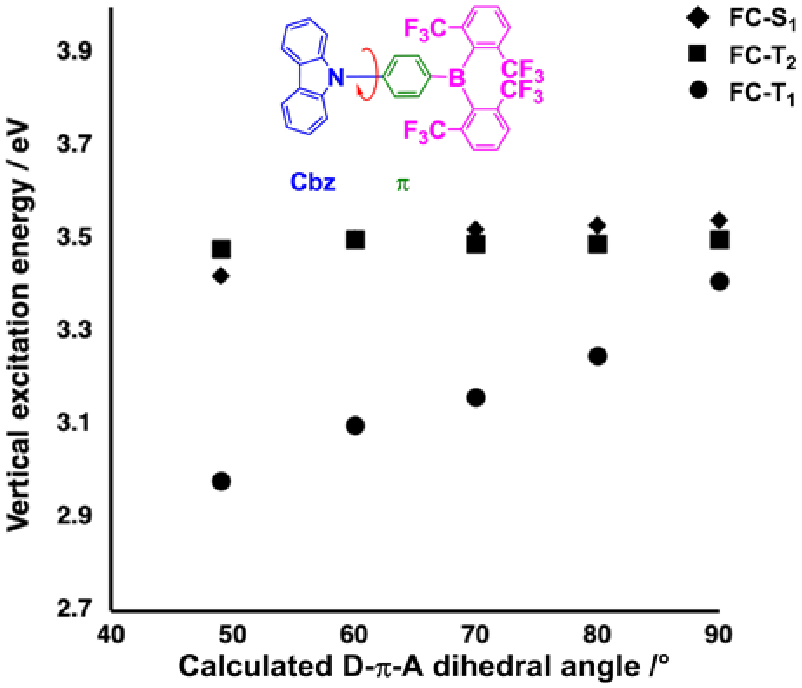

Figure S6. Modulation of calculated excited state energy levels upon increase in the torsional angle in intervals of 10° between the donor (Cbz) and the π-bridge–acceptor (1,4-C_6H_4-B(^{F}Xyl)$_2$) in **Cbz-π** (**1**).

Table S5. Frontier molecular orbital energies and HOMO–LUMO gaps of the various donors and bridges used in the model systems computed at the BLYP-D3(BJ)/TZ2P/COSMO (toluene) level.

**Donor**	**HOMO [eV]**	**LUMO [eV]**	$\Delta E^{\mathbf{D}}_{\mathbf{H\text{–}L}}$ **[eV]**
^{MeO_3}Ph-H	–5.2	–0.6	4.6
Cbz-H	–5.0	–1.8	3.2
^{Me}Cbz-H	–4.9	–1.6	3.3
Phox-H	–4.3	–1.4	2.9
***π*-Bridge**			$\Delta E^{\boldsymbol{\pi}}_{\mathbf{H\text{–}L}}$ **[eV]**
^{FMe}***π***-H_2	–7.2	–2.4	4.8
π-H_2	–6.3	–1.2	5.1
^{Me}***π***-H_2	–5.8	–1.0	4.8
^{MeO}***π***-H_2	–5.1	–0.9	4.2

Discussion

The optimal γ values for all benchmark molecules fall in a very small window between 0.26-0.28 a_0^{-1} (Table S2). This would mean that the interelectronic distance where the DFT exchange at short-range changes to HF exact exchange at long-range is very similar in these systems. Table S4 lists the performance of our formalism to compute the S–T gap and the emission energies based on statistical parameters such as the mean deviation (MD) and mean absolute deviation (MAD). Our results show excellent accuracy to predict the S–T gap with an MD of 0.02 eV and an MAD of 0.08 eV. The maximum error is found for **B7** (0.30 eV). This large error stems from the difficulty in assigning the correct ^{3}CT state which relaxes to T_1 resulting from a heavy mixing between ^{3}LE and ^{3}CT. In this case, the excited states are either heavily mixed together with no predominant pair of MOs involved in the transition, or the assignment of the transition based on Λ and R_{eh} parameters is inconclusive. We selected FC-S_2 as the state with higher CT character and the state which relaxes to relaxed S_1 state due to favorable Λ and R_{eh} parameters (Λ = 0.345 vs. 0.555 and R_{eh} = 5.73 Å vs 2.51 Å) as well as close-lying energies (0.02 eV), compared to FC-S_1, albeit the assignment is not strictly ^{1}CT. Considering the composition of the transitions based on MOs, FC-T_2 is composed of a heavy mixture of MOs which are similar to that of FC-S_2 and contains a small component from HOMO → LUMO (33% and 16% in case of FC-S_2 and FC-T_2, respectively) indicating a small CT nature in both states. However, the MO composition of the transition to FC-T_5 also has a small HOMO → LUMO component (7%), but the charge transfer metrics are unfavorable compared to FC-T_2. Mapping the difference density profile of the relaxed T_1 state (Figure S7) provides the closest match to the NTO derived from FC-T_2 state, thereby indicating FC-T_2 might be the relaxed T_1 state. We also considered higher FC-T_n states where n > 5, but, all of them were predominantly ^{3}LE in nature. Therefore, we assumed FC-S_2 and FC-T_2 to be the relaxed ^{1}CT and ^{3}CT state, respectively. As expected, removing **B7** from our test set gave an excellent linear relationship (correlation coefficient R^2 = 0.94) and an excellent MAD of 0.05 eV. So, overall, our

protocol can successfully predict the gap in unknown molecules, except in cases in which the transitions are composed of heavily mixing of MOs, and assigning the nature of the transition becomes problematic.

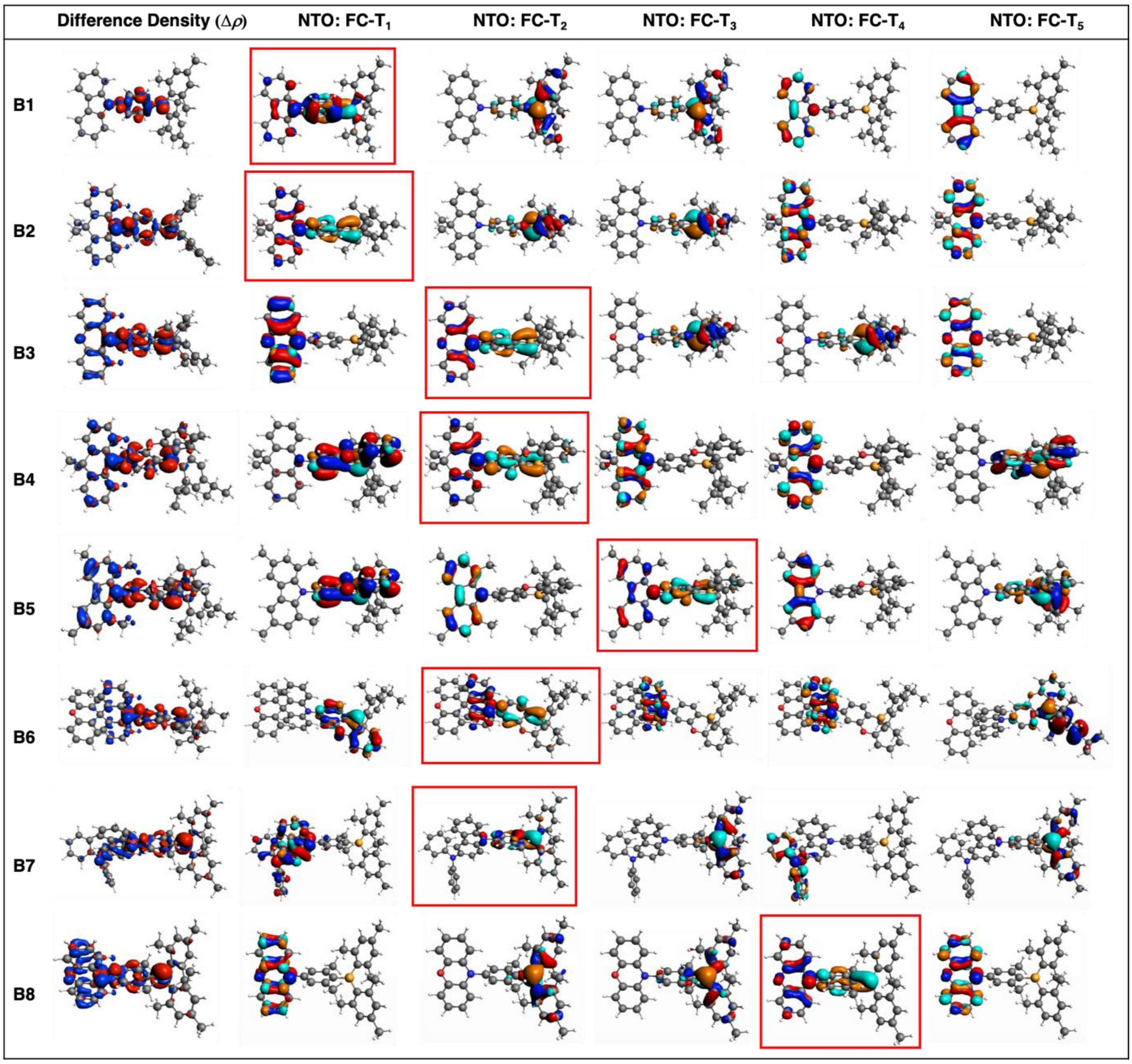

Figure S7. Mapping difference density profiles ($\Delta\rho$) plotted at isovalue = ±0.002 au (hole density in blue and the electron density in red) with the NTO of the corresponding FC-T_n state plotted at isovalue = ±0.05 au for all the benchmark systems.

Due to the approximations involved, $\Delta E(S_1)$ exhibits a larger variation in errors with MD and MAD of 0.10 eV and 0.15 eV, respectively. The small deviation is a result of the cancellation of errors emerging mostly from a large overestimation for **B7** (0.47 eV) and consistent small underestimation for **B4**, **B5** and **B6** (≈ –0.12 eV). Poor performance of our protocol to estimate the fluorescence energy of **B7** is again due to ambiguous assignment of the FC-S_2 as the relaxed S_1 state, details of which are mentioned above. Therefore, our approximation of equating $\lambda_r(T_1)$ and $\lambda_r(S_1)$ is very poor in this case,

which is reflected in a large error in predicting the fluorescence energy maximum. Interestingly, **B2** and **B3** show a very small deviation which might be due to cancellation of errors. For example, if $\lambda_r(T_1)$ is larger than the true $\lambda_r(S_1)$, a cancellation of red-shift, and blue-shift originating from the emission energy resembling more $\Delta E^{0\text{-}0}(S_1)$, can occur which might, fortunately, lead to better estimation of the fluorescence energy maximum. On the other hand, if $\lambda_r(T_1)$ is smaller than the true $\lambda_r(S_1)$, then the blue-shift adds up and might lead to more error-prone results as for **B1** or **B7**. But, fortunately, barring **B7**, the errors are quite small with MAD of 0.10 eV, which means our approximations work very well for the rest of the molecules. However, the correlation between $\Delta E(S_1)$ and the experimental emission energy is very poor (R^2 = 0.53) but, when **B7** is removed from our test set, the correlation increases to R^2 = 0.81 (see Figure S4). This stems from irregular estimations of the emission energy. As a result, our methodology for predicting the fluorescence energy maximum should be used carefully for benchmarking purposes, but it can definitely provide a good prediction of the color of the prompt or the delayed emission.

Statistically speaking, all of the computed $\Delta E_{\mathrm{VE}}(T_1)$ are underestimated, leading to an MD and an MAD of 0.22 eV (Table S4). The underestimation might be the result of using the optimal γ value calculated from ground state geometry to optimize the T_1 state. However, an excellent correlation of 0.96 (Figure S5) suggests that the error is systematic and, hence, can be corrected. Correcting the raw $\Delta E_{\mathrm{VE}}(T_1)$ by the MAD, reduces the resulting unsigned error from 0.220 eV to 0.003 eV. Overall, our methodology can predict the phosphorescence emission energy maximum of an unknown model system belonging to a similar class of benchmark systems with an unsigned error of 0.22 eV.

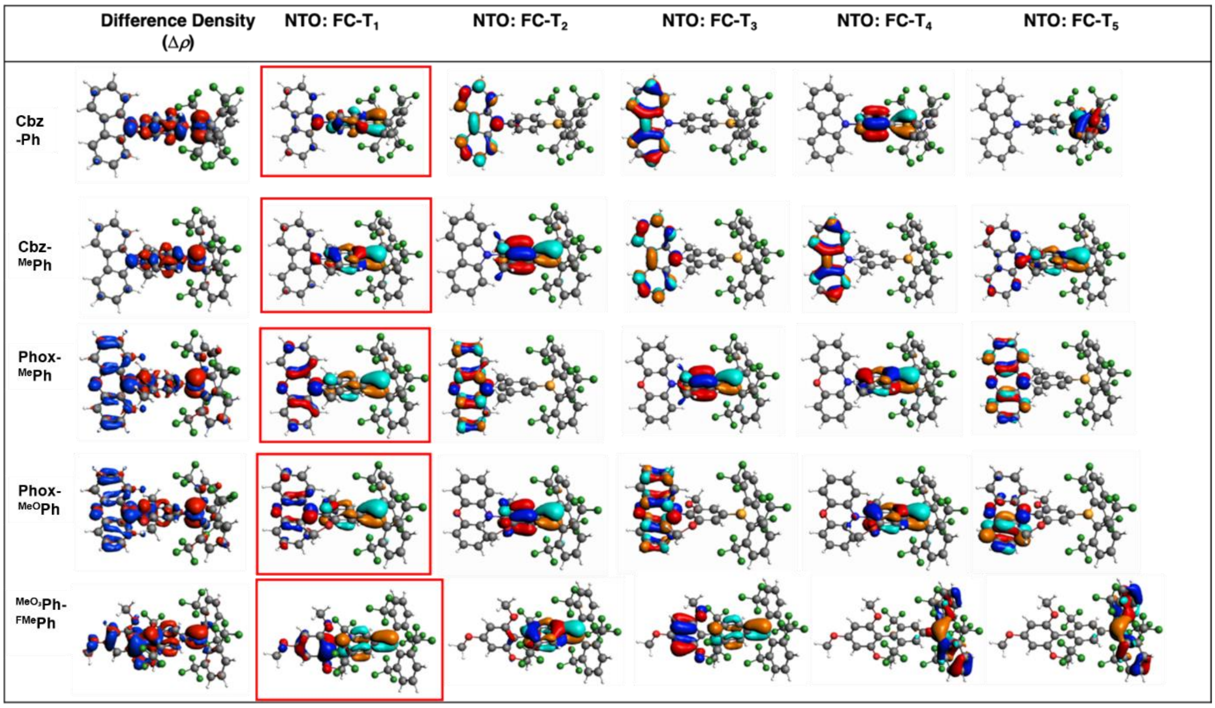

Figure S8. Mapping difference density profiles ($\Delta\rho$) plotted at isovalue = ±0.002 au (hole density in blue and the electron density in red) with the NTO of the corresponding FC-T_n state plotted at isovalue = ±0.05 au for the synthesized model systems.

Synthesis and characterization

General experimental details

Unless otherwise noted, the following conditions apply.

All syntheses were carried out using standard Schlenk and glovebox techniques under an argon atmosphere. The solvents were dried using a solvent purification system (SPS) from Innovative Technology Inc. and were degassed and stored over molecular sieves under argon. Deuterated solvents (CD_2Cl_2, $CDCl_3$, C_6D_6, acetone-d_6, and DMSO-d_6) used for NMR spectroscopy were purchased from Cambridge Isotope Laboratories. C_6D_6 was dried over molecular sieves and stored under an argon atmosphere before use. Trimethyl borate was purchased from Sigma Aldrich and distilled before use. Boron trifluoride diethyl etherate was purchased from Merk Millipore. *n*-Butyllithium (2.5 M solution in hexane) was purchased from Acros Organics and used as received. B_2pin_2 was kindly provided by AllylChem Co. Ltd. (Dalian, China). Bis-1,3-trifluoromethylbenzene was purchased from ABCR, distilled and degassed before use. $[Ir(COD)(OMe)]_2$ was prepared according to a literature procedure.[28] The compounds 9-(4-bromophenyl)-9*H*-carbazole **(i)**[29] 2-(3,5-dimethoxyphenyl)-4,4,5,5-tetramethyl-1,3,2-dioxa-borolane,[30] and potassium(3,5-dimethoxyphenyl)trifluoroborate[31] were synthesized according to literature procedures. All other starting materials were purchased from commercial sources

and were used without further purification.

Column chromatography

Column chromatography was performed on silica gel 60 (40-63 μm) (purchased from VWR), or basic alumina 90 active (purchased from Merck), and automated flash chromatography was performed on silica gel (Biotage SNAP catridge KP-Sil 10 g or KP-Sil 100 g), obtained from Biotage, using a Biotage® Isolera Four Flash system. Solvents were generally removed using a rotary evaporator *in vacuo* at a maximum temperature of 55 °C.

NMR spectra

NMR spectra were recorded on a Bruker Avance 200 (operating at ^{1}H: 199.9 MHz, ^{11}B{^{1}H}: 64.1 MHz, ^{19}F{^{1}H}: 188.1 MHz) or a Bruker Avance 500 FT NMR spectrometer (operating at ^{1}H: 500 MHz, ^{11}B{^{1}H}: 160 MHz, ^{13}C{^{1}H}: 126 MHz, ^{19}F{^{1}H}: 470.6 MHz,^{29}Si{^{1}H}: 99.3 MHz). Chemical shifts (δ) are given in ppm and are referenced to external BF_3·Et_2O (^{11}B{^{1}H}) and $CFCl_3$ (^{19}F{^{1}H}). ^{1}H NMR spectra were referenced via residual proton resonances of $CDCl_3$ (7.26 ppm), CD_2Cl_2 (5.32 ppm), acetone-d_6 (2.05 ppm) C_6D_6 (7.16 ppm).[32] ^{13}C{^{1}H} spectra were referenced to $CDCl_3$ (77.16 ppm), CD_2Cl_2(53.84 ppm), acetone-d_6 (29.84 ppm) C_6D_6 (128.06 ppm).[33] GCMS analyses were performed on an Agilent Technologies GCMS system (GC 7890A, EI-MS 5975C). HRMS were recorded using a Thermo Scientific Exactive Plus Orbitrap MS system with either an Atmospheric Sample Analysis Probe (ASAP) or by Electro-Spray Ionization (ESI).

Crystal structure determination

Crystals suitable for single-crystal X-ray diffraction were selected, coated in perfluoropolyether oil, and mounted on MiTeGen sample holders. Diffraction data were collected on Bruker X8 Apex II 4-circle diffractometers with CCD area detectors using MoK_α radiation monochromated by graphite (**2, FB(FXyl)$_2$, iv', iv, 5**) or multi-layer focusing mirrors (**3, 4**), or on a Bruker D8 Quest diffractometer with a CMOS area detector and multi-layer mirror monochromated MoK_α radiation (**iii**). The crystals were cooled using an Oxford Cryostreams or Bruker Kryostream low-temperature device. Data were collected at 100 K. The images were processed and corrected for Lorentz-polarization effects and absorption as implemented in the Bruker software packages. The structures were solved using the intrinsic phasing method (SHELXT)[34] and Fourier expansion technique. All non-hydrogen atoms were refined in anisotropic approximation, with hydrogen atoms 'riding' in idealized positions, by full-matrix least squares against F^2 of all data, using SHELXL[35] software and the SHELXLE graphical user interface.[36] Diamond[37] software was used for graphical representation. Other structural information

was extracted using Mercury[38] and OLEX2[34] software. Crystal data and experimental details are listed in Table S6; full structural information has been deposited with Cambridge Crystallographic Data Centre. CCDC-1949269 (**1**), 1949270 (**2**), 1949271 (**3**), 1949272 (**4**), 1949273 (**5**), 1949275 (**iii**), 1949277 (**iv'**), 1949276 (**iv**) and 1949274 (**FB(FXyl)$_2$**)

Photophysical measurements

All measurements were performed in standard quartz cuvettes (1 cm x 1 cm cross-section). UV–visible absorption spectra were recorded using an Agilent 8453 diode array UV-visible spectrophotometer.
The extinction coefficients of **1 - 5** were calculated from 6 independently prepared samples in hexane.
The emission spectra were recorded using an Edinburgh Instruments FLSP920 spectrometer equipped with a double monochromator for both excitation and emission, operating in right-angle geometry mode, and all spectra were fully corrected for the spectral response of the instrument. All solutions used for photophysical measurements had a concentration lower than 2×10^{-5} M to minimize inner filter effects during fluorescence measurements.
The fluorescence quantum yields were measured using a calibrated integrating sphere (inner diameter: 150 mm) from Edinburgh Instruments combined with the FLSP920 spectrometer described above. For solution-state and solid-state measurements, the longest-wavelength absorption maximum of the compound in the respective solvent was chosen as the excitation wavelength.
Fluorescence lifetimes were recorded using the time-correlated single-photon counting (TCSPC) method using the same FLSP920 spectrometer described above. Solutions were excited with a picosecond pulsed diode laser at emission maxima of 376.6 nm (**1**, **2** and **5**), 472.6 nm (**3**) and 508.8 nm (**4**). The full width at half maximum (FWHM) of the laser pulses were ca. 70–200 ps, while the instrument response function (IRF) had a FWHM of ca. 1.0 ns, measured from the scatter of a Ludox solution at the excitation wavelength. Decays were recorded to at least 10000 counts in the peak channel with a record length of at least 1000 channels. The band pass of the monochromator was adjusted to give a signal count rate of <10 kHz. Iterative reconvolution of the IRF with one decay function and non-linear least-squares analysis were used to analyze the data. The quality of the fit was judged by the calculated value of the reduced χ^2 and visual inspection of the weighted residuals.

The time-gated emission spectrum of **2** at 77 K was recorded with a 5 ms delay after opening the shutter to the excitation source. Due to the large difference in lifetime of the prompt fluorescence and the phosphorescence (a factor of 10^8), it was possible to record the phosphorescence spectrum without the fluorescence signal. Spectra were background corrected for the dark noise and spectral sensitivity of the spectrometer.

Electrochemical measurements

Cyclic voltammetry experiments were conducted in an argon-filled glovebox using a Gamry Instruments Reference 600 potentiostat. A standard three-electrode cell configuration was employed using a platinum disk working electrode, a platinum wire counter electrode, and a silver wire reference electrode separated by a Vycor frit, serving as the reference electrode. The redox potentials are referenced to the ferrocene/ferrocenium ([Fc/Fc$^+$]) redox couple by using decamethylferrocene ([Cp*$_2$Fe]; $E_{1/2}$ = –0.532 V in CH_2Cl_2) as an internal standard. Tetra-*n*-butylammonium hexafluorophosphate ([*n*Bu_4N][PF_6]) was employed as the supporting electrolyte. Compensation for resistive losses (*iR* drop) was employed for all measurements

Synthetic procedure

2-iodo-1,3-bis(trifluoromethyl)benzene (FXylI)

The synthesis reported herein is an optimization of the conditions reported by Schlosser and co-workers.[39] The synthetic conditions were optimized in terms of batch size, yield as well as cost efficiency.

A solution of MeLi in Et_2O (1.6 M solution in Et_2O, 80 mmol, 1.0 eq.) was added dropwise to a solution of KO^{t}Bu (17.95 g, 160 mmol, 2.0 eq.) in THF (250 mL) at –78 °C. After addition, the solution was stirred at –78 °C for 30 min. Then 1,3-bis(trifluoromethyl)benzene (12.3 mL, 80.0 mmol, 1.0 eq.) was added dropwise and the dark purple reaction was stirred for 3 h at –78 °C. Then, iodine (20.3 g, 80 mmol, 1.0 eq.) was rapidly added as a solid. The reaction was stirred overnight and slowly warmed to ambient temperature. Afterwards, the reaction was diluted with 200 mL of pentane and filtered through a silica plug (pentane/silica, 5 cm). All volatiles were removed under reduced pressure to give a brown oil. The crude product was sublimed (2 x 10^{-2} mbar, 30 to 80 °C) to give 2-iodo-1,3-bis(trifluoromethyl)benzene as a colorless solid (18.7 g, 63.6 mmol, 80%).

^{1}H NMR (200 MHz, 298 K, $CDCl_3$): δ = 7.82 (d, ^3J = 8 Hz, 2H), 7.58 (t, ^3J = 8 Hz, 1H) ppm;

^{19}F NMR (188.1 MHz, 298 K, $CDCl_3$): δ = –61.9 ppm;

Elem. Anal. Calc. (%) for $C_8H_3F_6I$: C 28.26, H 0.89; found: C 28.57, H 0.87;

The data fit those previously reported.[40]

Bis(2,6-bis(trifluoromethyl)phenyl)fluoroborane (FB(FXyl)$_2$)

A solution of *n*BuLi (7.7 mL, 2.3 M in hexane, 2.20 eq.) was added dropwise to a solution of 2-iodo-1,3-bis(trifluoromethyl)benzene (5.00 g, 14.7 mmol, 2.00 eq.) in MTBE (30 mL) at –78 °C. The reaction was warmed to ambient temperature and stirred for 2 h. After cooling to –78 °C, BF_3•OEt_2 (0.89 mL, 7.35 mmol, 1.00 eq.) was added dropwise and the reaction was warmed to ambient temperature overnight. All volatiles were removed *in vacuo* and the remaining solid was extracted with hexane (4 x 40 mL). The solution was concentrated *in vacuo.* After crystallization at –30 °C and removal of the residual solvent, bis(2,6-bis(trifluoromethyl)phenyl)fluoroborane[41] was obtained as colorless crystals (1.75 g, 3.84 mmol, 52%).

^{1}H NMR (200 MHz, 298 K, $CDCl_3$): δ = 7.34 (d, ^3J = 8 Hz, 2H), 6.76 (t, ^3J = 8 Hz, 1H) ppm;

^{11}B NMR (64.1 MHz, 298 K, $CDCl_3$): δ = 47.5 ppm;

^{19}F NMR (188.1 MHz, 298 K, $CDCl_3$): δ = –56.7 (d, $J_{F\text{-}F}$ = 14 Hz) ppm;

The data fit those previously reported.[42]

9-(4-(bis(2,6-bis(trifluoromethyl)phenyl)boryl)phenyl)carbazole (1)

A solution of *n*BuLi in hexane (1.30 mL, 2.3 M, 1.20 eq.) was added dropwise to a suspension of 9-(4-bromophenyl)-9*H*-carbazole (950 mg, 2.95 mmol, 1.20 eq.) in hexane (60 mL) at –78 °C. The reaction was slowly warmed to ambient temperature and stirred overnight. The reaction was cooled to –78 °C and a solution of bis-(2,6-bis(trifluoromethyl)phenyl)-fluoroborane (1.12 g, 2.46 mmol, 1.00 eq.) in Et_2O (40 mL) was added dropwise. The reaction was slowly warmed to ambient temperature and stirred overnight. All volatiles were removed *in vacuo*. The product was then purified by column chromatography (hexane/CH_2Cl_2 (2:1)) and recrystallized from hexane to give 9-(4-(bis(2,6-bis(trifluoromethyl)phenyl)boryl)phenyl)-9H-carbazole as light blue crystals (1.04 g, 1.99 mmol, 80 %).

^{1}H NMR (500 MHz, 298 K, $CDCl_3$): δ = 8.02 (m, 2H), 7.51 (d, ^3J = 8 Hz, 4H), 7.47 – 7.49 (m, 2H), 7.20 – 7.29 (m, 8H), 6.85 (t, ^3J = 8 Hz, 2H) ppm;

^{13}C{^{1}H} NMR (126 MHz, 298 K, $CDCl_3$): δ = 145.0 (C_q, 1C), 142.2 (C_q, 1C), 140.8 (C_q, 2C), 139.2 (C_q, 2C), 138.5 (CH, 2C), 135.9 (C_q, 4C), 130.8 (CH, 2C), 130.2 (CH, 4C), 126.6 (CH, 2C), 125.5 (CH, 2C), 124.6 (C_q, 4C, q, $^1J_{CF}$ = 274 Hz), 124.4 (C_q, 2C), 120.9 (CH, 2C), 120.7 (CH, 2C), 110.2 (CH, 2C) ppm;

^{11}B{^{1}H} NMR (160 MHz, 298 K, $CDCl_3$): δ = 72.9 ppm;

^{19}F{^{1}H} NMR (470 MHz, $CDCl_3$): δ = –52.8 ppm;

Elem. Anal. Calc. (%) for $C_{34}H_{18}BF_{12}N$: C 60.12, H 2.67, N 1.59; found: C 60.67, H 2.65, N 2.21;

HRMS (APCI, pos): Calc.: 680.1413 [m/z]; found: m/z = 680.1403 [MH^+].

9-(4-bromo-2,6-dimethylphenyl)carbazole (ii)

Carbazole (1.01 g, 6 mmol) and Cs_2CO_3 (10.5 g, 30 mmol) were suspended in 30 mL of dry and degassed DMF. The suspension was stirred for 15 min at ambient temperature before 5-bromo-2-fluoro-1,3-dimethylbenzene (2.43 g, 12 mmol) was added. The reaction was heated to reflux overnight. Then water was added and the reaction was extracted with ethyl acetate (3 x 70 mL). The organic phase was dried of $MgSO_4$ and all volatiles were removed *in vacuo*. The resulting light yellow solid was recrystallized from hexane to give 9-(4-bromo-2,6-dimethylphenyl)-9H-carbazole as an off-white solid in 70% yield (1.49 g, 4.26 mmol)

^{1}H NMR (500 MHz, 298 K, $CDCl_3$): δ = 7.99 (m, 2H), 7.21 (m, 2H), 7.11 (m, 2H), 6.75 (m, 2H), 1.66 (m, 2H) ppm;

^{13}C{^{1}H} NMR (126 MHz, 298 K, $CDCl_3$): δ = 140.5 (C_q, 2C), 140.2 (C_q, 2C), 133.9 (C_q, 1C), 131.7 (CH, 2C), 126.3 (CH, 2C), 123.2 (C_q, 2C), 122.6 (C_q, 1C), 120.7 (CH, 2C), 119.8 (CH, 2C), 109.4 (CH, 2C), 17.5 (CH_3, 2C) ppm;

HRMS (APCI, neg): Calc.: 350.0373 [m/z]; found: m/z = 350.0379 [M^-].

9-(4-(bis(2,6-bis(trifluoromethyl)phenyl)boryl)-2,6-dimethylphenyl)carbazole (2)

A solution of 9-(4-bromo-2,6-dimethylphenyl)-9H-carbazole (350 mg, 1 mmol) in 20 mL of hexane was cooled to – 78 °C and *n*BuLi (1.6 M, 1.1 mmol, 0.69 mL) was added. The reaction was warmed to ambient temperature and stirred overnight. Then the reaction was cooled to – 78 °C again and a solution of bis(2,6-bis(trifluoromethyl)phenyl)fluoroborane (456 mg, 1 mmol) in 20 mL of hexane was added. The reaction was warmed to ambient temperature and stirred for 2 d. Then, 2 mL of methanol were added, all volatiles removed *in vacuo*, and the residue was purified by column chromatography (hexane/CH_2Cl_2, silica) to give 9-(4-(bis(2,6-bis(trifluoromethyl)phenyl)boryl)-2,6-dimethylphenyl)-9H-carbazole as a light green solid (170 mg, 0.24 mmol, 24%).

^{1}H NMR (500 MHz, $CDCl_3$): δ = 8.20 – 8.17 (m, 2H), 8.01 (d, 4H, ^3J = 8 Hz), 7.79(t, 2H, ^3J = 8 Hz), 7.42 – 7.38 (m, 2H), 7.30 – 7.26 (m, 2H), 7.08 (s, 2H), 6.88 (dt, 2H, J_1 = 8 Hz, J_2 = 1 Hz), 1.78 (s, 6H) ppm.

^{13}C{^{1}H} NMR (126 MHz, $CDCl_3$): δ = 140.1 (C_q, 2C), 139.0 (C_q, 1C), 138.6 (CH, 1C), 137.1 (C_q, 1C), 136.8 (CH, 2C), 130.7 (CH, 2C), 130.2 (CH, 4C), 126.3 (CH, 2C), 124.7 (C_q, 4C, q, $^1J_{CF}$ = 275 Hz,), 123.1 (C_q, 1C), 120.6 (CH, 2C), 119.7 (CH, 2C), 109.5 (CH, 2C), 17.5 (CH_3, 2C) ppm.

^{11}B{^{1}H} NMR (160 MHz, 298 K, $CDCl_3$): δ = 70.6 ppm.

^{19}F{^{1}H} NMR (470 MHz, 298 K, $CDCl_3$): δ = –53.40 ppm.

Elem. Anal. Calc. (%) for $C_{36}H_{22}BF_{12}N$: C 61.13, H 3.13, N 1.64; found: C 61.38, H 2.97, N 1.64

HRMS (ASAP, pos): Calc.: 708.1726 [m/z]; found: m/z = 708.1711 [MH^+].

2-(4-iodo-3,5-dimethylphenyl)-4,4,5,5-tetramethyl-1,3,2-dioxaborolane

A solution of B_2pin_2 (4.03 g, 15.9 mmol), $[Ir(COD)OMe]_2$ (0.21 g, 0.32 mmol) (COD = 1,5-cyclooctadiene) and 4,4'-di-*tert*butyl-2,2'-bipyridine (dtbpy) (0.18 g, 0.64 mmol) in hexane (30 mL) was stirred for 10 min at r.t. Then, 2-iodo-1,3-dimethylbenzene (2.46 g, 10.60 mmol) was added and the reaction was stirred for 3 d at 60 °C. The reaction was filtered through a silica plug and washed with hexane (200 mL) and the product eluted with a CH_2Cl_2:hexane mixture (200 mL, 1:9). All volatiles were removed *in vacuo*, to give 2-(4-iodo-3,5-dimethylphenyl)-4,4,5,5-tetramethyl-1,3,2-dioxaborolane as a white solid (3.38 g, 9.43 mmol, 89%).

^{1}H NMR (500 Hz, 298 K, $CDCl_3$): δ = 7.46 – 7.44 (m, 2H), 2.49 – 2.45 (m 6H,), 1.34 (s, 12H) ppm;

^{11}B{^{1}H} NMR (160 Hz, 298 K, $CDCl_3$): δ = 31.2 ppm;

^{13}C{^{1}H} NMR (126 Hz, 298 K, $CDCl_3$): δ = 141.6 (C_q, 2C), 132.9 (CH, 2C), 113.0 (C_q, 1C), 83.9 (C_q, 2C), 29.5 (CH_3, 2C), 24.9 (CH_3, 4C) ppm;

HRMS (ASAP pos): calc.: 359.0674 [m/z]; found: m/z = 359.0668 [MH^+].

Potassium(4-iodo-3,5-dimethylphenyl)trifluoroborate

To a solution of 2-(4-iodo-3,5-dimethylphenyl)-4,4,5,5-tetramethyl-1,3,2-dioxaborolane (3.70 g, 10.3 mmol) in THF (30 mL), a saturated solution of KHF_2 (3.63 g, 46.5 mmol, 4.5 eq.) in water was added. The reaction was stirred over night at ambient temperature. All volatiles were removed *in vacuo* and the remaining solid was extracted with acetone (3 x 50 mL). The solvent was removed *in vacuo* and the crude product was recrystallized from ethanol to give potassium(4-iodo-3,5-dimethylphenyl)trifluoroborate as a white solid (3.14 g, 9.29 mmol, 90%).

^{1}H NMR (500 Hz, 298 K, acetone-d_6): δ = 7.20 (s, 2H), 2.36 – 2.35 (m, 6H) ppm;

^{11}B{^{1}H} NMR (160 Hz, 298 K, acetone-d_6): δ = 3.3 (q, $^{1}J_{BF}$ = 51 Hz) ppm;

^{19}F{^{1}H} NMR (470.6 Hz, 298 K, acetone-d_6): δ = –142.82 ppm;

^{13}C{^{1}H} NMR (126 Hz, 298 K, acetone-d_6): δ = 138.6 (C_q, 2C,), 131.3 (CH, 2C, q, $^{3}J_{CF}$ = 2 Hz), 104.2 (C_q, 1C), 28.9 (CH_3, 2C) ppm;

HRMS (ESI neg): calc.: 298.9710 [m/z]; found: m/z = 298.9723 [M^-].

Bis(2,6-bis(trifluoromethyl)phenyl)(4-iodo-3,5-dimethylphenyl)borane (iii)

To a solution of 2-iodo-1,3-bis(trifluoromethyl)benzene (3.37 g, 9.89 mmol) in MTBE (40 mL) a solution of *n*BuLi (1.6 M in hexane, 8.27 mL, 13.2 mmol) was slowly added at –78 °C and the mixture was stirred for 30 min. The reaction was warmed to ambient temperature. and stirred for 2 h. The reaction mixture was added to a suspension of potassium(4-iodo-3,5-dimethylphenyl)trifluoroborate (1.52 g, 4.50 mmol) in THF (20 mL) at –78 °C and then stirred overnight. The reaction was quenched with MeOH (1 mL) and all volatiles were removed *in vacuo*. The residue was dissolved in hexane and filtered through a plug of silica (silica/hexane). The solvent was evaporated to give bis(2,6-bis(trifluoromethyl)phenyl)(4-iodo-3,5-dimethylphenyl)borane as a light yellow solid (2.01 g, 3.02 mmol, 67% yield).

^{1}H NMR (500 Hz, 298 K, $CDCl_3$): δ = 7.94 (d, 4H, ^{3}J = 8 Hz), 7.75 (t, 2H, ^{3}J = 8 Hz), 6.82 (s, 2H), 2.40 (s, 6H) ppm:

^{11}B{^{1}H} NMR (160 Hz, 298 K, $CDCl_3$): δ = 69.5 ppm;

^{19}F{^{1}H} NMR (470.6 Hz, 298 K, $CDCl_3$): δ = –53.15 ppm;

^{13}C{^{1}H} NMR (126 Hz, 298 K, $CDCl_3$): δ = 140.9 (C_q, 2C), 134.4 (CH, 4C), 130.5 (CH, 2C), 130.1 (CH, 2C), 123.9 (CF_3, 4C, q, $^{1}J_{CF}$ = 274 Hz), 115.8 (C_q, 2C), 29.7 (CH_3, 2C) ppm;

HRMS (ASAP neg): calc.: 668.0036 [m/z]; found: m/z = 668.0048 [M^-].

10-(4-(bis(2,6-bis(trifluoromethyl)phenyl)boryl)-2,6-dimethylphenyl) phenoxazine (3)

A suspension of bis(2,6-bis(trifluoromethyl)phenyl)(4-iodo-3,5-dimethylphenyl)borane (400 mg, 0.6 mmol), phenoxazine (148 mg, 0.8 mmol), NaO^{t}Bu (88 mg, 0.92 mmol), Pd(dba)$_3$*CHCl$_3$ (12 mg, 0.01 mmol) and Xanthphos (8 mg, 0.01 mmol) in toluene was heated to 90 °C for 18 h. Afterwards, all volatiles were removed *in vacuo* and the product mixture was purified by column chromatography (CH_2Cl_2/hexane). The product was recrystallized from hexane to give 10-(4-(bis(2,6-bis(trifluoromethyl)phenyl)boryl)-2,6-dimethylphenyl)-10H-phenoxazine as a yellow solid (201 mg, 0.27 mmol, 46%).

^{1}H NMR (500 Hz, 298 K, C_6D_6): δ = 7.49 (d, 4 H, ^3J = 8 Hz), 7.21 (s, 2 H), 6.83 (t, 2 H, ^3J = 8 Hz), 6.78 – 6.67 (m, 2 H), 6.54 – 6.38 (m, 4 H), 5.99 – 5.85 (m, 2 H), 1.98 (s, 6 H) ppm;

^{11}B{^{1}H} NMR (160 Hz, 298 K, C_6D_6): δ = 71.2 ppm;

^{19}F{^{1}H} NMR (470.6 Hz, 298 K, C_6D_6): δ = –52.75 ppm;

^{13}C{^{1}H} NMR (126 Hz, 298 K, C_6D_6): δ = 147.1 ($C_{q,}$ 1C), 144.4 ($C_{q,}$ 2C), 139.8 ($C_{q,}$ 2C), 139.2 ($C_{q,}$ 2C), 138.5 ($C_{q,}$ 2C), 138.3 (CH, 4C), 132.3 (C_q, 2C), 130.9 (CH, 2C), 130.2 (CH, 4C), 124.6 (C_q, 4C, q, $^1J_{CF}$ = 273 Hz), 124.3 (CH, 2C), 121.9 (CH, 2C), 116.1 (CH, 2C), 111.8 (CH, 2C), 17.5 (CH_3, 2C) ppm;

HRMS (APCI neg): calc.: 723.1597 [m/z]; found: m/z = 723.1617 [M^-];

Elem. Anal. Calc. (%) for: C 59.78, H 3.07, N 1.94; found: C 60.12, H 3.04, N 2.12.

Bis(2,6-bis(trifluoromethyl)phenyl)(3,5-dimethoxyphenyl)borane (iv')

To a solution of 2-iodo-1,3-bis(trifluoromethyl)benzene (1.36 g, 4.0 mmol) in MTBE (20 mL) a solution of *n*BuLi (1.6 M in hexane, 2.75 mL, 4.4 mmol) was added at –78 °C and the mixture was stirred for 30 min. The reaction was warmed to ambient temperature and stirred for 2 h. The reaction was cooled to –78 °C again and potassium(3,5-dimethoxyphenyl)trifluoroborate (488 mg, 2.0 mmol) was added as a solid. The suspension was slowly warmed to ambient temperature and stirred overnight. All volatiles were removed *in vacuo* and the resulting solid extracted with hexane (3 x 80 mL). The solvent was evaporated and the crude product purified by column chromatography (pentane/CH_2Cl_2) to give bis(2,6-bis(trifluoromethyl)phenyl)(3,5-dimethoxyphenyl)borane as a light green solid (350 mg, 0.61 mmol, 30 %)

^{1}H NMR (500 Hz, 298 K, $CDCl_3$): δ = 7.48 (d, 4H, ^3J = 8 Hz), 7.98 (t, 2H, ^3J = 8 Hz), 6.67 (d, 2H, ^{4}J = 2.3 Hz), 6.65 (t, 1H, ^4J = 2 Hz), 3.20 (s, 6H) ppm;

^{11}B{^{1}H} NMR (160 Hz, 298 K, $CDCl_3$): δ = 70.4 ppm;

^{19}F{^{1}H} NMR (470.6 Hz, 298 K, $CDCl_3$): δ = –52.96 ppm;

^{13}C{^{1}H} NMR (126 Hz, 298 K, $CDCl_3$): δ = 160.6 (C_q, 2C), 148.4 (C_q, 1C), 139.1 (C_q, 2C), 136.1 (C_q, 4C), 130.7 (CH, 2C), 130.1 (CH, 4C), 124.7 (C_q, 4C, q, $^1J_{CF}$ = 280 Hz) 115.2 (CH, 2C), 105.7 (CH, 1C), 54.7 (CH_3, 2C) ppm;

HRMS (APCI neg): calc.: 574.0979 [m/z]; found: m/z = 574.0979 [M^-];

Elem. Anal. Calc. (%) for $C_{24}H_{15}BF_{12}O_2$: C 50.21, H 2.63; found: C 50.53, H 2.67.

Bis(2,6-bis(trifluoromethyl)phenyl)(4-iodo-3,5-dimethoxyphenyl)borane (iv)

To a solution of bis(2,6-bis(trifluoromethyl)phenyl)(3,5-dimethoxyphenyl)borane (350 mg, 0.61 mmol) and *N*-iodosuccinimide (150 mg, 0.67 mmol) in CH_2Cl_2 (20 mL), $FeCl_3$ (10 mg, 0.06 mmol) was added. The reaction was heated to 40 °C for 18 h. Additional *N*-iodosuccinimide (60 mg, 0.27 mmol) was added and the reaction was stirred at ambient temperature for 24 h. The reaction was quenched with 10 mL of a saturated $Na_2S_2O_{3(aq)}$ solution and extracted with Et_2O (3 x 50 mL). The organic phases were dried over sodium sulfate and all volatiles were removed *in vacuo* to give the crude product. Pure bis(2,6-bis(trifluoromethyl)phenyl)(4-iodo-3,5-dimethoxyphenyl)borane was obtained by recrystallization from hexane (360 mg, 0.51 mmol, 84%).

^{1}H NMR (500 Hz, 298 K, $CDCl_3$): δ = 7.47 (d, 4H, ^{3}J = 8 Hz), 6.83 (t, 2H, ^{3}J = 8 Hz), 6.43 (s, 2H), 3.17 (s, 6H) ppm;

^{11}B{^{1}H} NMR (160 Hz, 298 K, $CDCl_3$): δ = 70.0 ppm;

^{19}F{^{1}H} NMR (470.6 Hz, 298 K, $CDCl_3$): δ = –52.92 ppm;

^{13}C{^{1}H} NMR (126 Hz, 298 K, $CDCl_3$): δ = 159.3 (C_q, 2C), 147.3 (C_q, 1C), 138.4 (C_q, 2C), 136.1 (C_q, 4C), 130.5 (CH, 2C), 129.9 (CH, 4C), 124.2 (C_q, 4C, q, $^{1}J_{CF}$ = 276 Hz) 111.7 (CH, 2C), 86.9 (CH, 1C), 55.4 (CH_3, 2C) ppm;

HRMS (APCI neg): calc.: 699.9945 [m/z]; found: m/z = 699.9952 [M^-]

10-(4-(bis(2,6-bis(trifluoromethyl)phenyl)boryl)-2,6-dimethoxyphenyl)phenoxazine (4)

A suspension of bis(2,6-bis(trifluoromethyl)phenyl)(4-iodo-3,5-dimethoxyphenyl)borane (360 mg, 0.5 mmol), phenoxazine (122 mg, 0.67 mmol), NaO^{t}Bu (73 mg, 0.76 mmol), Pd(dba)$_3$*CHCl$_3$ (12 mg, 0.01 mmol) and Xanthphos (8 mg, 0.01 mmol) in toluene was heated to 90 °C for 3 d. Afterwards, all volatiles were removed *in vacuo* and the crude product was purified by column chromatography (CH_2Cl_2/pentane). The product was recrystallized from hexane to give 10-(4-(bis(2,6-bis(trifluoromethyl)phenyl)boryl)-2,6-dimethoxyphenyl)-10H-phenoxazine as a red solid (220 mg, 0.29 mmol, 58%).

^{1}H NMR (500 Hz, 298 K, $CDCl_3$): δ = 7.50 (d, 4H, ^3J = 8 Hz), 6.84 (t, 2H, ^3J = 8 Hz), 6.72 (dd, 2 H, J_1 = 1 Hz, J_2 = 8 Hz), 6.58 (td, 2 H, J_1 = 2 Hz, J_2 = 8 Hz), 6.46 (td, 2 H, J_1 = 2 Hz, J_2 = 8 Hz), 6.27 (dd, 2 H, J_1 = 1 Hz, J_2 = 8 Hz), 3.03(s, 6H) ppm;

^{11}B{^{1}H} NMR (160 Hz, 298 K, $CDCl_3$): δ = 71.0 ppm;

^{19}F{^{1}H} NMR (470.6 Hz, 298 K, $CDCl_3$): δ = –52.81 ppm;

^{13}C{^{1}H} NMR (126 Hz, 298 K, $CDCl_3$): δ = 158.3 (C_q, 2C), 148.0 (C_q, 1C), 145.0 (C_q, 2C), 138.9 (C_q, 2C), 136.0 (C_q, 4C), 133.6 (CH, 2C), 131.0 (CH, 4C), 124.7 (C_q, 4C, q, $^1J_{CF}$ = 280 Hz), 130.3 (CH, 2C), 123.9 (CH, 2C), 121.6 (C_q, 2C), 116.0 (CH, 2C), 119.6 (CH, 1C), 113.7 (CH, 2C), 112.6 (CH, 2C), 55.3 (CH, 2C) ppm;

HRMS (APCI pos): calc.: 755.1496 [m/z]; found: m/z = 755.1478 [MH^+];

Elem. Anal. Calc. (%) for $C_{36}H_{22}BF_{12}NO_3$: C 57.24, H 2.94, N 1.85; found: C 58.03, H 2.92, N 2.24.

2,4,6-trimethoxy-2',6'-bis(trifluoromethyl)-1,1'-biphenyl

To a solution of 2-iodo-1,3-bis(trifluoromethyl)benzene (1.0 g, 2.94 mmol) in THF (10 mL), *n*BuLi (2.5 M in hexane, 1.24 mL, 3.09 mmol, 1.05 eq.) was added at –78 °C. The reaction was slowly warmed to ambient temperature over 3 h. Then, $ZnCl_2$ (0.42 g, 3.09 mmol, 1.05 eq.) was added and the reaction was stirred for 1 h at ambient temperature. Then, 2-iodo-1,3,5-trimethoxybenzene (0.86 g, 3.09 mmol, 1.0 eq.) $Pd(PPh_3)_2Cl_2$ (0.21 g, 0.29 mmol, 0.1 eq.), XanthPhos (0.17 g, 0.29 mmol, 0.1 eq) and CuBr (0.42 g, 2.94 mmol, 1.0 eq.) were added and the reaction was heated to 70 °C for 12 h. The reaction was poured into water and extracted with Et_2O (3 x 50 mL). The solvent was removed *in vacuo* and 2,4,6-trimethoxy-2',6'-bis(trifluoromethyl)-1,1'-biphenyl was purified by column chromatography (hexane/CH_2Cl_2). Yield: 240 mg (21%).

^{1}H NMR (500 Hz, 298 K, $CDCl_3$): δ = 7.93 (*d*, 2H, ^{3}J = 8 Hz), 7.55 (*t*, 1H, ^{3}J = 8 Hz), 6.17 (*s*, 2H), 3.86 (*s*, 3H), 3.66 (*s*, 6H) ppm;

^{19}F{^{1}H} NMR (470.6 Hz, 298 K, $CDCl_3$): δ = –61.89 ppm;

^{13}C{^{1}H} NMR (126 Hz, 298 K, $CDCl_3$): δ = 162.3 (C_q, 2C), 159.2 (C_q, 1C), 134.4 (C_q, m, 1C), 132.4 (C_q, 2C, q, $^{2}J_{CF}$ = 30 Hz), 129.4 (CH, 2C, q, $^{3}J_{CF}$ = 5 Hz), 127.6 (CH, 1C), 123.5 (C_q, 2C, q, $^{1}J_{CF}$ = 274 Hz), 104.9 (C_q, 1C), 90.1 (CH, 2C), 55.7 (CH_3, 2C), 55.4 (CH_3, 2C) ppm;

HRMS (ASAP pos): calc.: 382.0953 [m/z]; found: m/z = 382.0946 [MH^+].

4,4,5,5-tetramethyl-2-(2',4',6'-trimethoxy-2,6-bis(trifluoromethyl)-[1,1'-biphenyl]-4-yl)-1,3,2-dioxaborolane

A solution of B_2pin_2 (133 mg, 0.52 mmol), $[Ir(COD)OMe]_2$ (11 mg, 0.016 mmol) and dtbpy (9 mg, 32 mmol) in hexane (20 mL) was stirred for 10 min at ambient temperature. Then, 2,4,6-trimethoxy-2',6'-bis(trifluoromethyl)-1,1'-biphenyl (200 mg, 0.52 mmol) was added and the reaction was stirred over night at ambient temperature. The reaction was filtered through a silica plug and washed with hexane (100 mL) and the product was eluted with a CH_2Cl_2:pentane mixture (200 mL, 3:7). All volatiles were removed *in vacuo*, to give 2-(4-iodo-3,5-dimethylphenyl)-4,4,5,5-tetramethyl-1,3,2-dioxaborolane as a white solid (130 mg, 0.26 mmol, 49%).

^{1}H NMR (500 Hz, 298 K, $CDCl_3$): δ = 8.34 (s, 2H), 6.16 (s, 2H), 3.86 (s, 3H), 3.64 (s, 6H), 1.37 (s. 12H) ppm;

^{11}B{^{1}H} NMR (160 Hz, 298 K, $CDCl_3$): δ = 30.5 ppm;

^{19}F{^{1}H} NMR (470.6 Hz, 298 K, $CDCl_3$): δ = 61.68 ppm;

^{13}C{^{1}H} NMR (126 Hz, 298 K, $CDCl_3$): δ = 162.3 (C_q, 2C), 159.1 (C_q, 1C), 137.1 (C_q, 1C, sept., $^3J_{CF}$ = 2 Hz), 135.4 (CH, 2C, q, $^3J_{CF}$ = 6 Hz), 131.9 (C_q, 2C, q, $^2J_{CF}$ = 29 Hz), 128.9 (C_q, 1C), 123.7 (C_q, 2C, q, $^1J_{CF}$ = 275 Hz), 105.0 (C_q, 1C), 90.1 (CH, 2C), 84.7 (C_q, 2C), 55.7 (CH_3, 2C), 55.4 (CH_3, 1C), 25.0 (CH_3, 4C) ppm;

HRMS (ASAP pos): calc.: 507.1772 [m/z]; found: m/z = 507.1768 [MH^+].

Potassium(2',4',6'-trimethoxy-2,6-bis(trifluoromethyl)-[1,1'-biphenyl]-4-yl)trifluoroborate

To a solution of 4,4,5,5-tetramethyl-2-(2',4',6'-trimethoxy-2,6-bis(trifluoromethyl)-[1,1'-biphenyl]-4-yl)-1,3,2-dioxaborolane (127 mg, 0.25 mmol) in THF a saturated solution of KHF_2 (88 mg, 1,13 mmol, 4.5 eq.) in water was added. The reaction was stirred for 45 min at ambient temperature. All volatiles were removed *in vacuo* and the remaining solid extracted with acetone (3 x 15 mL). The solvent was removed *in vacuo* and the crude product was washed with pentane (2 x 30 mL) to give potassium(2',4',6'-trimethoxy-2,6-bis(trifluoromethyl)-[1,1'-biphenyl]-4-yl)trifluoroborate as a white solid (121 mg, 0,25 mmol, 99%).

^{1}H NMR (500 Hz, 298 K, acetone-d_6): δ = 8.08 (s, 2H), 6.24 (s, 2H), 3.85 (s, 3H), 3.62 (s, 6H) ppm;

^{11}B{^{1}H} NMR (160 Hz, 298 K, acetone-d_6): δ = 2.8 ppm;

^{19}F{^{1}H} NMR (470.6 Hz, 298 K, acetone-d_6): δ = –61.10, –144.06 ppm;

^{13}C{^{1}H} NMR (126 Hz, 298 K, acetone-d_6): δ = 162.9 (C_q, 2C), 160.2 (C_q, 1C), 133.1 (CH, 2C), 131.1 (C_q, 2C), 130.3 (C_q, 2C, q, $^{2}J_{CF}$ = 28 Hz), 125.6 (C_q, 2C, q, $^{1}J_{CF}$ = 274 Hz), 106.7 (C_q, 1C), 90.7 (CH, 2C), 55.7 (CH_3, 2C), 55.5 (CH_3, 1C) ppm;

HRMS (ESI neg): calc.: 447.0820 [m/z]; found: m/z = 447.0814 [M^+].

Bis(2,6-bis(trifluoromethyl)phenyl)(2',4',6'-trimethoxy-2,6-bis(trifluoromethyl)-[1,1'-biphenyl]-4-yl)borane

To a solution of 2-iodo-1,3-bis(trifluoromethyl)benzene (210 mg, 0.61 mmol) in Et_2O (10 mL) a solution of *n*BuLi (2.5 M in hexane, 0.27 mL, 0.68 mmol) was added at –78 °C and the mixture was stirred for 30 min. The reaction was then warmed to ambient temperature and stirred for 2 h. The reaction was then cooled to –78 °C and potassium(2',4',6'-trimethoxy-2,6-bis(trifluoromethyl)-[1,1'-biphenyl]-4-yl)trifluoroborate (100 mg, 0.21 mmol) was added as a solid. The suspension was slowly warmed to ambient temperature and stirred for 2 d. All volatiles were removed *in vacuo* and the resulting solid was extracted with hexane (3 x 20 mL). The solvent was evaporated and the crude product was purified by column chromatography (hexane/CH_2Cl_2) to give bis(2,6-bis(trifluoromethyl)phenyl)(2',4',6'-trimethoxy-2,6-bis(trifluoromethyl)-[1,1'-biphenyl]-4-yl)borane as a light blue solid (25 mg, 0.03 mmol, 15%)

^{1}H NMR (500 Hz, 298 K, C_6D_6): δ = 8.17 (s, 2H), 7.39 (d, 4H, ^3J = 8 Hz), 6.72 (t, 2H, ^3J = 8 Hz), 6.09 (s, 2H), 3.31 (s, 3H), 3.26 (s, 6H) ppm;

^{11}B{^{1}H} NMR (160 Hz, 298 K, C_6D_6): δ = 71.0 ppm;

^{19}F{^{1}H} NMR (470.6 Hz, 298 K, C_6D_6): δ = –52.83 (br), –61.63 (s) ppm;

^{13}C{^{1}H} NMR (126 Hz, 298 K, C_6D_6): δ = 163.2 (C_q, 2C), 159.5 (C_q, 1C), 145.2 (C_q, 2C), 140.4 (C_q, 1C), 137.6 (C_q, 1C), 136.8 (CH, 2C, q, $^3J_{CF}$ = 5 Hz), 135.8 (C_q, 4C, q, $^2J_{CF}$ = 31 Hz), 132.6 (C_q, 2C, q, $^2J_{CF}$ = 30 Hz), 131.5 (CH, 2C), 130.3 (CH, 4C, q, $^3J_{CF}$ = 5 Hz), 124.4 (C_q, 4C, q, $^1J_{CF}$ = 275 Hz), 124.4 (C_q, 2C, q, $^1J_{CF}$ = 272 Hz), 104.8 (C_q, 1C), 90.5 (CH, 2C), 55.3 (CH_3, 2C), 54.7 (CH_3, 1C) ppm;

HRMS (ASAP neg): calc.: 816.1145 [m/z]; found: m/z = 816.1153 [M^+].

Structures in the solid state

Table S6. Single-crystal X-ray diffraction data and structure refinements of **1**, **2**, **3**, **4**, **5**, **FB(FXyl)$_2$**, **iii**, **iv'** and **iv**.

Data	**1**	**2**	**3**
CCDC number	1949269	1949270	1949271
Empirical formula	$C_{34}H_{18}BF_{12}N_3$	$C_{36}H_{22}BF_{12}N$	$C_{36}H_{22}BF_{12}NO$
Formula weight / g·mol^{-1}	679.30	707.35	723.35
T / K	100(2)	100(2)	100(2)
λ / Å, radiation	MoKα 0.71073	MoKα 0.71073	MoKα 0.71073
Crystal size / mm³	0.15×0.30×0.30	0.14×0.40×0.67	0.23×0.36×0.41
Crystal color, habit	colorless block	colorless plate	yellow block
μ / mm^{-1}	0.146	0.140	0.141
Crystal system	Triclinic	Monoclinic	Monoclinic
Space group	$P\bar{1}$	$P2_1/c$	$P2_1/c$
a / Å	8.897(6)	8.903(4)	10.190(5)
b / Å	13.114(4)	7.855(3)	17.215(9)
c / Å	13.376(6)	43.996(18)	18.029(9)
α / °	104.185(18)	90	90
β / °	106.14(3)	95.273(14)	96.36(2)
γ / °	95.50(3)	90	90
Volume / Å^3	1430.7(12)	3064(2)	3143(3)
Z	2	4	4
ρ_{calc} / g·cm^{-3}	1.577	1.534	1.529
F(000)	684	1432	1464
θ range / °	1.627 – 26.876	2.297 – 28.374	1.640 – 26.119
Reflections collected	18113	47352	30980
Unique reflections	6169	7632	6243
Parameters / restraints	433 / 0	453 / 0	462 / 0
GooF on F^2	1.027	1.030	1.017
R_1 [I>2σ(I)]	0.0336	0.0400	0.0380
wR^2 (all data)	0.0817	0.1018	0.0866
Max. / min. residual electron density / e·Å^{-3}	0.327 / –0.259	0.358 / –0.334	0.261 / –0.194

-Table S6 Continued-

Data	**4**	**5**	**FB(FXyl)$_2$**
CCDC number	1949272	1949273	1949274
Empirical formula	$C_{36}H_{22}BF_{12}NO_3$	$C_{33}H_{19}BF_{18}O_3$	$C_{16}H_6BF_{13}$
Formula weight / g·mol^{-1}	755.35	816.29	456.02
T / K	100(2)	100(2)	100(2)
λ / Å, radiation	MoKα 0.71073	MoKα 0.71073	MoKα 0.71073
Crystal size / mm³	0.12×0.20×0.38	0.09×0.25×0.43	0.11×0.32×0.48
Crystal color, habit	orange plate	colorless plate	colorless plate
μ / mm^{-1}	0.147	0.176	0.207
Crystal system	Monoclinic	Orthorhombic	Monoclinic
Space group	$P2_1/c$	$Pca2_1$	$P2_1/n$
a / Å	9.372(4)	31.798(14)	12.932(4)
b / Å	17.270(7)	12.137(4)	9.272(6)
c / Å	19.717(7)	8.395(4)	15.166(7)
α / °	90	90	90
β / °	95.729(14)	90	113.26(2)
γ / °	90	90	90
Volume / Å^3	3175(2)	3240(2)	1670.7(14)
Z	4	4	4
ρ_{calc} / g·cm^{-3}	1.580	1.674	1.813
F(000)	1528	1632	896
θ range / °	1.571 – 26.874	2.111 – 26.789	2.656 – 26.021
Reflections collected	29146	40159	9349
Unique reflections	6764	6901	3288
Parameters / restraints	480 / 0	499 / 1	386 / 229
GooF on F^2	1.028	1.022	1.021
R_1 [I>2σ(I)]	0.0497	0.0350	0.0333
wR^2 (all data)	0.1243	0.0773	0.0840
Max. / min. residual electron density / e·Å^{-3}	0.480 / –0.260	0.275 / –0.228	0.218 / –0.269

-Table S6 Continued-

Data	**Iii**	**iv'**	**iv**
CCDC number	1949275	1949277	1949276
Empirical formula	$C_{24}H_{14}BF_{12}I$	$C_{24}H_{15}BF_{12}O_2$	$C_{24}H_{14}BF_{12}IO_2$
Formula weight / g·mol^{-1}	668.06	574.17	700.06
T / K	100(2)	100(2)	100(2)
λ / Å, radiation	MoKα 0.71073	MoKα 0.71073	MoKα 0.71073
Crystal size / mm³	0.52×0.55×0.58	0.19×0.29×0.42	0.20×0.37×0.37
Crystal color, habit	yellow block	colorless plate	yellow block
μ / mm^{-1}	1.471	0.170	1.418
Crystal system	Monoclinic	Monoclinic	Orthorhombic
Space group	$P2_1/c$	$P2_1/n$	*Pbca*
a / Å	7.999(5)	8.598(3)	14.928(7)
b / Å	13.770(8)	20.097(11)	9.307(3)
c / Å	21.6673(13)	13.285(7)	35.349(13)
α / °	90	90	90
β / °	100.16(2)	93.38(3)	90
γ / °	90	90	90
Volume / Å^3	2349.1(19)	2291.6(19)	4912(3)
Z	4	4	8
ρ_{calc} / g·cm^{-3}	1.889	1.664	1.893
F(000)	1296	1152	2720
θ range / °	1.760 – 30.707	1.840 – 28.321	2.642 – 30.574
Reflections collected	36626	41544	154227
Unique reflections	7292	5660	7539
Parameters / restraints	345 / 0	354 / 0	363 / 0
GooF on F^2	1.047	1.026	1.122
R_1 [I>2σ(I)]	0.0325	0.0341	0.0302
wR^2 (all data)	0.0797	0.0870	0.0753
Max. / min. residual electron density / e·Å^{-3}	1.973 / –1.581	0.330 / –0.309	0.573 / –1.079

Table S7. Selected bond lengths (Å) and angles (°) of **1 - 5**. Atomic numbering and the labels of the aryl rings R1, R2 and R3 are shown for compound **1** in Figure 2 and are used for **2 - 5** accordingly.

	**1**	**2**	**3**	**4**	**5**
B–C(R1)	1.557(2)	1.548(2)	1.553(3)	1.563(3)	1.568(4)
B–C(R2)	1.598(2)	1.603(2)	1.597(3)	1.598(3)	1.599(5)
B–C(R3)	1.606(2)	1.605(2)	1.599(3)	1.601(4)	1.601(5)
C(R1)–N/C[a)]	1.4168(18)	1.4296(17)	1.436(2)	1.421(3)	1.501(4)
C–N/C	1.4008(19)	1.3951(18)	1.401(2)	1.406(3)	1.388(4)
C–N/C	1.4055(18)	1.3937(19)	1.401(2)	1.411(3)	1.400(4)
C–C (R1,central):					
a	1.408(2)	1.4008(19)	1.400(2)	1.405(3)	1.397(4)
b	1.409(2)	1.4013(19)	1.399(2)	1.393(3)	1.394(4)
c	1.385(2)	1.3848(19)	1.389(2)	1.392(3)	1.390(4)
d	1.397(2)	1.3990(19)	1.395(2)	1.402(3)	1.403(4)
e	1.393(2)	1.4002(19)	1.389(2)	1.397(3)	1.402(4)
f	1.383(2)	1.3896(19)	1.400(2)	1.388(3)	1.393(4)
∠ NC_3-R1 (central)	43.06(7)	76.08(6)	82.05(7)	89.55(8)	88.37(12)
Shortest B–F	2.708(2)	2.845(2)	2.747(3)	2.719(3)	2.705(4)
	2.933(2)	2.793(2)	2.845(6)	2.873(4)	2.898(4)
	2.516(2)	2.725(2)	2.844(2)	2.926(3)	2.713(4)
	2.853(2)	2.852(2)	2.836(2)	2.815(3)	2.918(4)

a) The ring-connecting bond is a C–C bond in compound **5**, while it is a C–N bond in compounds **1–4**.

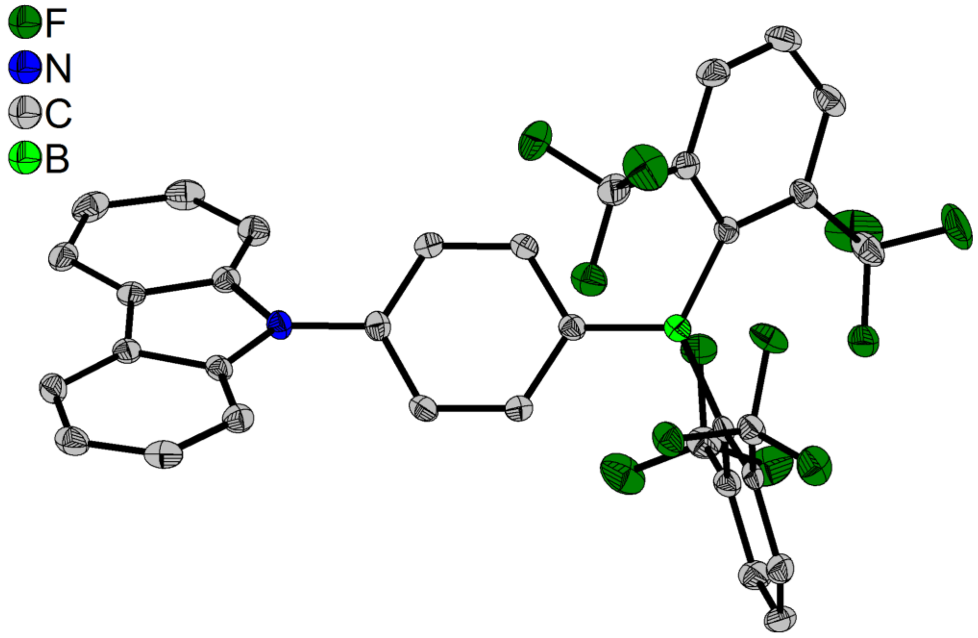

Figure S9. The solid-state molecular structure of **1** determined by single-crystal X-ray diffraction at 100 K. All ellipsoids are drawn at the 50% probability level, and H atoms are omitted for clarity.

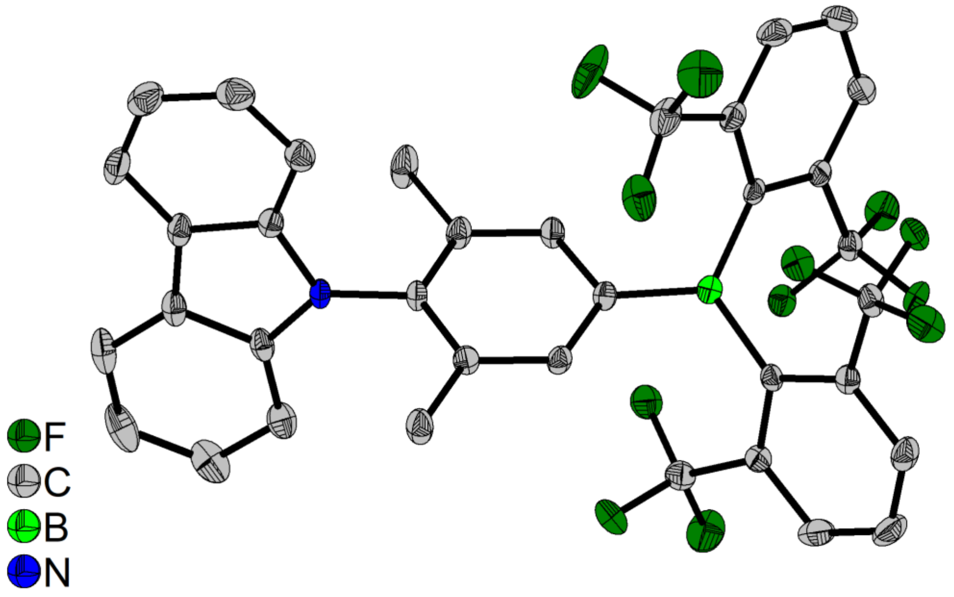

Figure S10. The solid-state molecular structure of **2** determined by single-crystal X-ray diffraction at 100 K. All ellipsoids are drawn at the 50% probability level, and H atoms are omitted for clarity.

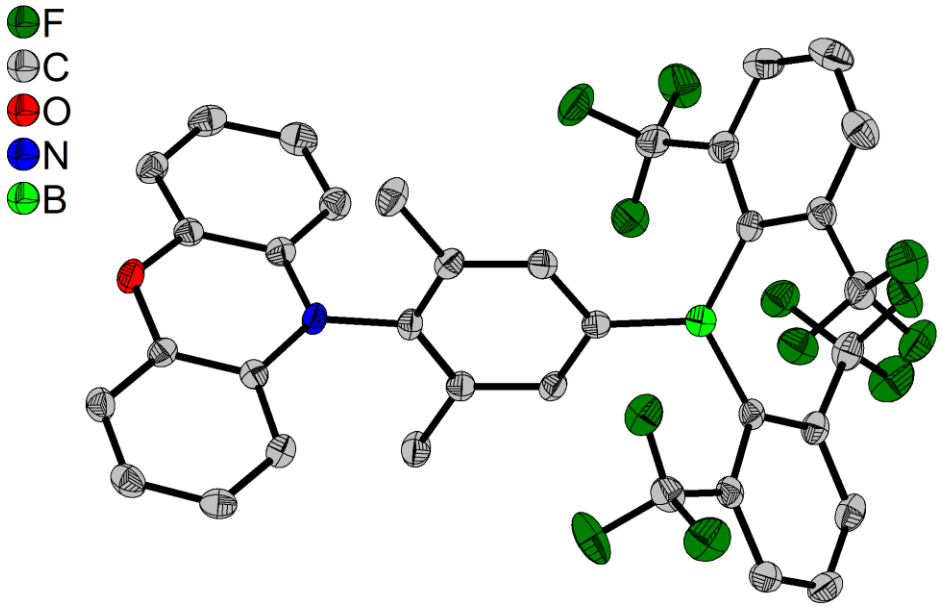

Figure S11. The solid-state molecular structure of **3** determined by single-crystal X-ray diffraction at 100 K. All ellipsoids are drawn at the 50% probability level, and H atoms are omitted for clarity.

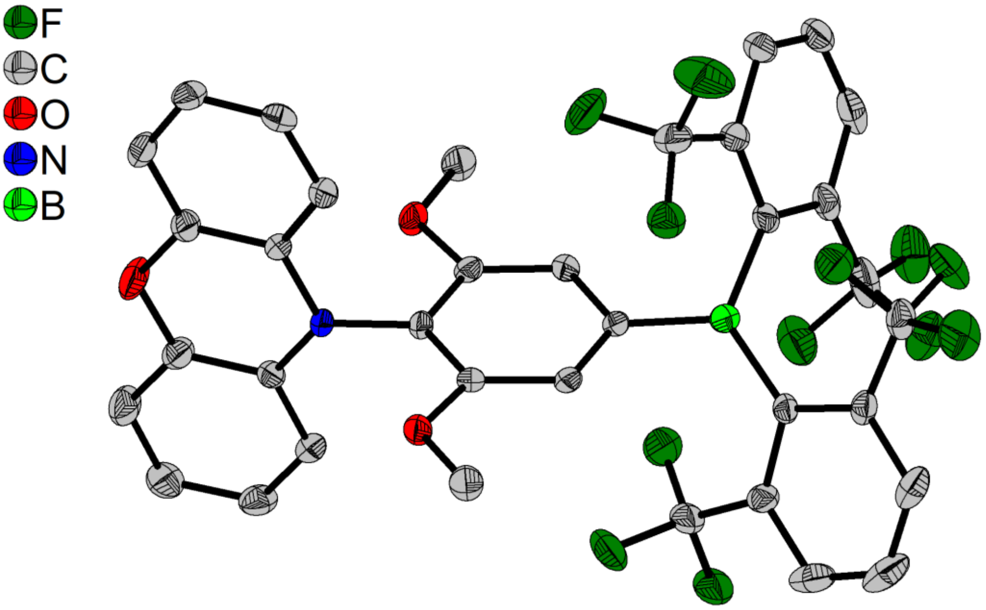

Figure S12. The solid-state molecular structure of **4** determined by single-crystal X-ray diffraction at 100 K. All ellipsoids are drawn at the 50% probability level, and H atoms are omitted for clarity.

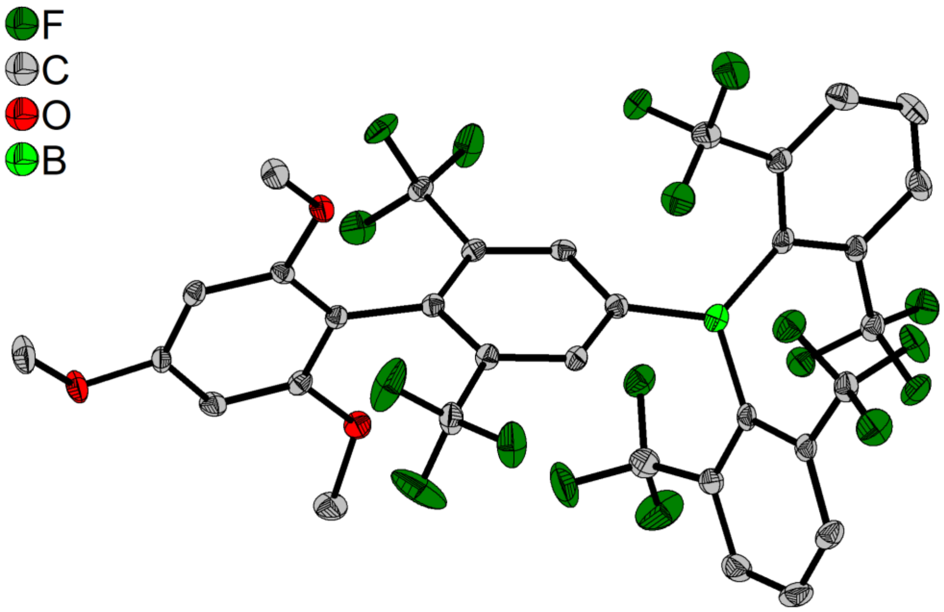

Figure S13. The solid-state molecular structure of **5** determined by single-crystal X-ray diffraction at 100 K. All ellipsoids are drawn at the 50% probability level, and H atoms are omitted for clarity.

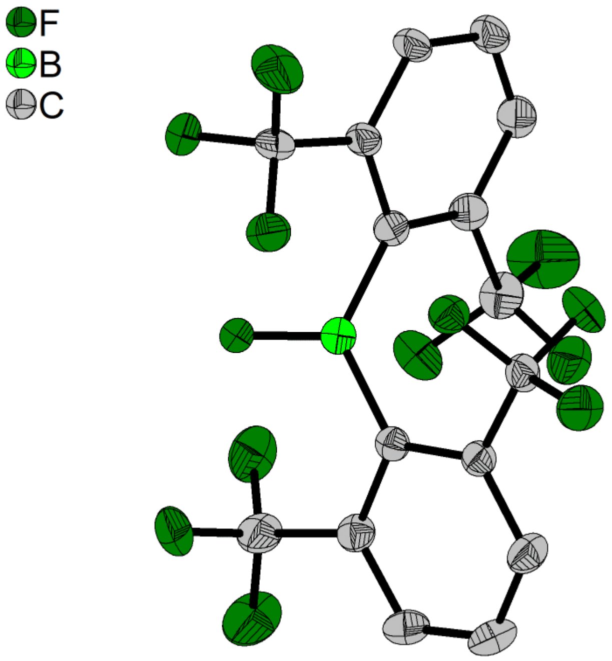

Figure S14. The solid-state molecular structure of **FB(FXyl)$_2$** determined by single-crystal X-ray diffraction at 100 K. All ellipsoids are drawn at the 50% probability level, and H atoms are omitted for clarity. One of the xylyl groups is disordered. Only the part with 80.2 % occupancy is shown here.

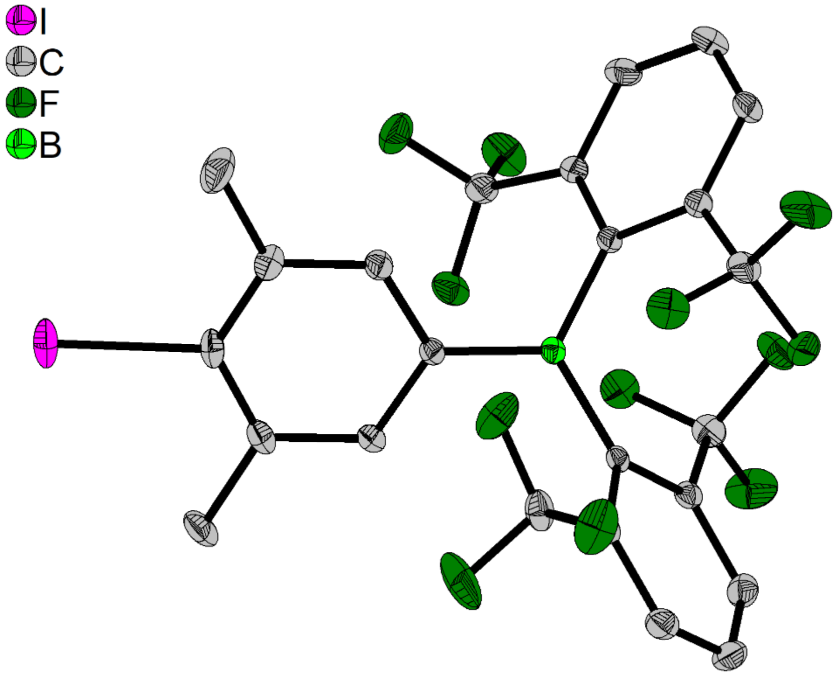

Figure S15. The solid-state molecular structure of **iii** determined by single-crystal X-ray diffraction at 100 K. All ellipsoids are drawn at the 50% probability level, and H atoms are omitted for clarity.

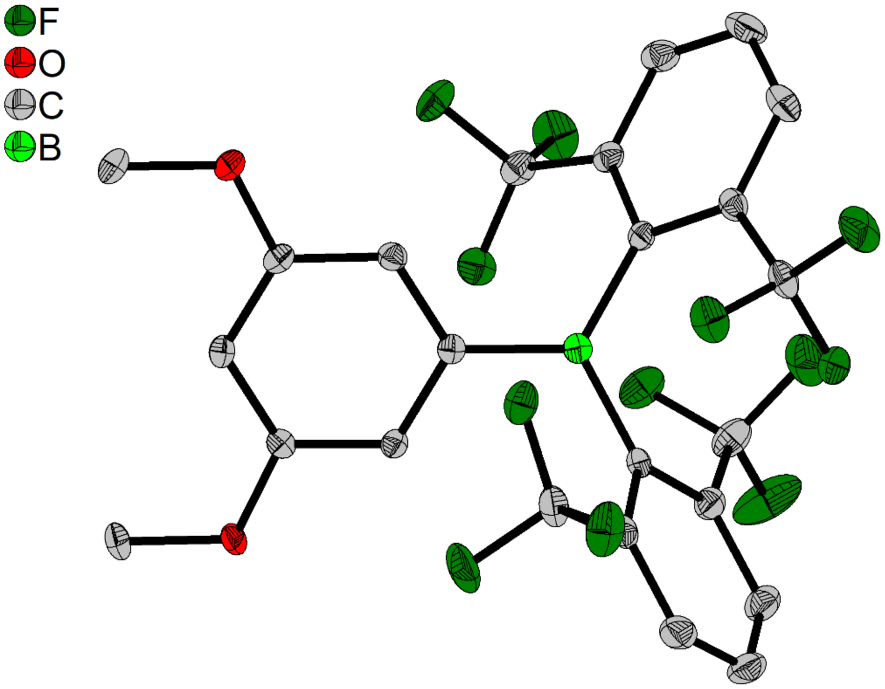

Figure S16. The solid-state molecular structure of **iv’** determined by single-crystal X-ray diffraction at 100 K. All ellipsoids are drawn at the 50% probability level, and H atoms are omitted for clarity.

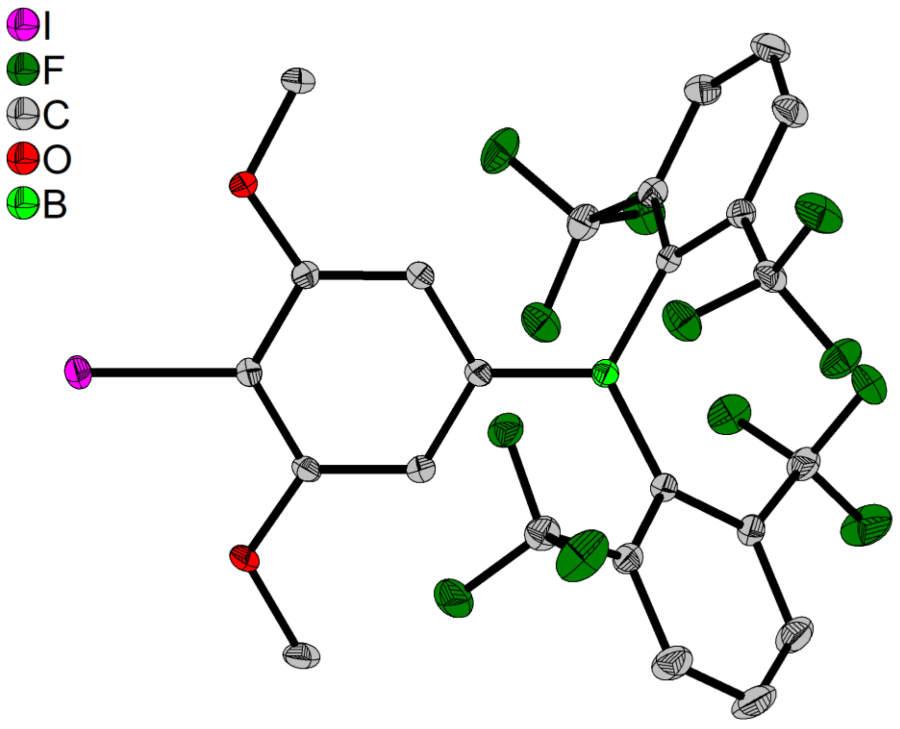

Figure S 17. The solid-state molecular structure of **iv** determined by single-crystal X-ray diffraction at 100 K. All ellipsoids are drawn at the 50% probability level, and H atoms are omitted for clarity.

Table S8. Selected bond lengths (Å) and angles (°) of **1** – **5**. Aryl rings are labeled according to Figure 2.

	**1**	**2**	**3**	**4**	**5**
B–C	1.557(2)	1.548(2)	1.553(3)	1.563(3)	1.568(4)
	1.598(2)	1.603(2)	1.597(3)	1.598(3)	1.599(5)
	1.606(2)	1.605(2)	1.599(3)	1.601(4)	1.601(5)
C–N/C	1.4168(18)	1.4296(17)	1.436(2)	1.421(3)	1.501(4)
	1.4008(19)	1.3951(18)	1.401(2)	1.406(3)	1.388(4)
	1.4055(18)	1.3937(19)	1.401(2)	1.411(3)	1.400(4)
C–C (R1, central):					
a	1.408(2)	1.4008(19)	1.400(2)	1.405(3)	1.397(4)
b	1.409(2)	1.4013(19)	1.399(2)	1.393(3)	1.394(4)
c	1.385(2)	1.3848(19)	1.389(2)	1.392(3)	1.390(4)
d	1.397(2)	1.3990(19)	1.395(2)	1.402(3)	1.403(4)
e	1.393(2)	1.4002(19)	1.389(2)	1.397(3)	1.402(4)
f	1.383(2)	1.3896(19)	1.400(2)	1.388(3)	1.393(4)
∠ BC_3-(N/C)C_3	60.18(7)	44.83(7)	50.11(7)	55.51(11)	47.61(14)
∠ BC_3-R1 (central)	17.83(10)	31.25(6)	32.44(8)	34.16(12)	40.77(11)
∠ B1C_3-R2 (terminal)	67.13(6)	47.98(6)	54.07(6)	55.44(8)	59.87(11)
∠ B1C_3-R3 (terminal)	54.91(6)	53.04(6)	47.17(8)	43.51(9)	38.64(15)
∠ NC_3-R1 (central)	43.06(7)	76.08(6)	82.05(7)	89.55(8)	88.37(12)
∠ N/C-moiety-R1 (central)	43.64(5)	76.36(4)	81.54(5)	88.37(5)	87.73(10)
Sum ∠ CBC	359.99(12)	359.99(12)	359.95(15)	360.0(2)	360.0(3)
Sum ∠ CNC/CCC	359.99(12)	357.01(11)	359.83(14)	356.31(18)	359.6(3)
Shortest B–F	1.3338(19)	1.330(2)	1.327(2)	1.326(3)	1.325(4)
	1.3367(19)	1.3357(18)	1.329(2)	1.332(3)	1.330(4)
	1.3398(19)	1.3388(18)	1.337(2)	1.339(3)	1.332(4)
		1.3395(16)	1.338(2)	1.340(3) 2x	1.333(4) 3x
			1.339(2) 3x		1.337(4)
					1.338(4)
					1.339(4)

Table S 9. Aryl···aryl (π···π) distances (Å) in crystals of **1** at 100 K: centroid-centroid distance, interplanar separation, and offset shift.

**Aryl···Aryl**	Centroid-centroid distance	Interplanar separation	Offset shift
**Phen···Phen**	3.712(2)	3.447(2)	1.378(4)

The structures of the intermediates iii, iv, FB(^{F}Xyl)$_2$ and ^{MeO}π-B(^{F}Xyl)$_2$ along with their structural parameters can be found in Figures S9-S17 and Table S6. The acceptor moieties in compounds **1** - **5** exhibit one shorter B–C bond to the bridge (R1) (1.548(2) - 1.568(4) Å) and two longer B–C bonds to the terminal ^{F}Xyl groups (1.597(3) - 1.606(2) Å) (Table S7). This can be partially attributed to the donor – acceptor nature of the compounds, which will shorten the B–C bond to R1, as well as the larger steric hinderance of the ^{F}Xyl moieties, which will elongate the B–C bonds to R2 and R3. An opposite behavior is observed for the nitrogen donor moieties in compounds **1** - **4**, which exhibit one longer C–N bond to R1 (1.417(2) - 1.436(2) Å) and two shorter C–N bonds (1.394(2) - 1.411(3) Å) to the terminal aryls of the donor moieties (Table S7). The same phenomenon is observable in compound **5**, in which the donor is connected to R1 via a C–C bond instead of a C–N bond, i.e., the C–C bond to R1 is longer than the aromatic C–C bonds of the donor moiety. For both the C–C and the C–N bonds, this is an expected behavior as the central carbon and nitrogen atoms are part of a heteroaryl or aryl moiety.

NMR spectra

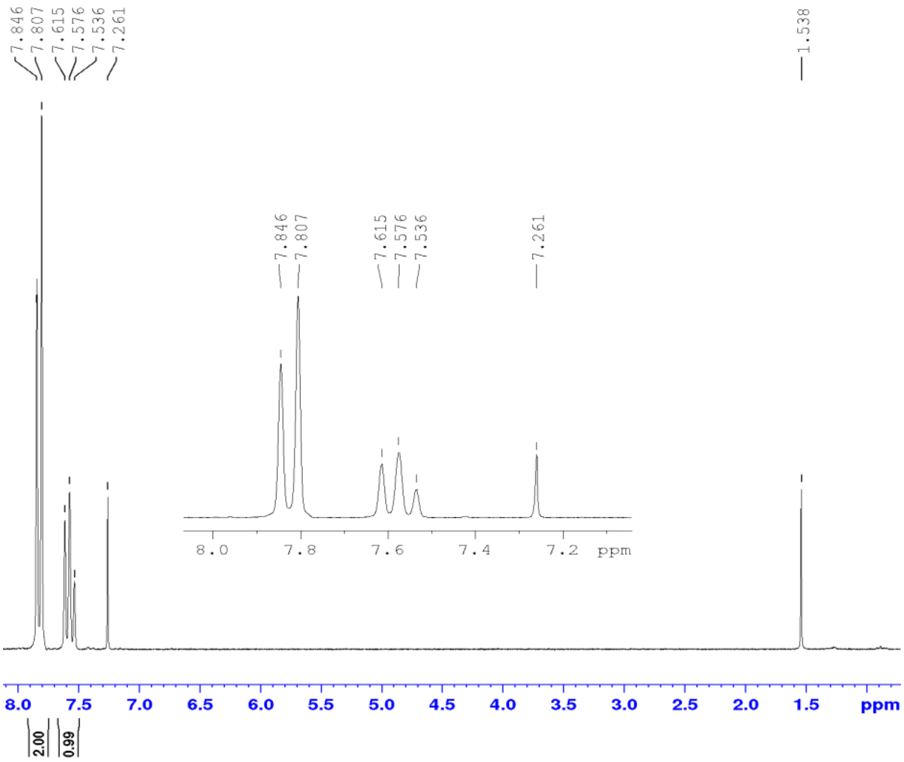

Figure S18. ^{1}H NMR spectrum (200 MHz, 298 K, $CDCl_3$) of **2-iodo-1,3-bis(trifluoromethyl)benzene**.

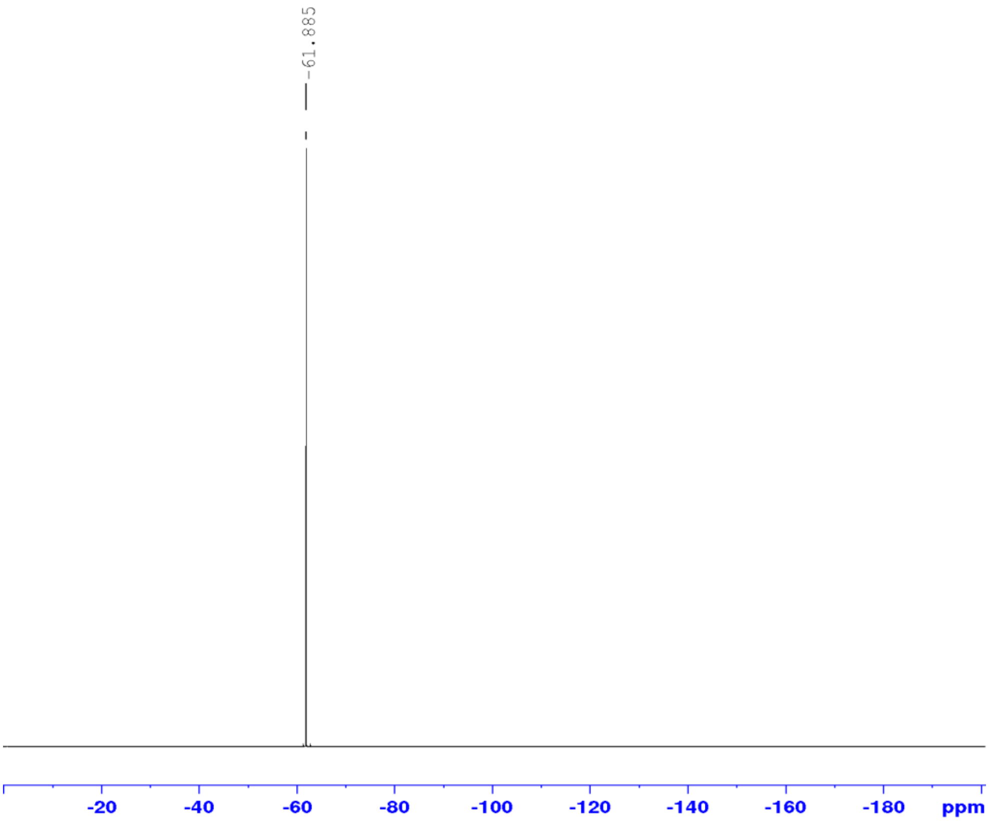

Figure S19. ^{19}F NMR spectrum (188 MHz, 298 K, $CDCl_3$) of **2-iodo-1,3-bis(trifluoromethyl)benzene**.

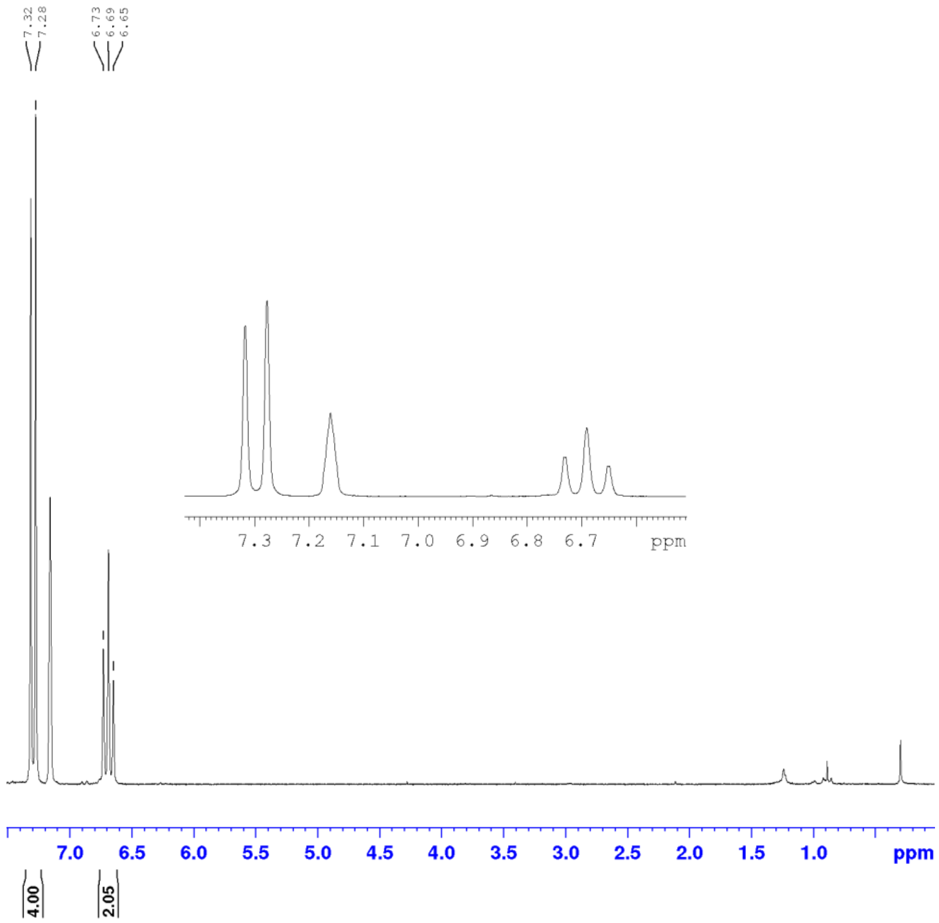

Figure S20. ^{1}H NMR spectrum (200 MHz, 298 K, C_6D_6) of **FB(FXyl)$_2$**.

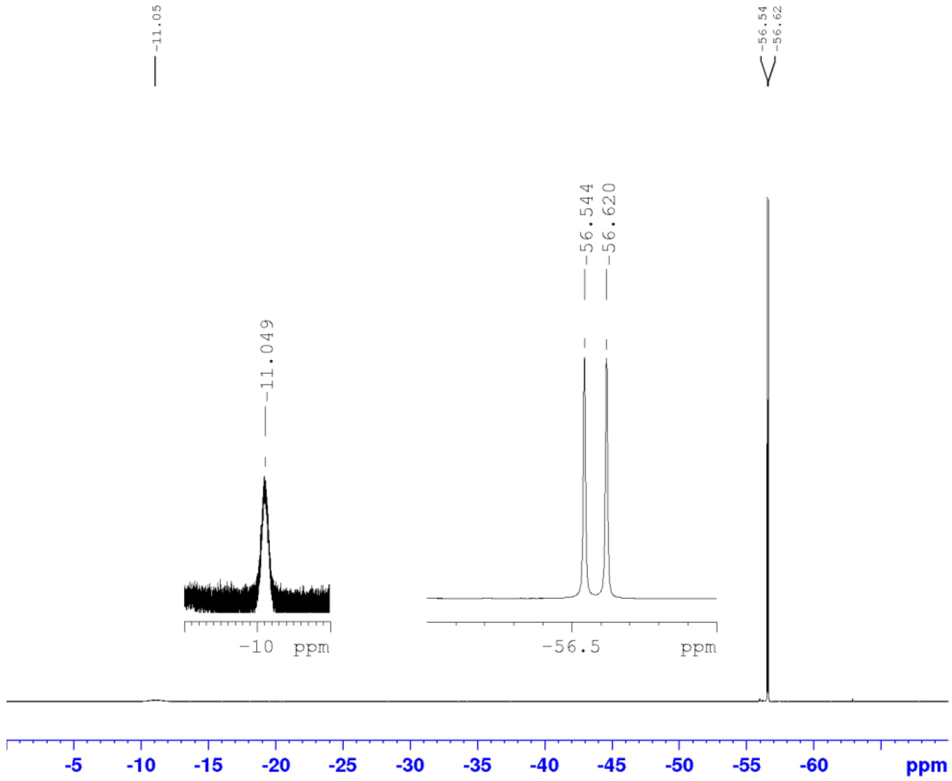

Figure S21. ^{19}F NMR spectrum (188 MHz, 298 K, C_6D_6) of **FB(FXyl)$_2$**.

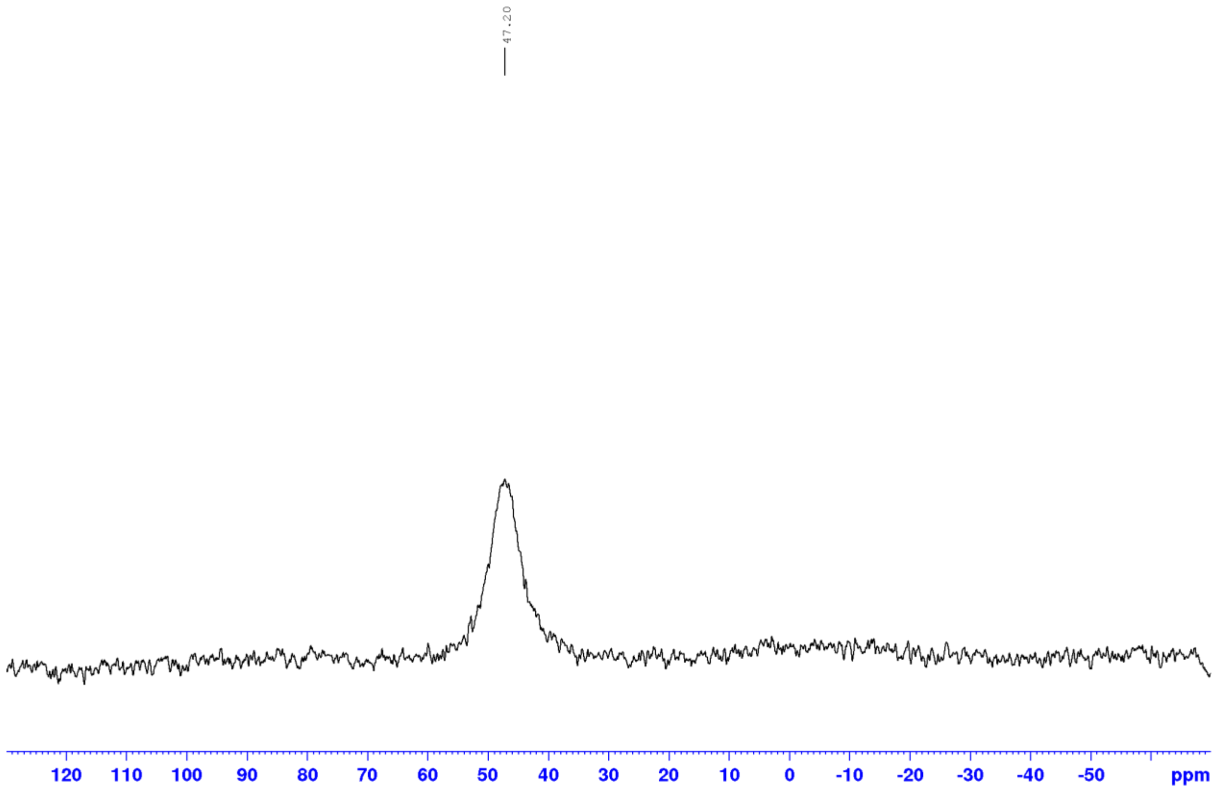

Figure S 22. ^{11}B NMR spectrum (64 MHz, 298 K, C_6D_6) of **FB(FXyl)$_2$**.

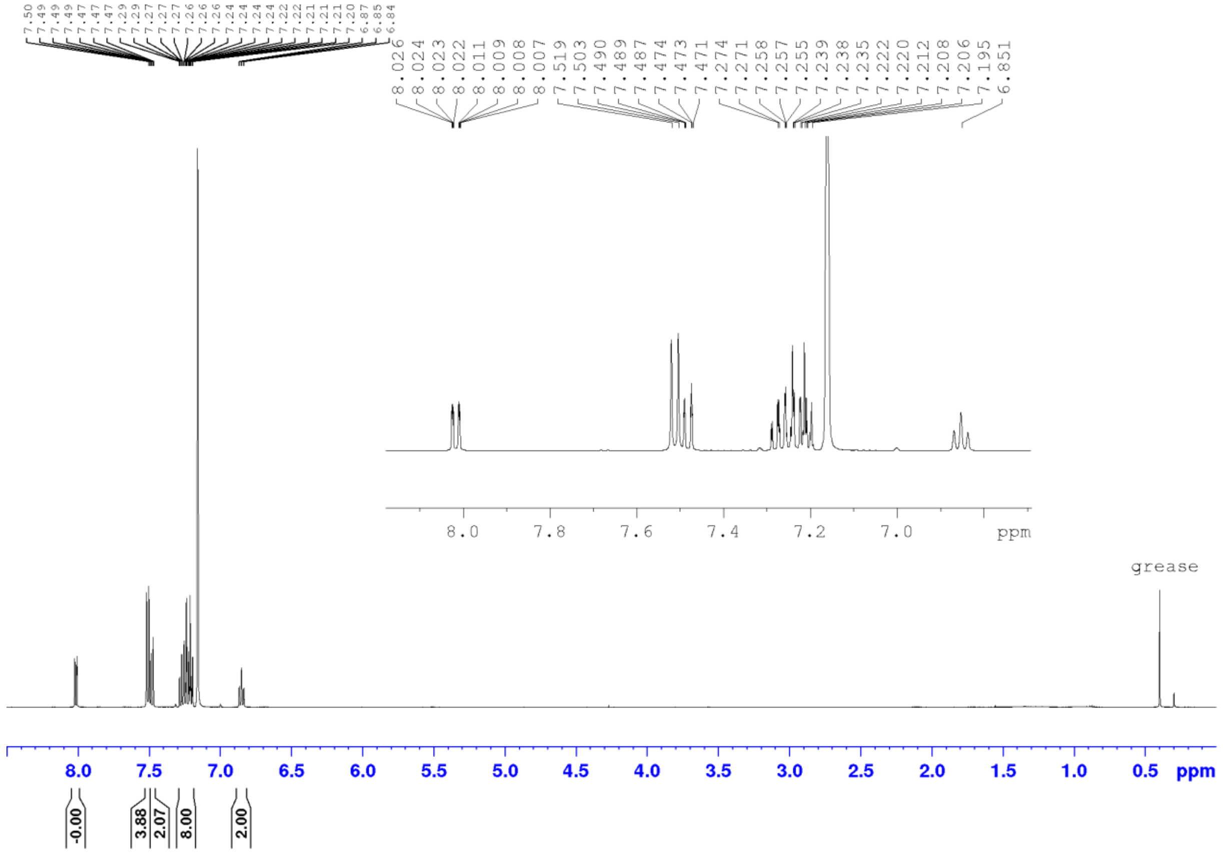

Figure S 23. ^{1}H NMR spectrum (500 MHz, 298 K, C_6D_6) of compound **1**.

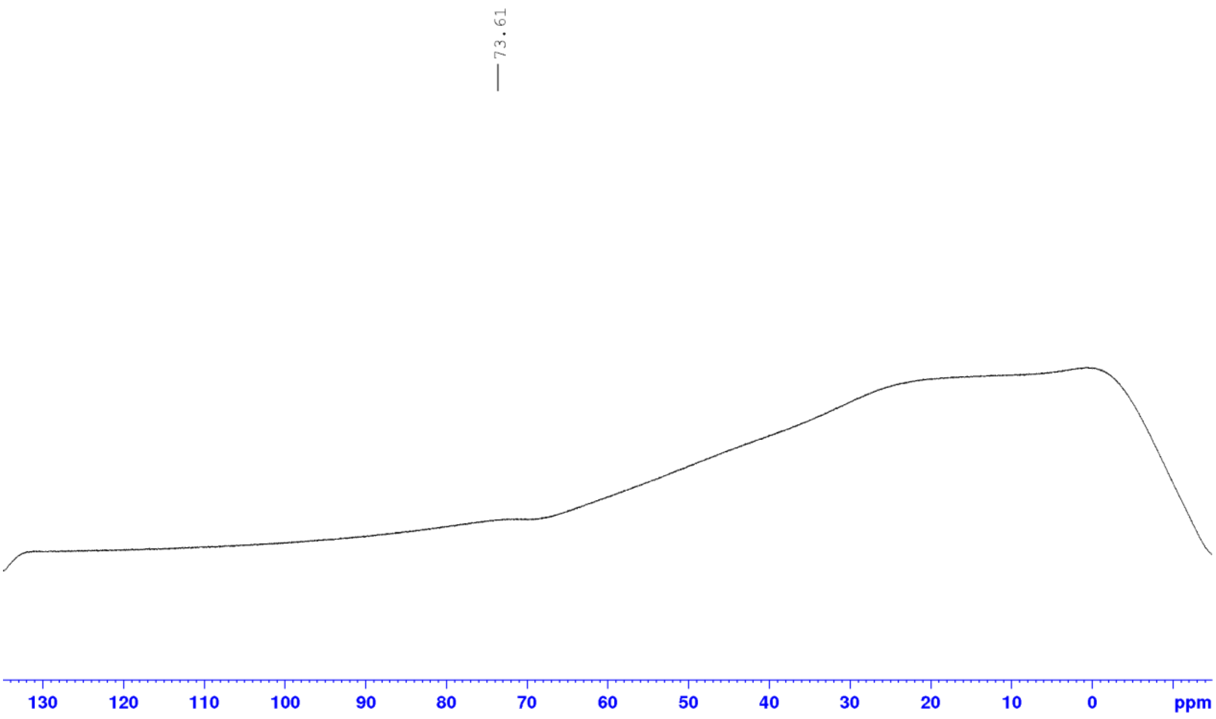

Figure S 24. $^{11}B\{^{1}H\}$ NMR spectrum (160 MHz, 298 K, C_6D_6) of compound **1**.

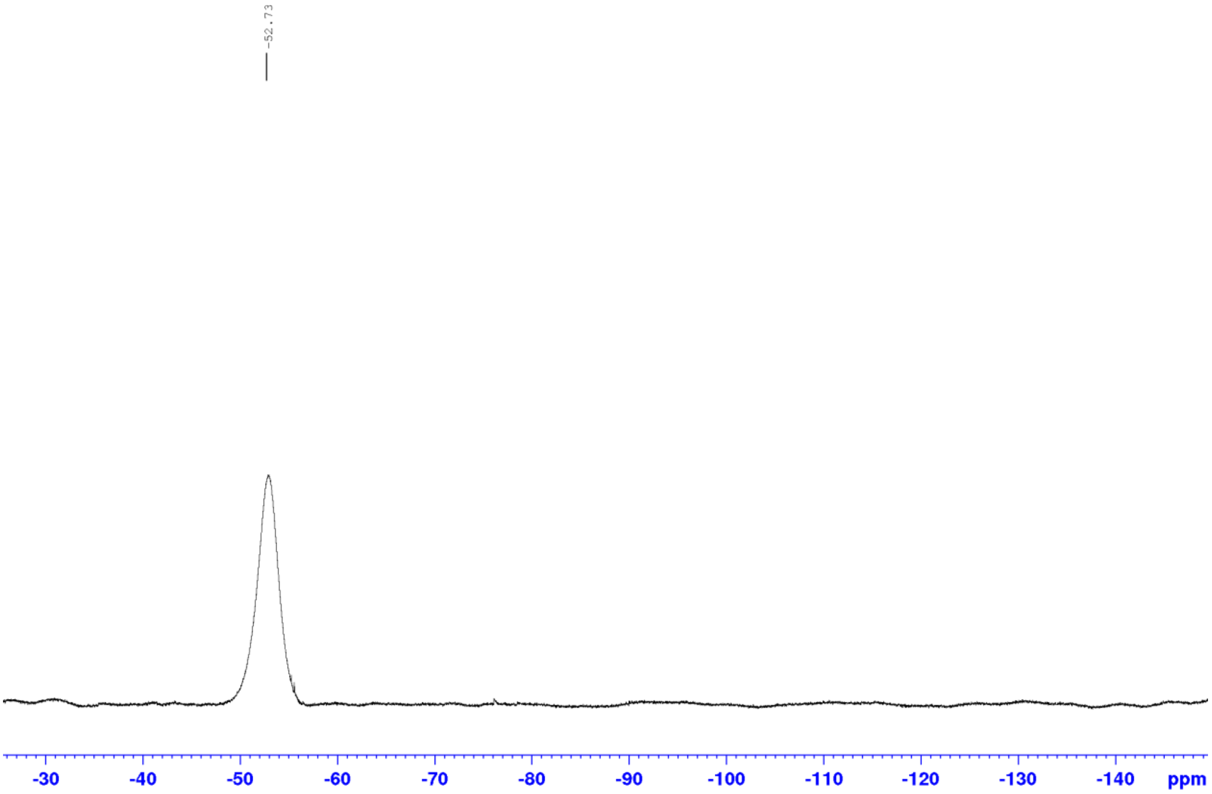

Figure S 25. $^{19}F\{^{1}H\}$ NMR spectrum (470 MHz, 298 K, C_6D_6) of compound **1**

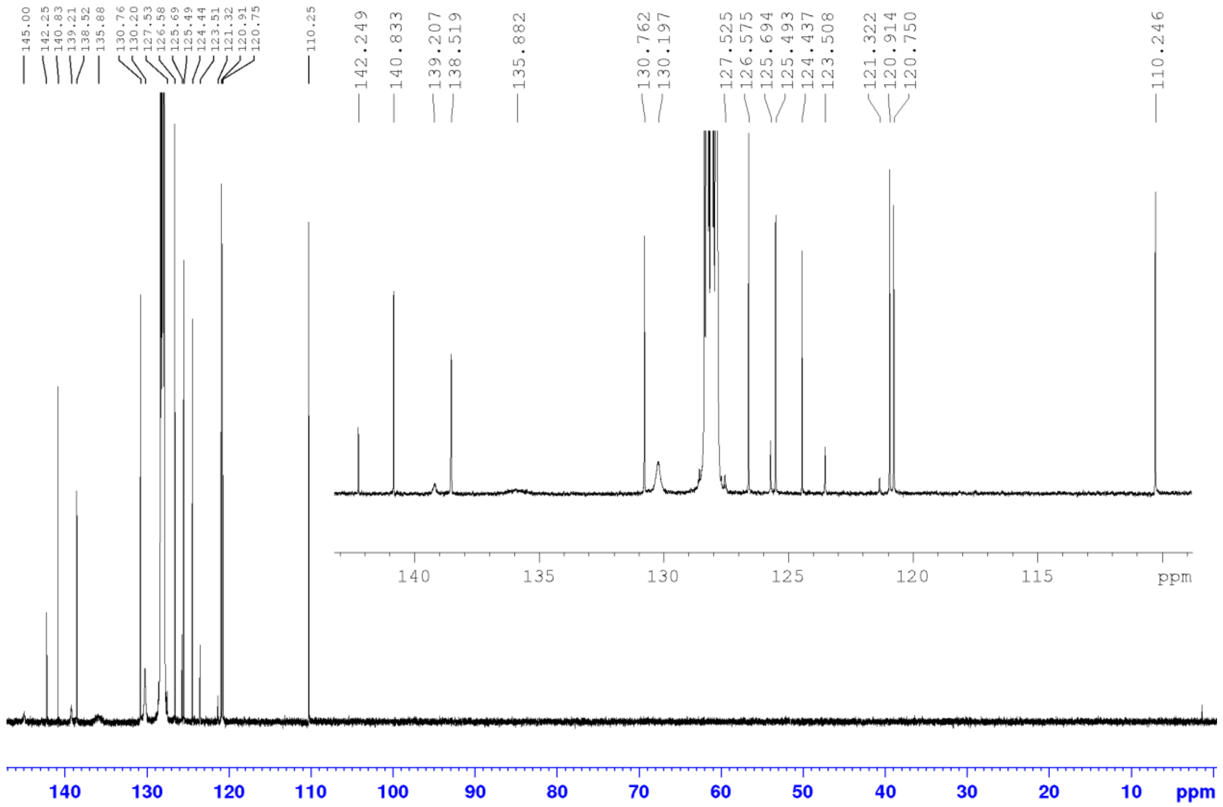

Figure S26. $^{13}C\{^{1}H\}$ NMR spectrum (126 MHz, 298 K, C_6D_6) of compound **1**.

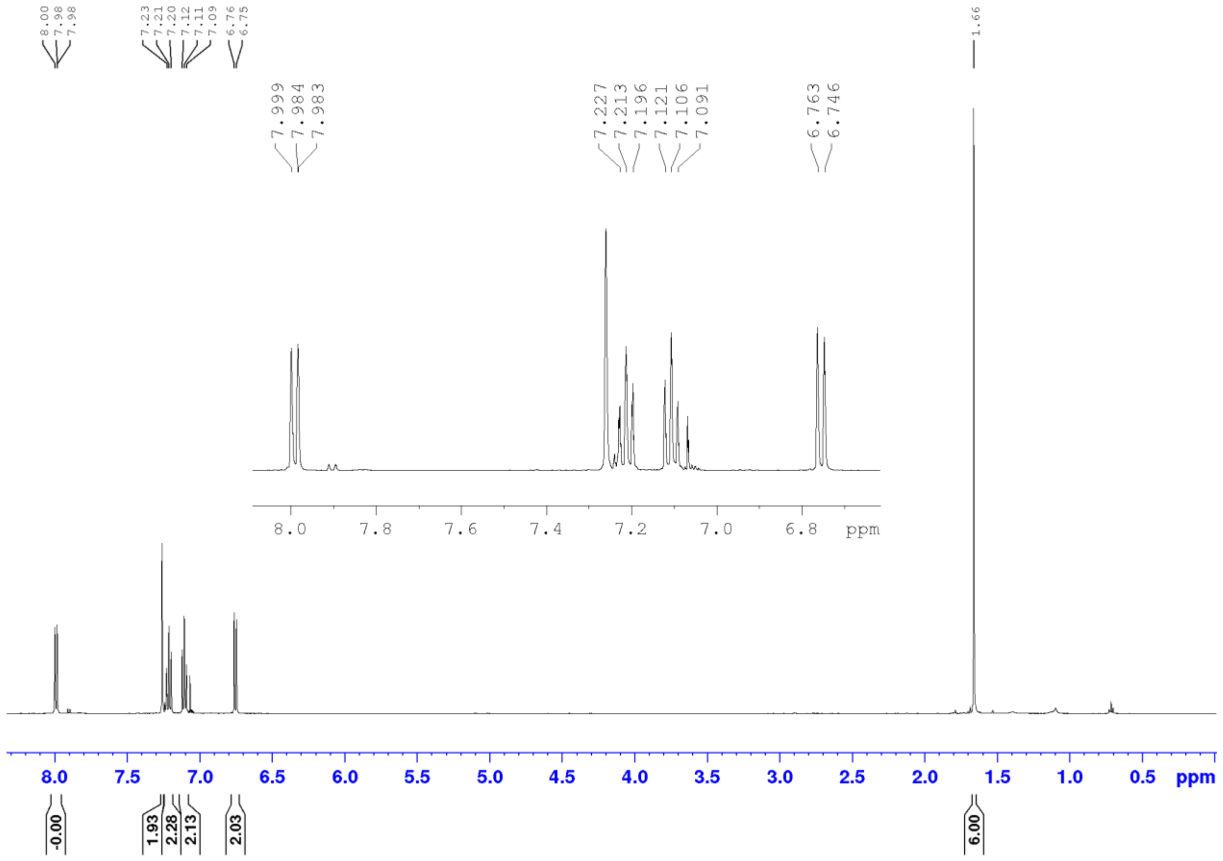

Figure S27. ^{1}H NMR spectrum (500 MHz, 298 K, $CDCl_3$) of **9-(4-bromo-2,6-dimethylphenyl)carbazole** (**ii**).

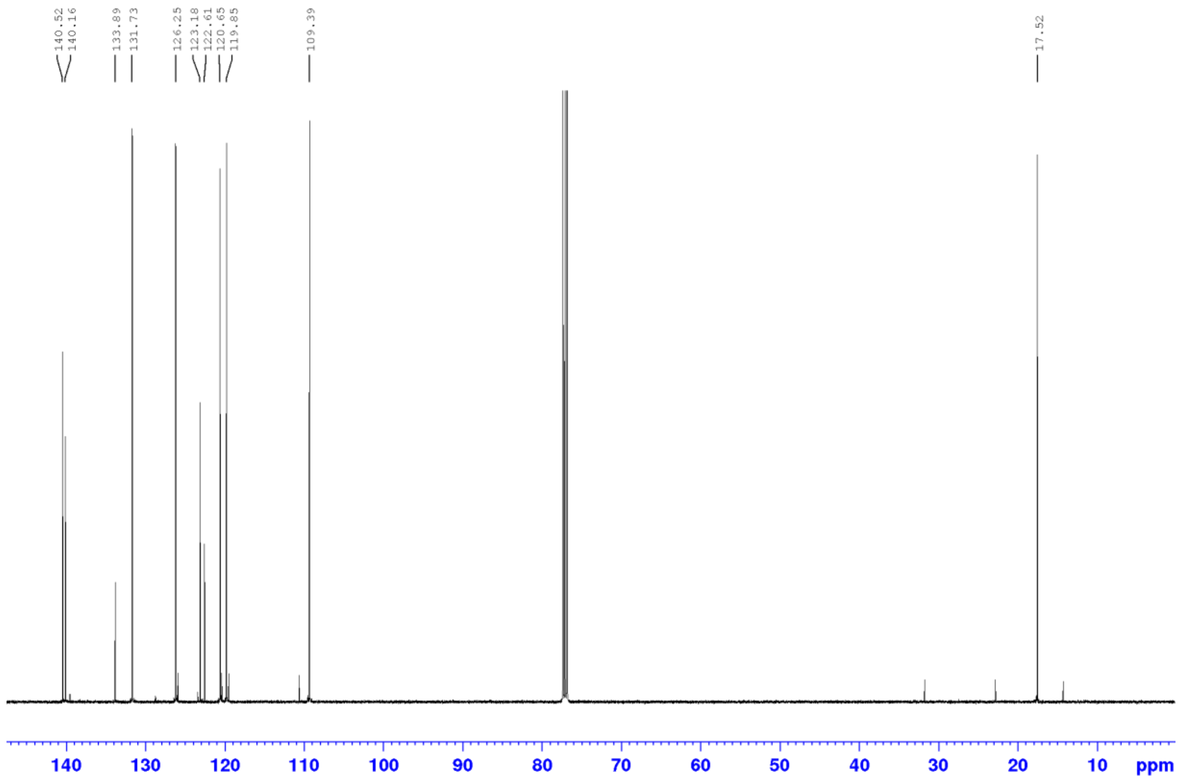

Figure S28. $^{13}C\{^{1}H\}$ NMR spectrum (126 MHz, 298 K, $CDCl_3$) of **9-(4-bromo-2,6-dimethylphenyl)carbazole** (**ii**).

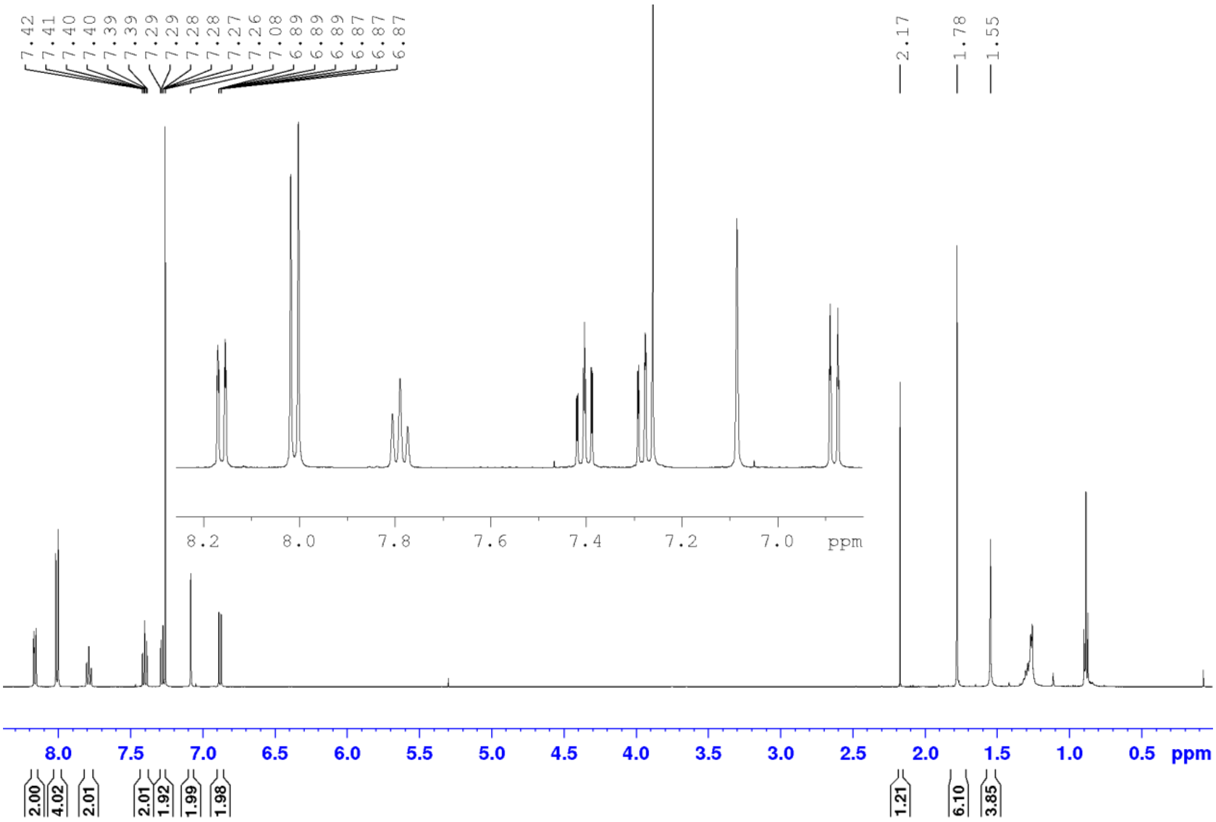

Figure S29. ^{1}H NMR spectrum (500 MHz, 298 K, $CDCl_3$) of compound **2**.

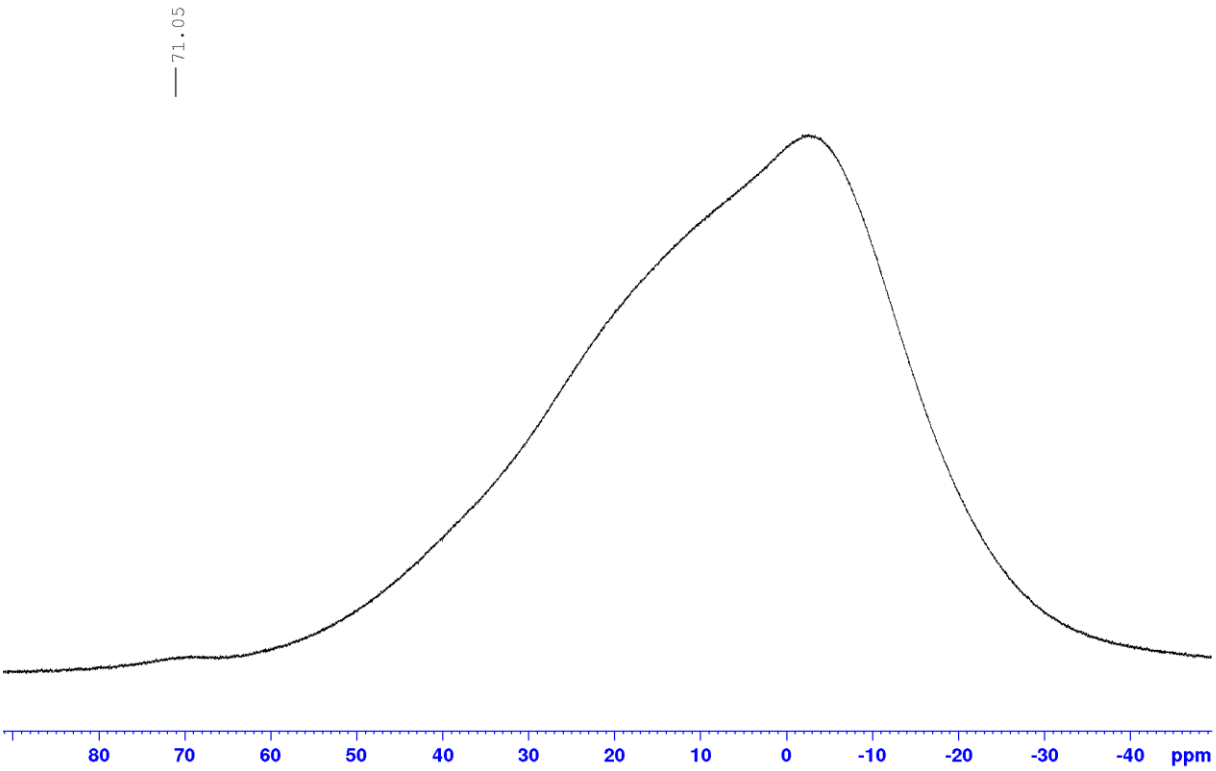

Figure S30. ^{11}B{^{1}H} NMR spectrum (160 MHz, 298 K, $CDCl_3$) of compound **2**.

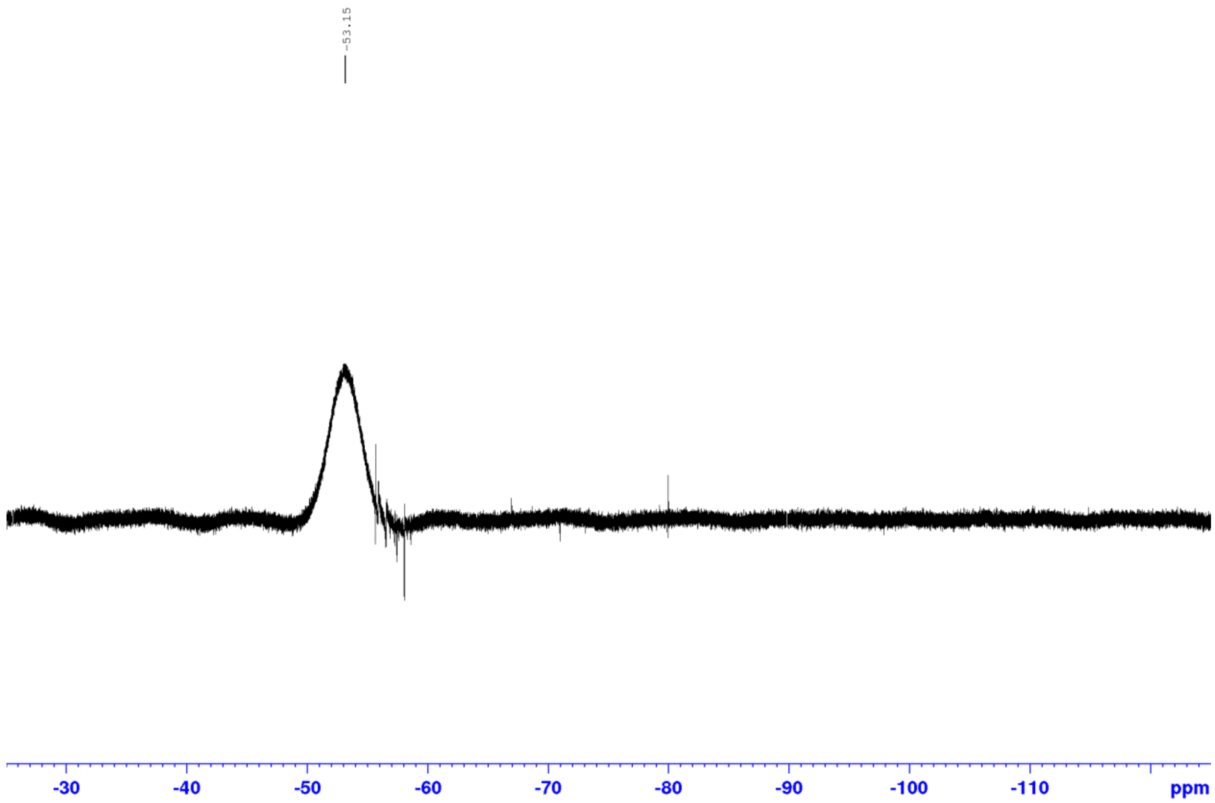

Figure S31. $^{19}F\{^1H\}$ NMR spectrum (470 MHz, 298 K, $CDCl_3$) of compound **2**.

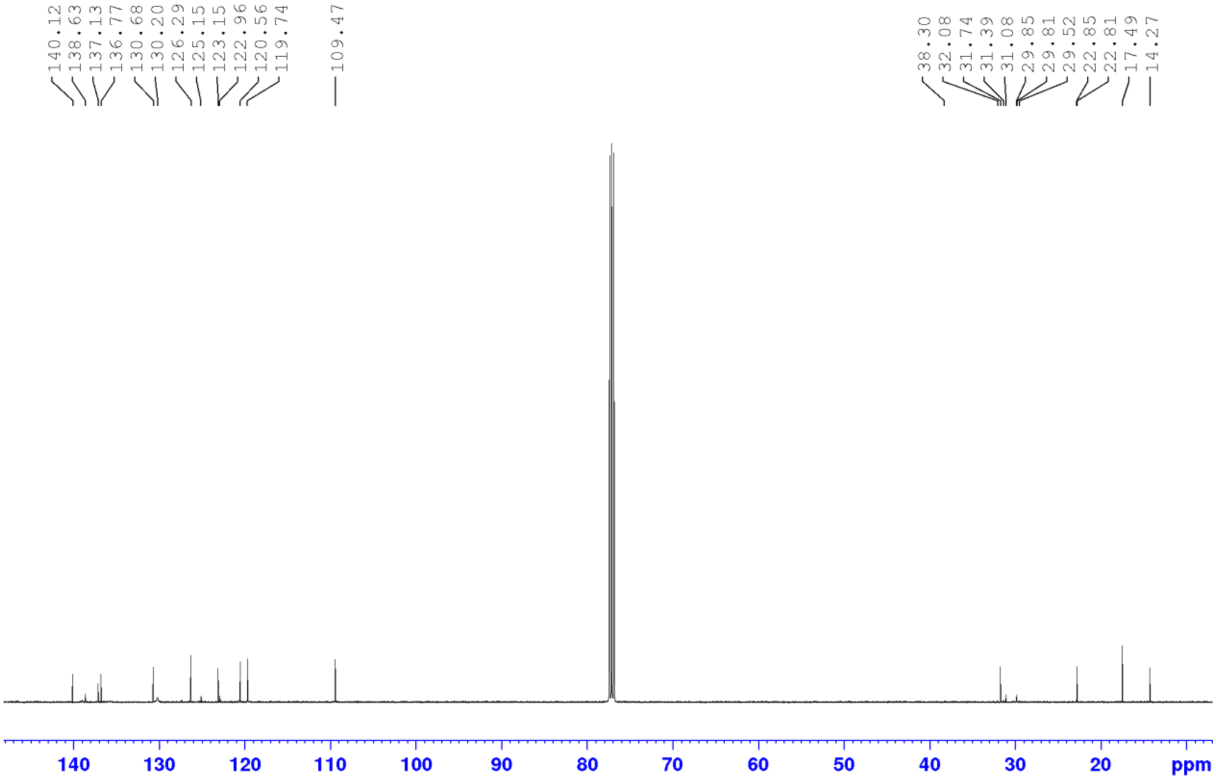

Figure S32. $^{13}C\{^{1}H\}$ NMR spectrum (126 MHz, 298 K, $CDCl_3$) of compound **2**.

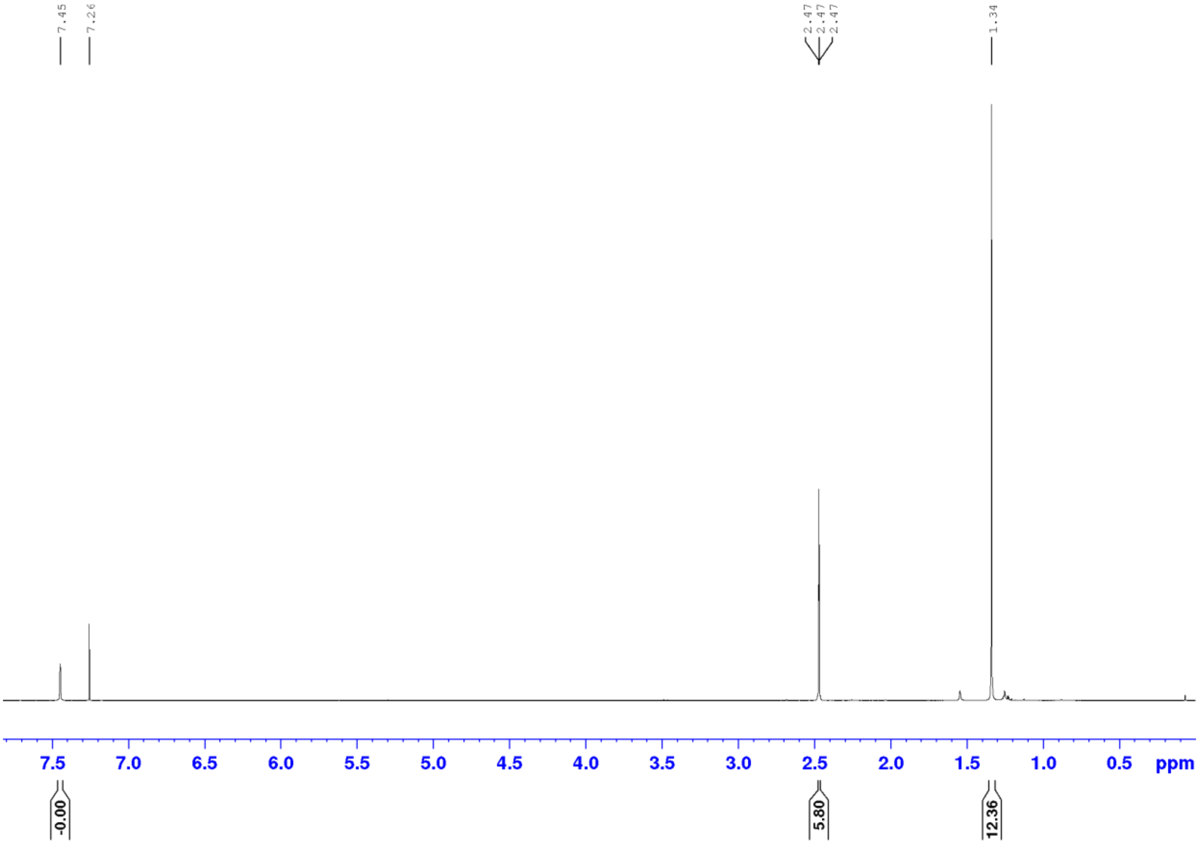

Figure S33. ^{1}H NMR spectrum (500 MHz, 298 K, $CDCl_3$) of **2-(4-iodo-3,5-dimethylphenyl)-4,4,5,5-tetramethyl-1,3,2-dioxaborolane**.

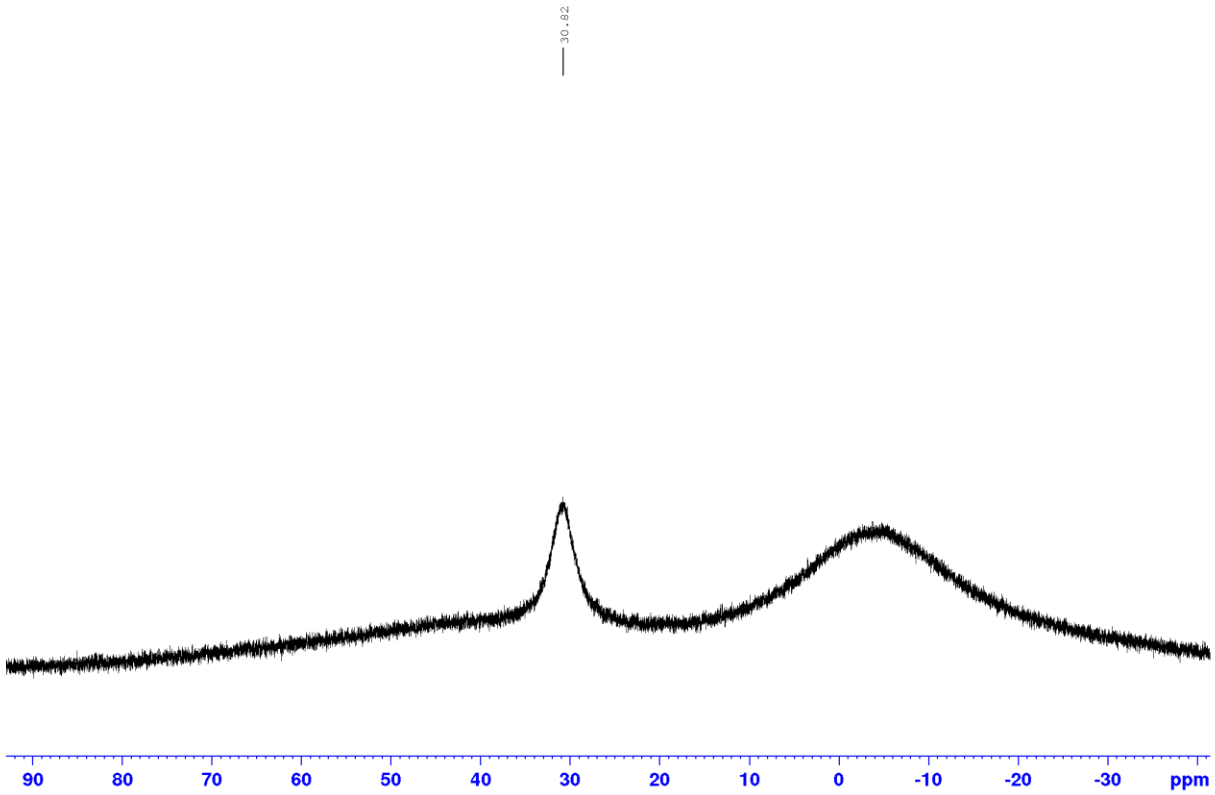

Figure S34. $^{11}B\{^{1}H\}$ NMR spectrum (160 MHz, 298 K, $CDCl_3$) **of 2-(4-iodo-3,5-dimethylphenyl)-4,4,5,5-tetramethyl-1,3,2-dioxaborolane**.

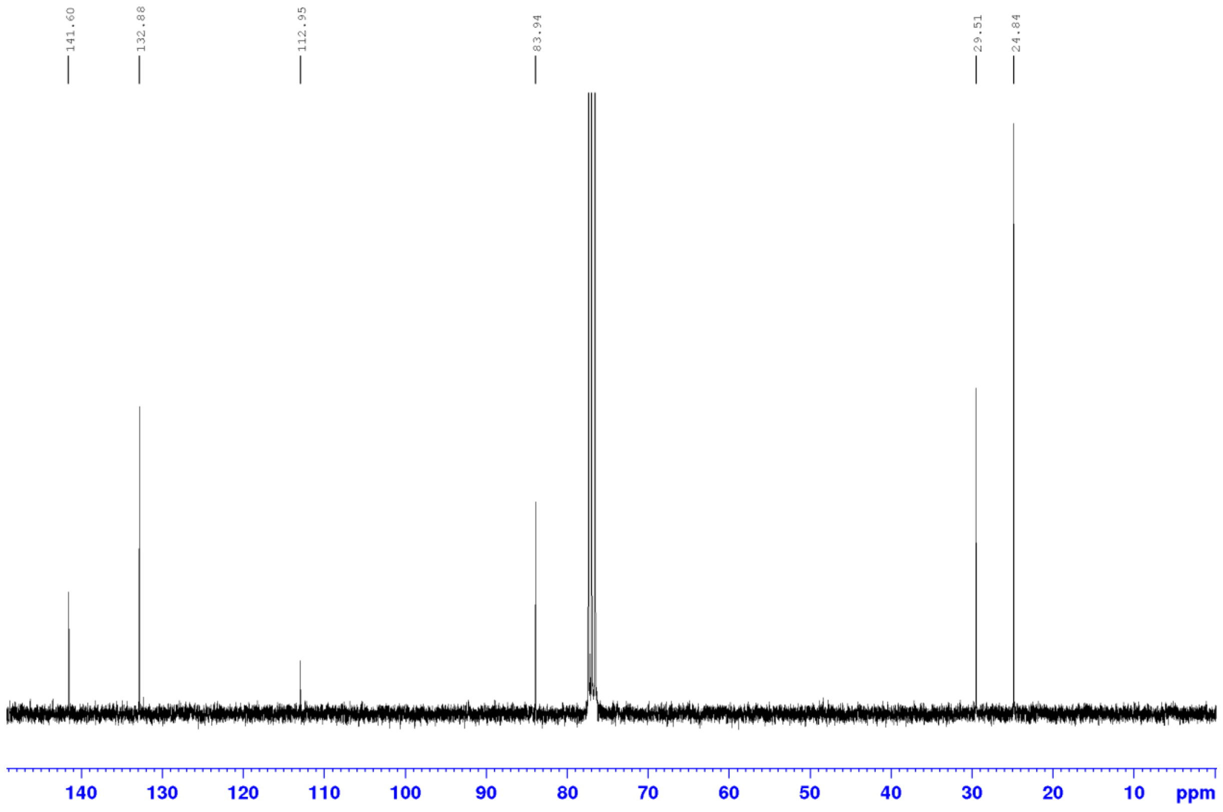

Figure S35. $^{13}C\{^1H\}$ NMR spectrum (126 MHz, 298 K, $CDCl_3$) of **2-(4-iodo-3,5-dimethylphenyl)-4,4,5,5-tetramethyl-1,3,2-dioxaborolane**.

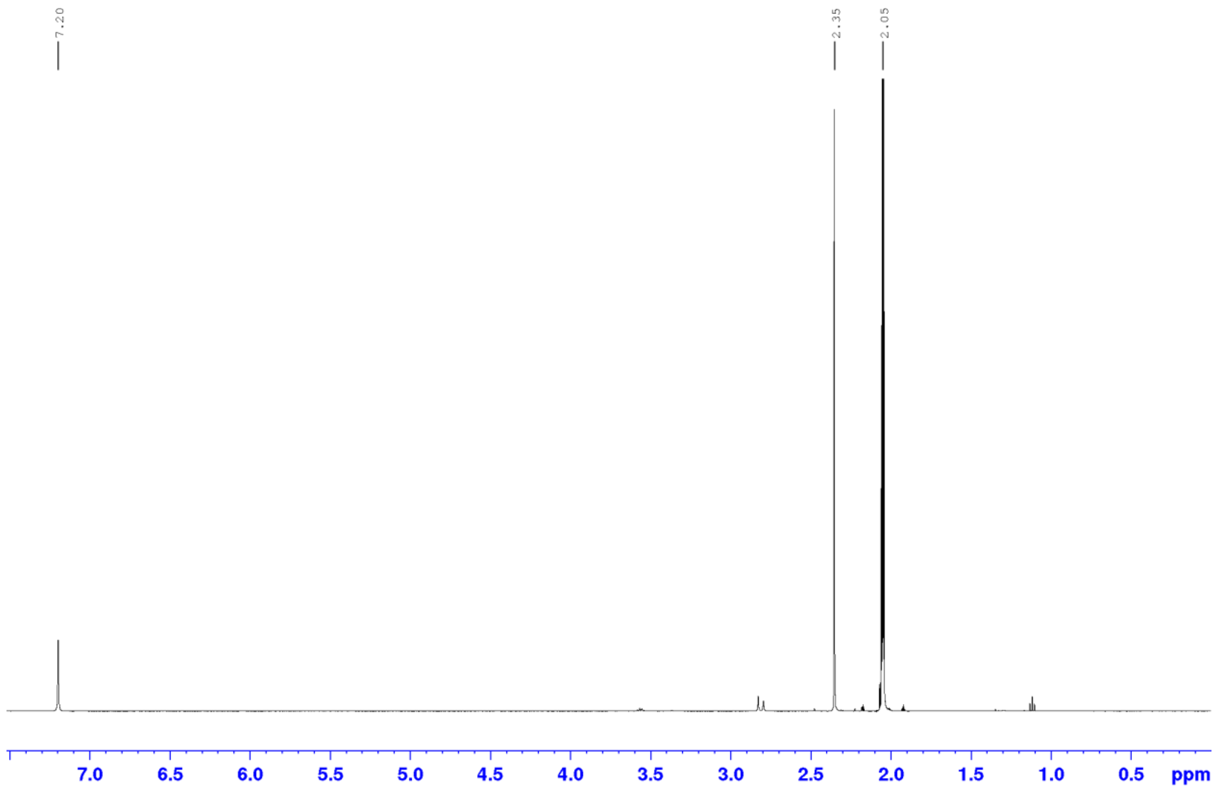

Figure S36. ^1H NMR spectrum (500 MHz, 298 K, acetone-d_6) of **potassium(4-iodo-3,5-dimethylphenyl) trifluoroborate**.

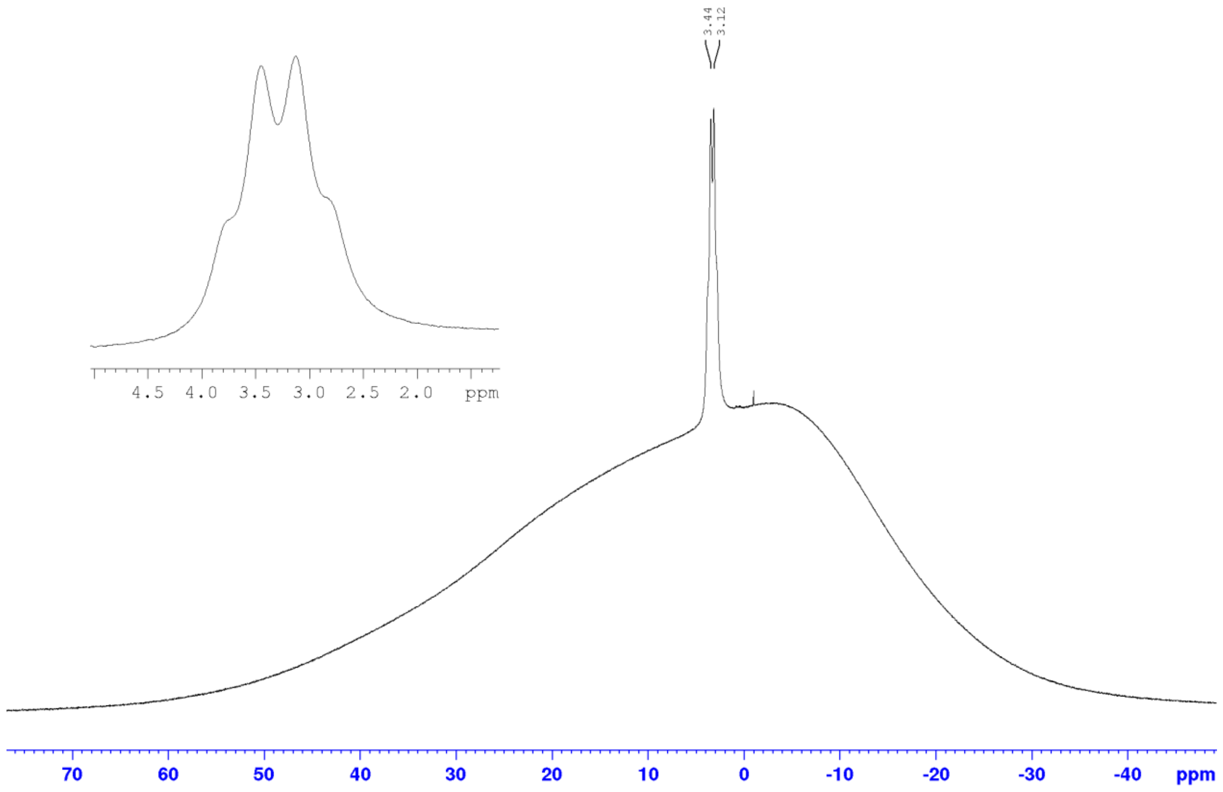

Figure S37. $^{11}B\{^{1}H\}$ NMR spectrum (160 MHz, 298 K, acetone-d_6) of **potassium(4-iodo-3,5-dimethylphenyl) trifluoroborate**.

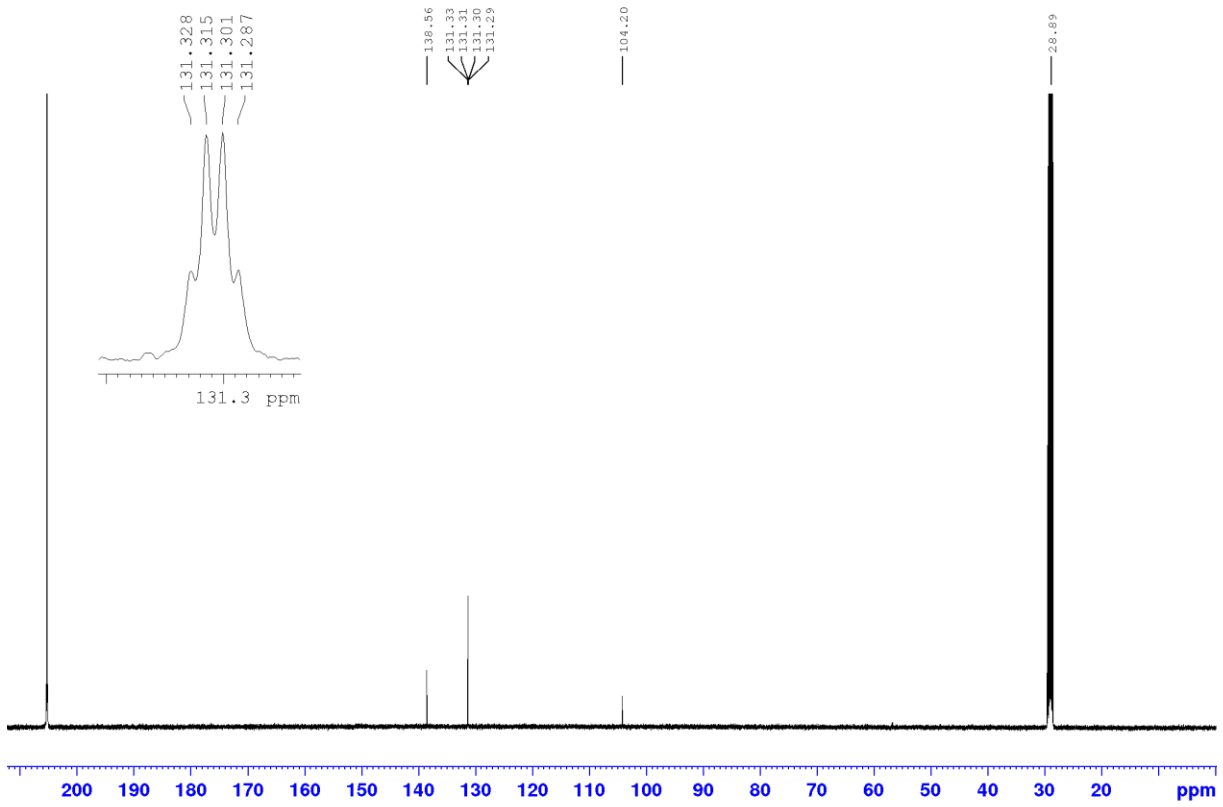

Figure S38. $^{13}C\{^{1}H\}$ NMR spectrum (126 MHz, 298 K, acetone-d_6) of **potassium(4-iodo-3,5-dimethylphenyl) trifluoroborate**.

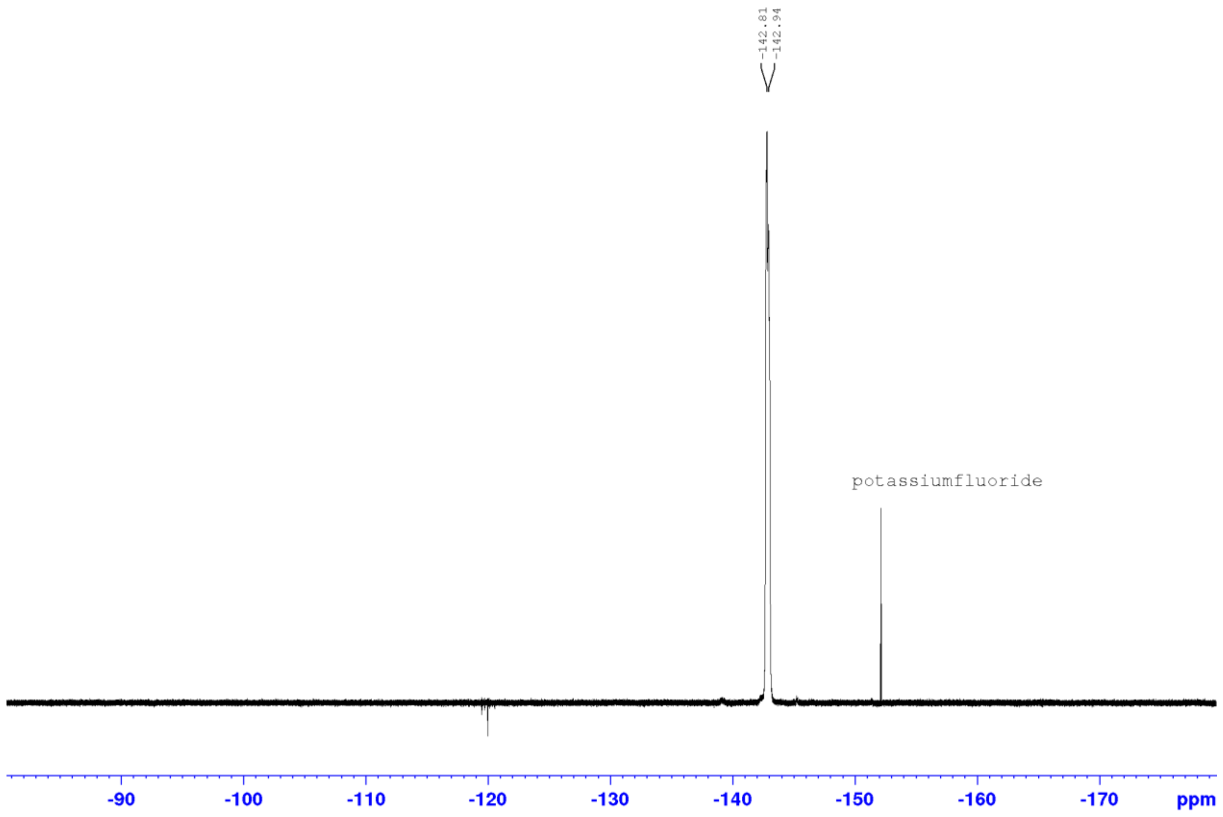

Figure S39. $^{19}F\{^{1}H\}$ NMR spectrum (470 MHz, 298 K, acetone-d$_6$) of **potassium(4-iodo-3,5-dimethylphenyl) trifluoroborate**.

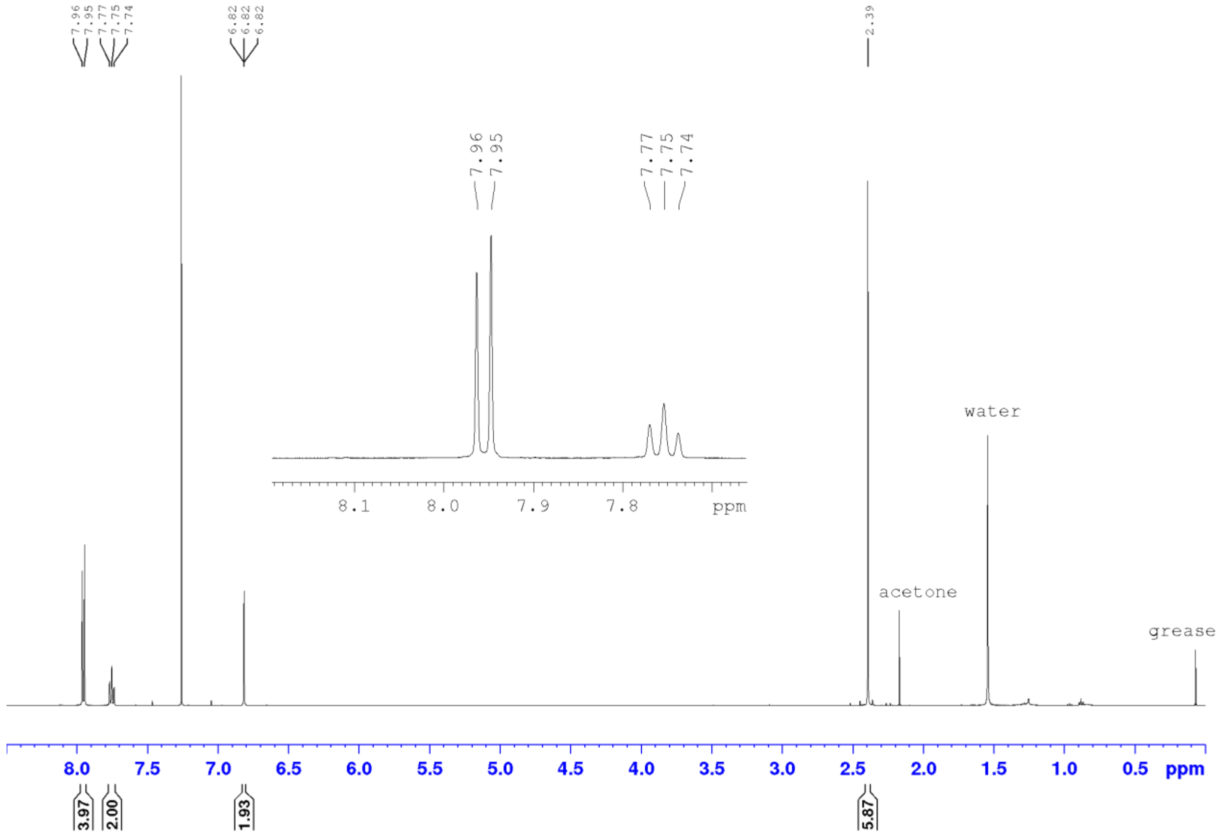

Figure S40. ^{1}H NMR spectrum (500 MHz, 298 K, $CDCl_3$) of compound **iii**.

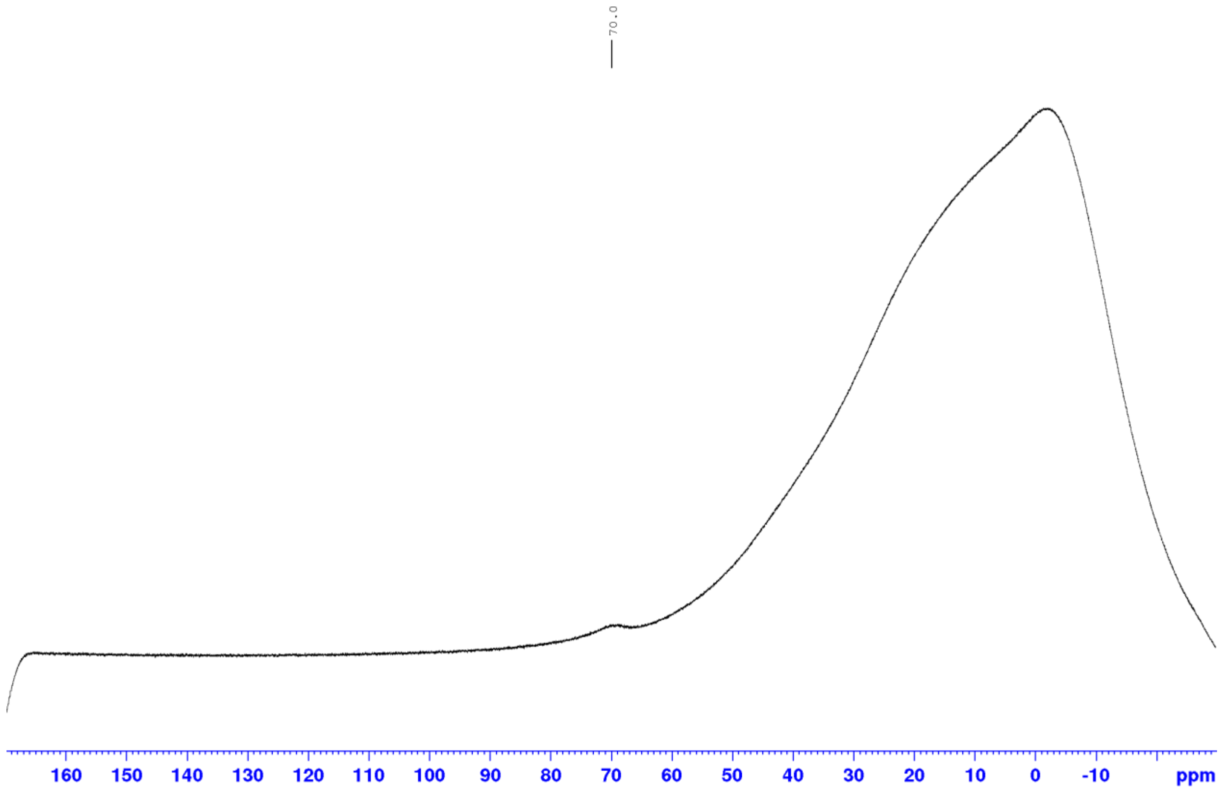

Figure S41. $^{11}B\{^{1}H\}$ NMR spectrum (160 MHz, 298 K, $CDCl_3$) of compound **iii**.

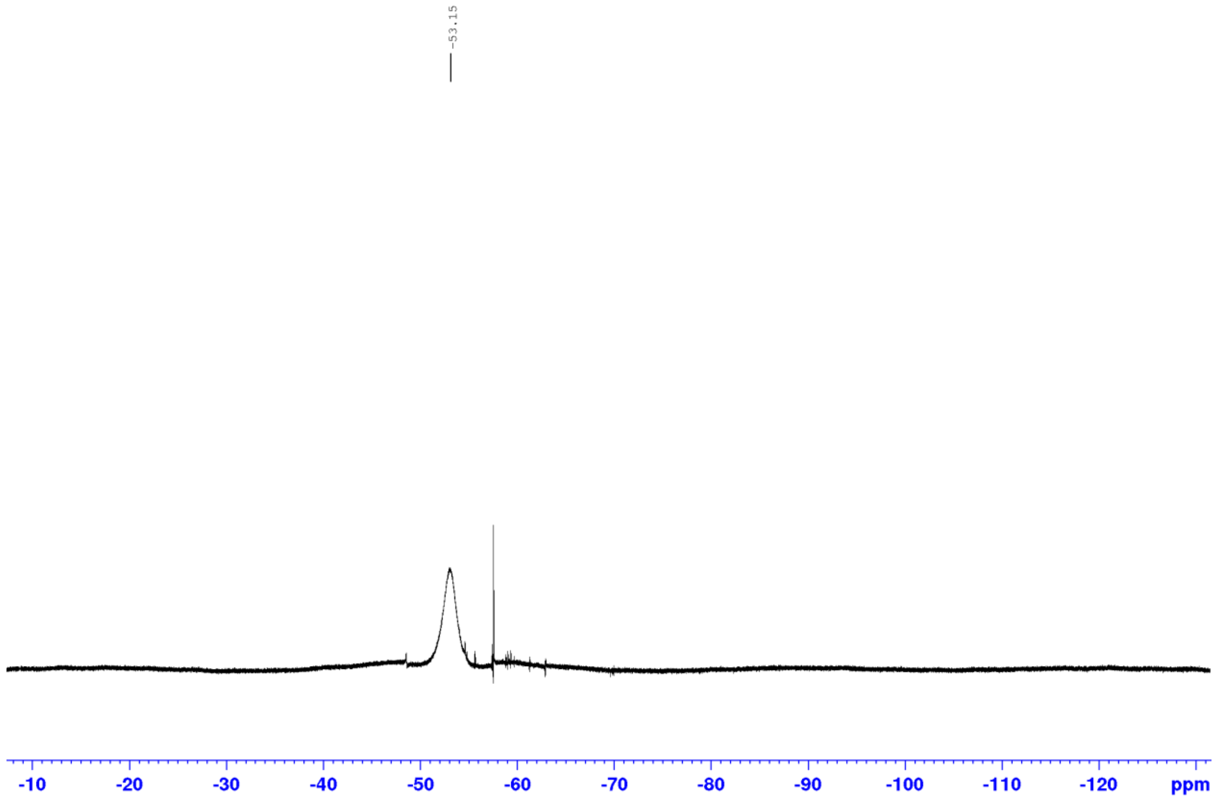

Figure S42. $^{19}F\{^{1}H\}$ NMR spectrum (470 MHz, 298 K, $CDCl_3$) of compound **iii**.

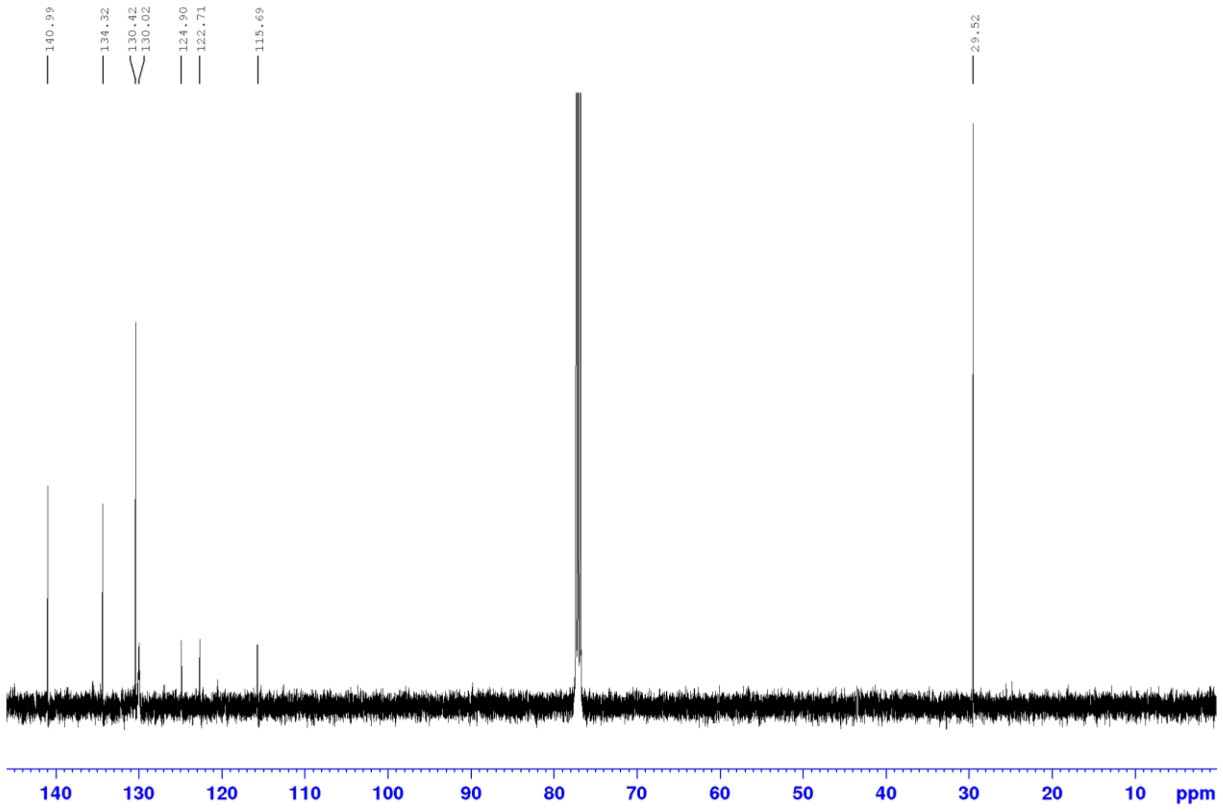

Figure S43. $^{13}C\{^{1}H\}$ NMR spectrum (126 MHz,298 K, $CDCl_3$) of compound **iii**.

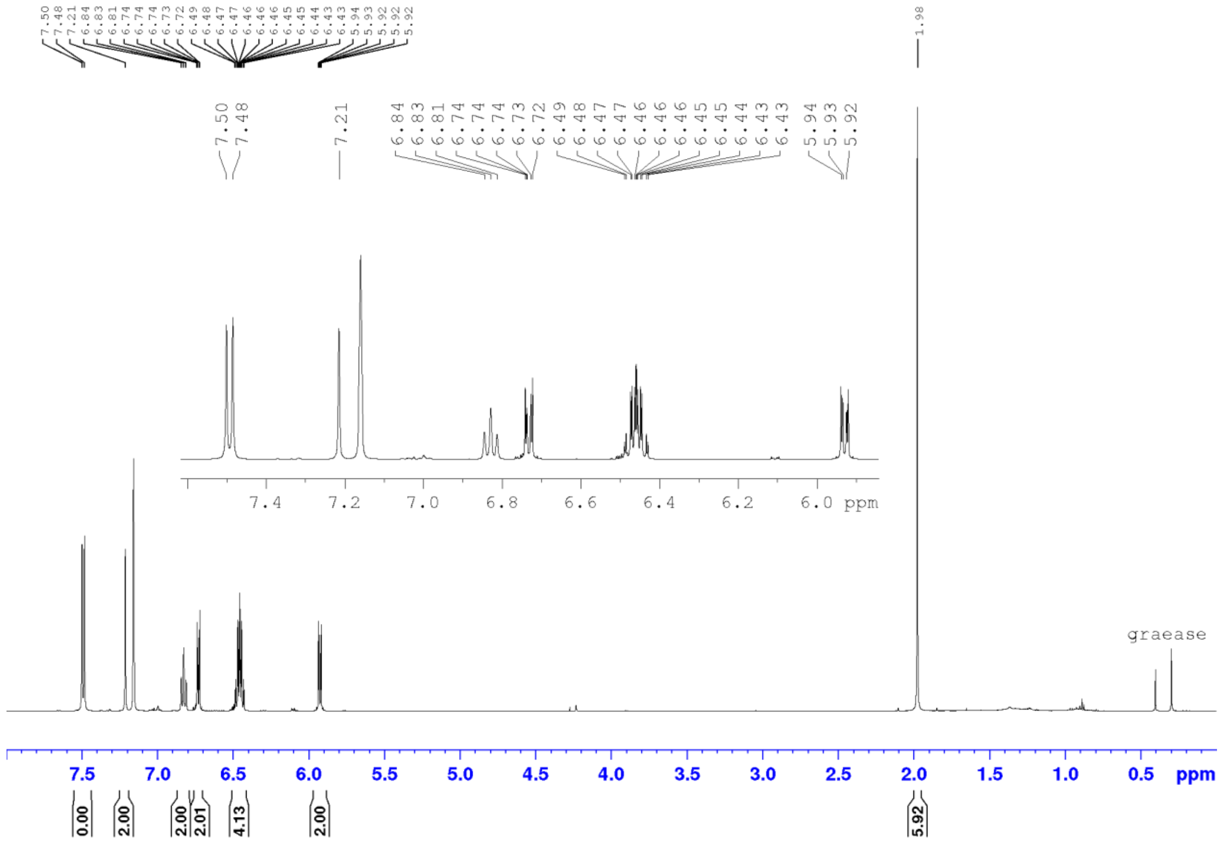

Figure S44. ^{1}H NMR spectrum (500 MHz, 298 K, C_6D_6) of compound **3**.

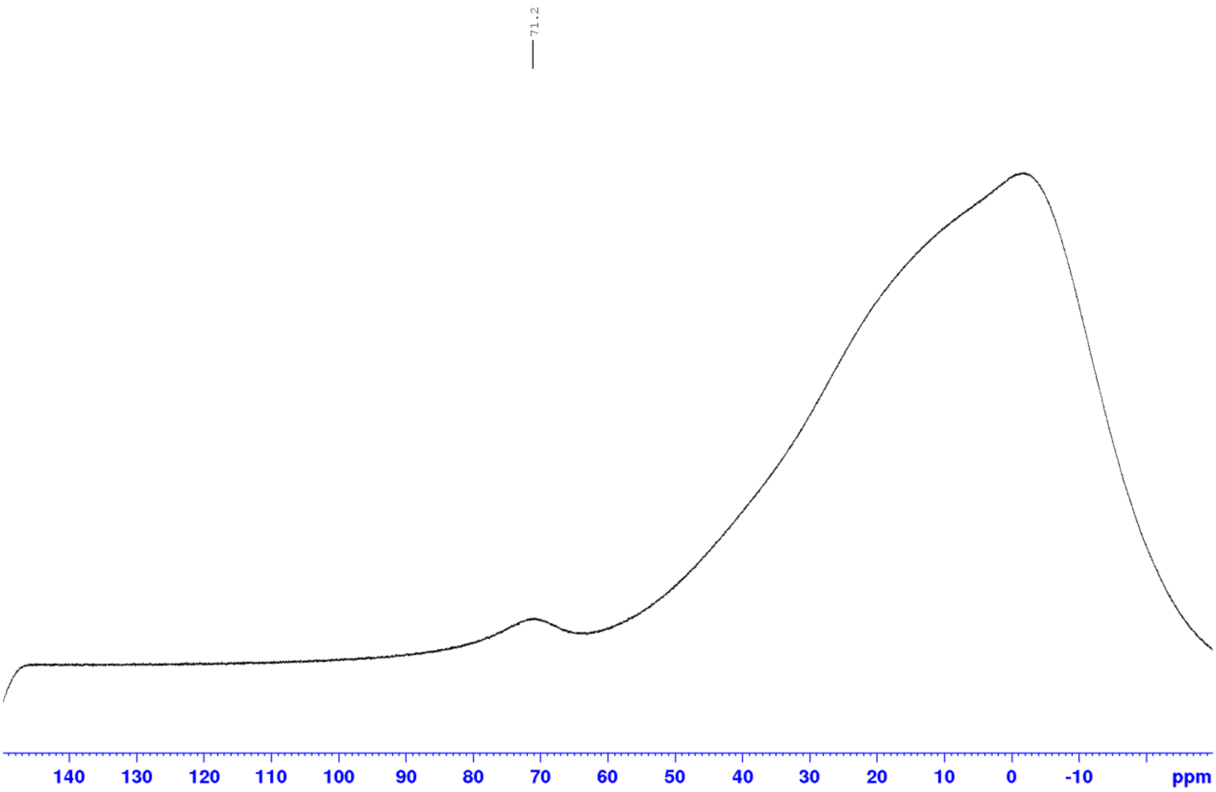

Figure S45. $^{11}B\{^1H\}$ NMR spectrum (160 MHz, 298 K, C_6D_6) of compound **3**.

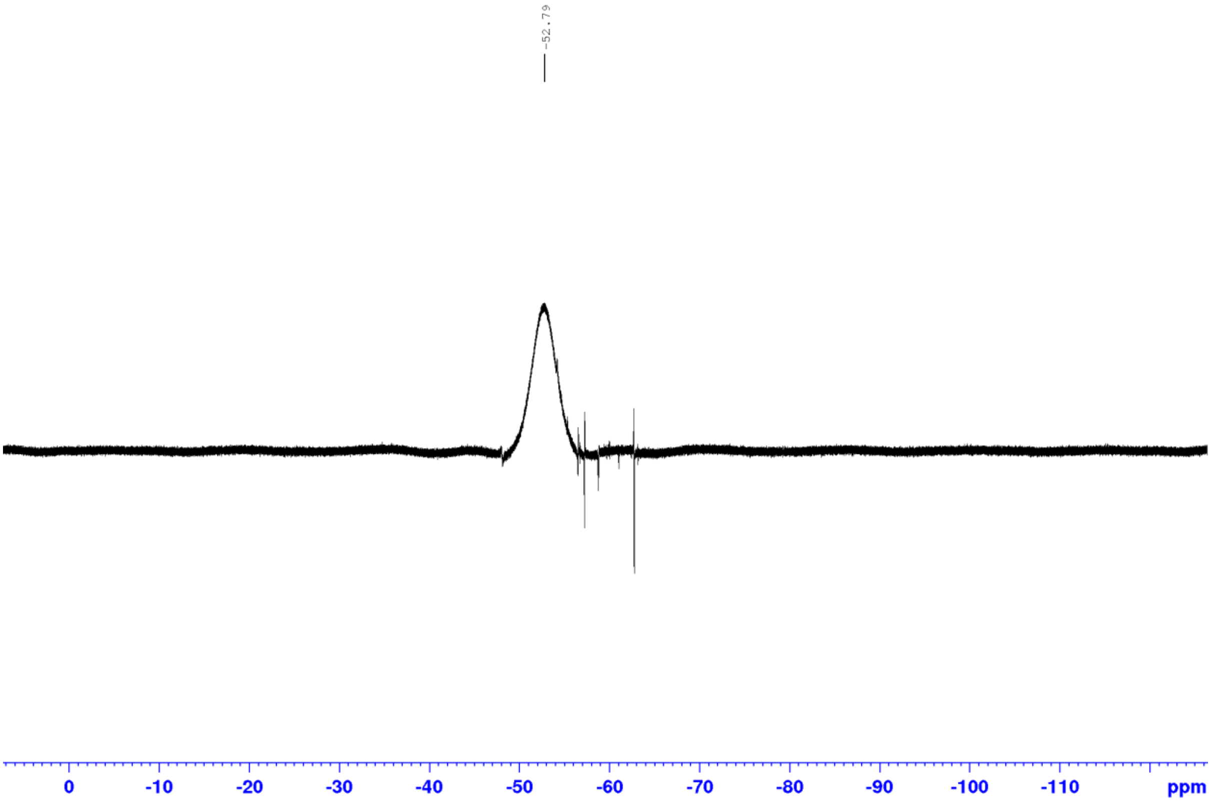

Figure S46. $^{19}F\{^1H\}$ NMR spectrum (470 MHz, 298 K, C_6D_6) of compound **3**.

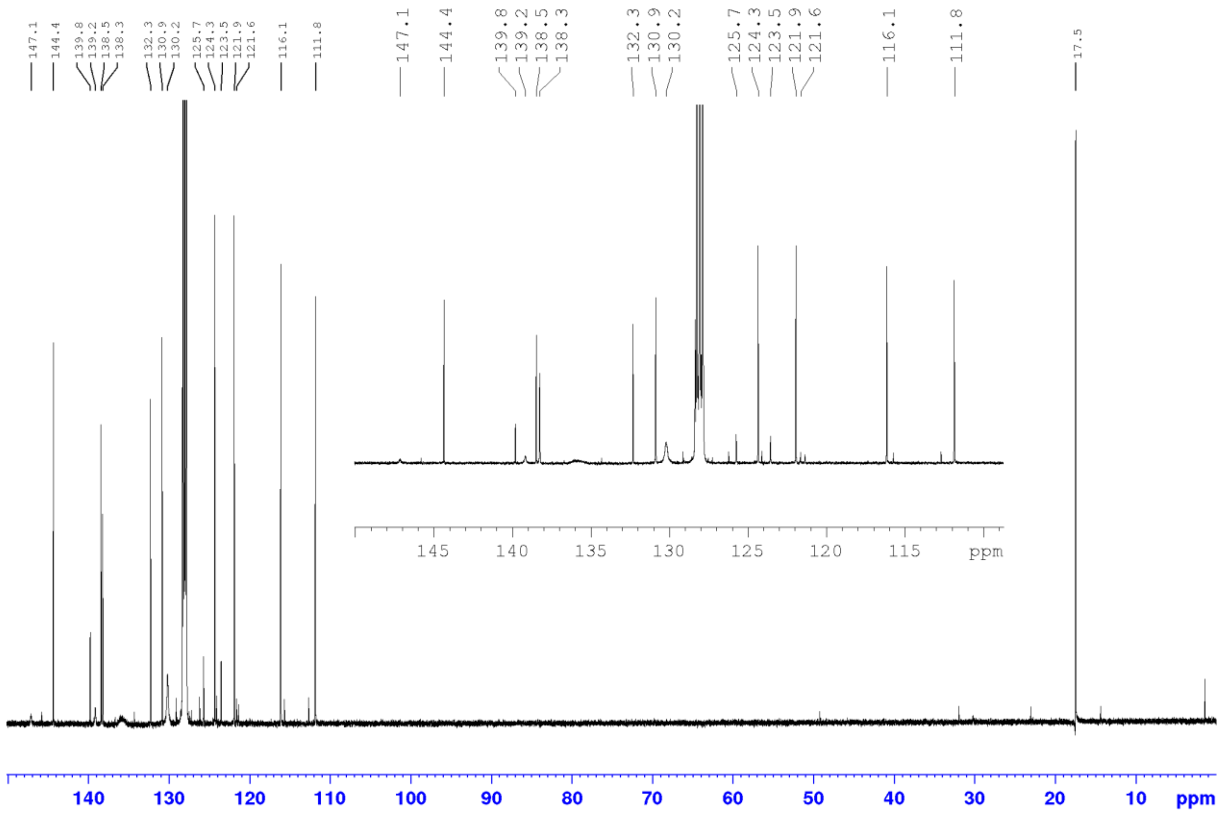

Figure S47. $^{13}C\{^{1}H\}$ NMR spectrum (126 MHz, 298 K, C_6D_6) of compound **3**.

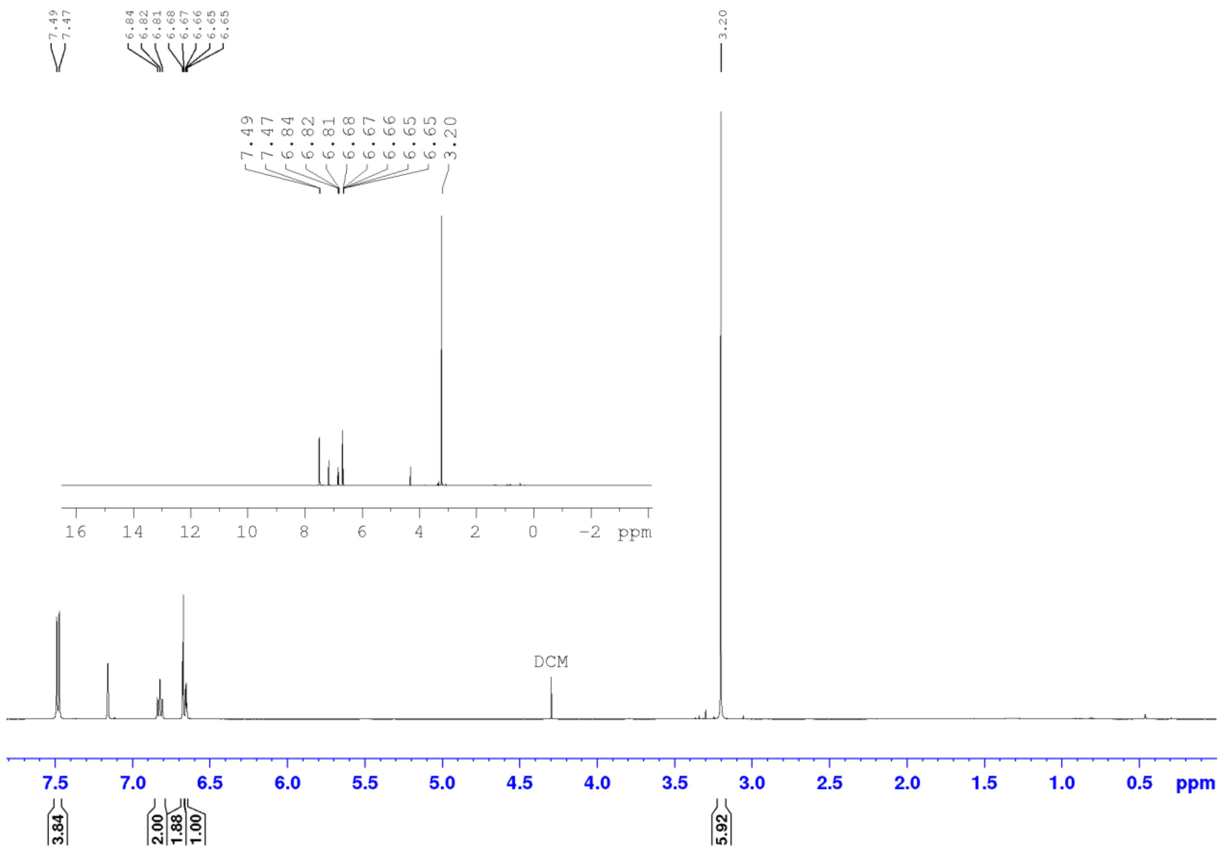

Figure S48. ^{1}H NMR spectrum (500 MHz, 298 K, C_6D_6) of compound **iv'**.

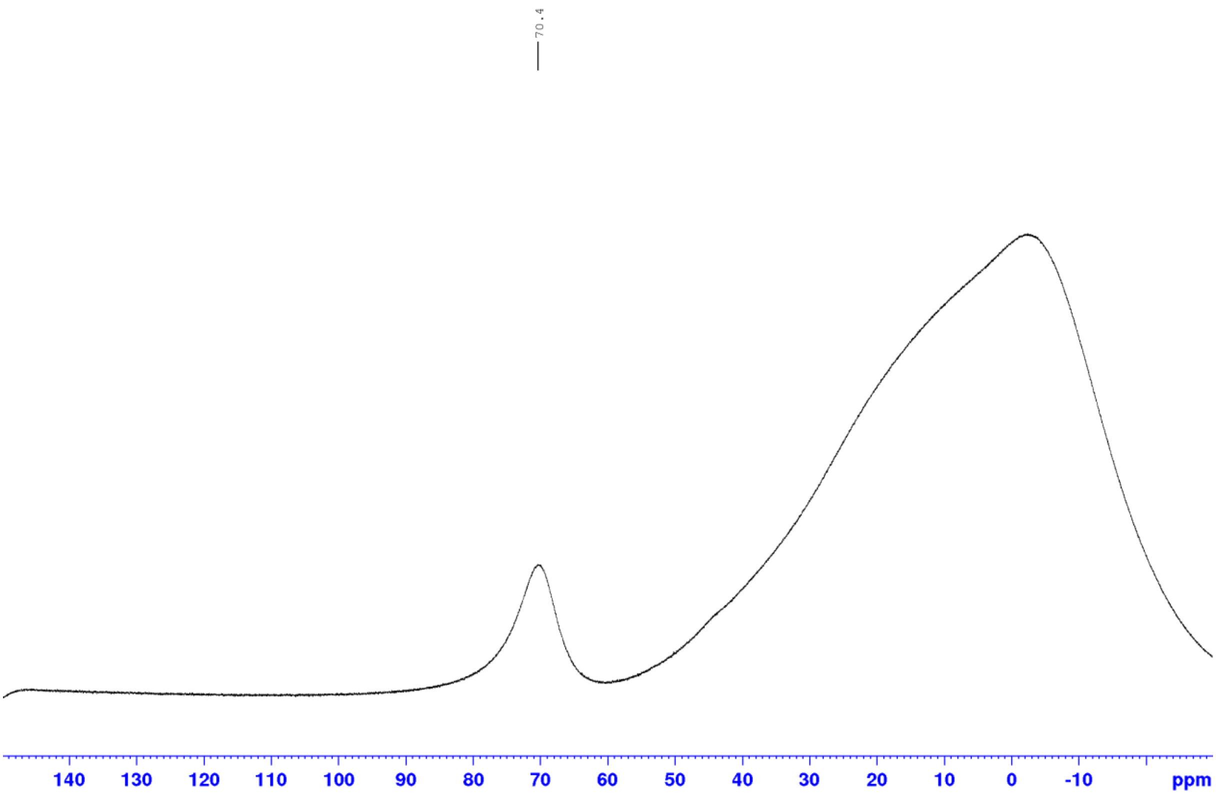

Figure S49. $^{11}B\{^{1}H\}$ NMR spectrum (160 MHz, 298 K, C_6D_6) of compound **iv'**.

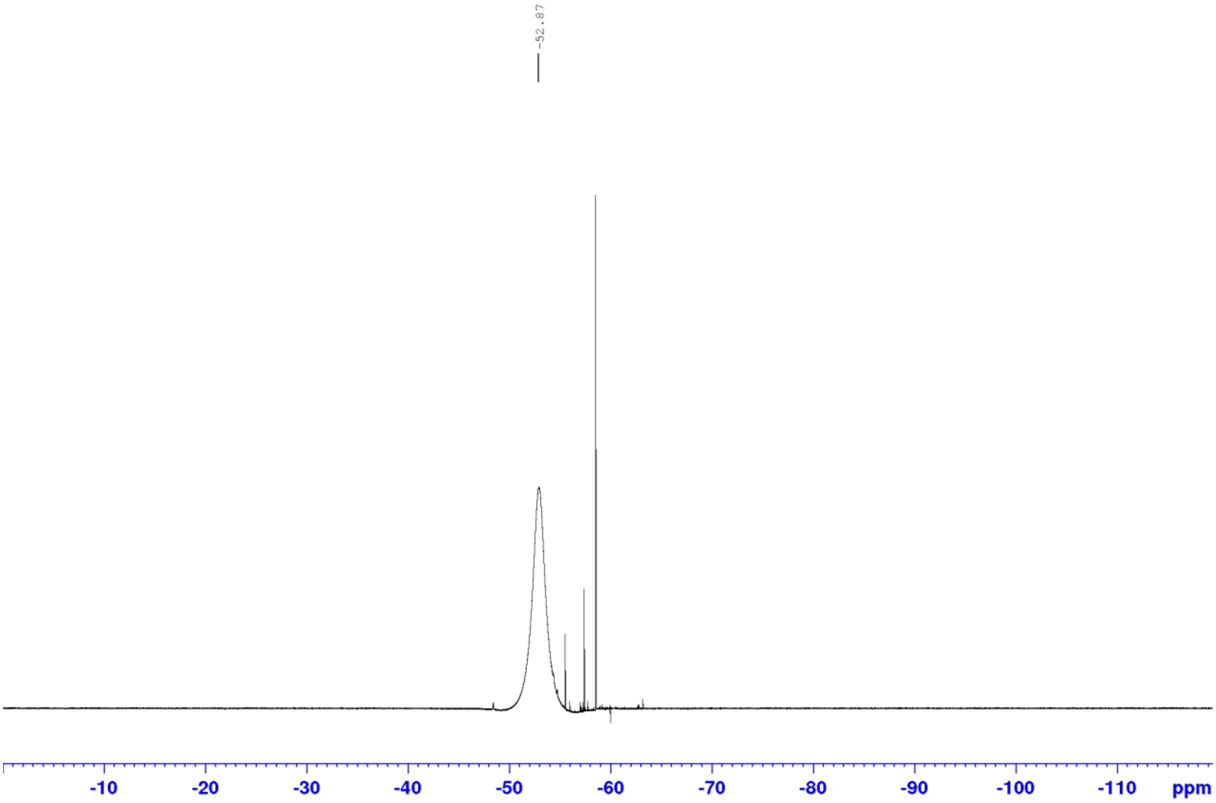

Figure S50. $^{19}F\{^{1}H\}$ NMR spectrum (470 MHz, 298 K, C_6D_6) of compound **iv'**.

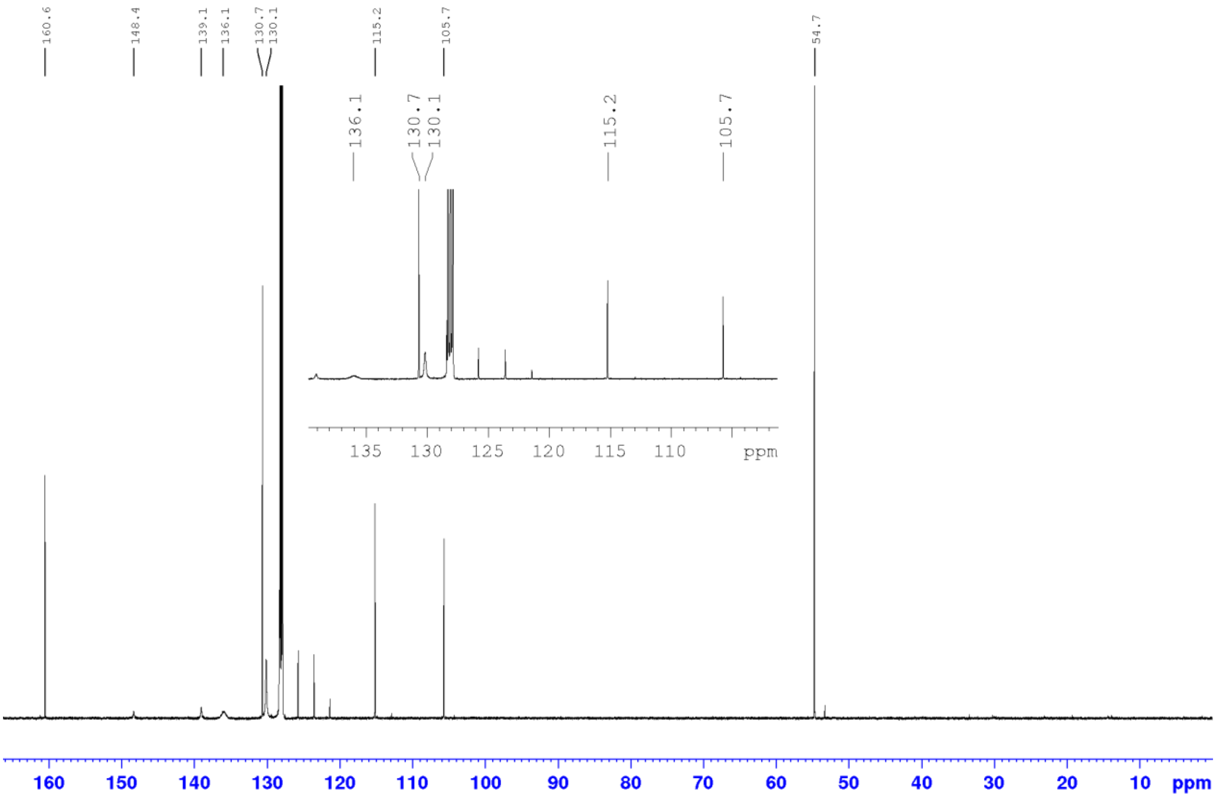

Figure S51. $^{13}C\{^1H\}$ NMR spectrum (126 MHz, 298 K, C_6D_6) of compound **iv’**.

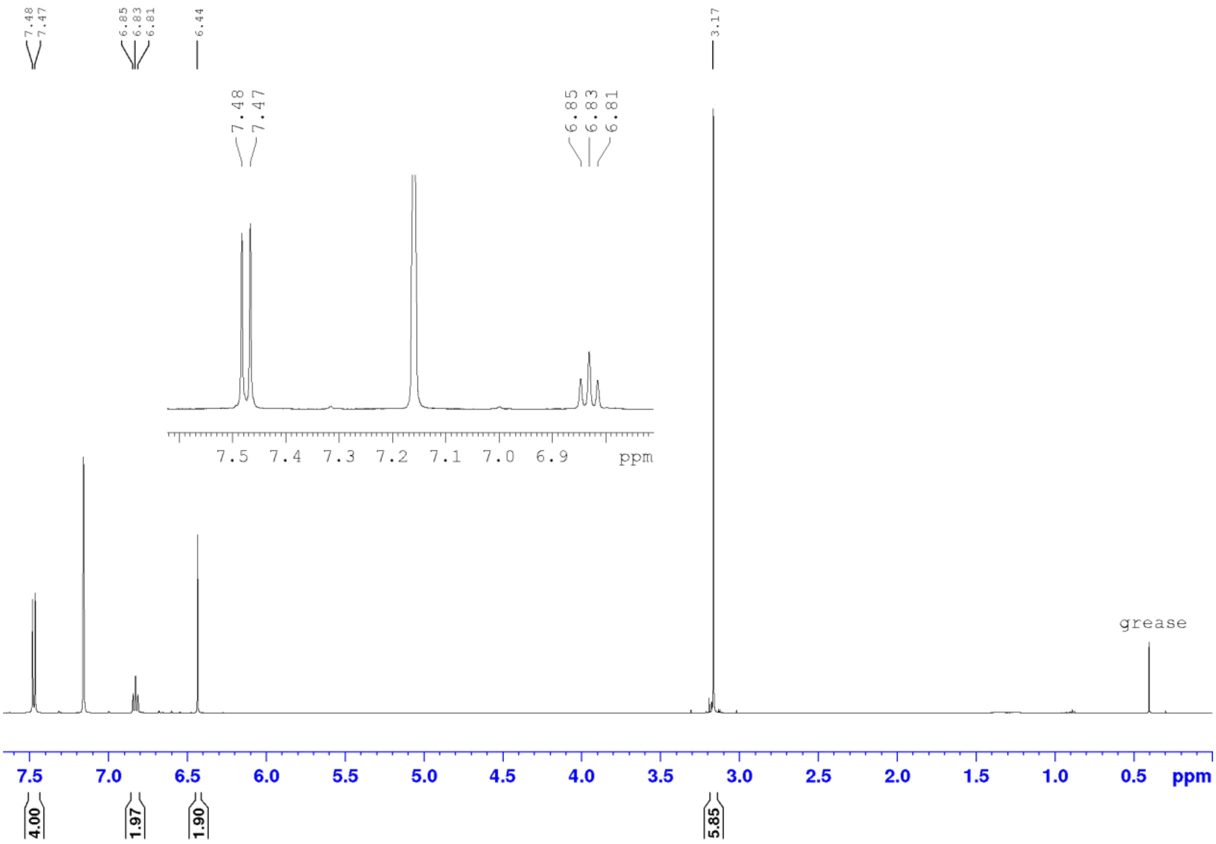

Figure S52. ^1H NMR spectrum (500 MHz, 298 K, C_6D_6) of compound **iv**.

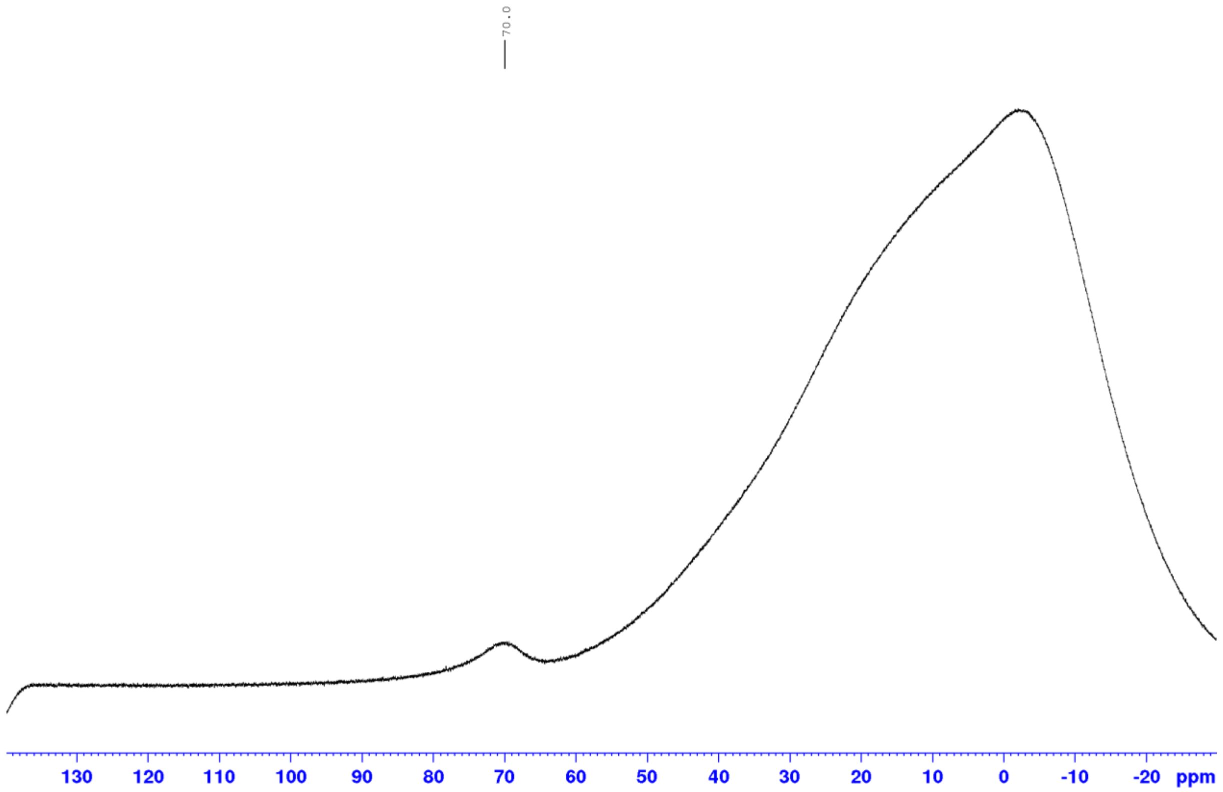

Figure S53. $^{11}B\{^{1}H\}$ NMR spectrum (160 MHz, 298 K, C_6D_6) of compound **iv**.

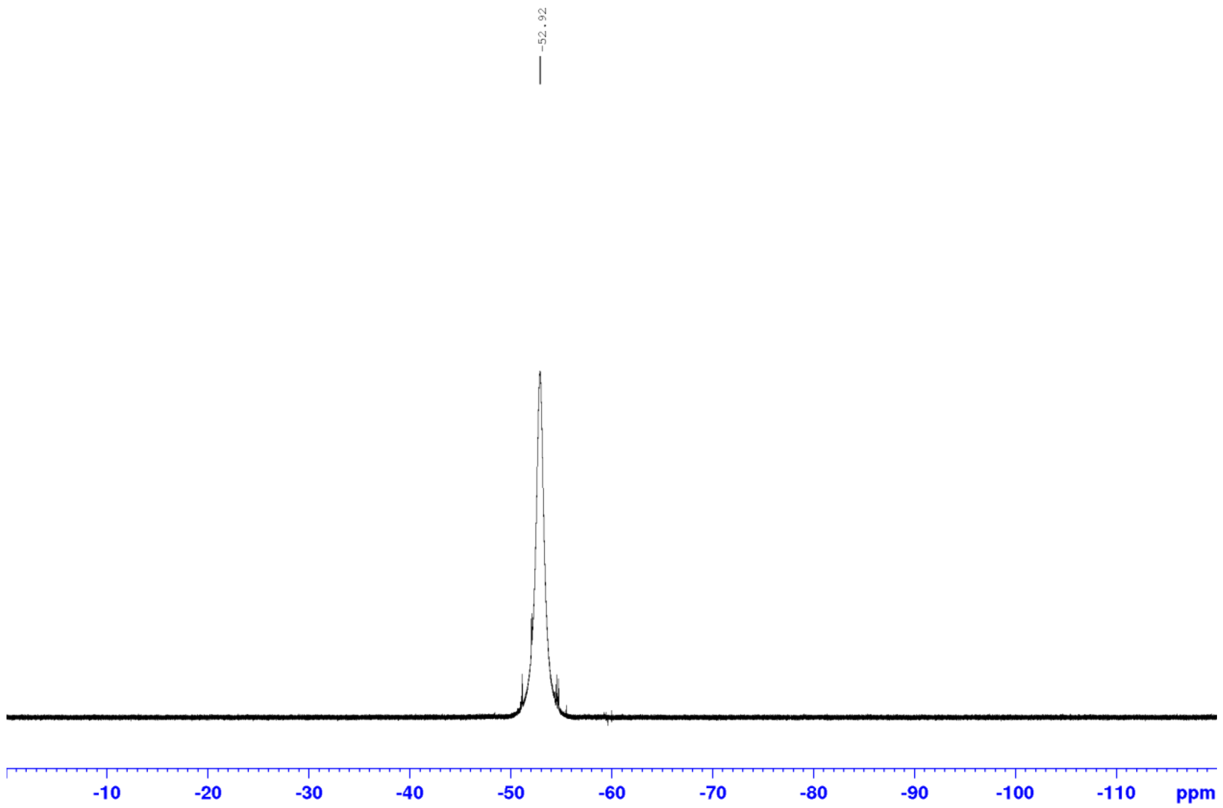

Figure S54. $^{19}F\{^{1}H\}$ NMR spectrum (470 MHz, 298 K, C_6D_6) of compound **iv**.

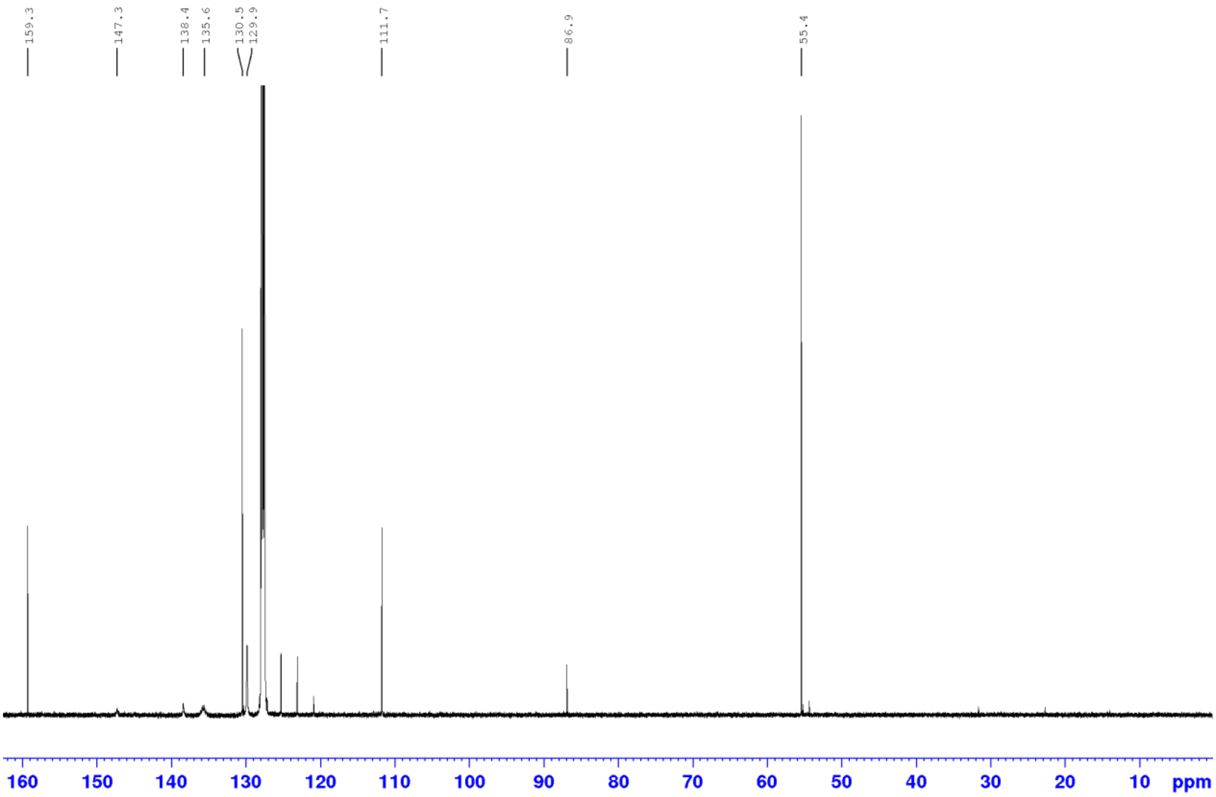

Figure S55. $^{13}C\{^{1}H\}$ NMR spectrum (126 MHz, 298 K, C_6D_6) of compound **iv**.

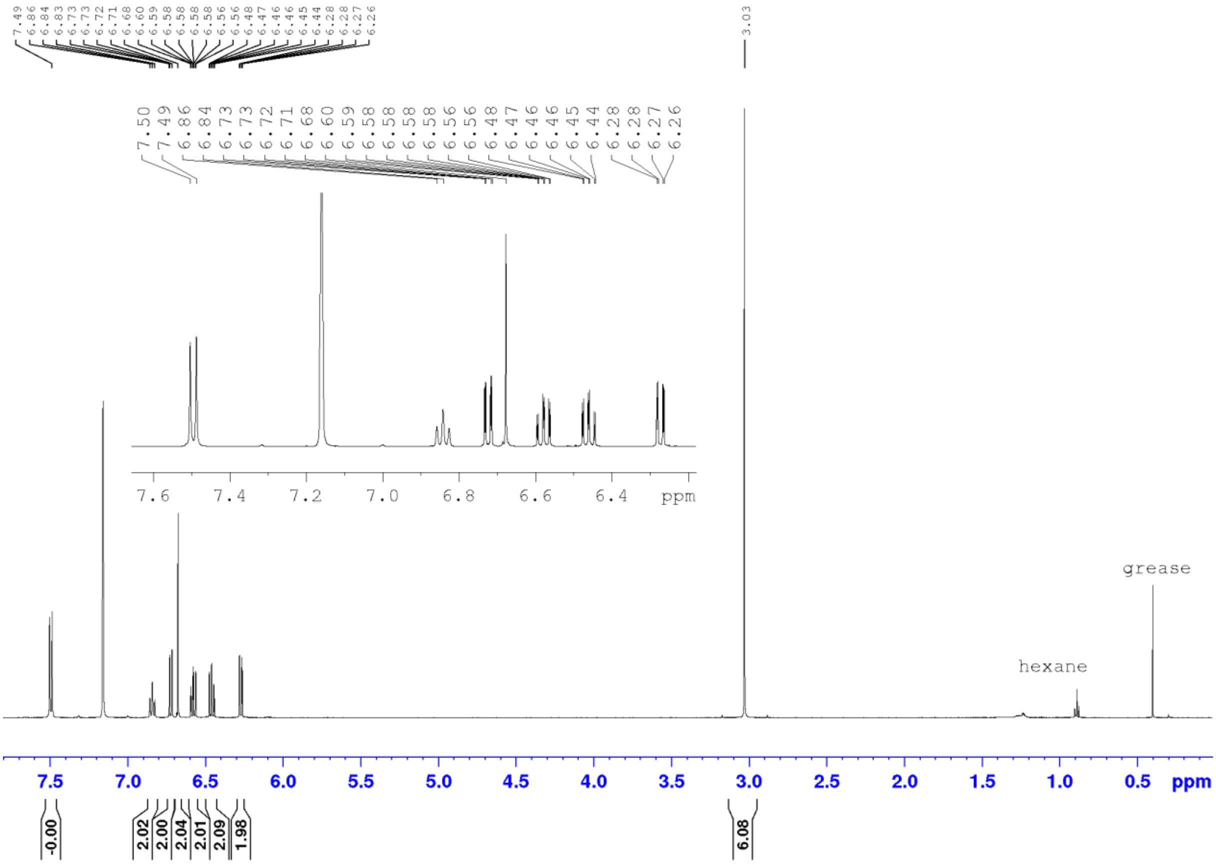

Figure S56. ^{1}H NMR spectrum (500 MHz, 298 K, C_6D_6) of compound **4**.

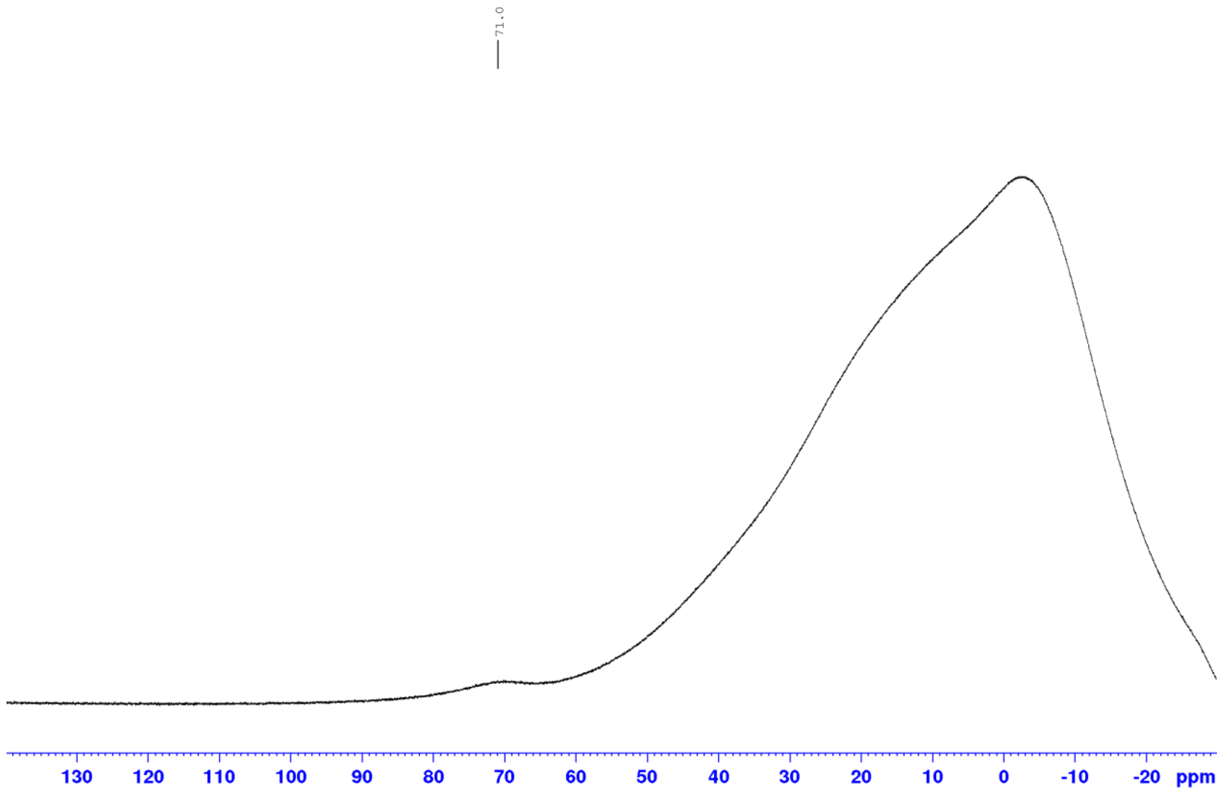

Figure S57. $^{11}B\{^1H\}$ NMR spectrum (160 MHz, 298 K, C_6D_6) of compound **4.**

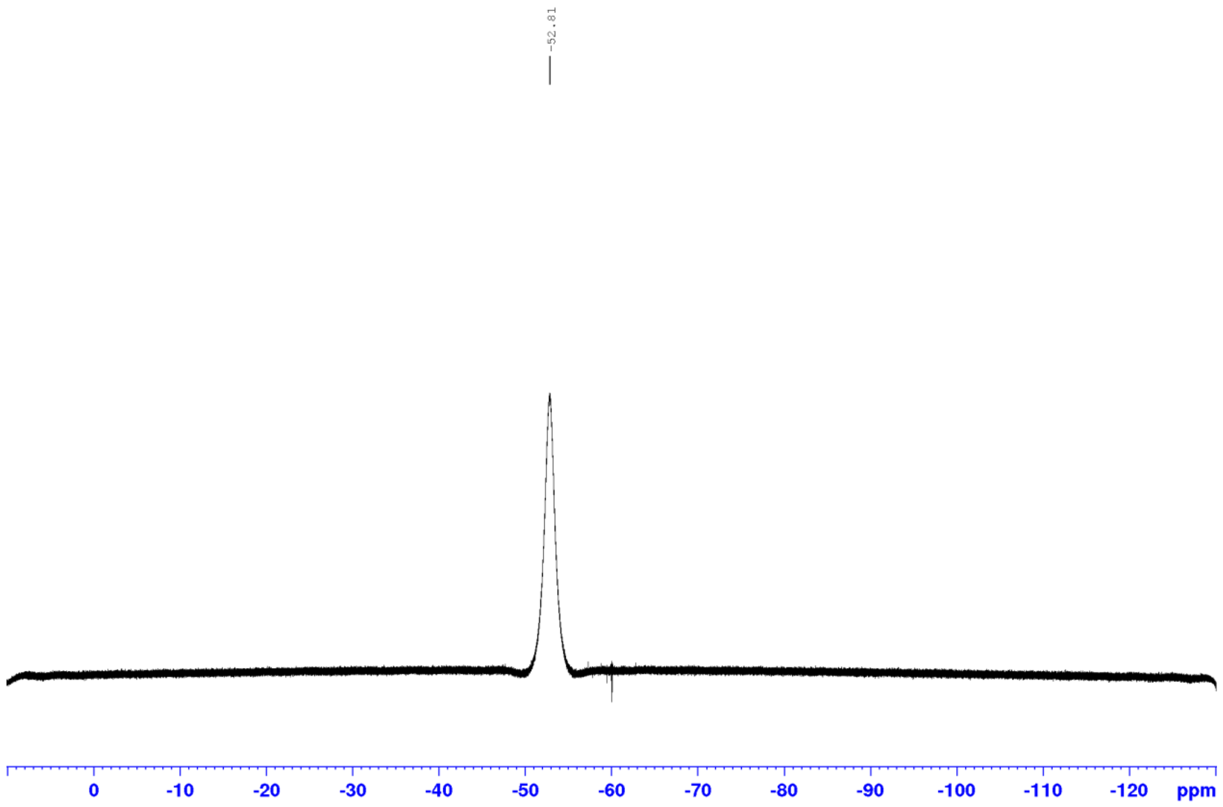

Figure S58. $^{19}F\{^1H\}$ NMR spectrum (470 MHz, 298 K, C_6D_6) of compound **4**.

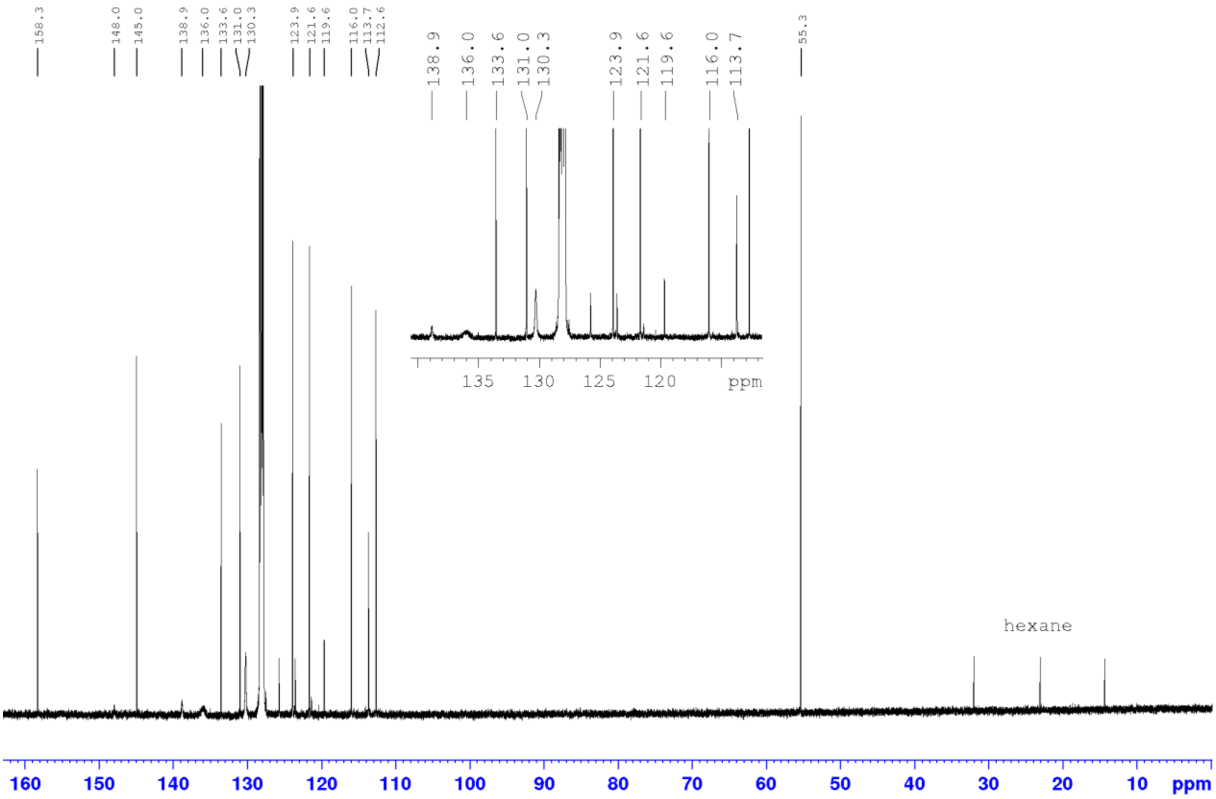

Figure S59. $^{13}C\{^{1}H\}$ NMR spectrum (126 MHz, 298 K, C_6D_6) of compound **4**.

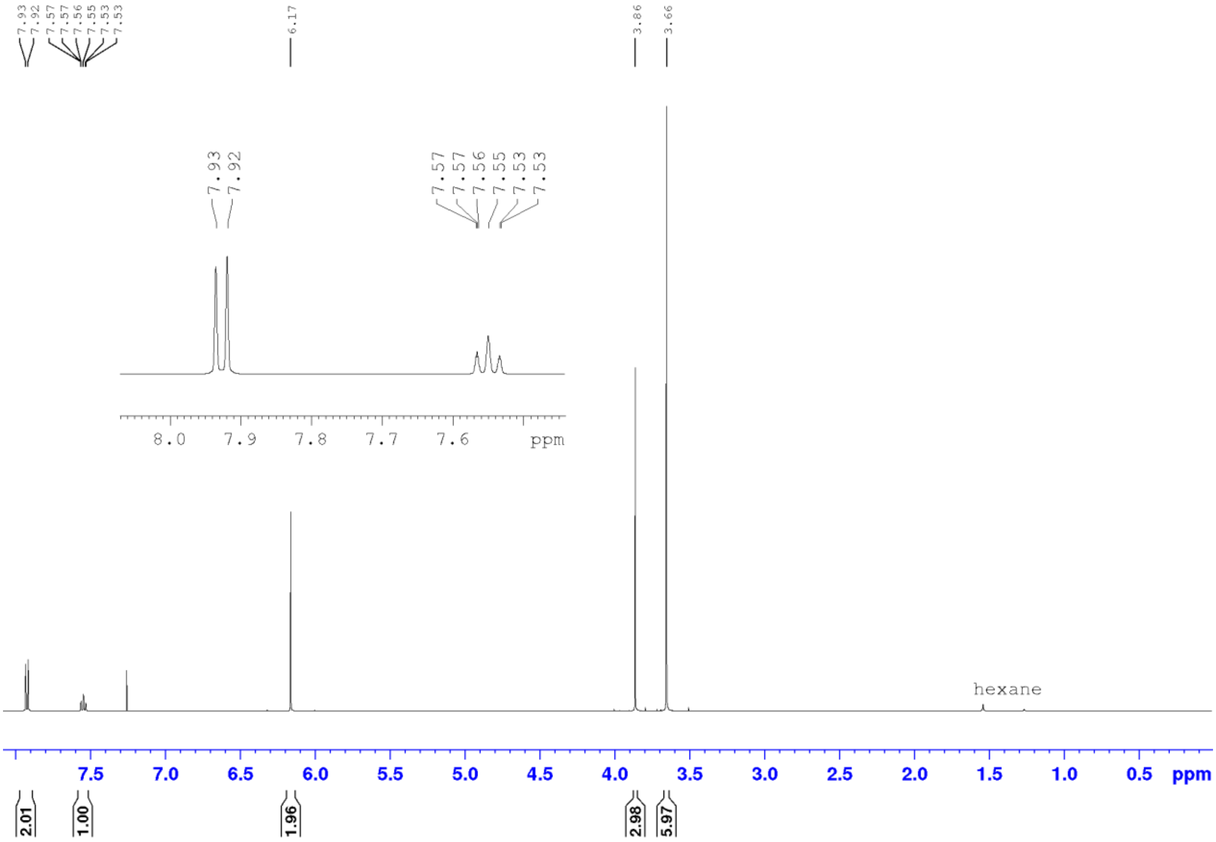

Figure S60. ^{1}H NMR spectrum (500 MHz, 298 K, $CDCl_3$) of **2,4,6-trimethoxy-2,6-bis(trifluoromethyl)-1,1'-biphenyl**.

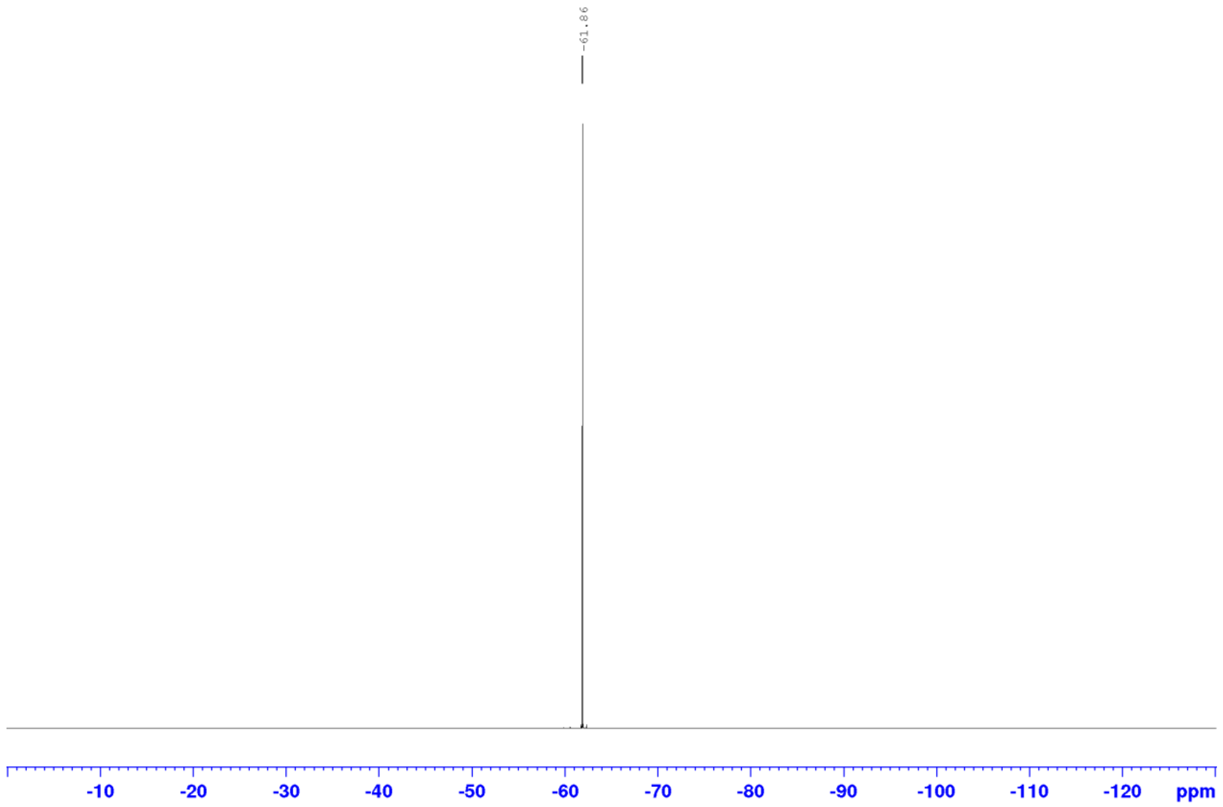

Figure S61. $^{19}F\{^1H\}$ NMR spectrum (470 MHz, 298 K, $CDCl_3$) of **2,4,6-trimethoxy-2,6-bis(trifluoromethyl)-1,1'-biphenyl**.

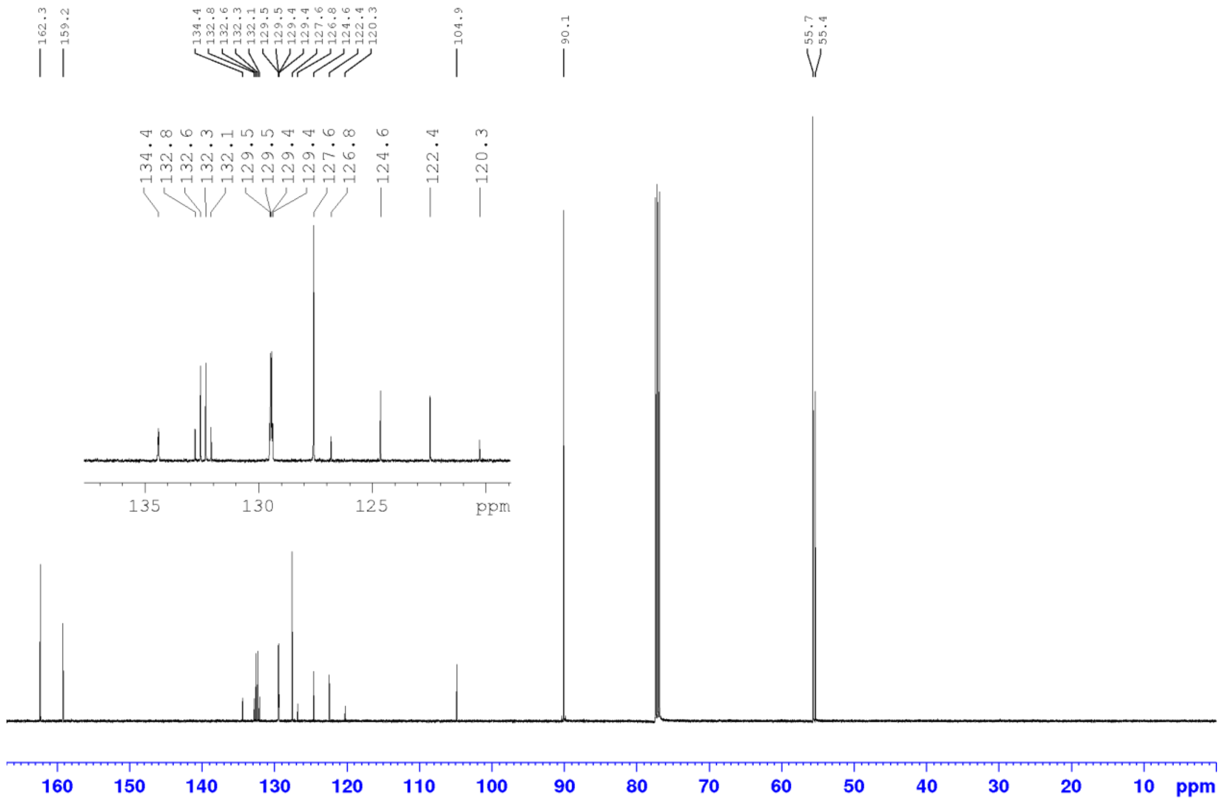

Figure S62. $^{13}C\{^1H\}$ NMR spectrum (126 MHz, 298 K, $CDCl_3$) of **2,4,6-trimethoxy-2,6-bis(trifluoromethyl)-1,1'-biphenyl**.

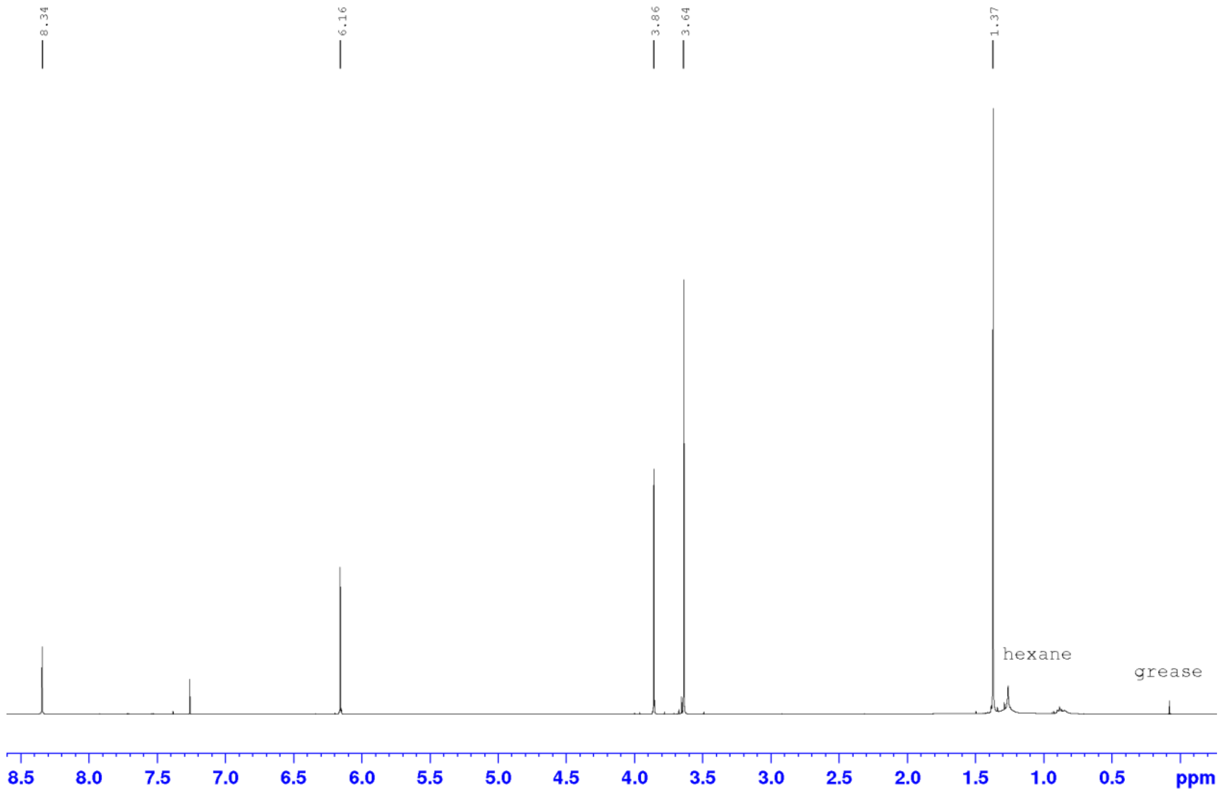

Figure S63. ^{1}H NMR spectrum (500 MHz, 298 K, $CDCl_3$) of **4,4,5,5-tetramethyl-2-(2',4',6'-trimethoxy-2,6-bis(trifluoromethyl)-[1,1'-biphenyl]-4-yl)-1,3,2-dioxaborolane**.

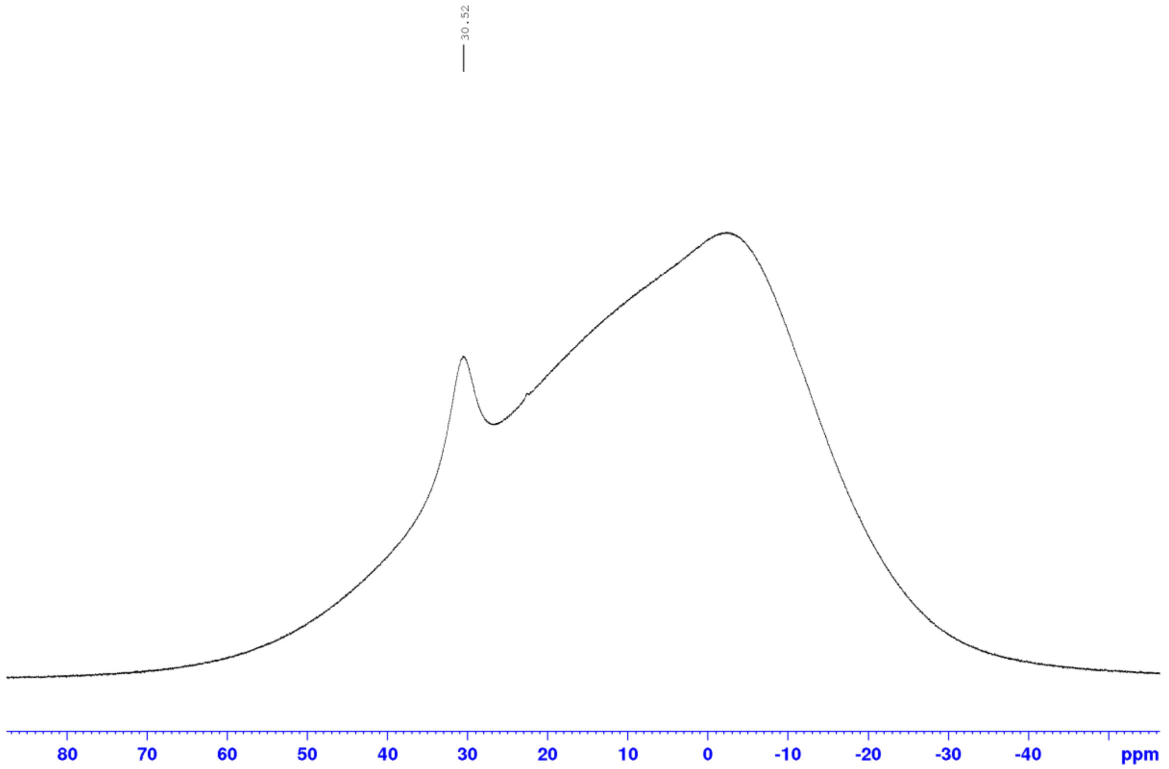

Figure S64. $^{11}B\{^{1}H\}$ NMR spectrum (160 MHz, 298 K, $CDCl_3$) of **4,4,5,5-tetramethyl-2-(2',4',6'-trimethoxy-2,6-bis(trifluoromethyl)-[1,1'-biphenyl]-4-yl)-1,3,2-dioxaborolane.**

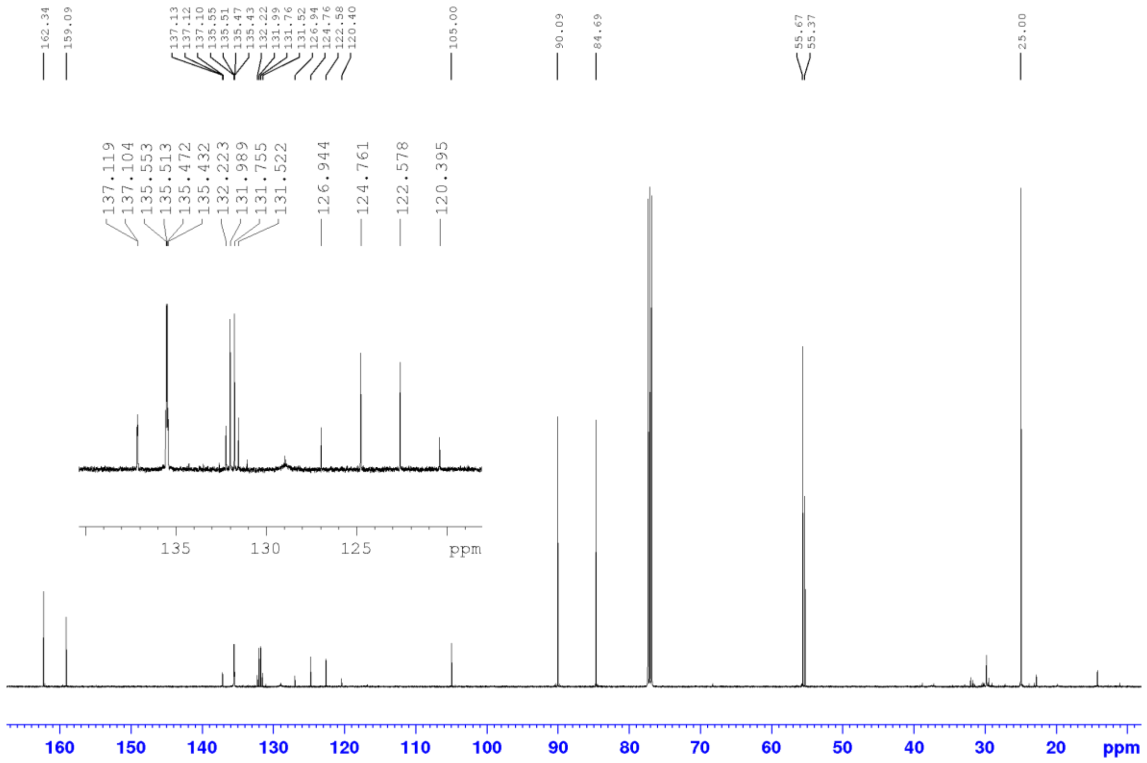

Figure S65. $^{13}C\{^{1}H\}$ NMR spectrum (126 MHz, 298 K, $CDCl_3$) of **4,4,5,5-tetramethyl-2-(2',4',6'-trimethoxy-2,6-bis(trifluoromethyl)-[1,1'-biphenyl]-4-yl)-1,3,2-dioxaborolane**.

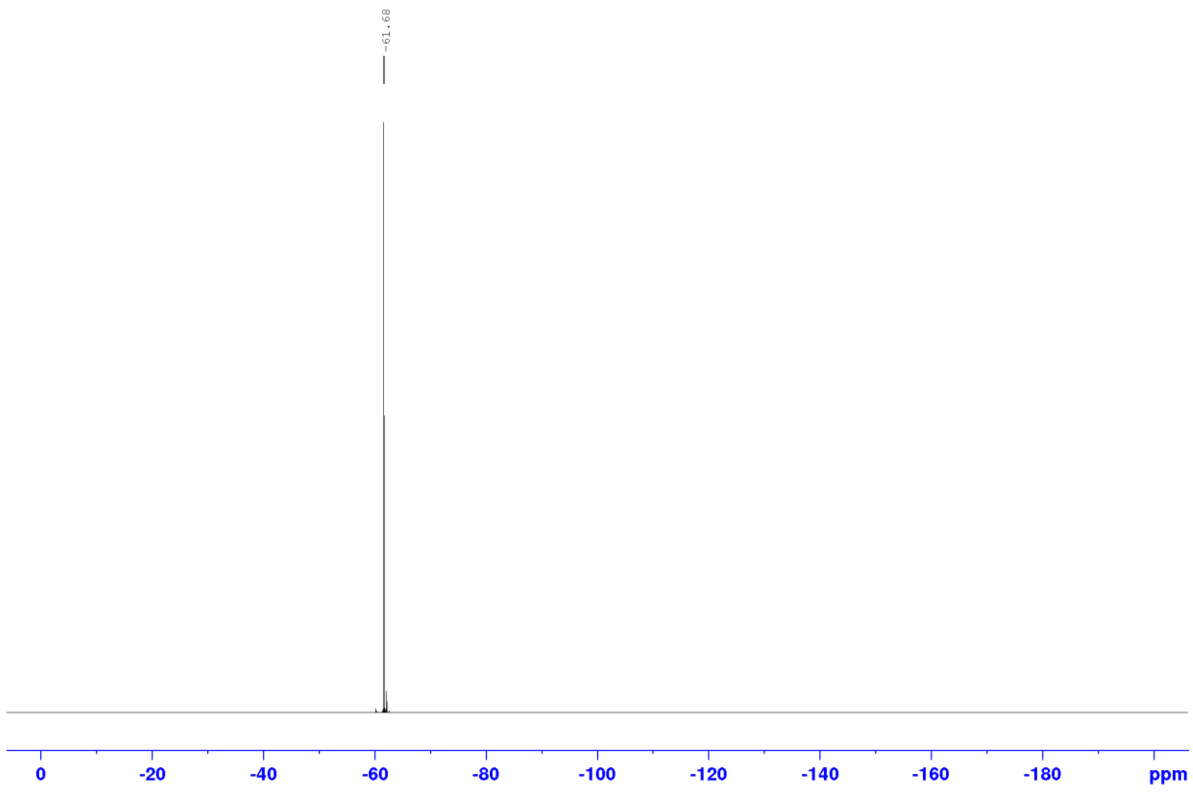

Figure S66. $^{19}F\{^{1}H\}$ NMR spectrum (470 MHz, 298 K, $CDCl_3$) of **4,4,5,5-tetramethyl-2-(2',4',6'-trimethoxy-2,6-bis(trifluoromethyl)-[1,1'-biphenyl]-4-yl)-1,3,2-dioxaborolane**.

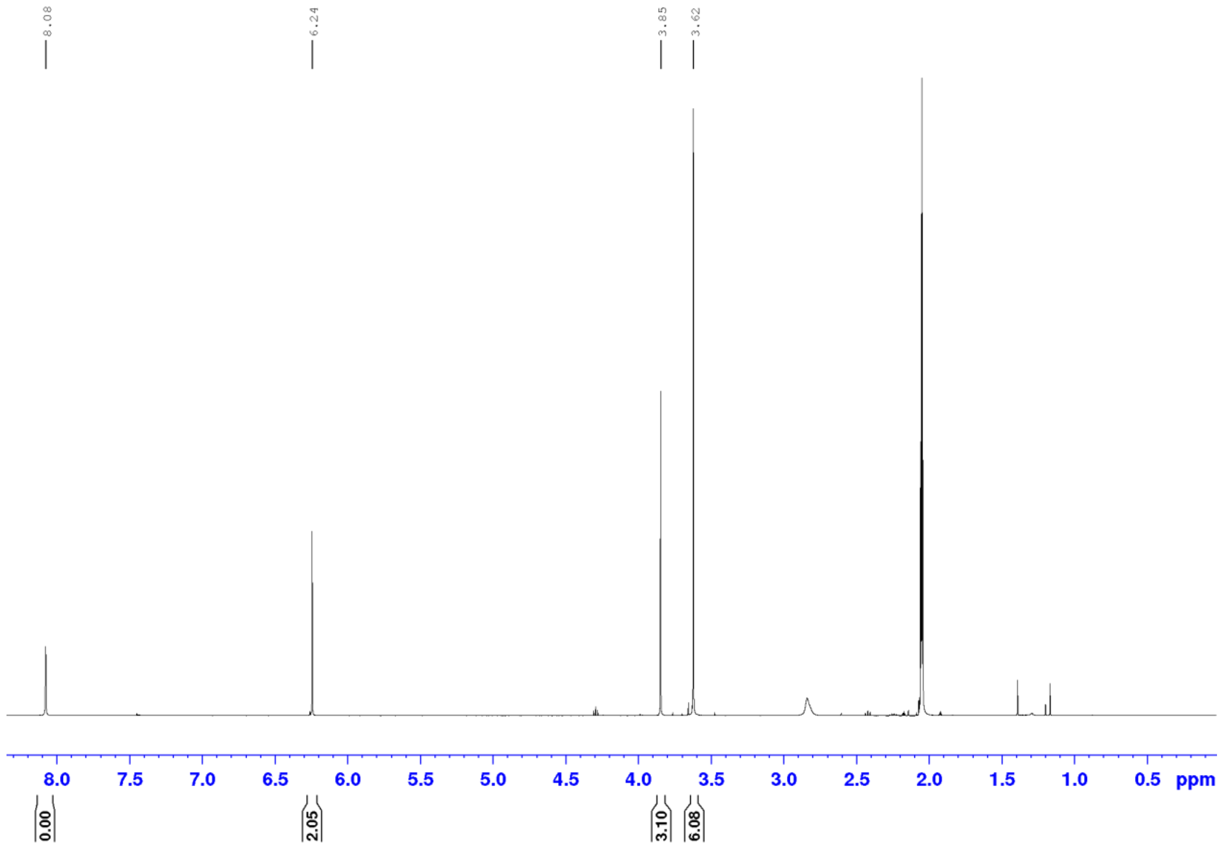

Figure S67. ^{1}H NMR spectrum (500 MHz, 298 K, acetone-d_6) of **potassium(2',4',6'-trimethoxy-2,6-bis(trifluoromethyl)-[1,1'-biphenyl]-4-yl)trifluoroborate**.

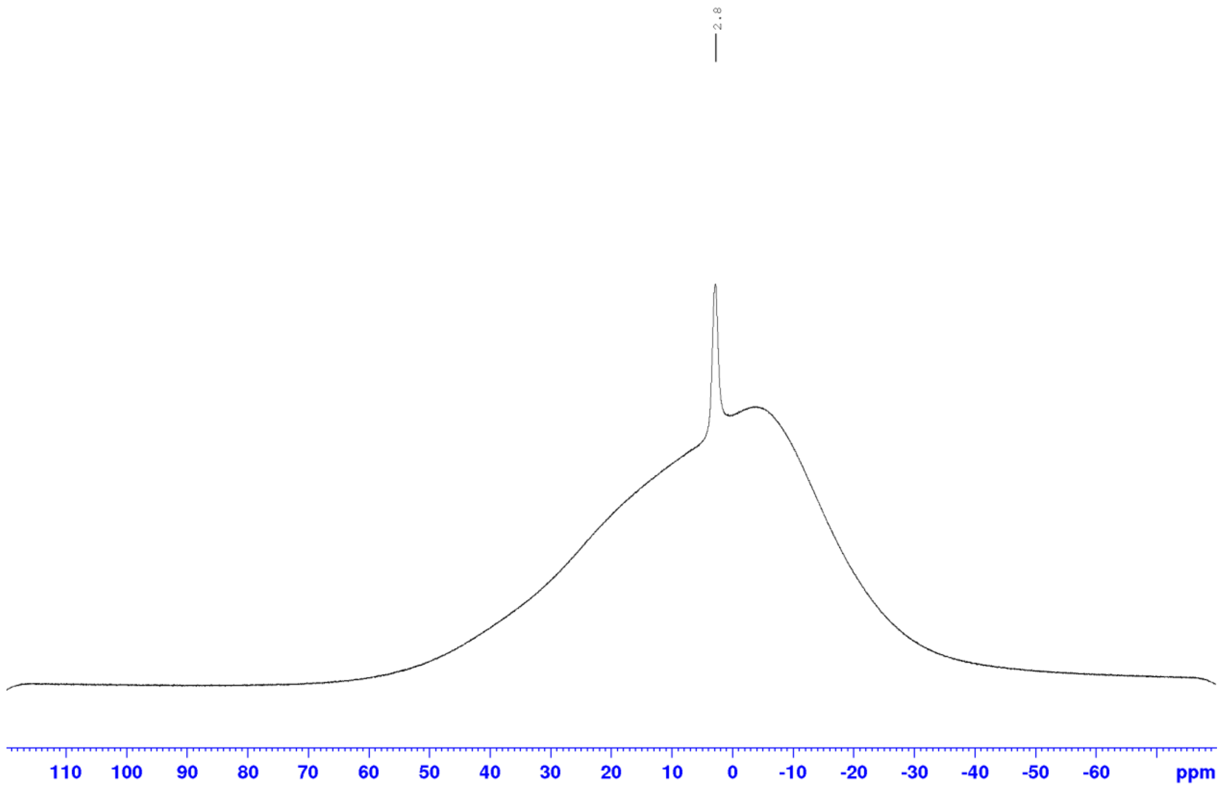

Figure S68. ^{11}B{^{1}H} NMR spectrum (160 MHz, 298 K, acetone-d_6) of **potassium(2',4',6'-trimethoxy-2,6-bis(trifluoromethyl)-[1,1'-biphenyl]-4-yl)trifluoroborate**.

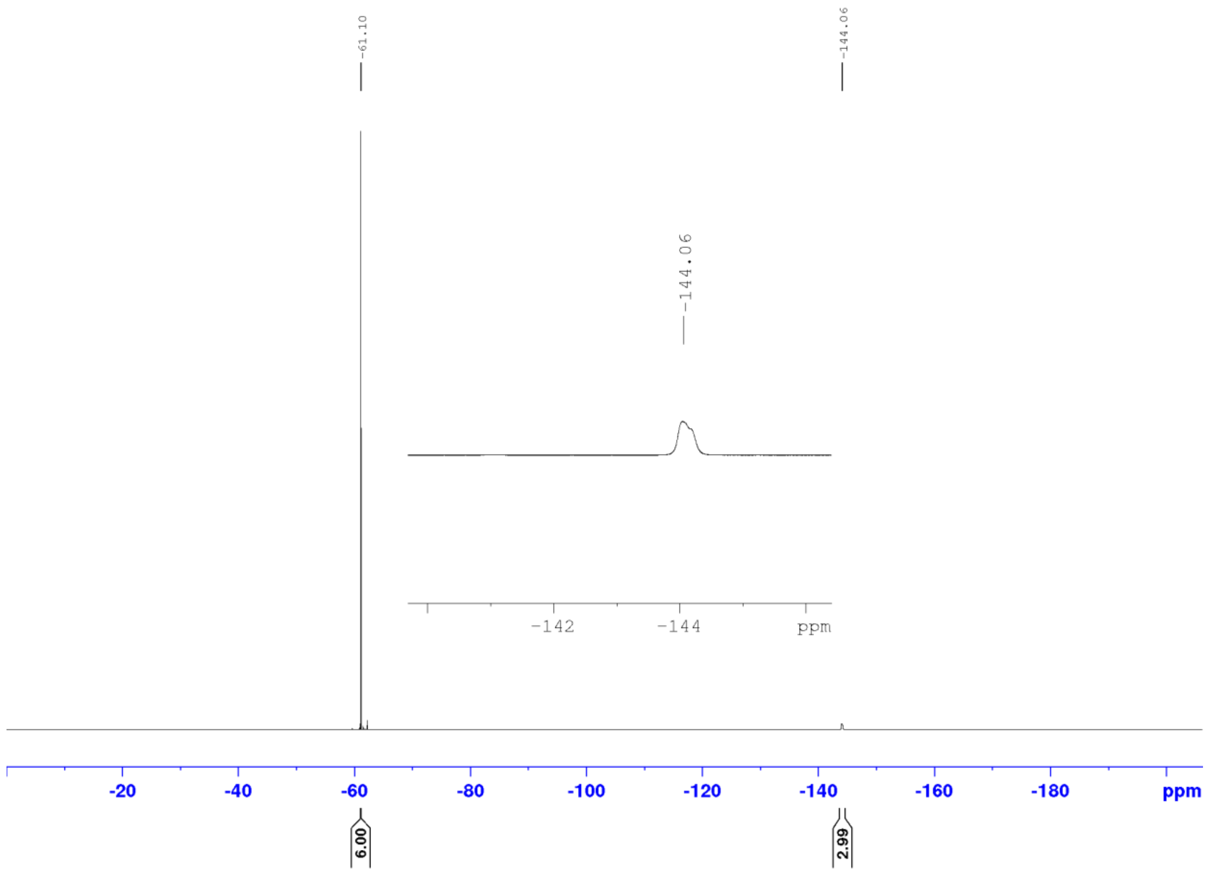

Figure S69. $^{19}F\{^{1}H\}$ NMR spectrum (470 MHz, 298 K, acetone-d_6) of **potassium(2',4',6'-trimethoxy-2,6-bis(trifluoromethyl)-[1,1'-biphenyl]-4-yl)trifluoroborate**.

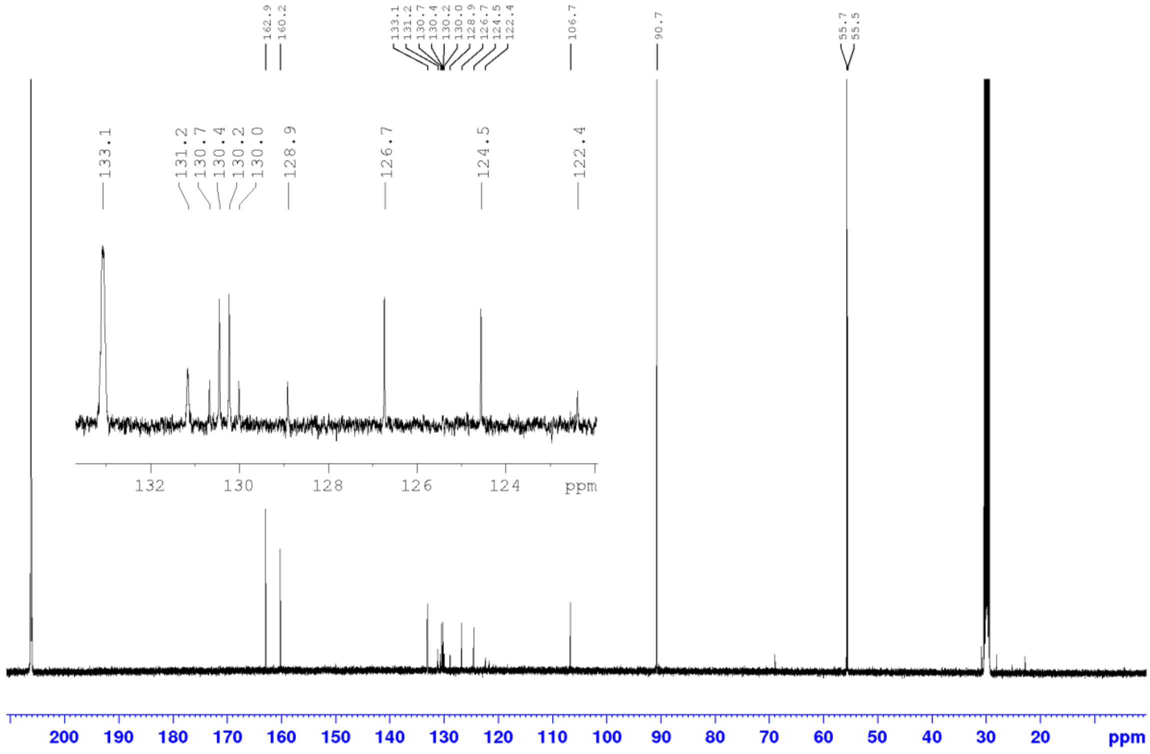

Figure S70. $^{13}C\{^{1}H\}$ NMR spectrum (126 MHz, 298 K, acetone-d_6) of **potassium(2',4',6'-trimethoxy-2,6-bis(trifluoromethyl)-[1,1'-biphenyl]-4-yl)trifluoroborate**.

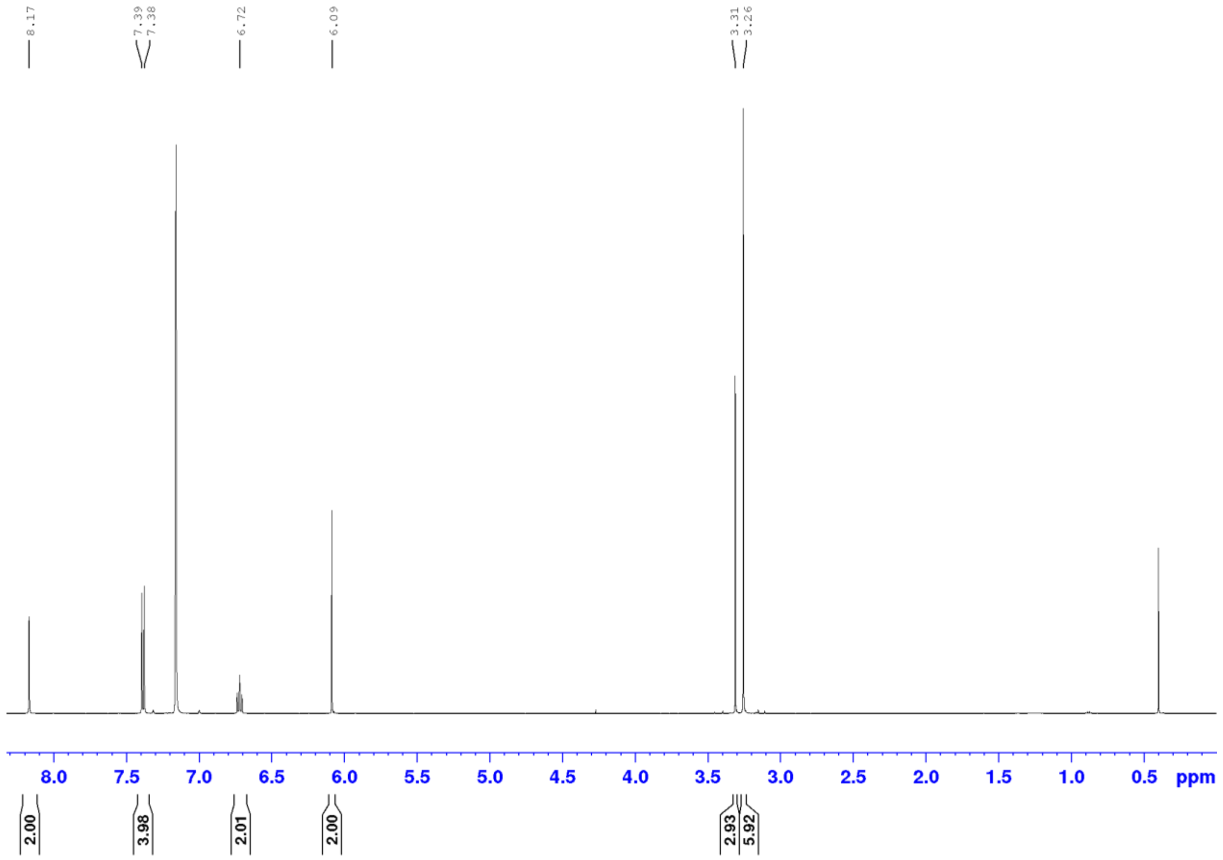

Figure S71. ^{1}H NMR spectrum (500 MHz, 298 K, C_6D_6) of compound **5**.

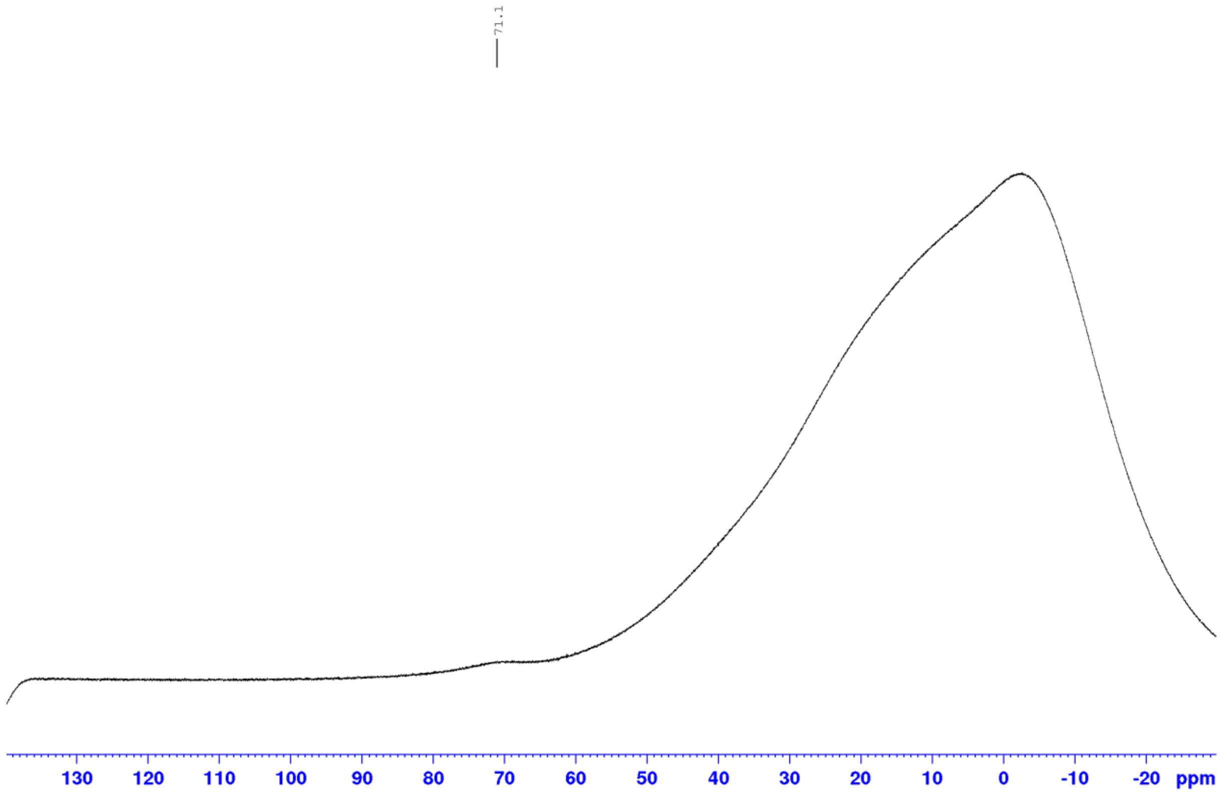

Figure S72. $^{11}B\{^{1}H\}$ NMR spectrum (160 MHz, 298 K, C_6D_6) of compound **5**.

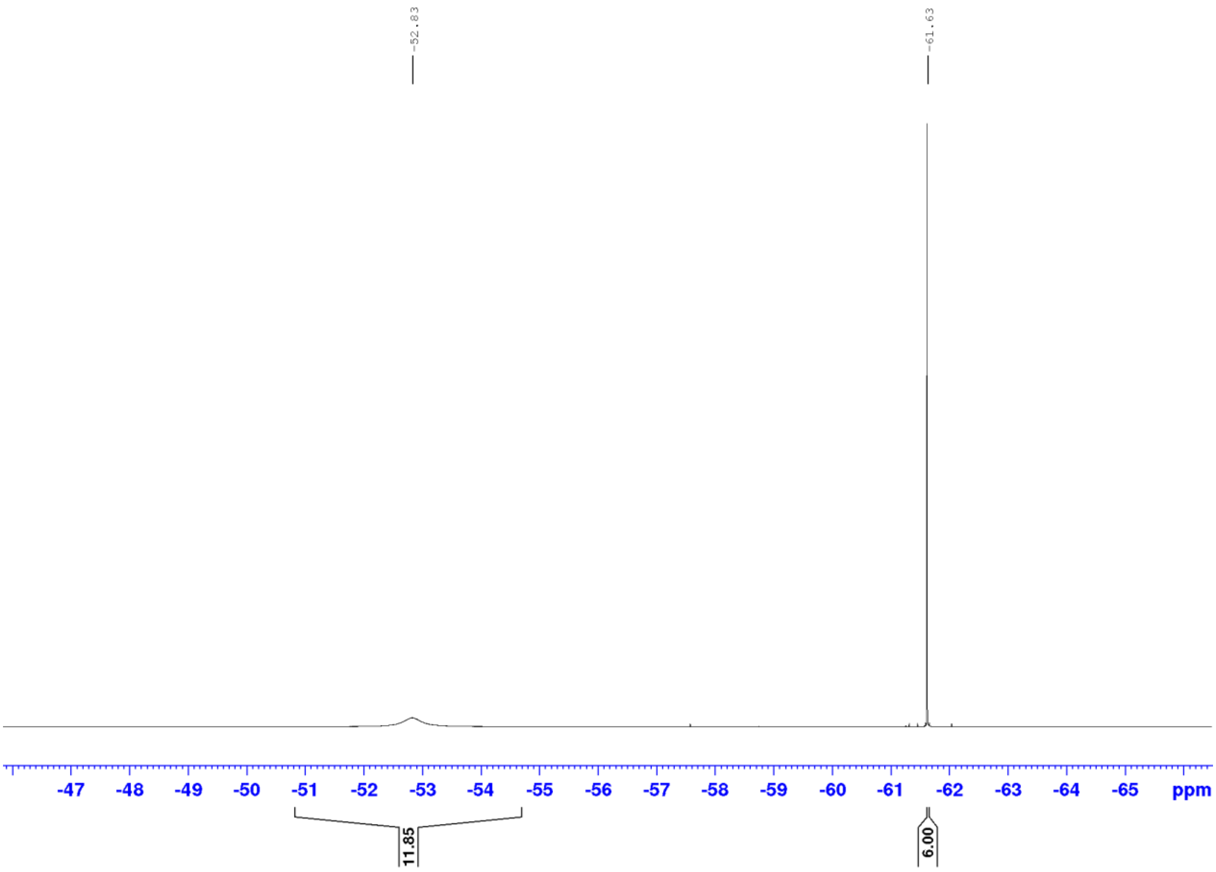

Figure S73. ^{19}F{^{1}H} NMR spectrum (470 MHz, 298 K, C_6D_6) of compound **5**.

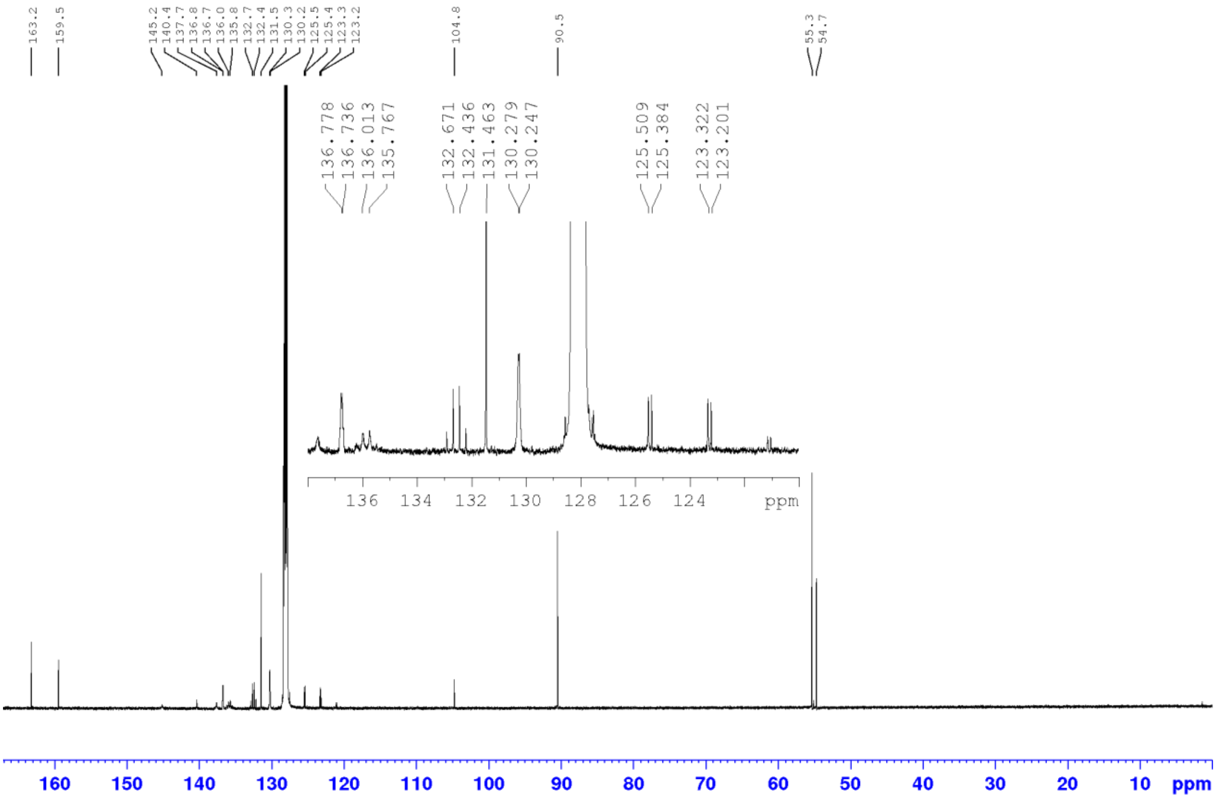

Figure S74. ^{13}C{^{1}H} NMR spectrum (126 MHz, 298 K, C_6D_6) of compound **5**.

Photophysical data

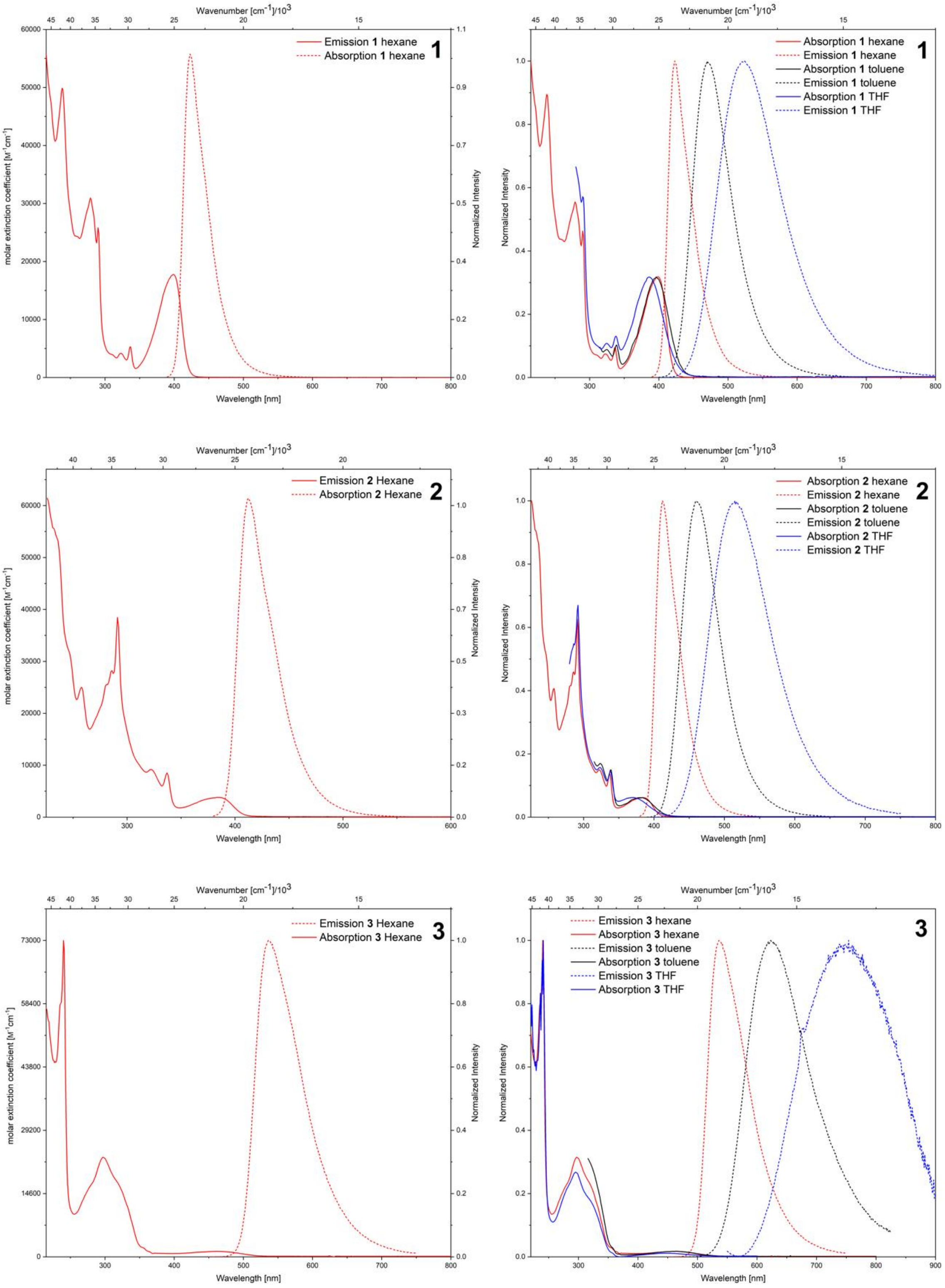

Figure S75. Absorption and emission spectra of **1** (1st row; left: hexane with extinction coefficient; right: normalized spectra in hexane, toluene and THF), **2** (2nd row; left: hexane with extinction coefficient; right: normalized spectra in hexane, toluene and THF), **3** (3rd row; left: hexane with extinction coefficient; right: normalized spectra in hexane, toluene and THF).

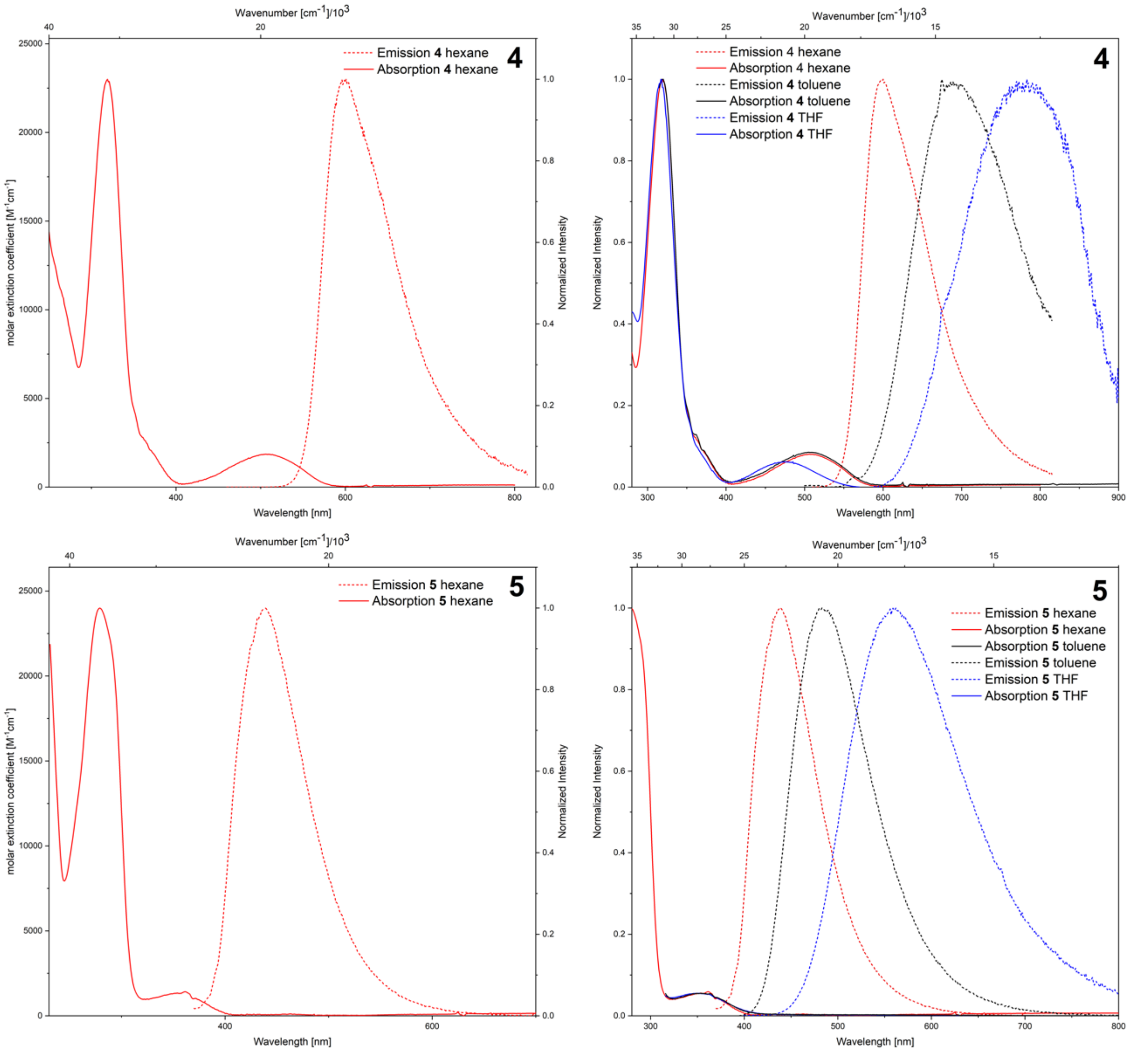

Figure S75. cont. Absorption and emission spectra of **4** (1st row; left: hexane with extinction coefficient; right: normalized spectra in hexane, toluene and THF) and **5** (2nd row; left: hexane with extinction coefficient; right: normalized spectra in hexane, toluene and THF).

Linear fit results of plotting $ln\left(\frac{B_{DF}\tau_{DF}}{B_{PF}\tau_{DF}}\right)$ vs. 1/K

Table S10. Energy gaps obtained from the fits.

Compound	Solvent	S-T gap [eV]
**3**	Toluene	0.043
**4**	Toluene	0.030
**5**	2-MeTHF	0.034

Table S11. Linear fit results of plotting $\boldsymbol{ln\left(\frac{B_{DF}\tau_{DF}}{B_{PF}\tau_{DF}}\right)}$ vs. 1/K.

		**Intercept**		**Slope**		**Statistics**
**Compound**	**Solvent**	**Value**	**Standard Error**	**Value**	**Standard Error**	**Adj. R-Square**
**3**	Toluene	2.20893	0.00946	-496.36761	2.5967	0.99975
**4**	Toluene	-1.04653	0.02534	-346.68245	6.9631	0.99718
**5**	2-MeTHF	0.27125	0.1051	-396.48542	20.29432	0.96698

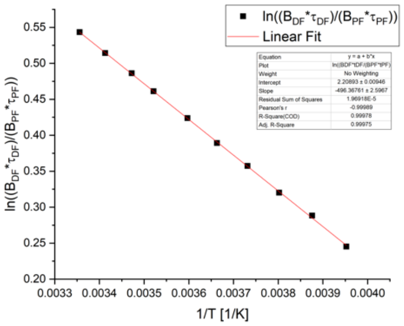

Figure S76. Linear fit of **3.**

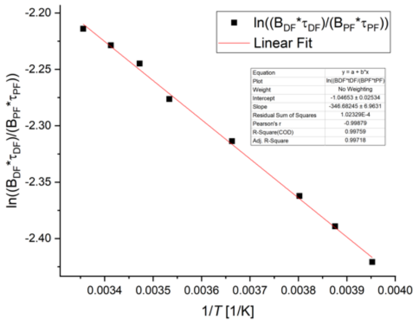

Figure S77. Linear fit of **4.**

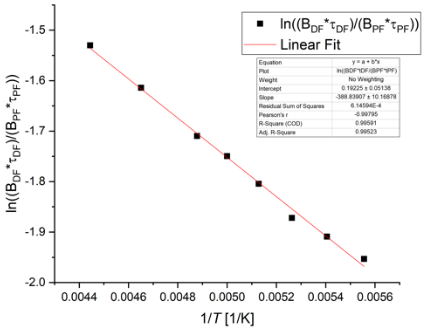

Figure S78. Linear fit of **5.**

Electrochemical data

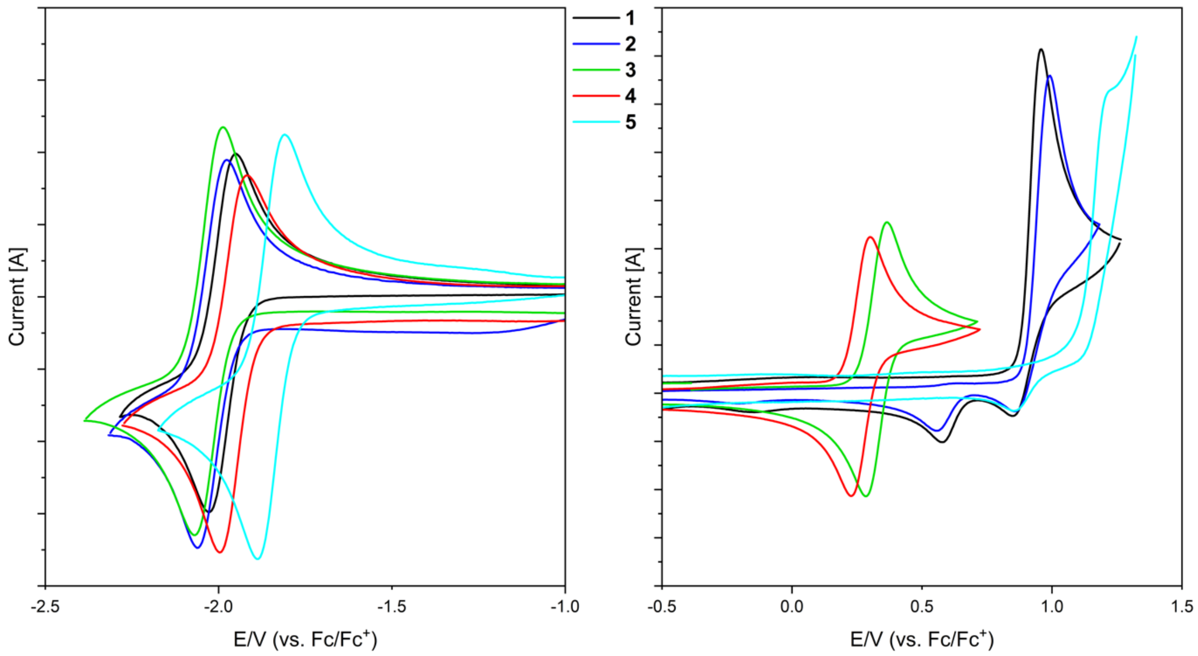

Figure S79. Cyclic voltammograms of **1** – **5**, reversible reductions (left) and oxidations (right). Potentials are given *vs.* ferrocene/ferrocenium (Fc/Fc^+).

Table S12. Reversible reduction and oxidation potentials of **1 - 5**.

Compound	$E_{1/2}$ vs. Fc/Fc^+ [V][a)]		E_{pa} vs. Fc/Fc^+ [V][a)]	MO energies exp. [eV][b)]	
	1st reduction potential	1st oxidation potential		HOMO	LUMO
**1**	–1.99		0.96	–6.20[c)]	–3.27
**2**	–2.02		0.99	–6.27[c)]	–3.24
**3**	–2.03	0.33		–5.39	–3.23
**4**	–1.96	0.27		–5.33	–3.30
**5**	–1.85		1.21	–6.45 [c)]	–3.40

a) Cyclic voltammograms were recorded in CH_2Cl_2/0.1 M [*n*Bu_4N][PF_6] with a scan rate of 250 mVs^{-1}.

b) Determined from the half wave potentials: HOMO = –(5.16+$E_{1/2,ox}$) eV, LUMO = –(5.16+$E_{1/2, red}$) eV.[42-43]

c) HOMO energies were estimated from E(HOMO) = E(LUMO)–E(onset, abs) with onset, abs: **1** = 423 nm (2.93 eV); **2** = 409 nm (3.03 eV); **5** = 407 nm (3.05 eV).

XYZ coordinates

Table S13. Cartesian coordinates (Å), energies (*E*, Hartree), and number of imaginary vibrational frequencies (N_{imag}) of the optimized structures at ground singlet (S_0) and triplet state (T_1) calculated at DFT/TZ2P/ZORA-BLYP-D3(BJ)//U-DFT/TZ2P/ZORA-LC-BLYP* for the benchmark molecules.

B1
E = –16.399
$\mathbf{N_{imag}}$ = 0
$\mathbf{S_0}$

C	-2.149312	-7.176319	-3.707052
C	-1.468305	-3.948517	-0.997891
C	-0.651772	-3.658416	0.117424
C	-0.192584	-2.369860	0.375722
C	-1.298130	-1.590082	-1.642468
C	-1.785131	-2.875950	-1.860521
H	-0.390990	-4.461165	0.803236
H	0.400744	-2.158433	1.260387
H	-1.820406	-7.919059	-2.972519
H	-1.518404	-0.786985	-2.339546
H	-2.410205	-3.065412	-2.730065
C	-4.594370	-4.035800	-0.447430
H	-5.607598	-4.039966	-0.031262
H	-3.902835	-4.351822	0.338059
H	-3.528361	-6.582753	0.921466
H	-3.540252	-8.338879	1.141214
H	-3.906471	-7.626372	-0.443194
H	1.979164	-10.220529	0.210751
H	0.484816	-10.917251	0.877569
H	1.448180	-9.794603	1.844450
H	1.376794	-4.965705	-1.318191
C	-7.025857	-6.090462	-4.343468
H	-4.579656	-7.208939	-4.819680
H	0.313691	-5.332733	-2.668526
H	1.782650	-6.291606	-2.417812
H	-7.909813	-5.892231	-3.727681
H	-7.110382	-7.101585	-4.758046
H	-7.048110	-5.388742	-5.189175
H	-1.300808	-6.504438	-3.879796
H	-2.363046	-7.693556	-4.647851
C	-0.505270	-1.328285	-0.513113
C	-1.176619	-6.616921	-0.719469
C	-1.800518	-7.611609	0.084030
C	-1.052222	-8.687386	0.575667
C	0.306941	-8.843588	0.273442
C	0.910205	-7.878500	-0.539589
C	0.202341	-6.769814	-1.023346
C	-3.273245	-7.536704	0.445834
H	-1.545204	-9.430747	1.201535
C	1.097276	-10.009737	0.825289
H	1.959299	-7.993961	-0.811027
C	0.953911	-5.788108	-1.908333
B	-1.995377	-5.392015	-1.266498
C	-3.329030	-5.580180	-2.076152
C	-4.524698	-4.924442	-1.679159
C	-5.707860	-5.119938	-2.404460
C	-5.750701	-5.927516	-3.545652
C	-4.568486	-6.569084	-3.937777
C	-3.375448	-6.424621	-3.220232
H	-4.339906	-2.997272	-0.692739
H	-6.621733	-4.633320	-2.064396
H	-2.299442	4.718316	-0.042669
H	0.145949	4.411458	0.311674
N	-0.020501	-0.015655	-0.271042
C	2.452978	-0.484133	0.063109
C	3.679500	0.132128	0.327765
C	1.315888	0.328538	0.005960
C	3.775094	1.524079	0.525209
C	1.396948	1.740863	0.185694
C	2.639649	2.333433	0.450263
C	0.056552	2.268003	0.015229
C	-2.172595	1.321019	-0.448479
C	-2.691842	2.616183	-0.363439
C	-0.794728	1.159749	-0.269087
C	-1.862536	3.724623	-0.100064
C	-0.490109	3.556457	0.095223
H	2.391666	-1.555617	-0.096716
H	4.577657	-0.478395	0.379128
H	4.744785	1.970054	0.731166
H	2.717839	3.409082	0.589227
H	-2.821257	0.473111	-0.642589
H	-3.759560	2.767755	-0.501461

B1
E = –23.480
$\mathbf{N_{imag}}$ = 0
$\mathbf{T_1}$

C	-2.055328	-7.020079	-3.783855
C	-1.459339	-3.951050	-1.005301
C	-0.447353	-3.665966	-0.001218
C	0.024247	-2.418335	0.227705
C	-1.493630	-1.539596	-1.509312
C	-1.940852	-2.795103	-1.743980
H	-0.089208	-4.492284	0.608157
H	0.734922	-2.235737	1.028811
H	-1.579846	-7.631158	-3.010520
H	-1.832168	-0.709742	-2.123261
H	-2.663825	-2.964743	-2.538293
C	-4.622183	-4.129761	-0.480415
H	-5.669902	-3.961770	-0.211737
H	-4.120333	-4.603780	0.369032
H	-3.390091	-6.472913	1.194005
H	-3.592333	-8.230050	1.177748
H	-3.918717	-7.270141	-0.282918
H	1.828010	-10.280517	0.296478
H	0.323936	-10.898466	1.009268
H	1.348987	-9.792141	1.925818
H	1.148287	-4.954559	-1.437233
C	-6.930844	-6.132923	-4.433930

H	-4.458063	-7.157199	-4.902313
H	0.333569	-5.674242	-2.819354
H	1.861613	-6.364367	-2.236948
H	-7.826250	-5.985110	-3.822459
H	-6.974342	-7.134102	-4.874310
H	-6.972424	-5.411649	-5.259278
H	-1.317388	-6.271443	-4.096290
H	-2.271030	-7.654987	-4.648587
C	-0.486191	-1.300137	-0.514607
C	-1.190608	-6.604772	-0.678763
C	-1.821446	-7.540312	0.168250
C	-1.115425	-8.629401	0.669414
C	0.220056	-8.847575	0.354430
C	0.839288	-7.936045	-0.489576
C	0.164700	-6.829363	-0.998953
C	-3.258214	-7.374207	0.583367
H	-1.629114	-9.333635	1.324144
C	0.969202	-10.018548	0.922002
H	1.879907	-8.095761	-0.772293
C	0.916332	-5.910269	-1.923326
B	-1.980155	-5.364605	-1.265148
C	-3.300910	-5.564043	-2.114164
C	-4.519431	-4.969344	-1.724927
C	-5.677474	-5.172830	-2.471023
C	-5.681259	-5.948615	-3.622056
C	-4.482561	-6.536522	-4.006402
C	-3.310454	-6.362729	-3.277163
H	-4.150030	-3.149172	-0.618291
H	-6.609306	-4.719136	-2.133097
H	-2.322890	4.649832	0.120742
H	0.129269	4.369150	0.369073
N	-0.019736	-0.024283	-0.276746
C	2.455034	-0.458289	-0.072150
C	3.674115	0.161433	0.163547
C	1.316615	0.336926	-0.039844
C	3.756716	1.533924	0.397935
C	1.389788	1.727082	0.161268
C	2.616786	2.328557	0.385701
C	0.039696	2.243912	0.035885
C	-2.176340	1.280456	-0.318889
C	-2.701446	2.559519	-0.200399
C	-0.796951	1.144499	-0.227973
C	-1.879195	3.662907	0.027514
C	-0.504251	3.511334	0.160227
H	2.402810	-1.518879	-0.291557
H	4.581403	-0.435189	0.151402
H	4.727286	1.988593	0.573559
H	2.689171	3.402119	0.537499
H	-2.825205	0.422885	-0.457673
H	-3.775929	2.698623	-0.273954

B2

E = –18.138

$\mathbf{N_{imag}}$ = 0

$\mathbf{S_0}$

N	-0.018266	-0.013068	-0.273869
C	0.982350	2.723936	0.225745
C	-0.386752	2.208253	0.691096
C	-0.831609	0.892611	0.431281
C	3.299951	-0.276447	-1.929462
C	3.816944	1.005867	-1.722259
C	3.045420	1.940062	-1.028155
C	1.768475	1.643282	-0.530036
C	1.256901	0.344461	-0.748364
C	2.035440	-0.604987	-1.448281
C	-1.241787	3.064226	1.400300
C	-2.106934	0.483922	0.882912
H	3.878009	-1.025535	-2.465535
H	4.802419	1.275712	-2.092660
H	3.452065	2.934919	-0.868510
C	-2.501396	2.663809	1.850651
C	-2.930678	1.360153	1.584492
H	-3.134093	3.357876	2.397587
H	-3.907178	1.020129	1.921389
C	1.805287	3.169247	1.471671
H	-0.913243	4.078832	1.608125
H	-2.448002	-0.524711	0.681162
H	1.642871	-1.601500	-1.613357
C	0.768046	3.945840	-0.717148
H	1.280248	3.954327	2.024942
H	2.783104	3.560293	1.173293
H	1.963698	2.319591	2.144314
H	1.727506	4.350629	-1.054360
H	0.224674	4.744497	-0.202348
H	0.190214	3.647426	-1.598376
C	-0.506511	-1.346370	-0.518945
C	-1.206816	-6.624266	-0.671098
C	-1.873113	-7.610051	0.109131
C	-1.153176	-8.683621	0.645612
C	0.218397	-8.846551	0.410914
C	0.864274	-7.890030	-0.379453
C	0.185204	-6.783155	-0.905758
C	-3.361770	-7.528928	0.397438
H	-1.678417	-9.420004	1.253133
C	0.976979	-10.010456	1.009711
H	1.924937	-8.011099	-0.597966
C	0.981733	-5.810520	-1.760828
B	-1.993438	-5.402959	-1.265922
C	-3.297298	-5.585448	-2.120968
C	-4.494631	-4.899049	-1.784997
C	-5.651126	-5.087456	-2.553240
C	-5.663362	-5.916569	-3.679877
C	-4.479081	-6.587420	-4.011965
C	-3.313300	-6.451379	-3.249539
H	-4.286752	-2.961349	-0.825086
H	-6.568262	-4.577152	-2.260205
C	-6.908037	-6.071180	-4.525774
H	-4.467164	-7.243565	-4.881742
C	-2.083254	-7.235158	-3.671813
C	-1.462266	-3.956659	-0.999354
C	-0.733718	-3.648671	0.169513
C	-0.273368	-2.356604	0.420229
C	-1.211141	-1.623924	-1.695068
C	-1.695697	-2.911932	-1.919200
H	-0.532407	-4.437799	0.890334
H	0.274460	-2.120154	1.328837
H	-1.803328	-7.974881	-2.914265
H	-1.380121	-0.825593	-2.413207
H	-2.262703	-3.120577	-2.823489

C	-4.594350	-3.984033	-0.574861
H	-5.627255	-3.941527	-0.213090
H	-3.958384	-4.311217	0.252264
H	-3.640601	-6.565739	0.839633
H	-3.661732	-8.316823	1.095637
H	-3.951052	-7.638675	-0.519162
H	1.888369	-10.227005	0.442128
H	0.360481	-10.916312	1.037039
H	1.276156	-9.788904	2.043844
H	1.355172	-4.970694	-1.162279
H	0.387389	-5.380311	-2.571587
H	1.847558	-6.313870	-2.204160
H	-7.811739	-5.838985	-3.952083
H	-6.998202	-7.089913	-4.919986
H	-6.879479	-5.389775	-5.387691
H	-1.213351	-6.584086	-3.815191
H	-2.266648	-7.760464	-4.614497

B2

E = –25.652

N$_{imag}$ = 0

T$_1$

N	-0.016649	-0.002687	-0.272022
C	0.988650	2.719592	0.231176
C	0.079391	1.961396	1.173651
C	-0.421463	0.680241	0.878420
C	2.139709	0.401166	-3.228245
C	2.548482	1.723033	-3.086161
C	2.149015	2.448450	-1.974987
C	1.315961	1.907074	-1.002751
C	0.858322	0.589506	-1.187178
C	1.314026	-0.167226	-2.283465
C	-0.332699	2.564337	2.356428
C	-1.375983	0.085014	1.725024
H	2.479479	-0.193390	-4.070923
H	3.200347	2.179316	-3.825485
H	2.509190	3.466286	-1.853636
C	-1.226637	1.951528	3.220032
C	-1.762096	0.711047	2.889672
H	-1.526144	2.453312	4.135577
H	-2.495961	0.237812	3.535067
C	2.303890	3.063558	0.959453
H	0.046664	3.551589	2.605128
H	-1.813485	-0.865082	1.443633
H	1.018563	-1.205811	-2.368910
C	0.285737	4.025900	-0.190349
H	2.100086	3.659728	1.853655
H	2.962757	3.643550	0.306904
H	2.830889	2.153607	1.262895
H	0.924135	4.601598	-0.866749
H	0.072087	4.645780	0.685072
H	-0.657847	3.813085	-0.702360
C	-0.499164	-1.311627	-0.513432
C	-1.433729	-6.575640	-0.397593
C	-2.304602	-7.396020	0.353397
C	-1.812678	-8.463068	1.099651
C	-0.457401	-8.772632	1.134477
C	0.401129	-7.976266	0.389508
C	-0.059125	-6.894397	-0.360990
C	-3.784212	-7.122921	0.397544
H	-2.513100	-9.075379	1.669109
C	0.056419	-9.917039	1.961479
H	1.466643	-8.209517	0.379245
C	0.956698	-6.107250	-1.145885
B	-1.983823	-5.361833	-1.262439
C	-3.067144	-5.610679	-2.397425
C	-4.322902	-4.966350	-2.375314
C	-5.267946	-5.214014	-3.370908
C	-5.017471	-6.084999	-4.422035
C	-3.782274	-6.723346	-4.446433
C	-2.821622	-6.504890	-3.463137
H	-4.255116	-3.027282	-1.419384
H	-6.237111	-4.716842	-3.312962
C	-6.033941	-6.319712	-5.503400
H	-3.559027	-7.419387	-5.256054
C	-1.504581	-7.223002	-3.587616
C	-1.469747	-3.955074	-1.002094
C	-0.681352	-3.621914	0.155030
C	-0.227779	-2.358410	0.404990
C	-1.259421	-1.594729	-1.677320
C	-1.733587	-2.856849	-1.894214
H	-0.446299	-4.414983	0.862154
H	0.374052	-2.147942	1.286982
H	-1.261201	-7.779542	-2.676761
H	-1.487730	-0.788080	-2.371307
H	-2.330534	-3.049242	-2.783533
C	-4.705380	-4.016803	-1.270919
H	-5.792475	-3.889871	-1.229634
H	-4.355085	-4.371363	-0.296752
H	-3.990911	-6.156009	0.872611
H	-4.306460	-7.896178	0.970302
H	-4.215994	-7.077963	-0.607525
H	1.038737	-10.249203	1.610596
H	-0.628390	-10.771114	1.927892
H	0.162366	-9.626796	3.014397
H	1.215153	-5.169132	-0.638807
H	0.574486	-5.828628	-2.132607
H	1.877864	-6.685489	-1.275120
H	-7.039763	-6.049013	-5.166595
H	-6.046263	-7.369060	-5.816880
H	-5.810000	-5.717562	-6.392987
H	-0.684362	-6.513329	-3.751529
H	-1.518802	-7.919666	-4.432109

B3

E = –16.605

***N*$_{imag}$** = 0

S$_0$

N	-2.537848	2.175404	-0.189336
O	-1.435728	4.745791	0.243775
C	-2.618155	4.358423	0.877355
C	-3.187811	3.085376	0.667255
C	0.753822	1.943998	-1.953434
C	1.268480	3.218428	-1.715028
C	0.517531	4.136354	-0.964921
C	-0.730588	3.776865	-0.472764
C	-1.265792	2.492889	-0.705085
C	-0.502404	1.580980	-1.449229

C	-3.226049	5.290903	1.708171
C	-4.396759	2.785441	1.312657
H	1.323077	1.220437	-2.531071
H	2.241914	3.505844	-2.102286
H	0.887906	5.137157	-0.758906
C	-4.435024	4.979324	2.348786
C	-5.015663	3.727252	2.145733
H	-4.909045	5.713515	2.994106
H	-5.953387	3.470795	2.631654
H	-0.897334	0.588118	-1.635769
H	-2.747654	6.257822	1.840207
H	-4.852617	1.813477	1.157760
C	-3.071140	0.851920	-0.369297
C	-3.876118	-4.408040	-0.367400
C	-4.509942	-5.366278	0.472079
C	-3.774835	-6.438108	0.990974
C	-2.421346	-6.626518	0.681845
C	-1.809256	-5.697986	-0.166653
C	-2.502453	-4.593062	-0.678338
C	-5.978252	-5.256818	0.843408
H	-4.273951	-7.152677	1.644781
C	-1.645695	-7.787758	1.263561
H	-0.764812	-5.839852	-0.443068
C	-1.744024	-3.651855	-1.600506
B	-4.678511	-3.187200	-0.941132
C	-6.032422	-3.367287	-1.713953
C	-7.197898	-2.654494	-1.325250
C	-8.401429	-2.846104	-2.016784
C	-8.493171	-3.704502	-3.117376
C	-7.339835	-4.400602	-3.502735
C	-6.128321	-4.261972	-2.816289
H	-6.895377	-0.698942	-0.428301
H	-9.292348	-2.315084	-1.682716
C	-9.789360	-3.865844	-3.880497
H	-7.389427	-5.079197	-4.353760
C	-4.937169	-5.075354	-3.291039
C	-4.108611	-1.744680	-0.735001
C	-3.310585	-1.428047	0.385416
C	-2.807977	-0.142502	0.580189
C	-3.846516	0.566072	-1.497623
C	-4.370278	-0.716098	-1.664582
H	-3.085816	-2.206065	1.111319
H	-2.204206	0.099788	1.451062
H	-4.628065	-5.805975	-2.535968
H	-4.037340	1.352549	-2.223299
H	-4.991078	-0.931627	-2.531107
C	-7.212072	-1.707530	-0.135965
H	-8.222855	-1.631603	0.278843
H	-6.540359	-2.029525	0.664668
H	-6.219178	-4.278812	1.275206
H	-6.246968	-6.022224	1.578339
H	-6.621065	-5.382079	-0.034444
H	-0.765673	-8.024222	0.655895
H	-2.269139	-8.686244	1.337652
H	-1.293934	-7.551250	2.277616
H	-1.325162	-2.804185	-1.044535
H	-2.378731	-3.231795	-2.385666
H	-0.912215	-4.177711	-2.081240
H	-10.650677	-3.574401	-3.270002
H	-9.931257	-4.901533	-4.210370
H	-9.792126	-3.234791	-4.780331
H	-4.065628	-4.443177	-3.495346
H	-5.182207	-5.614815	-4.211435

B3
E = –23.878
N_{imag} = 0
T$_1$

N	-2.471005	2.154681	-0.099078
O	-1.424511	4.719765	0.300603
C	-2.236629	4.153458	1.231154
C	-2.785126	2.872767	1.041607
C	-0.409017	2.646443	-3.110405
C	0.060255	3.945564	-2.893665
C	-0.293749	4.631174	-1.749690
C	-1.129705	4.022634	-0.827793
C	-1.632246	2.726664	-1.039678
C	-1.240196	2.038752	-2.198265
C	-2.521659	4.899311	2.363190
C	-3.664141	2.371403	2.013867
H	-0.110817	2.105583	-4.002795
H	0.716157	4.414572	-3.620335
H	0.068842	5.633101	-1.541855
C	-3.370117	4.377254	3.317978
C	-3.945387	3.116022	3.135710
H	-3.600035	4.955899	4.207097
H	-4.627325	2.719839	3.881234
H	-1.602443	1.028278	-2.349832
H	-2.074085	5.883121	2.464788
H	-4.111653	1.396668	1.856151
C	-3.004094	0.845959	-0.302058
C	-4.038400	-4.389678	-0.115881
C	-4.851050	-5.190248	0.716665
C	-4.319270	-6.276578	1.403736
C	-2.977799	-6.626230	1.299514
C	-2.176817	-5.849517	0.476040
C	-2.678425	-4.749227	-0.219182
C	-6.309128	-4.873906	0.912113
H	-4.976391	-6.871821	2.038864
C	-2.418520	-7.792504	2.063578
H	-1.125085	-6.112857	0.357405
C	-1.721987	-3.986269	-1.096333
B	-4.640202	-3.153874	-0.916171
C	-5.839234	-3.375535	-1.937368
C	-7.074162	-2.710682	-1.787117
C	-8.119908	-2.937906	-2.681708
C	-7.994173	-3.808429	-3.753808
C	-6.779292	-4.466930	-3.905638
C	-5.720525	-4.267820	-3.025639
H	-6.913382	-0.763168	-0.862559
H	-9.068910	-2.423944	-2.524228
C	-9.119808	-4.023468	-4.725150
H	-6.651532	-5.162955	-4.735527
C	-4.435351	-5.005997	-3.285697
C	-4.071434	-1.764522	-0.703709
C	-3.150559	-1.461264	0.352937
C	-2.638190	-0.207421	0.558728
C	-3.899305	0.603783	-1.362398
C	-4.413439	-0.653255	-1.543112

H	-2.856973	-2.263743	1.026737
H	-1.940363	-0.019074	1.372846
H	-4.115578	-5.579074	-2.409627
H	-4.186212	1.421390	-2.021516
H	-5.104463	-0.822693	-2.366481
C	-7.327160	-1.756762	-0.650338
H	-8.401837	-1.644159	-0.472268
H	-6.847909	-2.096588	0.272123
H	-6.436826	-3.897860	1.395327
H	-6.790679	-5.627596	1.543343
H	-6.841730	-4.822296	-0.042773
H	-1.463804	-8.120653	1.640859
H	-3.108881	-8.642577	2.053997
H	-2.242758	-7.529422	3.113963
H	-1.362414	-3.076841	-0.599236
H	-2.202181	-3.658555	-2.022704
H	-0.851271	-4.601916	-1.345636
H	-10.076172	-3.705110	-4.298667
H	-9.204179	-5.077648	-5.009496
H	-8.961327	-3.450543	-5.647151
H	-3.622363	-4.307458	-3.517140
H	-4.542971	-5.690040	-4.133575

B4
E = –19.861
N_{imag} = 0
S_0

N	-0.982465	1.488642	-0.352755
C	0.308458	4.105662	0.119417
C	-1.119179	3.933415	-0.418189
C	-1.702813	2.664882	-0.632565
C	2.283265	0.313953	0.981706
C	2.936811	1.526146	1.223512
C	2.271844	2.720086	0.936634
C	0.970375	2.752371	0.414910
C	0.322249	1.520446	0.174039
C	0.991253	0.309581	0.462408
C	-1.884323	5.069248	-0.720680
C	-3.021199	2.576710	-1.133523
H	2.775791	-0.631822	1.195137
H	3.945650	1.544067	1.627367
H	2.784667	3.659315	1.124535
C	-3.186934	4.987140	-1.217163
C	-3.753587	3.725400	-1.421964
H	-3.746809	5.891802	-1.439315
H	-4.766249	3.630023	-1.806755
C	0.258645	4.943288	1.432007
H	-1.448077	6.051966	-0.564319
H	-3.468581	1.603044	-1.296158
H	0.493305	-0.635045	0.277176
C	1.159860	4.861159	-0.944950
H	-0.191097	5.924538	1.251633
H	1.264926	5.101599	1.831992
H	-0.337066	4.424121	2.190373
H	2.182302	5.016411	-0.585984
H	0.724287	5.840587	-1.167140
H	1.203923	4.283627	-1.874539
H	-2.248104	-7.731621	1.026295
B	-3.519269	-3.650644	-1.243394
O	-1.947186	-2.210482	-3.234240
C	-2.119625	-1.657173	-1.983217
C	-2.865933	-2.287174	-0.963578
C	-3.539937	-5.873645	-4.422112
C	-2.762707	-5.086089	-5.294284
C	-2.241820	-3.866151	-4.874972
C	-2.503938	-3.433172	-3.566843
C	-3.277230	-4.190606	-2.660070
C	-3.787212	-5.426088	-3.131185
C	-1.495236	-0.415388	-1.794387
C	-2.962622	-1.605995	0.274172
H	-3.940443	-6.824720	-4.763758
H	-2.565240	-5.430939	-6.306398
H	-1.640643	-3.247369	-5.535555
C	-1.619202	0.213698	-0.561571
C	-2.354991	-0.376605	0.482849
H	-2.432425	0.141502	1.434391
H	-4.385278	-6.023576	-2.447036
H	-0.926569	0.043949	-2.597209
H	-3.533286	-2.075110	1.071717
C	-4.359269	-4.419799	-0.162924
C	-5.746658	-4.197400	-0.045237
C	-6.479516	-4.886403	0.931286
C	-5.868487	-5.795848	1.801469
C	-4.488783	-6.008193	1.674999
C	-3.731192	-5.335575	0.709801
H	-7.844317	-2.362183	0.474505
H	-7.551185	-4.715524	1.022857
H	-5.191274	-6.509451	4.457237
H	-3.995789	-6.711158	2.343368
H	-2.409906	-7.334883	-0.694682
C	-6.442190	-3.209585	-0.980128
H	-5.674444	-2.794085	-1.648253
C	-7.487630	-3.906761	-1.877020
H	-7.024639	-4.714844	-2.454652
H	-7.934059	-3.191791	-2.579097
H	-8.295148	-4.340379	-1.274945
C	-7.057073	-2.021946	-0.208878
H	-6.292836	-1.507605	0.384709
H	-7.500455	-1.297486	-0.903093
C	-6.686883	-6.531311	2.855743
H	-7.726540	-6.191398	2.750536
C	-6.670155	-8.059669	2.628329
H	-7.026577	-8.308850	1.622265
H	-7.312226	-8.566743	3.359190
H	-5.654805	-8.460519	2.735270
C	-6.223410	-6.179590	4.287275
H	-6.263393	-5.097374	4.456103
H	-6.861810	-6.670523	5.032210
C	-2.227387	-5.582707	0.599824
H	-1.851546	-4.961335	-0.225336
C	-1.479241	-5.137304	1.874826
H	-0.396880	-5.269933	1.755460
H	-1.799480	-5.725157	2.743407
H	-1.675836	-4.080990	2.090496
C	-1.909535	-7.049191	0.237498
H	-0.829517	-7.190565	0.107615

B4
E = –28.518

N_{imag} = 0

T₁

N	-0.989585	1.484455	-0.297206
C	0.413936	4.031145	0.085381
C	-0.863300	3.878918	-0.706290
C	-1.524288	2.642221	-0.844765
C	1.876753	0.278379	1.651620
C	2.540854	1.474136	1.915803
C	2.045355	2.665139	1.412970
C	0.880329	2.713260	0.657862
C	0.196856	1.503317	0.428088
C	0.719634	0.287430	0.911014
C	-1.443003	4.991653	-1.300674
C	-2.757195	2.564054	-1.524186
H	2.272817	-0.662349	2.020537
H	3.455099	1.474037	2.501446
H	2.588549	3.584938	1.607289
C	-2.637057	4.907806	-1.998274
C	-3.299985	3.687336	-2.100522
H	-3.061450	5.799429	-2.449703
H	-4.248209	3.619654	-2.624093
C	0.168231	5.025937	1.238948
H	-0.953044	5.956339	-1.210452
H	-3.266475	1.609840	-1.577826
H	0.198503	-0.634742	0.685003
C	1.516895	4.589512	-0.838072
H	-0.139725	5.997475	0.844478
H	1.083710	5.169066	1.818486
H	-0.613908	4.660188	1.910214
H	2.443125	4.738745	-0.277576
H	1.211655	5.553073	-1.253882
H	1.718496	3.902870	-1.665114
H	-1.970526	-7.275658	0.129271
B	-3.603435	-3.576190	-1.231554
O	-2.230566	-2.033726	-3.261080
C	-2.292049	-1.546438	-1.979509
C	-2.945309	-2.256641	-0.937697
C	-3.892153	-5.598929	-4.538705
C	-3.203737	-4.766648	-5.417462
C	-2.658934	-3.574970	-4.954928
C	-2.808197	-3.231824	-3.618936
C	-3.491656	-4.038221	-2.697903
C	-4.028082	-5.233758	-3.208853
C	-1.676418	-0.329067	-1.797157
C	-2.911970	-1.621684	0.348146
H	-4.320291	-6.531861	-4.896726
H	-3.089527	-5.042272	-6.462353
H	-2.116591	-2.904762	-5.616873
C	-1.671234	0.241020	-0.501478
C	-2.287018	-0.415296	0.564966
H	-2.274770	0.036823	1.554265
H	-4.567063	-5.883352	-2.520884
H	-1.189172	0.165369	-2.632556
H	-3.405002	-2.126154	1.175748
C	-4.355600	-4.429587	-0.130273
C	-5.760336	-4.462705	-0.078247
C	-6.409440	-5.219973	0.894984
C	-5.704979	-5.955845	1.836834
C	-4.316009	-5.918814	1.788329
C	-3.639413	-5.174402	0.827924
H	-7.284097	-2.210513	0.448509
H	-7.497482	-5.241202	0.928419
H	-5.043709	-6.412535	4.516255
H	-3.747292	-6.489071	2.520443
H	-2.091119	-6.118918	-1.211974
C	-6.565828	-3.611160	-1.042179
H	-5.983943	-3.541965	-1.969711
C	-7.931680	-4.188896	-1.396142
H	-7.851190	-5.220381	-1.754580
H	-8.400910	-3.588352	-2.182802
H	-8.609670	-4.182532	-0.534852
C	-6.715820	-2.191537	-0.489715
H	-5.736640	-1.744400	-0.290111
H	-7.251619	-1.551145	-1.200423
C	-6.429064	-6.772621	2.883278
H	-7.505037	-6.632037	2.712394
C	-6.122869	-8.263632	2.747508
H	-6.376763	-8.630092	1.747989
H	-6.688075	-8.845557	3.484115
H	-5.056412	-8.454912	2.913866
C	-6.110840	-6.289759	4.297671
H	-6.357821	-5.230399	4.418750
H	-6.674686	-6.864465	5.040940
C	-2.123228	-5.209497	0.766964
H	-1.799578	-4.238008	0.372337
C	-1.448359	-5.409148	2.119476
H	-0.363479	-5.297714	2.017317
H	-1.634259	-6.412071	2.521309
H	-1.800939	-4.679677	2.856036
C	-1.659055	-6.283389	-0.219875
H	-0.566618	-6.281980	-0.313336

B5

E = –20.465

N_{imag} = 0

S₀

C	-4.554745	0.690536	2.911702
N	-5.169363	4.438521	1.121950
C	-6.453982	2.024555	-3.894532
C	-5.484669	2.625975	-0.249100
C	-4.376010	1.842283	3.713070
C	-4.545289	3.153881	3.251071
C	-4.910702	3.286436	1.893101
C	-5.105012	2.145640	1.061566
C	-4.925556	0.852530	1.577173
C	-4.342723	4.315648	4.202011
C	-5.512137	4.049516	-0.189490
C	-5.828339	4.827294	-1.325052
C	-6.123521	4.112438	-2.493143
C	-6.115552	2.700796	-2.581514
C	-5.790103	1.959483	-1.446005
H	-5.768941	0.872323	-1.487362
C	-5.863181	6.341520	-1.343414
H	-6.371232	4.684646	-3.386412
C	-4.346363	-0.685433	3.510861
H	-4.094124	1.706853	4.756571
H	-5.076456	-0.016342	0.939732
H	-6.380776	0.935398	-3.808310
H	-5.776093	2.347554	-4.695516

H	-7.474358	2.270260	-4.217543
H	-4.121328	3.939005	5.205179
H	-5.232744	4.951188	4.265011
H	-3.514462	4.961721	3.890667
H	-5.009276	-0.850282	4.370424
H	-4.917214	6.777967	-1.005424
H	-6.647194	6.739179	-0.688721
H	-6.058793	6.695432	-2.360154
H	-3.316245	-0.811615	3.869769
H	-4.545554	-1.471185	2.774670
H	-1.947521	10.763673	7.042638
H	-3.321205	9.754190	7.531715
B	-4.578131	9.874172	3.024487
O	-7.107327	8.425650	2.908037
C	-5.954957	7.804435	2.476358
C	-4.693119	8.437495	2.489628
C	-7.261043	12.311580	4.405715
C	-8.416914	11.513356	4.296972
C	-8.340644	10.218961	3.792786
C	-7.091457	9.722018	3.392966
C	-5.908494	10.487956	3.483085
C	-6.034231	11.799802	4.004427
C	-6.125210	6.487153	2.028298
C	-3.592801	7.684439	2.013097
H	-7.335872	13.320850	4.802537
H	-9.380533	11.908212	4.609689
H	-9.222525	9.590427	3.703860
C	-5.013581	5.787786	1.575719
C	-3.738432	6.382315	1.560308
H	-2.891656	5.808239	1.195436
H	-5.133624	12.404280	4.083586
H	-7.106023	6.022031	2.040245
H	-2.613797	8.156709	2.010822
C	-3.204497	10.629769	3.103344
C	-2.766867	11.436253	2.032944
C	-1.527435	12.086395	2.115607
C	-0.704002	11.955612	3.239186
C	-1.149559	11.150395	4.296578
C	-2.381877	10.489846	4.243286
H	-2.026059	11.479779	-0.695847
H	-1.187797	12.708859	1.289035
H	1.758856	11.113558	4.360297
H	-0.522286	11.036221	5.178257
H	-3.597921	11.257054	6.621886
C	-3.636009	11.583661	0.785307
H	-4.563162	11.020409	0.963248
C	-4.044012	13.050408	0.531589
H	-4.561500	13.465184	1.404022
H	-4.714501	13.120982	-0.333719
H	-3.165477	13.675021	0.330390
C	-2.961088	10.959567	-0.455552
H	-2.725308	9.904061	-0.278815
H	-3.620806	11.024270	-1.329641
C	0.638188	12.674125	3.305974
H	0.757766	13.220598	2.360190
C	0.672887	13.710570	4.451715
H	-0.145652	14.432394	4.350434
H	1.622342	14.260175	4.448067
H	0.570476	13.219194	5.426999
C	1.815424	11.680216	3.422925
H	1.805028	10.962827	2.594337
H	2.774245	12.213041	3.408176
C	-2.836055	9.607756	5.405682
H	-3.849912	9.252962	5.172468
C	-1.943318	8.356090	5.551772
H	-2.310286	7.709630	6.358512
H	-0.909639	8.638631	5.784910
H	-1.934290	7.775315	4.622793
C	-2.931433	10.393876	6.730410

B5
E = –28.982
***N*imag** = 0
T1

C	-4.983657	0.684681	2.979019
N	-5.105590	4.392363	1.150267
C	-6.106880	2.195263	-3.934816
C	-5.444522	2.641669	-0.260178
C	-4.783425	1.805384	3.782711
C	-4.798042	3.116180	3.304930
C	-5.035176	3.246854	1.924082
C	-5.239673	2.123735	1.085228
C	-5.215802	0.854166	1.600424
C	-4.573320	4.257168	4.242424
C	-5.354569	4.052454	-0.168408
C	-5.511012	4.886983	-1.290714
C	-5.755088	4.223000	-2.493616
C	-5.844258	2.837496	-2.610724
C	-5.685962	2.034407	-1.464561
H	-5.757368	0.952152	-1.545913
C	-5.438169	6.378493	-1.261315
H	-5.883842	4.827611	-3.388705
C	-4.953124	-0.691697	3.561464
H	-4.604715	1.655347	4.845197
H	-5.371657	-0.014894	0.965275
H	-5.291232	1.514009	-4.202789
H	-6.210969	2.938858	-4.728041
H	-7.023332	1.595296	-3.898917
H	-4.408877	3.878426	5.253723
H	-5.427868	4.941982	4.251292
H	-3.711521	4.861449	3.940523
H	-4.771085	-0.668061	4.638162
H	-4.457542	6.726566	-0.919953
H	-6.163784	6.801525	-0.558418
H	-5.630796	6.778211	-2.259461
H	-4.167884	-1.293746	3.090107
H	-5.902060	-1.209403	3.380983
H	-2.689457	10.824516	6.765847
H	-3.548261	9.300504	7.075612
B	-4.540106	9.805736	3.053556
O	-7.037348	8.340851	2.987070
C	-5.890220	7.725924	2.549113
C	-4.633501	8.386960	2.562336
C	-7.267023	12.209824	4.438472
C	-8.396916	11.399150	4.362312
C	-8.282577	10.102560	3.869837
C	-7.041461	9.635438	3.461809
C	-5.875192	10.414462	3.522522
C	-6.037008	11.719569	4.025402

C	-6.063761	6.432933	2.102326
C	-3.531284	7.608960	2.083159
H	-7.350861	13.224129	4.821599
H	-9.366240	11.771338	4.683510
H	-9.146123	9.445947	3.796625
C	-4.937115	5.726837	1.636018
C	-3.675286	6.314455	1.629393
H	-2.819135	5.749171	1.266329
H	-5.153560	12.353905	4.084558
H	-7.051341	5.979556	2.111490
H	-2.546406	8.071166	2.081303
C	-3.179320	10.616736	3.081582
C	-2.902976	11.595610	2.109197
C	-1.699598	12.299288	2.141911
C	-0.741256	12.061879	3.117848
C	-1.012360	11.090963	4.076436
C	-2.205221	10.374105	4.071860
H	-2.651753	10.986355	-0.584291
H	-1.493614	13.056241	1.386445
H	1.837977	11.210122	3.791858
H	-0.271622	10.890911	4.848865
H	-4.211736	10.476904	5.919596
C	-3.892265	11.831137	0.981709
H	-4.891457	11.628321	1.387732
C	-3.893891	13.256429	0.438250
H	-4.026786	13.991813	1.238910
H	-4.709627	13.382839	-0.282118
H	-2.959345	13.491838	-0.084751
C	-3.648188	10.830274	-0.151773
H	-3.707445	9.800481	0.215968
H	-4.389953	10.957052	-0.949781
C	0.557376	12.837495	3.135523
H	0.530170	13.528730	2.281772
C	0.701397	13.670185	4.409713
H	-0.142822	14.356387	4.531212
H	1.626080	14.258259	4.388128
H	0.735549	13.020969	5.292640
C	1.767461	11.919903	2.959113
H	1.693944	11.342359	2.032007
H	2.696239	12.501274	2.933005
C	-2.492774	9.367253	5.171485
H	-3.132765	8.591759	4.731296
C	-1.250587	8.682335	5.732332
H	-1.542193	7.885521	6.425534
H	-0.618309	9.382570	6.291406
H	-0.641906	8.239852	4.936487
C	-3.287184	10.030769	6.300092

B6
E = –23.489
$\boldsymbol{N_{imag}}$ = 0
$\mathbf{S_0}$

N	0.427508	0.293120	0.311781
H	-0.978143	-1.084946	2.410255
C	-0.656018	-0.274275	-0.387784
C	-0.848665	-1.670530	-0.398305
C	3.524600	-2.078843	1.944245
C	3.637493	-0.683700	1.923186
C	2.618185	0.096553	1.383827
C	1.454956	-0.504538	0.853586
C	1.331802	-1.908465	0.881136
C	2.373876	-2.669580	1.423949
C	-1.567161	0.553213	-1.081151
C	-1.935498	-2.199359	-1.104737
H	4.316681	-2.693752	2.362906
H	4.523137	-0.197619	2.325520
H	2.719529	1.175552	1.368662
C	-2.642994	0.001589	-1.771741
C	-2.834777	-1.384961	-1.791630
H	-3.331023	0.659399	-2.297502
H	-3.672046	-1.822029	-2.328980
H	5.274890	8.327414	0.883961
H	-1.426298	1.627879	-1.077423
H	-2.076297	-3.277173	-1.107022
H	2.270613	-3.751485	1.440837
C	0.051653	-2.619639	0.410290
O	-0.605126	-5.453515	1.055210
C	0.093274	-5.135927	-0.100510
C	0.428596	-3.826914	-0.455652
C	-1.888114	-2.519696	3.728200
C	-2.151695	-3.878890	3.955649
C	-1.709782	-4.833672	3.043585
C	-1.002616	-4.428408	1.902590
C	-0.727122	-3.080318	1.654562
C	-1.184006	-2.136863	2.588853
C	0.450188	-6.227715	-0.906227
C	1.138605	-3.634082	-1.651207
H	-2.230384	-1.769078	4.435734
H	-2.699844	-4.192170	4.840548
H	-1.899375	-5.893367	3.192769
C	1.154137	-6.010136	-2.087194
C	1.502259	-4.703872	-2.464837
H	1.431213	-6.855194	-2.712379
H	2.051909	-4.527212	-3.385550
H	1.403896	-2.618764	-1.935523
H	0.167135	-7.227604	-0.588376
H	5.022317	7.373599	2.358585
B	1.022975	6.062632	0.378909
O	-0.374198	4.511747	2.416742
C	0.196454	3.858165	1.346853
C	0.888723	4.531755	0.317234
C	-0.258427	8.638838	2.979609
C	-0.904104	7.784374	3.894782
C	-0.929686	6.409408	3.685373
C	-0.300678	5.887768	2.545519
C	0.357487	6.707571	1.602776
C	0.358080	8.101613	1.857688
C	0.040142	2.463680	1.353523
C	1.424574	3.734766	-0.723630
H	-0.246631	9.711290	3.156669
H	-1.388745	8.199070	4.775381
H	-1.423142	5.736753	4.381525
C	0.584211	1.725137	0.310772
C	1.280917	2.355426	-0.737692
H	1.694033	1.749165	-1.538686
H	0.860504	8.748891	1.142575
H	-0.496864	1.974127	2.160131
H	1.960311	4.236607	-1.525557
C	1.780949	6.886148	-0.721630

C	1.092357	7.368829	-1.853434
C	1.787092	8.095110	-2.830566
C	3.156643	8.356415	-2.715213
C	3.830278	7.870722	-1.586182
C	3.163819	7.143272	-0.594007
H	-0.421080	6.771242	-4.174878
H	1.256210	8.468878	-3.704903
H	5.767281	8.018648	-3.759712
H	4.895855	8.063286	-1.479534
H	3.748941	8.469445	1.775994
C	-0.403240	7.099110	-2.008668
H	-0.723699	6.514648	-1.134444
C	-1.225602	8.405679	-2.000525
H	-1.036383	8.976341	-1.084378
H	-2.299338	8.188252	-2.056697
H	-0.962957	9.040488	-2.855215
C	-0.707358	6.243630	-3.257262
H	-0.153550	5.298539	-3.224375
H	-1.778327	6.014288	-3.318401
C	3.889227	9.147463	-3.792008
H	3.147091	9.410573	-4.558504
C	4.472576	10.465614	-3.235488
H	3.687529	11.077003	-2.776103
H	4.942895	11.049364	-4.036406
H	5.233961	10.265752	-2.471755
C	4.986287	8.301156	-4.476069
H	4.564850	7.380262	-4.895006
H	5.461059	8.866007	-5.287822
C	3.925901	6.620691	0.622884
H	3.201285	6.102157	1.266684
C	4.997284	5.582637	0.224713
H	5.495722	5.178692	1.114504
H	5.762851	6.034966	-0.416969
H	4.545031	4.749770	-0.325371
C	4.529457	7.767604	1.461310
H	5.505675	8.157654	1.195977
H	-1.674797	1.619880	-0.679086
H	-2.537521	-3.195392	0.007581
H	2.399395	-3.751498	0.950572
C	0.032272	-2.611680	0.502031
O	-0.824320	-5.396467	0.936351
C	-0.333555	-5.006400	-0.284232
C	0.104119	-3.716035	-0.548709
C	-0.614551	-2.929900	4.253799
C	-1.034234	-4.254180	4.360165
C	-1.090143	-5.053806	3.234890
C	-0.730517	-4.529458	1.996376
C	-0.312299	-3.212061	1.865787
C	-0.259037	-2.424880	3.016735
C	-0.304738	-5.994815	-1.264701
C	0.578904	-3.435312	-1.830565
H	-0.566088	-2.296607	5.134319
H	-1.315235	-4.663835	5.326032
H	-1.407705	-6.091338	3.286896
C	0.165656	-5.692597	-2.527968
C	0.612872	-4.404509	-2.815829
H	0.187860	-6.463708	-3.292590
H	0.985263	-4.161076	-3.806207
H	0.927939	-2.428751	-2.051865
H	-0.654901	-6.990733	-1.008773
H	4.745361	7.871607	2.762109
B	0.979311	6.029936	0.236990
O	-0.706578	4.527103	2.051848
C	0.004306	3.855814	1.088885
C	0.848572	4.532302	0.169154
C	-0.714655	8.630602	2.575857
C	-1.490685	7.796196	3.378653
C	-1.467584	6.422665	3.174303
C	-0.666123	5.898644	2.168347
C	0.129160	6.698846	1.335487
C	0.074969	8.083909	1.577172
C	-0.172347	2.491597	1.090602
C	1.526008	3.688210	-0.781419
H	-0.731474	9.706052	2.734271
H	-2.114326	8.213591	4.164810
H	-2.061442	5.747655	3.785333
C	0.551061	1.704147	0.146672
C	1.396107	2.321794	-0.783967
H	1.924147	1.713974	-1.515233
H	0.682209	8.733322	0.948200
H	-0.820579	2.027361	1.828034
H	2.160060	4.169438	-1.522325
C	1.906963	6.860921	-0.738585
C	1.519687	7.114595	-2.066730
C	2.346559	7.857986	-2.908048
C	3.563067	8.366183	-2.473850
C	3.947146	8.110717	-1.161287
C	3.145027	7.371522	-0.297829
H	0.259386	7.167914	-4.675376
H	2.039281	8.053755	-3.934375
H	6.352718	8.411279	-2.701833
H	4.899243	8.499658	-0.804653
H	4.084082	9.104634	1.669479
C	0.167109	6.633915	-2.560703
H	-0.072380	5.723440	-1.995688

B6
E = –33.400
$\boldsymbol{N_{imag}}$ = 0
$\mathbf{T_1}$

N	0.410271	0.289944	0.199838
H	0.070896	-1.391166	2.933423
C	-0.843126	-0.264881	-0.044473
C	-1.073175	-1.643630	0.113573
C	3.741151	-2.084811	1.080889
C	3.860744	-0.697404	1.035713
C	2.759783	0.082321	0.762591
C	1.511171	-0.517249	0.508156
C	1.375353	-1.912168	0.602259
C	2.504061	-2.671866	0.879299
C	-1.885299	0.574010	-0.492460
C	-2.351122	-2.131191	-0.114247
H	4.607228	-2.703379	1.295736
H	4.818628	-0.223367	1.226224
H	2.841278	1.162093	0.748023
C	-3.144441	0.060178	-0.706542
C	-3.386511	-1.296341	-0.505179
H	-3.940049	0.714384	-1.049081
H	-4.376853	-1.707075	-0.676861

C	-0.912837	7.670670	-2.238574
H	-0.940509	7.889458	-1.166068
H	-1.902755	7.311519	-2.544506
H	-0.708930	8.606735	-2.773434
C	0.135921	6.279942	-4.044275
H	0.927261	5.568656	-4.303946
H	-0.828860	5.829883	-4.302989
C	4.442813	9.173516	-3.402427
H	3.920642	9.234554	-4.367259
C	4.649022	10.598434	-2.888150
H	3.691843	11.110135	-2.745581
H	5.252967	11.182191	-3.592137
H	5.171131	10.588983	-1.924063
C	5.790636	8.491837	-3.639772
H	5.658188	7.481127	-4.039024
H	6.398041	9.067406	-4.347532
C	3.628144	7.048824	1.104866
H	2.736583	6.973666	1.740820
C	4.319095	5.682040	1.122259
H	4.623019	5.411915	2.140716
H	5.217456	5.706859	0.492738
H	3.653964	4.900432	0.740118
C	4.539819	8.109780	1.712799

B7

E = –22.820

$\boldsymbol{N_{imag}}$ = 0

S$_0$

C	-1.094203	0.614809	-1.797016
C	-2.418876	0.263927	-1.514336
C	-2.876560	0.223013	-0.169486
C	-1.958220	0.534997	0.869247
C	-0.633455	0.864519	0.562355
C	-0.200727	0.910384	-0.764933
N	1.149921	1.255887	-1.064913
B	-4.368677	-0.158830	0.162241
C	2.290797	0.637156	-0.529944
C	3.452323	1.268735	-1.065922
C	2.989549	2.313671	-1.958721
C	1.562048	2.276316	-1.935946
C	-5.217453	0.763936	1.106849
C	-4.995477	-1.458421	-0.456230
C	-5.959077	0.212668	2.187730
C	-6.691483	1.051722	3.036856
C	-6.754079	2.435964	2.836997
C	-6.033282	2.973952	1.763568
C	-5.260114	2.172679	0.915024
C	-6.267388	-1.420947	-1.091914
C	-6.799492	-2.581823	-1.666238
C	-6.132862	-3.811700	-1.604623
C	-4.887865	-3.848109	-0.965033
C	-4.305791	-2.701235	-0.411320
C	2.392892	-0.447182	0.348840
C	3.668766	-0.878605	0.709030
C	4.830317	-0.263707	0.188461
C	4.723375	0.804060	-0.709196
C	3.643782	3.270395	-2.748733
C	2.879611	4.172001	-3.493251
C	1.470256	4.129720	-3.448273
C	0.793001	3.186104	-2.670475
C	-4.508865	2.859826	-0.212611
C	-7.594345	3.319050	3.732767
C	-5.967208	-1.276394	2.489281
C	-2.945773	-2.849764	0.249317
C	-7.078527	-0.141527	-1.207848
C	-2.356342	0.514789	2.334332
C	-3.327752	-0.052124	-2.688826
N	6.120940	-0.738269	0.588933
C	7.089743	0.192909	1.045787
C	8.449568	0.044369	0.711294
C	9.391234	0.971774	1.160907
C	8.998140	2.070274	1.936737
C	7.644183	2.225898	2.261665
C	6.697308	1.296465	1.827844
C	6.412244	-2.122311	0.492123
C	7.227401	-2.757509	1.449903
C	7.498651	-4.122829	1.350588
C	6.954372	-4.886276	0.308827
C	6.135205	-4.259395	-0.638747
C	5.869072	-2.891102	-0.555831
C	-6.750394	-5.064987	-2.184383
H	-0.748064	0.642471	-2.826729
H	0.064799	1.104515	1.359746
H	-7.230402	0.612754	3.875896
H	-6.069095	4.048596	1.586956
H	-7.762150	-2.525858	-2.173541
H	-4.352141	-4.794858	-0.902111
H	1.510542	-0.933144	0.752567
H	3.780327	-1.708382	1.400629
H	5.620725	1.262710	-1.114638
H	4.730164	3.312082	-2.777129
H	3.373019	4.918199	-4.110852
H	0.895447	4.846507	-4.029650
H	-0.291640	3.162677	-2.638189
H	-3.429353	2.869292	-0.026067
H	-4.657884	2.359528	-1.175155
H	-4.844342	3.896475	-0.318323
H	-7.717262	2.876613	4.727255
H	-7.147491	4.313328	3.845087
H	-8.598565	3.458993	3.308378
H	-6.545250	-1.831043	1.742073
H	-4.959968	-1.706032	2.487798
H	-6.411381	-1.463613	3.472201
H	-2.696150	-3.908246	0.374670
H	-2.156892	-2.383420	-0.351150
H	-2.910826	-2.377848	1.236720
H	-6.476258	0.701551	-1.562322
H	-7.909131	-0.279296	-1.907538
H	-7.493878	0.156102	-0.238891
H	-2.888342	-0.403843	2.602952
H	-1.470835	0.589807	2.973058
H	-3.025565	1.347545	2.575083
H	-2.845783	0.225012	-3.631472
H	-3.569842	-1.119482	-2.727159
H	-4.280407	0.484230	-2.627322
H	8.761950	-0.796756	0.099958
H	10.436793	0.842035	0.890306
H	9.733632	2.793250	2.280061
H	7.322524	3.070751	2.866645

H	5.650207	1.421357	2.086943
H	7.643421	-2.177565	2.268069
H	8.128227	-4.595760	2.101070
H	7.163494	-5.950610	0.238670
H	5.708036	-4.835798	-1.456411
H	5.238102	-2.411568	-1.298042
H	-7.389847	-4.833806	-3.043443
H	-5.981759	-5.778042	-2.502356
H	-7.377280	-5.570683	-1.436404

B7

E = –32.355

N_{imag} = 0

T_1

	-1.040150	0.606054	-1.870380
C	-2.346759	0.255642	-1.591282
C	-2.840019	0.196037	-0.239258
C	-1.885914	0.519662	0.788496
C	-0.580129	0.850646	0.482818
C	-0.140962	0.898107	-0.842493
N	1.205606	1.233006	-1.136978
B	-4.301847	-0.189409	0.086822
C	2.306863	0.669883	-0.582158
C	3.481100	1.299021	-1.094579
C	3.024265	2.303000	-2.036677
C	1.619909	2.240078	-2.031836
C	-5.092555	0.485624	1.285303
C	-5.081902	-1.280285	-0.761184
C	-5.804123	-0.281552	2.236373
C	-6.533624	0.331997	3.251306
C	-6.609329	1.715739	3.371790
C	-5.906843	2.475588	2.445038
C	-5.159151	1.890432	1.424395
C	-6.383475	-1.045773	-1.262048
C	-7.072060	-2.034548	-1.959597
C	-6.526017	-3.293958	-2.185267
C	-5.244787	-3.528765	-1.702770
C	-4.525957	-2.555333	-1.010526
C	2.394166	-0.398882	0.327349
C	3.635980	-0.809064	0.725748
C	4.817811	-0.178841	0.242876
C	4.719618	0.885492	-0.686587
C	3.670700	3.234716	-2.835106
C	2.899021	4.088785	-3.614533
C	1.505084	4.021236	-3.586793
C	0.839904	3.096861	-2.790000
C	-4.439071	2.805632	0.470707
C	-7.437558	2.360617	4.446978
C	-5.764839	-1.786895	2.211532
C	-3.146225	-2.914948	-0.527951
C	-7.053919	0.292601	-1.096588
C	-2.246780	0.446596	2.246183
C	-3.251267	0.007661	-2.766119
N	6.052599	-0.607950	0.670160
C	7.185994	0.255515	0.633617
C	8.393371	-0.215518	0.123689
C	9.499374	0.619476	0.102202
C	9.406696	1.918929	0.587606
C	8.201600	2.382054	1.101694
C	7.090300	1.553460	1.130989
C	6.247881	-1.932925	1.160496
C	6.958953	-2.129766	2.341325
C	7.162893	-3.418060	2.810298
C	6.662396	-4.507434	2.106569
C	5.958956	-4.305627	0.925243
C	5.752721	-3.021026	0.445968
C	-7.301936	-4.362994	-2.901451
H	-0.704422	0.648925	-2.905123
H	0.116946	1.086422	1.285730
H	-7.054038	-0.292531	3.979115
H	-5.942588	3.563727	2.515971
H	-8.066621	-1.810767	-2.348600
H	-4.787027	-4.505202	-1.868424
H	1.496459	-0.873916	0.708541
H	3.729547	-1.617869	1.441355
H	5.621800	1.334605	-1.088879
H	4.755336	3.301844	-2.850480
H	3.385388	4.823615	-4.249186
H	0.925665	4.707635	-4.197313
H	-0.243593	3.048117	-2.756127
H	-3.352117	2.768266	0.615312
H	-4.611722	2.509528	-0.570608
H	-4.767182	3.841819	0.605372
H	-7.433898	1.763285	5.364862
H	-7.067008	3.362327	4.687567
H	-8.483968	2.465875	4.133765
H	-6.331790	-2.192481	1.366260
H	-4.738121	-2.153470	2.091352
H	-6.178278	-2.200622	3.137351
H	-2.968546	-3.990984	-0.628851
H	-2.368685	-2.384177	-1.091474
H	-3.004421	-2.626519	0.520178
H	-6.372531	1.111027	-1.358836
H	-7.943794	0.361202	-1.731091
H	-7.353581	0.469411	-0.057681
H	-2.777254	-0.487879	2.467305
H	-1.346736	0.501577	2.867793
H	-2.926021	1.250940	2.549239
H	-2.767074	0.322145	-3.696704
H	-3.532614	-1.046856	-2.861734
H	-4.194334	0.556287	-2.650727
H	8.457745	-1.231195	-0.255894
H	10.438741	0.253829	-0.302035
H	10.276173	2.569499	0.569663
H	8.127150	3.391662	1.494948
H	6.149965	1.902725	1.547823
H	7.344202	-1.273222	2.887078
H	7.711049	-3.571084	3.735286
H	6.824122	-5.515280	2.477572
H	5.576056	-5.153897	0.365525
H	5.216113	-2.856132	-0.483985
H	-7.933480	-3.937529	-3.688556
H	-6.634136	-5.100490	-3.358301
H	-7.963785	-4.903253	-2.212815

B8

E = –17.776

N_{imag} = 0

S$_0$

C	-1.804770	7.131518	3.496220
C	-3.772889	12.259217	4.509351
C	-2.406609	10.940763	6.558737
C	3.548442	5.051195	9.866367
C	0.028346	8.741916	10.602797
C	-2.542848	10.310864	5.312109
C	-2.050511	6.567453	2.236856
B	0.769012	6.107874	9.258307
C	-2.741185	7.289064	1.262678
C	-3.188335	8.587472	1.554150
C	-2.939631	9.145691	2.801468
C	-3.629069	12.879487	5.760470
C	-2.944844	12.215918	6.779436
C	0.017169	6.895196	8.116134
C	0.738417	7.716809	7.209016
C	2.246636	7.872242	7.281064
C	0.058402	8.402676	6.194583
C	-1.326959	8.309716	6.084239
C	-2.050148	7.518299	6.974486
C	-1.393929	6.797832	7.979897
C	-2.240285	5.946736	8.909164
C	0.516103	4.569886	9.435000
C	0.235197	4.024324	10.718546
C	-0.018208	2.654588	10.858090
C	-2.243769	8.429736	3.798330
C	0.028448	1.777252	9.768212
C	-0.261587	0.302660	9.939349
C	0.325680	2.314201	8.509891
N	-2.012577	9.035518	5.046442
C	0.547706	3.683652	8.323070
C	0.169558	4.883482	11.969845
C	0.851054	4.162155	6.913469
C	1.758501	6.875085	10.203385
C	3.056497	6.356370	10.468690
C	3.940444	7.065368	11.290633
O	-3.398268	10.447399	3.018152
C	3.578497	8.271658	11.902012
C	4.554137	9.027609	12.776387
C	2.293204	8.768689	11.655504
C	-3.234729	10.997308	4.291998
C	1.393829	8.108899	10.809573
H	-1.268094	6.568174	4.251966
H	-4.298261	12.747747	3.692770
H	-1.876071	10.426852	7.353045
H	4.628259	4.947580	10.013728
H	3.348068	4.987554	8.791786
H	3.056820	4.187779	10.327664
H	-0.130241	9.550757	11.323110
H	-0.787000	8.021302	10.723493
H	-0.067489	9.161601	9.595374
H	-1.697502	5.560934	2.027777
H	-2.935645	6.857819	0.284705
H	-3.729085	9.177943	0.819031
H	-4.049338	13.867704	5.924821
H	-2.823544	12.682173	7.753764
H	2.624673	8.359172	6.376764
H	2.756627	6.908783	7.383318
H	2.541315	8.476999	8.145474
H	0.605322	9.020101	5.485876
H	-3.131792	7.463283	6.876096
H	-2.089420	4.879242	8.715876
H	-3.302431	6.173380	8.774219
H	-1.991491	6.113256	9.962383
H	-0.251346	2.259424	11.846338
H	0.293545	-0.300068	9.212033
H	-1.330525	0.097050	9.786855
H	-0.003040	-0.040490	10.947138
H	0.383407	1.648086	7.649628
H	1.166054	5.218864	12.277273
H	-0.269972	4.317221	12.797255
H	-0.432388	5.786304	11.823129
H	1.722985	4.823290	6.877174
H	0.009926	4.725520	6.494730
H	1.049162	3.310109	6.255494
H	4.937148	6.661385	11.465289
H	5.262002	8.348203	13.263920
H	4.033533	9.603245	13.549659
H	5.141213	9.738683	12.178166
H	1.983539	9.697532	12.133446

B8

E = –25.543

N_{imag} = 0

T$_1$

C	-0.989030	7.729503	3.295394
C	-4.600441	11.714010	4.608096
C	-3.248076	10.408010	6.679395
C	3.401430	4.748750	9.522706
C	0.591338	8.875682	10.487616
C	-3.000744	10.035790	5.349281
C	-0.791272	7.475073	1.958756
B	0.724671	6.143904	9.208605
C	-1.462458	8.219959	0.982804
C	-2.345144	9.213660	1.350251
C	-2.548748	9.472519	2.696149
C	-4.843748	12.049604	5.923291
C	-4.160183	11.398868	6.956314
C	-0.021607	6.911090	8.099854
C	0.654405	7.819020	7.207283
C	2.143305	8.016816	7.265695
C	-0.023754	8.491446	6.210918
C	-1.400485	8.329165	6.052376
C	-2.099067	7.469495	6.900779
C	-1.440384	6.766905	7.889335
C	-2.285705	5.907530	8.787247
C	0.306931	4.671460	9.631714
C	0.110607	4.309835	10.982932
C	-0.197600	2.999479	11.335686
C	-1.866445	8.750388	3.691410
C	-0.316521	1.990045	10.387008
C	-0.670896	0.585589	10.784847
C	-0.121199	2.338418	9.057531
N	-2.094544	9.043731	5.023182
C	0.176733	3.644288	8.672533
C	0.194289	5.332580	12.084687
C	0.369391	3.913830	7.204434
C	1.950684	6.784740	9.988174

C	3.170396	6.093373	10.159486
C	4.210107	6.648618	10.899153
O	-3.448263	10.438215	3.015813
C	4.097805	7.893693	11.507671
C	5.238473	8.486146	12.284998
C	2.900497	8.577043	11.345126
C	-3.680856	10.717157	4.324240
C	1.845955	8.052137	10.600363
H	-0.481593	7.155297	4.062036
H	-5.101400	12.210785	3.783362
H	-2.705957	9.900969	7.469302
H	4.458407	4.469623	9.579168
H	3.097674	4.752571	8.469286
H	2.808755	3.965266	10.006966
H	0.617402	9.718611	11.185820
H	-0.301372	8.273402	10.690029
H	0.460250	9.274266	9.474115
H	-0.111810	6.683278	1.660624
H	-1.297836	8.009281	-0.068973
H	-2.893600	9.798192	0.618512
H	-5.561205	12.830202	6.154887
H	-4.345061	11.681583	7.987515
H	2.494300	8.548615	6.375470
H	2.660242	7.051941	7.331658
H	2.455098	8.581081	8.150899
H	0.520123	9.148955	5.534487
H	-3.176351	7.360770	6.785441
H	-2.106990	4.838449	8.631436
H	-3.348781	6.108265	8.619676
H	-2.048739	6.097508	9.840959
H	-0.353040	2.758790	12.388192
H	-0.357736	-0.133591	10.021700
H	-1.753785	0.471733	10.917916
H	-0.198526	0.307940	11.732668
H	-0.196897	1.566086	8.291093
H	1.224666	5.665441	12.249451
H	-0.193039	4.923697	13.023354
H	-0.379433	6.232414	11.833397
H	1.276146	4.501291	7.021858
H	-0.457897	4.503391	6.790592
H	0.434693	2.975632	6.643877
H	5.141694	6.090510	11.001767
H	5.777940	7.718886	12.849703
H	4.885837	9.246090	12.989136
H	5.964973	8.967984	11.619159
H	2.778378	9.551587	11.819466

Table S14. Cartesian coordinates (Å), energies (*E*, Hartree), and number of imaginary vibrational frequencies (N_{imag}) of the optimized structures at ground singlet (S_0) and triplet state (T_1) calculated at DFT/TZ2P/ZORA-BLYP-D3(BJ)//U-DFT/TZ2P/ZORA-LC-BLYP* for the model molecules.

Cbz-π
E = –15.365
$\mathbf{N_{imag}}$ = 0
$\mathbf{S_0}$

F	-0.034527	4.651416	13.619790
F	1.040839	6.084230	12.372155
F	-1.143080	6.260277	12.618410
F	1.372889	4.524144	7.340666
F	-0.671549	4.078193	6.653008
F	0.706654	2.448947	7.162291
F	4.807846	4.131118	10.162418
F	2.685361	3.797484	9.776660
F	3.994085	4.537065	8.164025
F	-0.234066	8.082922	10.775062
F	0.982954	9.582444	11.800690
F	0.639487	9.756828	9.640915
N	-1.931992	8.918745	5.199941
C	0.076163	6.815289	8.297972
C	0.778362	7.644992	7.394518
H	1.854346	7.757017	7.494992
C	0.131390	8.323946	6.368976
H	0.690029	8.961260	5.690873
C	-1.262540	8.217262	6.232063
C	-1.989356	7.411422	7.124042
H	-3.063945	7.314417	7.005984
C	-1.323033	6.714903	8.124998
H	-1.901634	6.082022	8.792093
C	0.107116	4.704379	9.939225
C	-0.358592	4.447634	11.256944
C	-1.152780	3.330712	11.561955
H	-1.507340	3.190847	12.577092
C	-1.482997	2.401015	10.585024
H	-2.099733	1.541043	10.827888
C	-0.984087	2.574036	9.299026
H	-1.189783	1.832603	8.533775
C	-0.208476	3.694455	8.984407
C	-0.113061	5.363184	12.445662
C	0.301322	3.705432	7.550641
C	2.173868	6.697196	9.953338
C	3.459123	6.092345	9.910753
C	4.631443	6.813511	10.188283
H	5.592156	6.315861	10.116537
C	4.576514	8.150074	10.560109
H	5.487570	8.702003	10.770477
C	3.333686	8.759355	10.689506
H	3.267411	9.788349	11.027775
C	2.163382	8.052082	10.395972
C	3.714685	4.648619	9.507517
C	0.888736	8.846745	10.641336
C	-1.565511	8.928496	3.838836
C	-2.488498	9.745783	3.123781
C	-2.350388	9.893544	1.736753
H	-3.046985	10.513363	1.177426
C	-1.311478	9.226993	1.083901
H	-1.191471	9.333684	0.008844
C	-0.420160	8.406904	1.803409
H	0.373761	7.885873	1.274193
C	-0.535664	8.242764	3.186973
C	-3.078070	9.723607	5.360379
C	-3.444846	10.253610	4.089252
C	-4.547475	11.113417	3.990945
H	-4.839294	11.526099	3.028246
C	-5.259283	11.438236	5.147516
H	-6.117433	12.102551	5.085896
C	-4.870532	10.921418	6.399073
H	-5.429915	11.197541	7.289411
C	-3.775148	10.061847	6.524588
B	0.809880	6.047266	9.433822
H	0.149063	7.598366	3.728324
H	-3.477703	9.680292	7.495717

Cbz-π
E = –22.626
$\mathbf{N_{imag}}$ = 0
$\mathbf{T_1}$

F	-0.238328	5.029402	13.631997
F	1.007467	6.236901	12.352026
F	-1.151952	6.477962	12.309030
F	1.172666	4.436375	7.279764
F	-0.902689	3.967282	6.852448
F	0.518542	2.382779	7.245700
F	4.759390	4.028355	9.752627
F	2.623935	3.750770	9.648121
F	3.686098	4.652535	7.980216
F	-0.130941	8.261241	10.683637
F	1.162604	9.643205	11.714636
F	0.949412	9.774707	9.564623
N	-1.918808	8.903163	5.218936
C	0.082881	6.804413	8.305180
C	0.766380	7.720970	7.429396
H	1.827442	7.895917	7.584630
C	0.134710	8.385178	6.426599
H	0.675183	9.104003	5.817734
C	-1.265316	8.216709	6.223995
C	-1.979079	7.328000	7.078678
H	-3.033896	7.147889	6.893080
C	-1.326900	6.646828	8.056562
H	-1.888842	5.948465	8.670625
C	0.107256	4.725056	9.977666
C	-0.296042	4.508565	11.314835
C	-0.957128	3.356703	11.728734
H	-1.246384	3.256003	12.769206
C	-1.253756	2.350508	10.831583
H	-1.769689	1.452623	11.156175
C	-0.870463	2.508471	9.515714
H	-1.072907	1.722857	8.794316
C	-0.204226	3.657385	9.103803

C	-0.155618	5.558863	12.385465
C	0.158910	3.633896	7.638417
C	2.188504	6.659251	9.938267
C	3.435472	5.994250	9.913351
C	4.623036	6.597621	10.314329
H	5.548177	6.033840	10.262769
C	4.636238	7.901551	10.766946
H	5.563701	8.370771	11.079002
C	3.442150	8.590243	10.823004
H	3.421629	9.609700	11.195692
C	2.254400	7.984715	10.427751
C	3.608908	4.614124	9.335149
C	1.053283	8.885058	10.591132
C	-1.435843	9.118302	3.919263
C	-2.385834	9.859716	3.193548
C	-2.148191	10.172444	1.868404
H	-2.873664	10.738832	1.291101
C	-0.964670	9.732677	1.282198
H	-0.761181	9.970472	0.242237
C	-0.047721	8.969644	2.002318
H	0.854774	8.614503	1.514322
C	-0.273915	8.636386	3.330949
C	-3.181005	9.507539	5.320622
C	-3.504432	10.104833	4.088778
C	-4.694505	10.793762	3.949626
H	-4.957477	11.266512	3.007432
C	-5.544535	10.883414	5.048353
H	-6.484258	11.420020	4.956432
C	-5.196464	10.316739	6.272810
H	-5.863476	10.424858	7.122616
C	-4.000723	9.630181	6.434839
B	0.803394	6.051918	9.422879
H	0.421647	8.006606	3.874185
H	-3.714810	9.228271	7.400367

Cbz-$^{Me}\pi$

E = –16.543

$\boldsymbol{N}_{\mathbf{imag}}$ = 0

S$_0$

F	-0.120267	3.979285	13.278245
F	0.782582	5.727717	12.333204
F	-1.409924	5.500298	12.359194
F	1.842698	4.924515	7.229704
F	-0.004829	4.219346	6.258517
F	1.596563	2.804070	6.753138
F	5.019828	4.761120	10.372932
F	3.039480	4.121791	9.712712
F	4.334356	5.274598	8.351145
F	-0.695534	7.671255	10.822734
F	0.115964	9.203979	12.155537
F	-0.046616	9.611842	10.006244
N	-1.961974	8.934193	5.232227
C	0.077751	6.816559	8.278912
C	0.704469	7.866511	7.577529
H	1.731242	8.131356	7.815728
C	0.059560	8.566488	6.556039
C	-1.272454	8.217823	6.260264
C	-1.949142	7.188777	6.943361
C	-1.249645	6.493370	7.931508
H	-1.762835	5.695676	8.462106
C	0.336102	4.544506	9.668981
C	-0.197340	4.044495	10.887390
C	-0.810018	2.783793	10.969806
H	-1.227653	2.455806	11.915235
C	-0.880629	1.950122	9.861793
H	-1.358105	0.977581	9.932880
C	-0.300764	2.368798	8.669563
H	-0.300374	1.710683	7.806651
C	0.292548	3.631544	8.575405
C	-0.224393	4.820245	12.195076
C	0.928029	3.911977	7.221591
C	1.990594	6.842092	10.148206
C	3.363535	6.478219	10.185343
C	4.351714	7.349581	10.670779
H	5.390391	7.038619	10.654229
C	4.016492	8.597804	11.178064
H	4.786526	9.266990	11.549668
C	2.675801	8.962035	11.231595
H	2.389411	9.912145	11.670944
C	1.688953	8.107161	10.730952
C	3.916217	5.163548	9.658017
C	0.271767	8.629473	10.914862
C	-2.192505	8.471510	3.931880
C	-2.912215	9.470979	3.209802
C	-3.253257	9.231627	1.870814
H	-3.799975	9.977769	1.298980
C	-2.878619	8.021627	1.280643
H	-3.137957	7.824582	0.243559
C	-2.163980	7.050412	2.011606
H	-1.881296	6.118666	1.527903
C	-1.808653	7.261738	3.347135
C	-2.518479	10.211371	5.364188
C	-3.120620	10.580264	4.123360
C	-3.753285	11.827203	4.016821
H	-4.220626	12.130673	3.082941
C	-3.776826	12.673733	5.128404
H	-4.263771	13.643022	5.057633
C	-3.179382	12.286855	6.345639
H	-3.214705	12.962536	7.196659
C	-2.543144	11.049465	6.482130
B	0.827449	6.040429	9.403950
H	-1.253517	6.516645	3.910363
H	-2.086782	10.747628	7.420837
C	-3.384558	6.853505	6.612415
H	-4.010730	7.752882	6.617173
H	-3.467756	6.412630	5.611500
H	-3.790140	6.140715	7.335803
C	0.760841	9.663829	5.790262
H	0.666941	9.512206	4.708986
H	0.323429	10.644573	6.013697
H	1.823029	9.695404	6.048394

Cbz-$^{Me}\pi$

E = –24.309

$\boldsymbol{N}_{\mathbf{imag}}$ = 0

T$_1$

F	-0.46591928	4.15350277	13.20735665
F	0.62001516	5.78472168	12.30726013
F	-1.51358253	5.57324261	11.95402590

F	1.83153000	4.90819527	7.13983130
F	-0.01133765	4.09664948	6.32865699
F	1.67328520	2.79813422	6.72794544
F	5.07152216	4.88219483	10.09593676
F	3.09059857	4.17564296	9.62037957
F	4.11724684	5.52764402	8.26383545
F	-0.75126042	7.77200816	10.74346921
F	0.03921832	9.21893694	12.13197735
F	0.07346225	9.64132794	10.00964564
N	-1.94888158	8.91539222	5.25126065
C	0.10022736	6.78996434	8.27520562
C	0.66430275	7.94909667	7.63936236
H	1.67345777	8.24824113	7.91426954
C	0.02515604	8.66367253	6.67476372
C	-1.27048628	8.20930132	6.23263531
C	-1.87017000	7.03192721	6.81374057
C	-1.19479996	6.38617137	7.80229480
H	-1.68474532	5.54822777	8.29347217
C	0.39692414	4.52292792	9.64686005
C	-0.10971937	4.01229849	10.86295941
C	-0.53294120	2.69571160	11.01306945
H	-0.91898388	2.36846721	11.97236903
C	-0.47367306	1.80974352	9.95621263
H	-0.80390432	0.78278780	10.07465769
C	0.02425773	2.25685787	8.74957430
H	0.10107383	1.57420274	7.90896716
C	0.45265922	3.57141321	8.60196984
C	-0.35065618	4.88203665	12.06877661
C	0.99128131	3.86521167	7.22254767
C	1.95540991	6.84122596	10.18804489
C	3.31628222	6.47211606	10.28022919
C	4.26846954	7.24782033	10.93351860
H	5.29908461	6.91100218	10.96120624
C	3.91694181	8.43860643	11.53663197
H	4.66073758	9.04290920	12.04584524
C	2.59689716	8.83682942	11.48652503
H	2.29035394	9.75797738	11.97247588
C	1.64442794	8.05823606	10.83837561
C	3.87928745	5.26626963	9.57469142
C	0.25449288	8.64224036	10.91957132
C	-2.36865070	8.41248703	4.01273784
C	-3.02405129	9.43060475	3.29787710
C	-3.48648581	9.17707113	2.01775386
H	-3.98868323	9.95078751	1.44362104
C	-3.27921794	7.91431033	1.47282524
H	-3.63681477	7.69831681	0.47043556
C	-2.59626051	6.92610152	2.18294697
H	-2.42506264	5.95761700	1.72307057
C	-2.11620557	7.16446048	3.46220605
C	-2.34039618	10.25893464	5.32770711
C	-3.01079058	10.61359775	4.14386897
C	-3.53532667	11.88809976	4.01187593
H	-4.06451564	12.18189741	3.10959270
C	-3.38675204	12.78179221	5.06721434
H	-3.79109296	13.78586047	4.97967466
C	-2.74834894	12.40231857	6.24856679
H	-2.66963921	13.11183038	7.06658819
C	-2.22593350	11.12681452	6.40386005
B	0.82933183	6.03880409	9.38724679
H	-1.55415326	6.41127076	4.00662998
H	-1.75828183	10.81440270	7.33318233
C	-3.27099262	6.62593975	6.46520546
H	-3.93821370	7.49343123	6.41188516
H	-3.32739659	6.11908448	5.49709196
H	-3.65618806	5.94252971	7.22522810
C	0.72602523	9.79101895	5.97702716
H	0.62419947	9.71273819	4.88885045
H	0.32943057	10.76902904	6.26608326
H	1.78968278	9.77312313	6.22483126

Cbz-$^{MeO}\pi$

E = –16.969

N_{imag} = 0

S_0

F	-0.077226	4.317614	13.444876
F	0.895659	5.928136	12.337845
F	-1.303323	5.858354	12.473622
F	1.656173	4.697311	7.262080
F	-0.260805	4.013187	6.420786
F	1.283008	2.560097	6.984227
F	4.974093	4.533428	10.247195
F	2.924502	3.989673	9.727391
F	4.217211	4.966584	8.232076
F	-0.516300	7.852849	10.745772
F	0.455692	9.383185	11.970112
F	0.233229	9.701128	9.810735
N	-1.944355	8.925107	5.199875
C	0.087337	6.775836	8.243465
C	0.764789	7.736825	7.460895
C	-3.548504	10.190030	6.690333
C	0.104882	8.418305	6.439753
C	-1.266074	8.157654	6.178366
C	-1.938142	7.178895	6.939422
C	-1.261023	6.505987	7.962028
C	-0.802866	8.121842	3.093074
C	0.248679	4.593647	9.788599
C	-0.262663	4.221361	11.061285
C	-0.958038	3.018120	11.260984
C	-2.981694	9.833280	5.465124
C	-1.137403	2.115603	10.221226
C	-3.425502	10.392400	4.230642
C	-0.582878	2.404481	8.979646
C	-4.463782	11.334653	4.241537
C	0.093766	3.610084	8.768880
C	-0.176124	5.086668	12.308776
C	0.690384	3.740048	7.375084
C	2.076249	6.795505	10.031181
C	3.423190	6.343218	10.028699
C	4.488761	7.174372	10.410338
B	0.828169	6.027601	9.394260
C	4.260664	8.471719	10.849457
C	-5.037685	11.696227	5.463100
C	2.950434	8.928225	10.939585
C	-3.766031	6.664047	5.412044
C	1.886793	8.112199	10.541894
C	3.862187	4.964157	9.562539
C	0.518053	8.741747	10.754018
C	-1.711857	8.895654	3.817084
C	-2.619094	9.792774	3.181459

C	-2.592575	9.917118	1.784929
C	-4.583683	11.129507	6.671339
C	-1.679148	9.153421	1.053824
C	2.058526	9.717803	5.901342
C	-0.797632	8.264775	1.702630
O	-3.263306	6.855033	6.767163
O	0.674313	9.385894	5.658731
H	1.806068	7.950617	7.664949
H	-3.200039	9.751834	7.620543
H	-1.815158	5.762793	8.525722
H	-0.122452	7.439134	3.593087
H	-1.355144	2.789769	12.243843
H	-1.679552	1.188851	10.382744
H	-0.667629	1.688653	8.168310
H	-4.816381	11.777808	3.313221
H	5.502972	6.793131	10.366996
H	5.089780	9.109095	11.141426
H	-5.844478	12.424457	5.485264
H	2.747638	9.921177	11.328083
H	-2.973265	6.288745	4.757778
H	-4.174640	7.595140	5.011205
H	-4.559704	5.919059	5.500356
H	-3.276060	10.594428	1.278363
H	-5.046930	11.427190	7.608720
H	-1.648141	9.240389	-0.029339
H	2.296788	10.501836	5.181634
H	2.704469	8.847682	5.732345
H	2.197117	10.093078	6.922491
H	-0.098792	7.678365	1.111061

Cbz-$^{MeO}\pi$

E = –25.049

N_{imag} = 0

T₁

F	-0.322825	4.863583	13.520855
F	0.933091	6.154396	12.336077
F	-1.229712	6.296414	12.176875
F	1.202786	4.618282	7.129801
F	-0.728077	3.726148	6.705509
F	0.982127	2.473298	7.153118
F	4.894927	4.274533	9.478057
F	2.778701	3.867155	9.424767
F	3.709086	4.996640	7.818125
F	-0.227117	8.218013	10.706279
F	0.978781	9.382777	12.060199
F	0.912446	9.907881	9.959424
N	-1.983883	8.917130	5.219486
C	0.085999	6.840219	8.315867
C	0.669747	7.933845	7.643083
C	-4.147171	9.469993	6.385046
C	0.000874	8.625204	6.659523
C	-1.329526	8.218992	6.211680
C	-1.930178	7.132268	6.903020
C	-1.263760	6.483012	7.892432
C	-0.284043	8.793880	3.362788
C	0.204155	4.673816	9.876750
C	-0.228527	4.392388	11.193412
C	-0.820167	3.187418	11.556758
C	-3.284713	9.443226	5.298787
C	-1.018987	2.188247	10.625187
C	-3.603216	10.061778	4.077952
C	-0.613486	2.412446	9.326942
C	-4.825222	10.699555	3.927583
C	-0.015789	3.616135	8.965674
C	-0.198500	5.422798	12.292033
C	0.375175	3.636304	7.507537
C	2.176756	6.718013	9.969621
C	3.457240	6.123434	9.881590
C	4.614053	6.741633	10.342753
B	0.827554	6.070789	9.398714
C	4.564118	7.996276	10.917335
C	-5.705204	10.712334	5.001328
C	3.339489	8.619873	11.025448
C	-3.207498	5.972396	5.307839
C	2.180588	7.997360	10.570275
C	3.694496	4.820307	9.163865
C	0.951511	8.839711	10.815116
C	-1.483370	9.192900	3.932932
C	-2.457057	9.893428	3.203617
C	-2.209543	10.242730	1.883869
C	-5.365009	10.110100	6.214114
C	-0.998658	9.876313	1.313544
C	1.681630	10.300554	6.553262
C	-0.052923	9.153741	2.044076
O	-3.181382	6.707405	6.537538
O	0.442741	9.744599	6.094751
H	1.669965	8.249760	7.914718
H	-3.884640	9.005209	7.328595
H	-1.763561	5.633643	8.348792
H	0.447722	8.219543	3.921582
H	-1.133990	3.038857	12.584033
H	-1.480707	1.248335	10.909594
H	-0.745614	1.638829	8.576185
H	-5.084571	11.188584	2.992479
H	5.565384	6.231301	10.240834
H	5.467271	8.477287	11.278660
H	-6.667503	11.206192	4.902197
H	3.269579	9.600930	11.486017
H	-2.551635	5.096518	5.379152
H	-2.895484	6.598833	4.462848
H	-4.239713	5.653459	5.160141
H	-2.954266	10.781567	1.304328
H	-6.065475	10.144032	7.043242
H	-0.787476	10.141202	0.281659
H	1.809502	11.227756	5.997062
H	2.506729	9.614181	6.338427
H	1.627727	10.499646	7.627774
H	0.879541	8.861013	1.570556

^{Me}Cbz-π

E = –16.534

N_{imag} = 0

S₀

F	-0.134317	5.180019	13.848851
F	1.035492	6.377274	12.447777
F	-1.111426	6.793973	12.726017
F	1.017371	4.367673	7.562266
F	-1.079610	4.016146	6.986881

F	0.192572	2.342866	7.614648
F	4.515821	3.858214	10.290626
F	2.356288	3.710745	10.007753
F	3.660342	4.189984	8.296015
F	-0.097990	8.336840	10.692131
F	1.287118	9.767087	11.597058
F	0.921232	9.830943	9.434966
N	-1.889390	8.847353	5.115813
C	0.004470	6.838066	8.347341
C	0.757862	7.518637	7.364797
H	1.841856	7.532129	7.433242
C	0.143461	8.164301	6.296727
H	0.729685	8.677713	5.539883
C	-1.252437	8.171432	6.202737
C	-2.027285	7.519649	7.168192
H	-3.109758	7.532521	7.078206
C	-1.402119	6.850536	8.215340
H	-2.014979	6.337109	8.950683
C	-0.112099	4.884168	10.175630
C	-0.553062	4.794768	11.523901
C	-1.438672	3.793240	11.952569
H	-1.768629	3.781186	12.985370
C	-1.890235	2.815504	11.076199
H	-2.576380	2.045338	11.415108
C	-1.423881	2.822137	9.766786
H	-1.725921	2.038253	9.079738
C	-0.557669	3.828693	9.328136
C	-0.180735	5.785763	12.615662
C	-0.102380	3.658523	7.886955
C	2.136625	6.652609	9.956981
C	3.354047	5.919633	9.929305
C	4.600114	6.538860	10.116109
H	5.504229	5.942959	10.059722
C	4.689768	7.899242	10.379312
H	5.657350	8.371075	10.520684
C	3.518274	8.639238	10.489377
H	3.564793	9.693354	10.743145
C	2.274973	8.032512	10.284572
C	3.451760	4.430825	9.635207
C	1.094880	8.969132	10.493360
C	-1.975332	8.364964	3.791524
C	-2.678508	9.335183	3.016671
C	-2.921458	9.114837	1.654007
H	-3.457323	9.852955	1.062359
C	-2.468594	7.928852	1.081501
H	-2.641976	7.729567	0.026874
C	-1.796950	6.971674	1.864558
H	-1.470557	6.044857	1.396984
C	-1.534456	7.145060	3.232132
C	-2.526492	10.104914	5.196708
C	-3.028499	10.425826	3.900081
C	-3.706781	11.632479	3.680106
H	-4.089750	11.878478	2.692754
C	-3.870299	12.507653	4.750936
H	-4.392008	13.451227	4.611352
C	-3.349303	12.186778	6.018234
H	-3.474279	12.896713	6.833421
C	-2.660094	10.994123	6.286195
B	0.699392	6.100302	9.532738
C	-0.833474	6.045528	4.001291
H	0.174933	6.339684	4.314196
H	-1.375676	5.766926	4.909739
H	-0.743858	5.154983	3.371915
C	-2.108007	10.748687	7.674345
H	-2.634870	9.937481	8.189828
H	-1.049321	10.473579	7.655094
H	-2.213157	11.654981	8.278319

^{Me}Cbz-π

E = –24.314

N_{imag} = 0

T1

F	-0.546152	5.234226	13.599019
F	1.383245	5.619463	12.706976
F	-0.352058	6.777654	12.100833
F	0.789985	4.557811	7.122145
F	-1.255017	3.861802	6.934487
F	0.361471	2.451421	7.232152
F	4.562383	4.164068	9.952757
F	2.629237	3.785682	10.839535
F	2.783425	4.310802	8.737066
F	-0.333301	8.646587	10.325789
F	0.888191	9.906617	11.571955
F	1.072225	10.025315	9.417287
N	-1.899075	8.869111	5.090282
C	-0.014891	6.859660	8.310367
C	0.687666	7.765074	7.472705
H	1.747026	7.932504	7.650588
C	0.082656	8.430194	6.434378
H	0.647215	9.110669	5.800460
C	-1.269171	8.197116	6.166008
C	-2.003466	7.305343	6.953032
H	-3.060090	7.149612	6.745858
C	-1.387323	6.668364	8.002389
H	-1.978292	6.001480	8.625239
C	0.041006	4.770707	9.984973
C	-0.245494	4.514674	11.352835
C	-0.828541	3.340138	11.801726
H	-1.023748	3.211762	12.860879
C	-1.177946	2.341100	10.910084
H	-1.640147	1.423077	11.258464
C	-0.918131	2.536347	9.570113
H	-1.165605	1.756418	8.855889
C	-0.317362	3.709204	9.116742
C	0.060510	5.531445	12.420126
C	-0.084516	3.679365	7.624708
C	1.962688	6.779183	10.114601
C	3.167441	6.054361	10.319968
C	4.318682	6.623084	10.841643
H	5.205929	6.012180	10.968017
C	4.349776	7.963402	11.184877
H	5.251918	8.412364	11.587780
C	3.205478	8.712566	11.010448
H	3.201953	9.761286	11.292998
C	2.041207	8.140745	10.500959
C	3.274025	4.595600	9.963194
C	0.905795	9.135367	10.446909
C	-2.335084	8.264207	3.917136
C	-2.911715	9.261319	3.095982

C	-3.408185	8.947099	1.854835
H	-3.848080	9.705398	1.213717
C	-3.313485	7.617853	1.431877
H	-3.699910	7.336709	0.456626
C	-2.708381	6.655204	2.225129
H	-2.619872	5.639019	1.850420
C	-2.182438	6.931475	3.490469
C	-2.173437	10.231295	5.051627
C	-2.801945	10.518668	3.817351
C	-3.208805	11.795585	3.518034
H	-3.697242	12.026700	2.575914
C	-2.990582	12.790978	4.475311
H	-3.298144	13.811569	4.267279
C	-2.412705	12.492762	5.699703
H	-2.290329	13.283856	6.434593
C	-1.991228	11.206776	6.050004
B	0.667030	6.132328	9.476946
C	-1.493312	5.844693	4.249900
H	-0.558451	6.188565	4.703667
H	-2.104830	5.467747	5.075658
H	-1.278028	5.010189	3.578287
C	-1.434567	10.965765	7.415549
H	-1.837162	10.054702	7.869429
H	-0.347858	10.836401	7.400321
H	-1.666141	11.817495	8.059552

^{Me}Cbz-^{Me}π

E = –17.717

N

N_{imag} = 0

S_0

F	-4.520129	1.348285	7.382748
F	-2.665940	1.678218	6.279227
F	-4.606655	2.099836	5.321814
F	-1.156504	-2.575493	3.512344
F	-2.890308	-3.299207	2.363674
F	-2.295806	-4.302074	4.222263
F	0.805367	-1.518050	7.381963
F	-1.049742	-1.849283	6.280369
F	0.890182	-2.271749	5.321737
F	-2.561865	2.400877	3.510107
F	-1.422309	4.128586	4.216937
F	-0.829194	3.123565	2.359069
N	-1.860643	-0.090418	-1.735620
C	-1.859344	-0.087814	2.501867
C	-0.660778	0.054469	1.772412
H	0.280353	0.163318	2.304727
C	-0.636246	0.044116	0.376737
C	0.654885	0.188862	-0.392776
C	-1.860208	-0.089540	-0.301460
C	-3.083749	-0.222361	0.377659
C	-4.375345	-0.368084	-0.390883
C	-3.058357	-0.230990	1.773332
H	-3.999166	-0.339168	2.306355
C	-3.089558	-0.821247	4.768067
C	-4.040839	-0.210732	5.630261
C	-5.209315	-0.873111	6.038638
H	-5.918733	-0.356550	6.675465
C	-5.464483	-2.180222	5.645743
H	-6.371253	-2.685207	5.964511
C	-4.523932	-2.839706	4.863425
H	-4.681075	-3.877153	4.585961
C	-3.367418	-2.178754	4.436609
C	-3.941321	1.217494	6.142540
C	-2.423757	-3.063047	3.636226
C	-0.627624	0.648554	4.766215
C	0.324451	0.039085	5.628255
C	1.493211	0.702032	6.034903
H	2.203212	0.186258	6.671721
C	1.747913	2.008727	5.640319
H	2.654908	2.514157	5.957741
C	0.806647	2.667240	4.858033
H	0.963463	3.704381	4.579240
C	-0.350159	2.005694	4.432954
C	0.225489	-1.388601	6.142119
C	-1.294610	2.888881	3.632298
C	-1.677927	-1.225213	-2.550580
C	-1.744975	-0.805935	-3.913087
C	-1.595522	-1.739482	-4.948312
H	-1.646362	-1.424699	-5.987763
C	-1.383070	-3.074841	-4.613979
H	-1.263081	-3.821186	-5.395390
C	-1.326053	-3.472145	-3.264474
H	-1.165071	-4.522829	-3.031373
C	-1.472068	-2.575919	-2.195063
C	-2.043800	1.043377	-2.551865
C	-1.977527	0.622435	-3.913897
C	-2.127573	1.554719	-4.950175
H	-2.077343	1.238667	-5.989272
C	-2.339809	2.890488	-4.617352
H	-2.460221	3.635884	-5.399605
C	-2.396057	3.289439	-3.268300
H	-2.556892	4.340409	-3.036390
C	-2.249454	2.394516	-2.197880
B	-1.858844	-0.086813	4.061334
C	-1.413395	-3.087299	-0.771434
H	-0.581950	-2.650831	-0.207486
H	-2.325400	-2.856533	-0.212695
H	-1.284582	-4.173762	-0.773599
C	-2.307355	2.907606	-0.774839
H	-3.138516	2.471850	-0.209918
H	-1.395061	2.677458	-0.216319
H	-2.436118	3.994072	-0.778228
H	1.501215	0.307952	0.289244
H	0.619838	1.058342	-1.060156
H	0.840697	-0.686167	-1.027114
H	-4.561430	0.506023	-1.026405
H	-5.221288	-0.486075	0.291809
H	-4.340782	-1.238559	-1.056994

^{Me}Cbz-^{Me}π

E = –25.605

N_{imag} = 0

T_1

F	-4.681789	1.389005	7.024675
F	-2.528733	1.196107	7.021582
F	-3.567485	1.887700	5.241070
F	-1.287640	-2.688968	3.011625
F	-3.227681	-3.310760	2.261543

F	-2.274164	-4.397539	3.875814
F	0.949633	-1.557317	7.037056
F	-1.203578	-1.365968	7.025504
F	-0.157595	-2.059056	5.249853
F	-2.431953	2.511094	3.004152
F	-1.452096	4.222267	3.870819
F	-0.489378	3.134810	2.262419
N	-1.848521	-0.090392	-1.766987
C	-1.856657	-0.089077	2.460905
C	-0.690515	0.200764	1.714510
H	0.236795	0.408714	2.245778
C	-0.660499	0.217162	0.336614
C	0.606132	0.503204	-0.415266
C	-1.850246	-0.090580	-0.343265
C	-3.043201	-0.397812	0.331624
C	-4.306931	-0.684652	-0.424927
C	-3.019342	-0.379804	1.709557
H	-3.949134	-0.586721	2.236875
C	-2.953070	-0.950364	4.747339
C	-3.774678	-0.427996	5.784646
C	-4.763349	-1.165423	6.418361
H	-5.356906	-0.702341	7.199492
C	-5.010703	-2.477295	6.050345
H	-5.788343	-3.054386	6.540752
C	-4.240240	-3.035489	5.050925
H	-4.403677	-4.069831	4.761737
C	-3.233915	-2.303553	4.422452
C	-3.631172	0.995198	6.255068
C	-2.487607	-3.133088	3.404170
C	-0.772204	0.777642	4.750389
C	0.045920	0.257436	5.791350
C	1.031451	0.996687	6.427833
H	1.622574	0.535238	7.211771
C	1.278691	2.308360	6.059027
H	2.053825	2.886960	6.551619
C	0.511494	2.864435	5.055912
H	0.674957	3.898547	4.765910
C	-0.491558	2.130514	4.424532
C	-0.098351	-1.165285	6.262988
C	-1.234518	2.957567	3.401768
C	-1.800530	-1.224984	-2.563773
C	-1.823057	-0.817870	-3.919474
C	-1.760775	-1.749359	-4.927408
H	-1.770903	-1.453745	-5.972742
C	-1.669374	-3.098730	-4.566084
H	-1.621041	-3.857846	-5.341488
C	-1.620266	-3.485769	-3.234498
H	-1.526241	-4.540966	-2.991432
C	-1.675882	-2.573345	-2.176504
C	-1.901962	1.043957	-2.564226
C	-1.887779	0.635910	-3.919718
C	-1.959056	1.566452	-4.927955
H	-1.956129	1.269918	-5.973075
C	-2.051080	2.915899	-4.567121
H	-2.106731	3.674272	-5.342758
C	-2.092246	3.303755	-3.235566
H	-2.187520	4.358863	-2.992601
C	-2.027535	2.392219	-2.177209
B	-1.860757	-0.087501	3.998392
C	-1.587007	-3.061644	-0.767537
H	-0.859964	-2.490171	-0.179905
H	-2.540035	-2.962521	-0.235095
H	-1.301629	-4.116583	-0.761481
C	-2.109524	2.881477	-0.768164
H	-2.833464	2.310116	-0.176552
H	-1.153902	2.783181	-0.240349
H	-2.395379	3.936281	-0.761382
H	1.435523	0.642772	0.282063
H	0.524866	1.411620	-1.025881
H	0.869602	-0.315732	-1.096535
H	-4.566208	0.132039	-1.110495
H	-5.139544	-0.820117	0.269365
H	-4.224661	-1.595677	-1.031478

Phox-$^{Me}\pi$

E = –16.752

N_{imag} = 0

S_0

F	-0.103865	3.949696	13.257065
F	0.782451	5.712838	12.323536
F	-1.408254	5.471017	12.359404
F	1.850126	4.942776	7.222398
F	-0.008846	4.278022	6.244122
F	1.576368	2.834797	6.707377
F	5.013406	4.791067	10.381974
F	3.039394	4.142521	9.711099
F	4.335210	5.303260	8.357296
F	-0.715432	7.655695	10.829521
F	0.077972	9.218913	12.137751
F	-0.092049	9.592114	9.983043
H	-4.042515	7.772735	6.759968
C	0.068524	6.824827	8.274100
C	0.704958	7.858040	7.557122
H	1.735190	8.115325	7.789054
C	0.059584	8.554592	6.533328
C	-1.278206	8.221600	6.254951
C	-1.963882	7.212521	6.954672
C	-1.266508	6.516255	7.943993
H	-1.782914	5.726049	8.482816
C	0.333887	4.548436	9.652404
C	-0.194664	4.036451	10.867699
C	-0.803977	2.773471	10.940436
H	-1.218091	2.435847	11.884043
C	-0.875471	1.949785	9.825115
H	-1.350347	0.975450	9.888675
C	-0.299565	2.380314	8.634971
H	-0.299547	1.729605	7.766533
C	0.290255	3.645185	8.550464
C	-0.219273	4.800151	12.182533
C	0.922814	3.941443	7.198458
C	1.974806	6.854908	10.146773
C	3.349563	6.499804	10.188050
C	4.330488	7.377570	10.676804
H	5.371259	7.073461	10.663572
C	3.985192	8.623404	11.183162
H	4.749540	9.297621	11.557396
C	2.641824	8.979042	11.232595
H	2.347953	9.927042	11.671462
C	1.662431	8.117902	10.728607
C	3.911369	5.188553	9.662067

C	0.240007	8.628007	10.906119
H	-3.504716	6.579126	5.578518
H	-3.777173	6.089750	7.265104
B	0.819237	6.047596	9.398210
H	0.236825	10.601976	5.865507
H	1.787900	9.765830	6.096111
C	-3.402421	6.892797	6.624398
C	0.756989	9.643125	5.752632
H	0.773297	9.410588	4.681167
H	-0.737066	6.727370	4.295081
N	-1.969968	8.938763	5.218211
O	-3.329940	10.352736	3.175989
C	-3.337023	10.812508	4.494596
C	-2.665873	10.121576	5.524362
C	-1.265404	6.848624	2.202396
C	-1.944441	7.569883	1.220265
C	-2.629293	8.743119	1.573150
C	-2.628245	9.179284	2.891823
C	-1.945922	8.461714	3.895721
C	-1.265266	7.290457	3.532351
C	-4.035863	11.984427	4.754020
C	-2.718941	10.646478	6.823915
H	-0.730714	5.938111	1.945350
H	-1.948481	7.233217	0.187418
H	-3.168508	9.328387	0.832954
C	-4.082970	12.500767	6.058321
C	-3.423680	11.828712	7.087687
H	-4.631133	13.417636	6.255768
H	-3.450416	12.215648	8.102855
H	-2.205731	10.125814	7.626007
H	-4.538116	12.482991	3.929181

Phox-$^{Me}\pi$
E = –24.706
N_{imag} = 0
T_1

F	-0.458597	4.298177	13.168078
F	1.403975	5.183337	12.526562
F	-0.488611	6.080460	11.949914
F	1.379861	4.961017	6.810661
F	-0.470094	3.939227	6.326243
F	1.369158	2.822550	6.568827
F	4.965704	4.833316	9.895749
F	3.098672	3.954080	10.532308
F	3.274470	4.833577	8.553714
F	-0.746794	8.187202	10.488548
F	0.127158	9.438488	12.005903
F	0.396728	9.938292	9.916343
H	-4.019862	7.432000	6.414014
C	0.045190	6.854001	8.245544
C	0.590604	8.006401	7.638197
H	1.569654	8.358455	7.957104
C	-0.051398	8.713434	6.640957
C	-1.304404	8.249287	6.225097
C	-1.910339	7.116367	6.779662
C	-1.222701	6.448835	7.773680
H	-1.694712	5.572520	8.213509
C	0.423149	4.565138	9.590677
C	0.112899	4.035737	10.874507
C	-0.252622	2.715653	11.086232
H	-0.480909	2.379302	12.091497
C	-0.347513	1.830545	10.026121
H	-0.640850	0.798505	10.189028
C	-0.052279	2.289781	8.759587
H	-0.099154	1.606494	7.916782
C	0.332737	3.611285	8.543685
C	0.145513	4.898534	12.107480
C	0.662898	3.868677	7.092849
C	1.883936	6.848092	10.197942
C	3.196516	6.338976	10.404915
C	4.177372	7.023404	11.105261
H	5.159549	6.578783	11.221192
C	3.920955	8.274546	11.639299
H	4.690102	8.815175	12.181336
C	2.662296	8.813383	11.473060
H	2.433273	9.784069	11.902867
C	1.667426	8.121861	10.785199
C	3.617196	5.004964	9.848786
C	0.359939	8.877188	10.781743
H	-3.235214	6.359790	5.249849
H	-3.580569	5.783584	6.884974
B	0.788365	6.084617	9.352080
H	-0.041602	10.828028	6.197097
H	1.555772	10.111670	6.442795
C	-3.256454	6.651698	6.307147
C	0.565449	9.931725	6.019216
H	0.677594	9.825020	4.933327
H	-0.529731	6.944319	4.279033
N	-1.997516	8.965363	5.181818
O	-3.335158	10.347225	3.166733
C	-3.466282	10.727800	4.463472
C	-2.798942	10.041035	5.494420
C	-0.950555	7.136430	2.176396
C	-1.639619	7.852920	1.188949
C	-2.435572	8.925763	1.528875
C	-2.545342	9.285393	2.862966
C	-1.861359	8.576308	3.867537
C	-1.055205	7.488311	3.500269
C	-4.281660	11.815101	4.735702
C	-2.970393	10.472834	6.818278
H	-0.326894	6.294185	1.895336
H	-1.546818	7.562750	0.147342
H	-2.980045	9.499794	0.785981
C	-4.435945	12.224351	6.042909
C	-3.779546	11.551349	7.081881
H	-5.071919	13.074653	6.267123
H	-3.909896	11.883443	8.106595
H	-2.453098	9.939808	7.609981
H	-4.777942	12.316123	3.910754

Phox-$^{MeO}\pi$
E = –17.177
N_{imag} = 0
S_0

F	-0.077226	4.317614	13.444876
F	0.895659	5.928136	12.337845
F	-1.303323	5.858354	12.473622
F	1.656173	4.697311	7.262080
F	-0.260805	4.013187	6.420786

F	1.283008	2.560097	6.984227
F	4.974093	4.533428	10.247195
F	2.924502	3.989673	9.727391
F	4.217211	4.966584	8.232076
F	-0.516300	7.852849	10.745772
F	0.455692	9.383185	11.970112
F	0.233229	9.701128	9.810735
N	-1.944355	8.925107	5.199875
C	0.087337	6.775836	8.243465
C	0.764789	7.736825	7.460895
C	-3.548504	10.190030	6.690333
C	0.104882	8.418305	6.439753
C	-1.266074	8.157654	6.178366
C	-1.938142	7.178895	6.939422
C	-1.261023	6.505987	7.962028
C	-0.802866	8.121842	3.093074
C	0.248679	4.593647	9.788599
C	-0.262663	4.221361	11.061285
C	-0.958038	3.018120	11.260984
C	-2.981694	9.833280	5.465124
C	-1.137403	2.115603	10.221226
C	-3.425502	10.392400	4.230642
C	-0.582878	2.404481	8.979646
C	-4.463782	11.334653	4.241537
C	0.093766	3.610084	8.768880
C	-0.176124	5.086668	12.308776
C	0.690384	3.740048	7.375084
C	2.076249	6.795505	10.031181
C	3.423190	6.343218	10.028699
C	4.488761	7.174372	10.410338
B	0.828169	6.027601	9.394260
C	4.260664	8.471719	10.849457
C	-5.037685	11.696227	5.463100
C	2.950434	8.928225	10.939585
C	-3.766031	6.664047	5.412044
C	1.886793	8.112199	10.541894
C	3.862187	4.964157	9.562539
C	0.518053	8.741747	10.754018
C	-1.711857	8.895654	3.817084
C	-2.619094	9.792774	3.181459
C	-2.592575	9.917118	1.784929
C	-4.583683	11.129507	6.671339
C	-1.679148	9.153421	1.053824
C	2.058526	9.717803	5.901342
C	-0.797632	8.264775	1.702630
O	-3.263306	6.855033	6.767163
O	0.674313	9.385894	5.658731
H	1.806068	7.950617	7.664949
H	-3.200039	9.751834	7.620543
H	-1.815158	5.762793	8.525722
H	-0.122452	7.439134	3.593087
H	-1.355144	2.789769	12.243843
H	-1.679552	1.188851	10.382744
H	-0.667629	1.688653	8.168310
H	-4.816381	11.777808	3.313221
H	5.502972	6.793131	10.366996
H	5.089780	9.109095	11.141426
H	-5.844478	12.424457	5.485264
H	2.747638	9.921177	11.328083
H	-2.973265	6.288745	4.757778
H	-4.174640	7.595140	5.011205
H	-4.559704	5.919059	5.500356
H	-3.276060	10.594428	1.278363
H	-5.046930	11.427190	7.608720
H	-1.648141	9.240389	-0.029339
H	2.296788	10.501836	5.181634
H	2.704469	8.847682	5.732345
H	2.197117	10.093078	6.922491
H	-0.098792	7.678365	1.111061

Phox-$^{MeO}\pi$
E = –25.096
N_{imag} = 0
T_1

F	-0.421493	4.623636	13.229316
F	1.472808	5.329360	12.463077
F	-0.375913	6.327871	11.902038
F	1.129650	4.798071	6.757985
F	-0.732186	3.733652	6.419991
F	1.120486	2.644275	6.707027
F	4.878991	4.553676	9.648234
F	2.991053	3.843865	10.425723
F	3.116896	4.625165	8.399984
F	-0.529056	8.375733	10.335763
F	0.478275	9.505093	11.869058
F	0.743417	10.046934	9.786439
H	-3.031231	9.384818	7.530342
C	0.037565	6.851957	8.160569
C	0.611785	7.983434	7.533836
H	1.599117	8.309869	7.840155
C	-0.056883	8.681879	6.551718
C	-1.327217	8.253331	6.131347
C	-1.915027	7.123941	6.725343
C	-1.248872	6.451738	7.727260
H	-1.716884	5.592008	8.193098
C	0.327790	4.609277	9.610245
C	0.036021	4.181767	10.936214
C	-0.403744	2.903636	11.246173
H	-0.611040	2.646496	12.279491
C	-0.596542	1.961551	10.249576
H	-0.946545	0.962499	10.489685
C	-0.323743	2.321356	8.946183
H	-0.445612	1.591636	8.150263
C	0.133939	3.600415	8.631752
C	0.179210	5.112938	12.110822
C	0.427443	3.733168	7.156302
C	1.971679	6.805051	10.025717
C	3.254053	6.207451	10.184774
C	4.319285	6.841239	10.806490
H	5.271124	6.327531	10.889635
C	4.184056	8.126363	11.304625
H	5.018712	8.626419	11.785818
C	2.960156	8.750680	11.182802
H	2.823836	9.749959	11.587386
C	1.881444	8.111096	10.573141
C	3.545641	4.824028	9.666762
C	0.635872	8.963500	10.624107
H	-6.177433	11.987229	6.185682
H	-4.993624	10.809711	8.023571

B	0.781738	6.086848	9.268901
H	-0.519606	8.677926	0.078447
H	-2.467531	10.093134	0.732198
H	-5.370353	11.749607	3.836851
C	-5.317525	11.362810	5.963500
C	-4.650560	10.696390	6.999971
H	0.002741	7.501779	4.180076
N	-2.018271	8.963496	5.106198
O	-3.370114	10.356097	3.099338
C	-3.781376	10.438627	4.391398
C	-3.108700	9.751163	5.417687
C	-0.140124	8.004933	2.094319
C	-0.829099	8.735913	1.117473
C	-1.906175	9.523141	1.466165
C	-2.292179	9.586664	2.797099
C	-1.603140	8.870786	3.792663
C	-0.518269	8.066841	3.414103
C	-4.882394	11.238324	4.660989
C	-3.560522	9.901933	6.736684
H	0.699465	7.379101	1.808448
O	0.412561	9.794284	5.922941
O	-3.138680	6.785843	6.233756
C	1.669065	10.302637	6.368006
C	-3.739641	5.604575	6.761821
H	1.848144	11.208221	5.787480
H	2.471427	9.578957	6.181963
H	1.633537	10.541885	7.437016
H	-3.090274	4.735819	6.603999
H	-4.675748	5.475946	6.217505
H	-3.946427	5.716514	7.832702

Cbz-$^{FMe}\pi$

E = –16.608

$\boldsymbol{N}_{\mathbf{imag}}$ = 0

$\mathbf{S_0}$

F	-0.062783	3.984874	13.304833
F	0.847122	5.721206	12.343431
F	-1.347427	5.528980	12.417803
F	1.792900	4.924535	7.248215
F	-0.106066	4.325728	6.302863
F	1.450084	2.832773	6.703054
F	5.010618	4.709698	10.348372
F	3.012119	4.093451	9.719782
F	4.304679	5.216825	8.331974
F	-0.647272	7.668443	10.836230
F	0.163462	9.267340	12.092551
F	-0.015007	9.574914	9.928219
N	-1.976450	8.945511	5.213158
C	0.071439	6.826543	8.293018
C	0.717119	7.823507	7.544385
H	1.755134	8.058437	7.747774
C	0.060144	8.514258	6.526568
C	-1.294231	8.241567	6.244690
C	-1.958209	7.255195	7.002440
C	-1.273864	6.556085	7.996313
H	-1.803084	5.793848	8.555753
C	0.316596	4.555071	9.687926
C	-0.192390	4.057878	10.917681
C	-0.817981	2.804383	11.007585
H	-1.216037	2.476179	11.961227
C	-0.925900	1.977521	9.897323
H	-1.413094	1.010569	9.976004
C	-0.372092	2.394357	8.691811
H	-0.400350	1.740428	7.826359
C	0.232664	3.650175	8.589660
C	-0.177994	4.830007	12.228788
C	0.839386	3.941946	7.226202
C	2.009930	6.832737	10.137207
C	3.376918	6.447629	10.168155
C	4.378557	7.309635	10.641623
H	5.412698	6.984362	10.622240
C	4.063135	8.566394	11.140929
H	4.844669	9.226884	11.503566
C	2.728368	8.952450	11.197554
H	2.457684	9.909690	11.631013
C	1.728383	8.108125	10.707445
C	3.905586	5.120562	9.644842
C	0.315679	8.641587	10.883626
C	-2.042482	8.552800	3.862857
C	-2.809805	9.518137	3.151393
C	-3.017970	9.341773	1.775799
H	-3.600354	10.064820	1.209940
C	-2.466688	8.224493	1.144280
H	-2.620938	8.076702	0.078506
C	-1.711260	7.282486	1.870981
H	-1.292812	6.420767	1.357179
C	-1.488331	7.433504	3.242434
C	-2.690743	10.147626	5.375191
C	-3.220592	10.529131	4.109950
C	-3.976631	11.706511	4.016145
H	-4.391805	12.019547	3.061262
C	-4.187875	12.470215	5.166433
H	-4.772263	13.384782	5.106707
C	-3.652554	12.071207	6.407557
H	-3.830579	12.682393	7.288678
C	-2.895214	10.902914	6.529553
B	0.831352	6.037605	9.421553
H	-0.906215	6.706105	3.800723
H	-2.483168	10.597610	7.486955
C	-3.419084	6.922231	6.736493
F	-4.238642	8.000922	6.920352
F	-3.621712	6.473101	5.460350
F	-3.872708	5.937648	7.573627
C	0.820880	9.572098	5.740499
F	0.884688	9.280418	4.406245
F	0.251694	10.810385	5.857152
F	2.113177	9.693591	6.177769

Cbz-$^{FMe}\pi$

E = –24.719

$\boldsymbol{N}_{\mathbf{imag}}$ = 0

$\mathbf{T_1}$

F	-0.318615	4.384851	13.362934
F	0.768417	5.922559	12.312934
F	-1.400970	5.843546	12.187416
F	1.674225	4.698216	7.179546
F	-0.283318	4.096038	6.458774
F	1.240526	2.612727	6.857078

F	4.979022	4.569019	9.981457
F	2.930282	4.030880	9.579345
F	4.056236	5.192599	8.126541
F	-0.529970	7.913691	10.856553
F	0.479472	9.399803	12.045506
F	0.316080	9.672541	9.905534
N	-1.978966	8.953544	5.207993
C	0.088614	6.805832	8.293483
C	0.661285	7.925097	7.624684
H	1.661867	8.248163	7.895317
C	0.013750	8.613416	6.633550
C	-1.290010	8.237419	6.232833
C	-1.891697	7.131951	6.879207
C	-1.220123	6.450437	7.858964
H	-1.724207	5.611609	8.329132
C	0.275869	4.586885	9.744423
C	-0.214013	4.171304	11.001539
C	-0.767477	2.913378	11.220246
H	-1.136001	2.660308	12.208451
C	-0.854203	1.992485	10.196612
H	-1.288583	1.012766	10.367382
C	-0.360192	2.339294	8.955907
H	-0.389316	1.621858	8.141978
C	0.195889	3.595291	8.738562
C	-0.277114	5.082626	12.199270
C	0.714347	3.775069	7.330091
C	2.055381	6.789203	10.080934
C	3.389525	6.327541	10.096237
C	4.441442	7.069727	10.624646
H	5.446035	6.661897	10.595067
C	4.219658	8.313418	11.179078
H	5.041118	8.892797	11.588003
C	2.927509	8.795510	11.217914
H	2.722498	9.757717	11.676404
C	1.875916	8.052083	10.692979
C	3.818239	5.034404	9.453363
C	0.534521	8.727846	10.863422
C	-1.953798	8.646438	3.862560
C	-2.752897	9.575095	3.159556
C	-2.871429	9.459421	1.797078
H	-3.475553	10.152988	1.219843
C	-2.187249	8.413681	1.155800
H	-2.273285	8.311293	0.078145
C	-1.403091	7.506599	1.863724
H	-0.889774	6.711780	1.332927
C	-1.270665	7.607031	3.240115
C	-2.776494	10.063082	5.406041
C	-3.287284	10.495223	4.162250
C	-4.112622	11.591155	4.122083
H	-4.526547	11.954904	3.186371
C	-4.415558	12.240951	5.329940
H	-5.067193	13.109424	5.311397
C	-3.903764	11.801740	6.547985
H	-4.160459	12.329911	7.460192
C	-3.068776	10.696470	6.609091
B	0.814993	6.051960	9.385138
H	-0.666934	6.913184	3.817356
H	-2.654641	10.333001	7.544796
C	-3.267465	6.700498	6.487996
F	-4.179467	7.704231	6.624679
F	-3.332853	6.328070	5.178425
F	-3.722439	5.663339	7.206719
C	0.688237	9.773050	5.975992
F	0.785142	9.613519	4.625817
F	-0.003024	10.934183	6.154501
F	1.931319	9.990902	6.430483

^{MeO_3}Ph-^{FMe}π

E = –14.741

N_{imag} = 0

S_0

F	-0.035622	3.960144	13.298349
F	0.857466	5.711098	12.347748
F	-1.335354	5.505068	12.435178
F	1.779399	4.961809	7.234423
F	-0.117661	4.348259	6.295232
F	1.453322	2.868540	6.687061
F	5.019159	4.739187	10.305313
F	3.019137	4.119008	9.685737
F	4.294148	5.266095	8.300900
F	-0.652925	7.664890	10.869514
F	0.162703	9.242406	12.149579
F	-0.030026	9.588435	9.992085
F	0.203915	10.829738	5.853428
C	0.052005	6.845421	8.312918
C	0.682127	7.856291	7.570122
H	1.714864	8.108215	7.779865
C	0.017908	8.533064	6.547424
C	-1.333623	8.249507	6.240690
C	-1.972017	7.244918	7.004946
C	-1.286596	6.555680	8.004888
H	-1.806950	5.783382	8.558692
C	0.319965	4.565634	9.685466
C	-0.181646	4.055400	10.912822
C	-0.802674	2.799030	10.994806
H	-1.195487	2.461343	11.947339
C	-0.912899	1.981563	9.877907
H	-1.396996	1.012527	9.950010
C	-0.365100	2.410685	8.674020
H	-0.395040	1.764149	7.803066
C	0.235282	3.669414	8.580032
C	-0.163114	4.815244	12.230900
C	0.835510	3.971734	7.215774
C	2.004535	6.847531	10.144313
C	3.374012	6.469925	10.159014
C	4.375134	7.331613	10.634150
H	5.410827	7.012336	10.601926
C	4.057262	8.580626	11.151079
H	4.838313	9.241214	11.514657
C	2.720958	8.958264	11.223651
H	2.448539	9.909014	11.670172
C	1.721269	8.114014	10.732511
C	3.905712	5.152429	9.615771
C	0.307678	8.639414	10.926556
F	2.067488	9.734198	6.218303
B	0.822895	6.055156	9.429306
C	0.780908	9.593426	5.766459
F	0.871954	9.285128	4.432728
C	-3.421250	6.869687	6.730240
F	-4.279166	7.922352	6.881872

F	-3.593701	6.390619	5.455364
F	-3.862945	5.883617	7.573665
O	-1.391765	7.355865	3.462049
C	-2.054860	8.977744	5.152568
C	-2.760826	10.164781	5.429157
C	-3.438501	10.861714	4.428854
C	-3.411977	10.362770	3.121700
C	-2.724060	9.188704	2.808487
C	-2.051852	8.506800	3.827151
O	-2.744292	10.597991	6.734848
H	-3.980019	11.777367	4.651114
O	-4.095791	11.085402	2.165649
H	-2.700497	8.795547	1.795668
H	-0.962193	6.958276	4.241618
H	-3.261283	11.420720	6.803675
H	-4.003141	10.640935	1.303359

^{MeO_3}Ph-^{FMe}π

E = –22.725

$\boldsymbol{N_{imag}}$ = 0

T_1

F	-0.323606	4.367504	13.350473
F	0.742211	5.921377	12.309054
F	-1.422577	5.812760	12.180279
F	1.689743	4.729391	7.185510
F	-0.242343	4.074868	6.453002
F	1.313144	2.634900	6.870412
F	4.987843	4.617780	9.969902
F	2.948851	4.065382	9.555125
F	4.068994	5.248011	8.119431
F	-0.551613	7.888235	10.860434
F	0.435554	9.355562	12.085567
F	0.284748	9.670560	9.953512
F	-0.086625	10.927899	6.193866
C	0.080840	6.804185	8.299151
C	0.632856	7.941141	7.643896
H	1.617172	8.293948	7.935327
C	-0.011136	8.603364	6.639210
C	-1.292909	8.193395	6.172537
C	-1.875902	7.086200	6.852103
C	-1.214007	6.424576	7.845728
H	-1.709877	5.577711	8.309651
C	0.286155	4.585034	9.742137
C	-0.211060	4.163662	10.991858
C	-0.753218	2.901049	11.202724
H	-1.128728	2.642598	12.186318
C	-0.818539	1.981584	10.178333
H	-1.243736	0.997385	10.343095
C	-0.315127	2.335035	8.944875
H	-0.327634	1.618999	8.129947
C	0.227725	3.596368	8.735237
C	-0.290428	5.069347	12.191908
C	0.754420	3.783367	7.331511
C	2.045619	6.801609	10.088966
C	3.383561	6.357818	10.094023
C	4.426125	7.107186	10.627093
H	5.435456	6.713357	10.589937
C	4.189524	8.339232	11.197322
H	5.003972	8.924310	11.610799
C	2.892390	8.802466	11.246140
H	2.675418	9.755868	11.716159
C	1.850864	8.052483	10.714715
C	3.827466	5.075862	9.441148
C	0.502721	8.711652	10.891079
F	1.870918	10.033294	6.445661
B	0.811068	6.057405	9.387990
C	0.632274	9.788926	6.002378
F	0.717701	9.648914	4.646497
C	-3.242271	6.642723	6.449740
F	-4.180566	7.605480	6.659697
F	-3.307272	6.371646	5.112760
F	-3.662475	5.544513	7.087841
O	-1.159008	7.624962	3.462570
C	-2.043195	8.979147	5.191815
C	-2.881858	10.075941	5.557297
C	-3.552991	10.773670	4.594858
C	-3.409808	10.397659	3.234368
C	-2.608720	9.336369	2.818656
C	-1.934914	8.637589	3.784339
O	-2.943745	10.340027	6.861384
H	-4.198614	11.613230	4.833557
O	-4.096793	11.131268	2.376380
H	-2.517411	9.070018	1.770699
H	-0.768695	7.260328	4.293973
H	-3.527088	11.096002	7.042798
H	-3.963271	10.830017	1.459199

References

[1] a) C. Fonseca Guerra, J. G. Snijders, G. te Velde, E. J. Baerends, *Theor. Chem. Acc.* **1998**, *99*, 391; b) G. te Velde, F. M. Bickelhaupt, E. J. Baerends, C. Fonseca Guerra, S. J. A. van Gisbergen, J. G. Snijders, T. Ziegler, *J. Comput. Chem.* **2001**, *22*, 931; c) E. J. Baerends, T. Ziegler, A. J. Atkins, J. Autschbach, O. Baseggio, D. Bashford, A. Bérces, F. M. Bickelhaupt, C. Bo, P. M. Boerrigter, L. Cavallo, C. Daul, D. P. Chong, D. V. Chulhai, L. Deng, R. M. Dickson, J. M. Dieterich, D. E. Ellis, M. van Faassen, L. Fan, T. H. Fischer, C. Fonseca Guerra, M. Franchini, A. Ghysels, A. Giammona, S. J. A. van Gisbergen, A. Goez, A. W. Götz, J. A. Groeneveld, O. V. Gritsenko, M. Grüning, S. Gusarov, F. E. Harris, P. van den Hoek, Z. Hu, C. R. Jacob, H. Jacobsen, L. Jensen, L. Joubert, J. W. Kaminski, G. van Kessel, C. König, F. Kootstra, A. Kovalenko, M. V. Krykunov, E. van Lenthe, D. A. McCormack, A. Michalak, M. Mitoraj, S. M. Morton, J. Neugebauer, V. P. Nicu, L. Noodleman, V. P. Osinga, S. Patchkovskii, M. Pavanello, C. A. Peeples, P. H. T. Philipsen, D. Post, C. C. Pye, H. Ramanantoanina, P. Ramos, W. Ravenek, J. I. Rodríguez, P. Ros, R. Rüger, P. R. T. Schipper, D. Schlüns, H. van Schoot, G. Schreckenbach, J. S. Seldenthuis, M. Seth, J. G. Snijders, M. Solà, M. Stener, M. Swart, D. Swerhone, V. Tognetti, G. te Velde, P. Vernooijs, L. Versluis, L. Visscher, O. Visser, F. Wang, T. A. Wesolowski, E. M. van Wezenbeek, G. Wiesenekker, S. K. Wolff, T. K. Woo, A. L. Yakovlev, ADF2018, SCM, Theoretical Chemistry, Vrije Universiteit, http://www.scm.com, Amsterdam, The Netherlands, **2018**.

[2] a) J. P. Perdew, *Phys. Rev. B* **1986**, *33*, 8822; b) J. P. Perdew, *Phys. Rev. B* **1986**, *34*, 7406; c) A. D. Becke, *Phys. Rev. A* **1988**, *38*, 3098; d) B. G. Johnson, P. M. W. Gill, J. A. Pople, *J. Chem. Phys.* **1993**, *98*, 5612; e) S. Grimme, *Chem. Eur. J.* **2004**, *10*, 3423; f) S. Grimme, S. Ehrlich, L. Goerigk, *J. Comput. Chem.* **2011**, *32*, 1456.

[3] A. Rosa, E. J. Baerends, S. J. A. van Gisbergen, E. van Lenthe, J. A. Groeneveld, J. G. Snijders, *J. Am. Chem. Soc.* **1999**, *121*, 10356.

[4] a) A. Berces, R. M. Dickson, L. Y. Fan, H. Jacobsen, D. Swerhone, T. Ziegler, *Comput. Phys. Commun.* **1997**, *100*, 247; b) H. Jacobsen, A. Berces, D. P. Swerhone, T. Ziegler, *Comput. Phys. Commun.* **1997**, *100*, 263; c) S. K. Wolff, *Int. J. Quantum Chem.* **2005**, *104*, 645.

[5] a) J. F. Janak, *Phys. Rev. B* **1978**, *18*, 7165; b) J. P. Perdew, M. Levy, *Phys. Rev. Lett.* **1983**, *51*, 1884; c) L. Kleinman, *Phys. Rev. B* **1997**, *56*, 16029.

[6] a) R. Baer, E. Livshits, U. Salzner, *Annu. Rev. Phys. Chem.* **2010**, *61*, 85; b) L. Kronik, T. Stein, S. Refaely-Abramson, R. Baer, *J. Chem. Theory Comput.* **2012**, *8*, 1515; c) H. Sun, J. Autschbach, *ChemPhysChem* **2013**, *14*, 2450; d) T. J. Penfold, *J. Phys. Chem. C* **2015**, *119*, 13535; e) H. Sun, S. Zhang, C. Zhong, Z. Sun, *J. Comput. Chem.* **2016**, *37*, 684.

[7] a) T. Korzdorfer, J. L. Brédas, *Acc. Chem. Res.* **2014**, *47*, 3284; b) H. Sun, C. Zhong, J. L. Brédas, *J. Chem. Theory Comput.* **2015**, *11*, 3851.

[8] F. Zapata, L. Ridder, J. Hidding, C. R. Jacob, I. Infante, L. Visscher, *J. Chem. Inf. Model.* **2019**, *59*, 3191.

[9] a) S. Hirata, M. Head-Gordon, *Chem. Phys. Lett.* **1999**, *314*, 291; b) A. Chantzis, A. D. Laurent, C. Adamo, D. Jacquemin, *J. Chem. Theory Comput.* **2013**, *9*, 4517.

[10] R. L. Martin, *J. Chem. Phys.* **2003**, *118*, 4775.

[11] a) M. J. Peach, P. Benfield, T. Helgaker, D. J. Tozer, *J. Chem. Phys.* **2008**, *128*, 044118; b) M. J. Peach, D. J. Tozer, *J. Phys. Chem. A* **2012**, *116*, 9783.

[12] C. A. Guido, P. Cortona, B. Mennucci, C. Adamo, *J. Chem. Theory Comput.* **2013**, *9*, 3118.

[13] a) P. K. Samanta, D. Kim, V. Coropceanu, J. L. Brédas, *J. Am. Chem. Soc.* **2017**, *139*, 4042; b) Y. Olivier, B. Yurash, L. Muccioli, G. D'Avino, O. Mikhnenko, J. C. Sancho-Garcia, C. Adachi, T. Q. Nguyen, D. Beljonne, *Phys. Rev. Mater.* **2017**, *1*, 075602.

[14] S. Huang, Q. Zhang, Y. Shiota, T. Nakagawa, K. Kuwabara, K. Yoshizawa, C. Adachi, *J. Chem. Theory Comput.* **2013**, *9*, 3872.

[15] a) F. B. Dias, K. N. Bourdakos, V. Jankus, K. C. Moss, K. T. Kamtekar, V. Bhalla, J. Santos, M. R. Bryce, A. P. Monkman, *Adv. Mater.* **2013**, *25*, 3707; b) J. Gibson, T. J. Penfold, *Phys. Chem. Chem. Phys.* **2017**, *19*, 8428; c) P. L. dos Santos, M. K. Etherington, A. P. Monkman, *J. Mater. Chem. C* **2018**, *6*, 4842.

[16] a) M. Numata, T. Yasuda, C. Adachi, *Chem. Commun.* **2015**, *51*, 9443; b) K. Suzuki, S. Kubo, K. Shizu, T. Fukushima, A. Wakamiya, Y. Murata, C. Adachi, H. Kaji, *Angew. Chem. Int. Ed.* **2015**, *54*, 15231; c) Y. Kitamoto, T. Namikawa, T. Suzuki, Y. Miyata, H. Kita, T. Sato, S. Oi, *Org. Electron.* **2016**, *34*, 216.

[17] a) W. J. Li, Y. Y. Pan, L. Yao, H. C. Liu, S. T. Zhang, C. Wang, F. Z. Shen, P. Lu, B. Yang, Y. G. Ma, *Adv. Opt. Mater.* **2014**, *2*, 892; b) Y. R. Jiang, Z. B. Hu, B. Zhou, C. Zhong, Z. R. Sun, H. T. Sun, *J. Phys. Chem. C* **2019**, *123*, 5616.

[18] a) P. V. Rysselberghe, *J. Phys. Chem.* **1931**, *36*, 1152; b) P. K. Nayak, N. Periasamy, *Org. Electron.* **2009**, *10*, 532.

[19] a) C. C. Pye, T. Ziegler, *Theor. Chem. Acc.* **1999**, *101*, 396; b) A. Klamt, G. Schuurmann, *J. Chem. Soc. Perk. Trans. 2* **1993**, 799; c) A. Klamt, *J. Phys. Chem.* **1995**, *99*, 2224.

[20] S. J. A. van Gisbergen, J. G. Snijders, E. J. Baerends, *J. Chem. Phys.* **1998**, *109*, 10644.

[21] M. Seth, T. Ziegler, *J. Chem. Theory Comput.* **2012**, *8*, 901.

[22] E. Van Lenthe, E. J. Baerends, *J. Comput. Chem.* **2003**, *24*, 1142.

[23] a) J. C. Slater, *Quantum Theory of Molecules and Solids*, 4 ed., McGraw-Hill, New York, **1974**; b) A. D. Becke, *J. Chem. Phys.* **1986**, *84*, 4524; c) T. V. Russo, R. L. Martin, P. J. Hay, *J. Chem. Phys.* **1994**, *101*, 7729; d) C. Lee, W. Yang, R. G. Parr, *Phys. Rev. B* **1988**, *37*, 785.

[24] a) E. v. Lenthe, E. J. Baerends, J. G. Snijders, *J. Chem. Phys.* **1993**, *99*, 4597; b) E. van Lenthe, E. J. Baerends, J. G. Snijders, *J. Chem. Phys.* **1994**, *101*, 9783; c) E. van Lenthe, J. G. Snijders, E. J. Baerends, *J. Chem. Phys.* **1996**, *105*, 6505.

[25] L. Kleinman, *Phys. Rev. B* **1997**, *56*, 16029.

[26] Z. Hu, B. Zhou, Z. Sun, H. Sun, *J. Comput. Chem.* **2017**, *38*, 569.

[27] a) X. K. Chen, S. F. Zhang, J. X. Fan, A. M. Ren, *J. Phys. Chem. C* **2015**, *119*, 9728; b) X. K. Chen, D. Kim, J. L. Brédas, *Acc. Chem. Res.* **2018**, *51*, 2215; c) J. Gibson, A. P. Monkman, T. J. Penfold, *ChemPhysChem* **2016**, *17*, 2956; d) C. M. Marian, *J. Phys. Chem. C* **2016**, *120*, 3715; e) F. B. Dias, J. Santos, D. R. Graves, P. Data, R. S. Nobuyasu, M. A. Fox, A. S. Batsanov, T. Palmeira, M. N. Berberan-Santos, M. R. Bryce, A. P. Monkman, *Adv. Sci.* **2016**, *3*, 1600080; f) M. K. Etherington, J. Gibson, H. F. Higginbotham, T. J. Penfold, A. P. Monkman, *Nat. Commun.* **2016**, *7*, 13680; g) T. Hosokai, H. Matsuzaki, H. Nakanotani, K. Tokumaru, T. Tsutsui, A. Furube, K. Nasu, H. Nomura, M. Yahiro, C. Adachi, *Sci. Adv.* **2017**, *3*, e1603282.

[28] A. J. Lennox, G. C. Lloyd-Jones, *Chem. Soc. Rev.* **2014**, *43*, 412.

[29] X. H. Shan, B. Yang, H. X. Zheng, J. P. Qu, Y. B. Kang, *Org. Lett.* **2018**, *20*, 7898.

[30] S. Konishi, T. Iwai, M. Sawamura, *Organometallics* **2018**, *37*, 1876.

[31] M. W. Drover, K. Nagata, J. C. Peters, *Chem. Commun.* **2018**, *54*, 7916.

[32] K. Schickedanz, T. Trageser, M. Bolte, H. W. Lerner, M. Wagner, *Chem. Commun.* **2015**, *51*, 15808.

[33] K. Schickedanz, J. Radtke, M. Bolte, H. W. Lerner, M. Wagner, *J. Am. Chem. Soc.* **2017**, *139*, 2842.

[34] X. Yin, J. Chen, R. A. Lalancette, T. B. Marder, F. Jakle, *Angew. Chem. Int. Ed.* **2014**, *53*, 9761.

[35] Z. Zhang, R. M. Edkins, J. Nitsch, K. Fucke, A. Steffen, L. E. Longobardi, D. W. Stephan, C. Lambert, T. B. Marder, *Chem. Sci.* **2015**, *6*, 308.

[36] K. Zhang, Q. Sun, Z. Zhang, L. Tang, Z. Xie, Z. Chi, S. Xue, H. Zhang, W. Yang, *Chem. Commun.* **2018**, *54*, 5225.

[37] N. Liu, B. Wang, W. W. Chen, C. L. Liu, X. Y. Wang, Y. F. Hu, *RSC Adv.* **2014**, *4*, 51133.

[38] S. Toyota, M. Asakura, M. Oki, F. Toda, *Bull. Chem. Soc. Jpn.* **2000**, *73*, 2357.

[39] M. Schlosser, F. Mongin, J. Porwisiak, W. Dmowski, H. H. Büker, N. M. M. Nibbering, *Chem. Eur. J.* **1998**, *4*, 1281.

[40] Z. Zhang, R. M. Edkins, M. Haehnel, M. Wehner, A. Eichhorn, L. Mailander, M. Meier, J. Brand, F. Brede, K. Müller-Buschbaum, H. Braunschweig, T. B. Marder, *Chem. Sci.* **2015**, *6*, 5922.

[41] M. Mantina, A. C. Chamberlin, R. Valero, C. J. Cramer, D. G. Truhlar, *J. Phys. Chem. A* **2009**, *113*, 5806.

[42] D. Reitzenstein, T. Quast, F. Kanal, M. Kullmann, S. Ruetzel, M. S. Hammer, C. Deibel, V. Dyakonov, T. Brixner, C. Lambert, *Chem. Mater.* **2010**, *22*, 6641.

[43] a) N. G. Connelly, W. E. Geiger, *Chem. Rev.* **1996**, *96*, 877; b) D. Tsiplakides, D. Archonta, C. G. Vayenas, *Top. Catal.* **2007**, *44*, 469.
